# Supplementary material for: Hierarchical organic microspheres from diverse molecular building blocks
Source: Nat Commun. 2024 Jun 13;15:5041. doi: 10.1038/s41467-024-49379-7 (PMC11176358; doi:10.1038/s41467-024-49379-7)
Supplement: Supplementary file 1 — Supplementary Information [file 41467_2024_49379_MOESM1_ESM.pdf]

Supplementary Information for

Hierarchical Organic Microspheres from Diverse Molecular Building Blocks

Yintao Li[1], Longlong Fan[2], Xinyan Xu[3], Yang Sun[1], Wei Wang[1], Bin Li[3], Samuel S.Veroneau[4], Pengfei Ji[1*]

[1]Department of Chemistry, Zhejiang University, Hangzhou, 310058, China

[2]Institute of High Energy Physics, the Chinese Academy of Sciences, Beijing, 100049, China

[3]College of Agriculture and Biotechnology, Zhejiang University, Hangzhou, 310058, China

[4]Department of Chemistry and Chemical Biology, Harvard University, Cambridge, Massachusetts, 02138, United States

*Corresponding author. Email: jipengfei@zju.edu.cn

Content

General information **2**

Reagents 2

Supplementary Methods **2**

Supplementary Method 1: General synthetic procedure of HOMs 2

Supplementary Method 2: 96-wells plates based high-throughput screening 3

Supplementary Method 3: The synthesis of the triple condensation product 3

Supplementary Method 4: MD simulation 3

Supplementary Method 5: PXRD structure solution and refinement 4

Supplementary Method 6: Analysis of precipitation kinetics 5

Supplementary Method 7: Retention curves of HOM-1 in alkaline solution 6

Supplementary Method 8: Surface modelling of HOMs 6

Supplementary Method 9: The Delaunay triangulation method 7

Supplementary Method 10: The radial distribution function method 8

Supplementary Method 11: Protein immobilization on HOMs.. 9

Supplementary Figure 10

Supplementary Table 69

Supplementary Table 1: Synthetic conditions for HOMs 69

Supplementary Table 2: HOMs growth and characteristics data index 70

Supplementary Table 3: Comparison of HRP loading capacity in this work to other reported carriers... 71

Supplementary References 72

General information

Reagents

2,4,6-trihydroxy benzene-1,3,5-tricarbaldehyde (97%), pyridine-2,6-dicarbaldehyde (98%), 2-hydroxyisophthalaldehyde (98%), 4,6-dihydroxy-5-methylisophthalaldehyde (97%), furan-2,5-dicarbaldehyde (98%), 4-amino-3-ethylbenzoic acid (98%), 1-methylpyrrolidin-2-one (NMP), deuteroxide (D_2O, 99.9% D%) were purchased from Shanghai Bide Pharmatech Co., Ltd. 4-amino-1-naphthoic acid (98%), isophthalaldehyde (98%), 6-amino-2-naphthoic acid (97%), 4'-amino-[1,1'-biphenyl]-4-carboxylic acid (97%) were purchased from Shanghai Haohong scientific Co., Ltd. 4-aminobenzoic acid (99%), N,N-dimethylacetamide (DMAc, >99.8%), 1-methylpyrrolidin-2-one (98%), sodium deuteroxide (NaOD, 99.5% D%, 40% in D_2O) and TMB liquid substrate solution were purchased from Shanghai Macklin Biochemical Co., Ltd. Dimethyl sulfoxide (DMSO), N,N-dimethylformamide (DMF), tetrahydrofuran (THF), toluene, ethanol (EtOH), isopropanol (iPrOH), acetonitrile (MeCN), ethyl acetate, 1,4-dioxane, toluene, acetone, dichloromethane (DCM) were purchased from Sinopharm Chemical Reagent Co., Ltd. N,N-diethylformamide (DEF) and methanol (MeOH) were purchased from Shanghai Titan Co., Ltd. All reagents were used as received without further purification.

Supplementary Methods

Supplementary Method 1: General synthetic procedure of HOMs

2,4,6-Trihydroxy benzene-1,3,5-tricarbaldehyde (100 mg, 475.9 μmol) was dissolved in 25 mL DMAc and 4-aminobenzoic acid (131 mg, 955.2 μmol) was dissolved in 25 mL iPrOH. After directly mixing the two solutions, a clear yellow solution was obtained. Precipitate occurred after several minutes which was isolated by centrifugation after 1 hr. The precipitate was washed with iPrOH three times to remove the excess amine and

residue DMAc. The final products (4,4'-(5-(hydroxymethylene)-2,4,6-trioxocyclohexane-1,3-diylidene)-bis(methaneylylidene))-bis(azanediyl))dibenzoic acid, **3**) was dried in a vacuum oven at 40 °C and 1 Pa. The amount of product was measured to be 180 mg in 84% yield. The synthetic procedures of other HOMs were similar to HOM-1, replacing 2,4,6-trihydroxy benzene-1,3,5-tricarbaldehyde with altered dialdehyde reactants and 4-aminobenzoic acid with altered amine reactants. Besides, the choice of aprotic solvent (solvent A) and protic solvent (solvent B) will also influence the final assembly structure. Solvents A and B used for the synthesis of HOM-2–16 are listed in the Supplementary Table 1.

Supplementary Method 2: 96-wells plates based high-throughput screening

The aldehyde was dissolved in THF (20 mM) and dispensed into a 96-well plate (100 μL per well). Following this, THF was removed using a nitrogen evaporator. Subsequently, 10 types of solvent A were added to each well. The amines, dissolved in 8 types of solvents B (40 mM), were then introduced into specific wells corresponding to the respective aldehyde solutions. By examining the state of the aggregations on the bottom of each well, the morphologies of assemblies could be determined to choose the optimal solvent combinations.

Supplementary Method 3: The synthesis of the triple condensation product

The triple condensation product, 4,4',4''-(((2,4,6-trioxocyclohexane-1,3,5-triylidene)tris(methaneylylidene))tris(azanediyl))tribenzoic acid (TACT, **3**) was also synthesized. 2,4,6-trihydroxy benzene-1,3,5-tricarbaldehyde (100 mg, 475.9 μmol) and 4-aminobenzoic acid (196 mg,1.43 mmol) were dissolved in 50 mL DMSO with stirring at room temperature for 24 hrs. 50 mL EtOH was added to the reaction solution, resulting in a cloudy orange precipitate. The final product was washed with EtOH and dried in a vacuum oven at 40 °C and 1 Pa.

Supplementary Method 4: MD simulation

If not specified otherwise, molecular models were optimized using DFT calculations using the M06-2X functional and the 6-311+G(d,p) basis set for all atoms in the Gaussian 16 software package[1,2]. MD simulations were conducted using the GROMACS 2020.1 package with the General AMBER Force Field (GAFF). Following a brief energy minimization, the NPT ensemble was employed with a time step of 1 fs. Bonds involving hydrogen atoms were constrained using the LINCS algorithm[3]. The temperature was maintained at 298 K using the velocity-rescaling thermostat with a relaxation time of τ_T = 1.0 ps, while the pressure was kept at 1.0 bar using the Berendsen barostat with a relaxation time of τ_p = 1.5 ps. Van der Waals and electrostatic forces were truncated at a distance of 1.4 nm using the Verlet list scheme, and long-range electrostatic interactions were treated utilizing the Barker–Watts reaction field with ε_{RF} = 62. The VMD program v. 1.9.3 was used for visualization of the simulations.

To understand the self-assembly modes of monomeric molecules at the nanoscale, we performed MD simulations on randomly placed BACT in a mixed solvent system with

methanol and DMSO. In the actual reaction system, the stoichiometric ratio between the monomer and solvent is approximately 1:5600 (20mM). In the simulation system, we increased the concentration by approximately 95-fold to increase the rate of aggregation. So, 100 BACT, 2978 DMSO, and 2978 MeOH molecules were randomly placed in a 10 nm × 10 nm × 10 nm box. During the simulation, more than 80% of molecules maintained planar configurations (Supplementary Fig 5). Thus, we categorized the molecular interactions observed in the snapshots of MD into two types: lateral interactions (within the molecular plane) and axial interactions (perpendicular to the molecular plane). The axial interactions were predominantly governed by π-π stacking. The lateral interactions are mainly composed of hydrogen-bonding interaction. Among all the functional groups, the terminal carboxyl group can act as both a hydrogen-bond acceptor and donor, while the enamine and enol groups can only serve as hydrogen-bond donors. The carbonyl group on the central aromatic ring can act as a hydrogen-bond acceptor. The carboxyl group can form hydrogen bonds with enol/enamine and its neighboring carbonyl groups. The enol group with its neighboring carbonyl group can also interact with the same part from another molecule. Due to its location in the central region of the molecule, the enamine group is spatially constrained and can only interact with the terminal carboxyl group. To make quantitative comparisons, considering only intermolecular interactions within two molecules, we performed DFT structure optimizations and calculated the binding energies for different types of hydrogen bonding patterns. The binding energy was calculated by the equation follow.

$$E_{BE} = E_{complex} - E_{separated} \qquad \text{(S1)}$$

E_{BE} refers to the binding energy. $E_{complex}$ refers to the single point energy of the molecular fragments containing two BACT molecules, considering the occurrence of potential intermolecular interactions. $E_{separated}$ is based on the sum of the single point energy of two separated BACT molecules.

The hydrogen bonding patterns and energies for various patterns are listed in Supplementary Fig. 6. It can be observed that the classical hydrogen bonding between carboxylic acids exhibits the highest association energy (–28.43 kcal mol^{-1}). However, as mentioned earlier, when the carboxyl group is engaged in carboxyl-carboxyl bonding, the enamine group cannot participate in hydrogen bonding. Consequently, a molecule can only interact with three other molecules through hydrogen bonding, resulting in relatively higher total binding energy. Conversely, if the carboxyl group forms a hydrogen bond with the enamine group, a molecule can potentially interact with up to five other molecules, leading to an optimized total binding energy. In our calculations, the former scenario yielded a total binding energy of –31.13 kcal mol^{-1}, while the latter reached –54.24 kcal mol^{-1}. So, to achieve a lower total interaction energy, interactions between carboxyl and amino groups are preferred.

Supplementary Method 5: PXRD structure solution and refinement

The powder diffraction patterns were collected at National Synchrotron Radiation

Laboratory at University of Science and Technology of China with a wavelength of 0.67092 Å. The measured samples were synthesized following standard conditions and were evaporated under atmospheric pressure and room temperature.

The structure solution was performed with the Materials Studio 2017 software on the powder XRD data. The result indicated a tetragonal unit cell with three edges of around 23.05 Å, 23.05 Å and 3.94 Å. Considering the chirality of the entangled fibrous morphology of the material observed under the electron microscope, the space group should be one of the Sohncke groups that have only symmetry operations of the first kind. The extinction condition of the space group P4 shows the best match with the observed PXRD data[4,5]. Unit cell parameters, sample displacement, background, and profile parameters were refined by Pawley refinement and fixed in the subsequent structure solution process, where the simulated annealing method built in Materials Studio was used[6]. The BACT was modelled as a rigid body with flexibilities on bond angles and torsion angles. After the reasonable structure model emerged, geometry optimization was performed with Forcite Module using COMPASS II force field. In the final structure, the carboxyl group indeed interacts with the enamine moiety, while the enol group forms a hydrogen bonding network with the enol group of another molecule. From a projection perspective, one molecule interacts with five adjacent molecules, consistent with the previous analysis. To assess the rationality of the obtained structure, we performed MD simulations on selected portions of the structure and found that there is a slight misalignment between different layers. Combining with the difference between the primary crystal structure and the actual crystal structure, we expanded the unit cell by multiplication of the primitive cell. Herein, the GSAS2 software was employed for the structural refinements by the Rietveld method[7], and rigid bonds were introduced into the refinement in order to restrict structural degrees of freedom. Besides, the background of the diffraction pattern was fitted by a Chebyschev function with 25 parameters. The Rwp factor was 7.273%. (Supplementary Fig. 7).

Supplementary Method 6: Analysis of precipitation kinetics

The precipitation kinetics were determined by turbidity measurement (OD_{600}). The OD_{600} was measured by the Thermo Scientific Varioskan LUX multimode microplate reader. A mixture of 100 μL of TP DMAc solution (20 mM) and 100 μL of ABA dispersed in different solvents (40 mM) was prepared in a quartz microplate. The turbidity was immediately measured, and subsequent turbidity readings were taken every 20 s. There is a dead time of approximately 16 s associated with instrument startup (from mixing solvents to measuring the first data point).

In the experiment investigating the influence of different solvent ratios on precipitation kinetics, slight variations were made in the synthetic conditions. TP and ABA were dispersed in DMAc and iPrOH, respectively, with concentrations of 100 mM and 200 mM. 20 μL of TP solution was diluted with a certain amount of DMAc and iPrOH according to the specific condition, followed by the addition of 20 μL of ABA solution. The subsequent testing conditions remained consistent with those described earlier.

In a series of concentration gradients, TP and ABA were dissolved in DMAc and iPrOH, respectively. The concentration of TP solutions was set at 5, 10, 20, and 40 mM, with the corresponding ABA solution concentrations being twice that of TP. After mixing 100 μL of TP and ABA solutions at their respective concentrations, the subsequent testing methods for measuring precipitation kinetics remained consistent with the ones previously employed.

In the investigation of the impact of the stoichiometric ratio between TP and ABA, the aldehyde and amine were dissolved in DMAc and iPrOH, respectively. Mixing equal volumes (100 μL) of 40 mM TP with 40 mM ABA, 20mM TP with 40 mM ABA, or 20mM TP with 60mM ABA solutions, the remaining methods for measuring precipitation kinetics were consistent with the methods previously utilized

Supplementary Method 7: Retention curves of HOM-1 in alkaline solution

The retention curves were recorded in the past study by measuring the absorbance of the supernatant at a wavelength of 400 nm using the Thermo Scientific Varioskan LUX multimode microplate reader. Specifically, 10 μL of a 10 mg mL^{-1} HOM-1 suspension was added to 990 μL of a phosphate buffer solution (7 mM, based on phosphate anion) at different pH levels. After 10 min oscillation, the mixture underwent centrifugation to separate the precipitate. Following this, the absorbance of the supernatant at 400 nm was measured. Water was used as a blank for absorbance at 400 nm, and the group with the highest absorbance (indicating complete decomposition with no remaining precipitate) was considered as the reference absorbance. The obtained absorbance values were then normalized. To record the retention curves of the metal ions modification groups, 10 μL of a 10 mg mL^{-1} HOM-1 suspension was added with either 495 μg $CaCl_2$ or 907 μg $MgCl_2 \cdot 6H_2O$.

Supplementary Method 8: Surface modelling of HOMs

The SEM images of HOMs were processed using ImageJ. The particles were approximated as ellipsoids in the conducted study. The major axis, intermediate axis, and minor axis of the ellipsoid were denoted as A, B, and C, respectively. A coordinate system was constructed with the major axis of the ellipsoid as the x-axis, the minor axis as the y-axis, and the intermediate axis as the z-axis. In ImageJ, the default coordinate origin for a certain SEM image was positioned at the top left corner of the image. The length of the image was parameterized using a scale bar, enabling the marking of longitudinal and transverse boundaries of the particles by selecting the topmost/bottommost points (maximum and minimum values of y) and two side points (maximum and minimum values of x) within each particle. Subsequently, the coordinates of all selected tip vertices and boundary-defining points were exported. The centre point of the ellipsoid was determined as the intersection point between lines connecting the maximum/minimum points on the y-axis and lines connecting the maximum/minimum points on the x-axis. The coordinates of this intersection point were represented as (a, b, 0). The difference between the maximum and minimum values on the x-axis was recorded as 2A, while the difference between the maximum

and minimum values on the y-axis was calculated as 2B. It was assumed that C had a consistent value with B. Based on these parameters, we constructed the surface equation of HOMs as follows.

$$\frac{(x-a)^2}{A}+\frac{(y-b)^2}{B}+\frac{z^2}{C}=1 \quad \text{(S2)}$$

Here, (x, y, z) was the coordinate of a point. (a, b, 0) was the centre coordinates of the ellipsoid. A, B, and C were the major axis, intermediate axis, and minor axis of the ellipsoid, respectively.

For echinate microspheres, the vertices of the particle surface tips were marked in ImageJ. For striated microspheres, the intersection points were also marked in ImageJ. Based on the coordinate system established above, we could obtain the coordinates for all the points. We processed the obtained data using MATLAB R2021a. Based on the surface model of HOMs, we computed the z-axis coordinates for all points. The code for estimation of the Z-coordinate value is shown in Supplementary Code 2.

Supplementary Method 9: The Delaunay triangulation method

In the closest packing mode, when the tips are distributed on the surface of the sphere, the maximum radius of particles that could diffuse and come into contact with the surface of HOMs was found to be $2/\sqrt{3}$ times the distance from tip to tip (Supplementary Fig. 33a). The actual surface stacking model of tips in HOMs deviated from the ideal model, resulting in a theoretical value that lacked practical reference. In practical operations, we only considered the point spacing. Delaunay triangulation, a commonly used method in computer graphics and computational geometry, was employed to organize a given set of points into a non-overlapping network of triangles. This method is defined based on specific geometric properties, ensuring that the circumcircle of each triangle does not contain any other points. Delaunay triangulation possesses several useful characteristics, making it widely applicable across various domains. The Delaunay triangulation exhibits the following properties[8]:

1. Maximization of minimum angle: In a Delaunay triangulation, the minimum interior angle of all triangles is maximized as much as possible. This helps reduce distortion and stretching, enhancing the quality of the generated triangular mesh.

2. Minimization of edge length differences: The differences in edge lengths between adjacent triangles are minimized as much as possible, aiding in maintaining uniformity and consistency in the mesh.

3. Uniqueness: For a given set of points, there exists a unique Delaunay triangulation.

For the Delaunay triangulation method, using the point set in the x-y projection plane, we employed the built-in Delaunay module in MATLAB R2021a to perform triangulation and obtain the corresponding point connectivity. Subsequently, taking into account the z-axis coordinates, we calculated the average length of all triangle edges.

However, in practical operations, boundary errors may occur, where two points that are originally not neighbouring are directly connected during the partition process. Hence, we need to filter out such erroneously included edges in our analysis. Based on the assumption mentioned earlier, we first consider the average length of all edges in the triangulated point set and calculate the first average. The threshold value will be √3 times the first average and all the larger lengths will be filtered (Supplementary Fig. 33b). Because, in the closest packing model, the distance from a tip to its second nearest tip is √3 times the shortest distance. Then, we calculate the second average of the remaining length and adopt it as the final result (Supplementary Fig. 31). The code for Delaunay triangulation is shown in Supplementary Code 3.

Supplementary Method 10: The radial distribution function method

The radial distribution function (RDF) is a statistical measure of the distance distribution between particles. It measures the probability density of finding other particles around a particular particle within a certain distance range. The RDF is defined as the ratio of the number density of particle pairs at a distance *r* to the reference particle number density in a unit volume. It is typically represented as *g(r)* and can be calculated using the following formula:

$$g(r) = \frac{1}{4\pi r^2 \rho} \langle \sum_{i \neq j}^{n} \delta(r - r_{ij}) \rangle \tag{S3}$$

Here, *r* is the distance, ρ is the reference particle number density, *N* is the total number of particles, r_{ij} is the distance between particle *i* and particle *j*, δ( ) is the Dirac delta function, and ⟨ ⟩ denotes averaging over all pairs of particles.

The RDF reflects the distribution of particles relative to a reference particle at different distances. When *g(r)* is equal to 1, it indicates that the distribution of particles at that distance is identical to that around the reference particle. When *g(r)* is greater than 1, it signifies a denser distribution of particles compared to the reference particle, while values less than 1 indicate a sparser distribution.

For practical processing, we calculated the pairwise distances between all points within the given point set, marked as *D*, regardless of whether they were neighboring. A maximum distance had been set according to the diameter of the largest particles which was denoted as r_{max}. We counted the number of distances that appear within a circular ring with an inner radius of *r* and a width of Δr and denoted it as *N(r)*, namely, the number of distances satisfying $r \leq D \leq r + \Delta r$. Also, the total number of distances within the maximum distance was counted as *N* (Supplementary Fig. 35a). Then, using the obtained data, we calculated the radial distribution function, denoted as *g(r)*, using the following formula.

$$g(r) = \frac{N(r) \cdot {r_{max}}^2}{N \cdot (\,2r\Delta r + \Delta r^2)} \tag{S4}$$

Here, r_{max} was the maximum distance, *r* was the inner diameter of the annulus, Δr was

the width of the annulus, *N* was the total number of distances shorter than the maximum distance, and *N(r)* is the number of distances satisfying $r \leq D \leq r + \Delta r$.

The corresponding *r* and *g(r)* data were imported into Origin for smoothing processing. Subsequently, the first peak value data was read, which represents the point spacing calculated based on the RDF method. In this method, it is challenging to obtain standard deviation information; therefore, only one peak value data is marked. The code for RDF is shown in Supplementary Code 4.

Supplementary Method 11: Protein immobilization on HOMs

HOMs were suspended in water to obtain a 10 mg ml^{-1} HOMs suspension. 1 mL suspension was taken and a certain volume of 10 mg mL^{-1} HRP solution was added. The samples were then oscillated on a metal bath at 10 °C for 12 hrs. Subsequently, the samples were centrifuged at 10,000 x g for 3 minutes, and the supernatant was collected. SDS-PAGE analysis was performed. The samples were diluted to the proper concentration before being mixed with a 6× protein loading buffer. Gel electrophoresis was performed with prefabricated gel using Tris-MOPS-SDS running buffer. Thermo Scientific PageRuler Prestained Protein Ladder is a mixture of 10 blue-, orange-, and green-stained proteins (10 to 180 kDa) for use as size standards in protein electrophoresis. The gels were stained by Genscript eStain L1 Protein Staining Device. The images of gels were recorded by Tanon 5200Multi. To investigate the potential influence of metal cations on the adsorption of HRP onto HOM-5, 10 μL of a 49.5 mg mL^{-1} $CaCl_2$ solution was simultaneously added to the protein adsorption system. The remaining procedures were conducted consistently.

Supplementary Figure

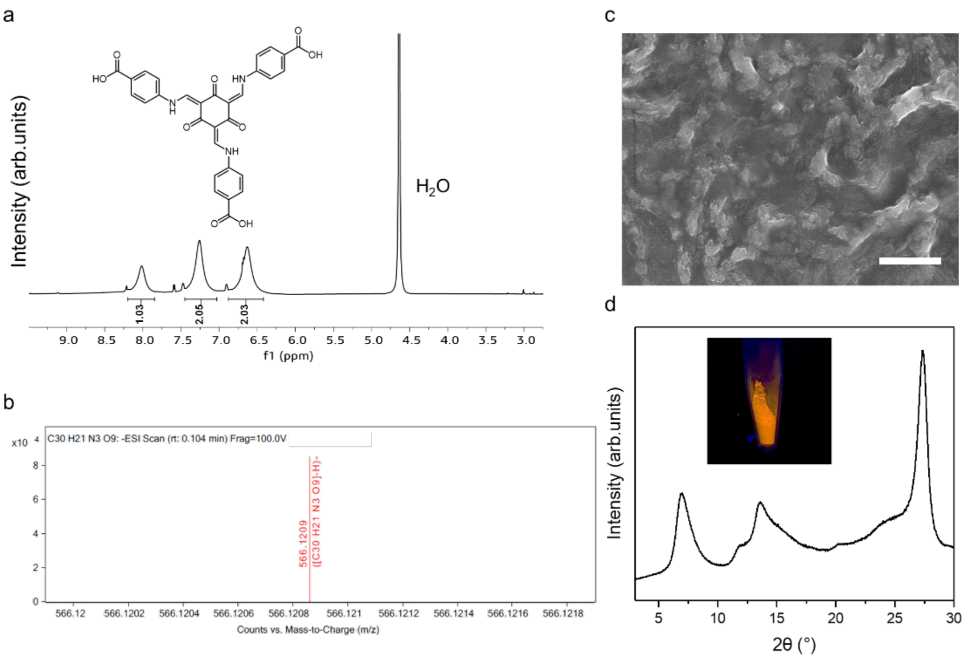

Supplementary Fig. 1 | a ^{1}H NMR spectrum of TACT. (5 mg in 23 mM NaOD in D_2O). **b** HRMS of the TACT. **c** SEM of TACT precipitate（scale bar of 1 μm). **d** The PXRD pattern for the TACT precipitate. The inserted image is the photo of the TACT powder emitting an orange fluorescence under the irradiation of 365 nm light.

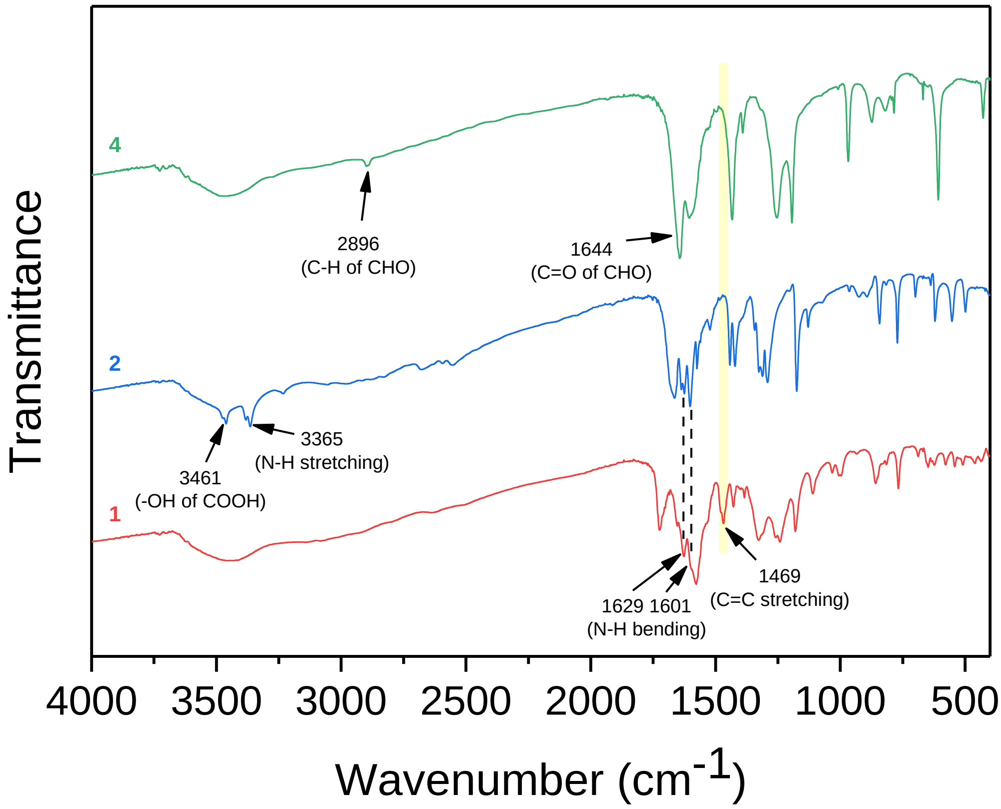

Supplementary Fig. 2 | Comparison of FT-IR spectra between as-synthesized HOM-1 (**4**) with the starting reactants (**1** and **2**). The right part is the enlargement of the spectra between 1000-1980 cm^{-1}. Due to the isomerization of the imine in HOM-1, the traditional imine stretching adsorption peak (at around 1610 cm^{-1}) is replaced by peaks from amine similar to its original amine. The peak for the C–H stretching of the aldehyde group is also invisible for the same reason. Instead, there is a new signal of C=C stretching in HOM-1 which confirms the tautomerization after the condensation.

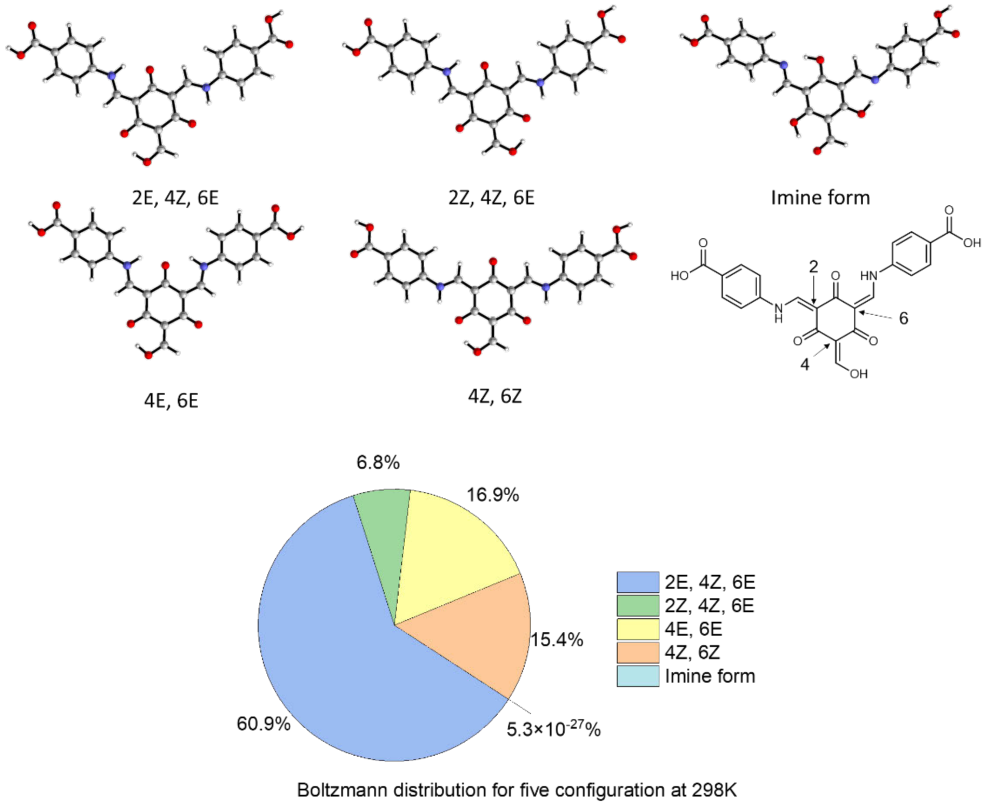

Supplementary Fig. 3 | Schematic diagrams of different conformations of BACT, along with their Boltzmann distributions at 298 K. DFT calculations using the B3LYP functional and the 6-311+G(d,p) basis set for all atoms in the Gaussian 16 software package.

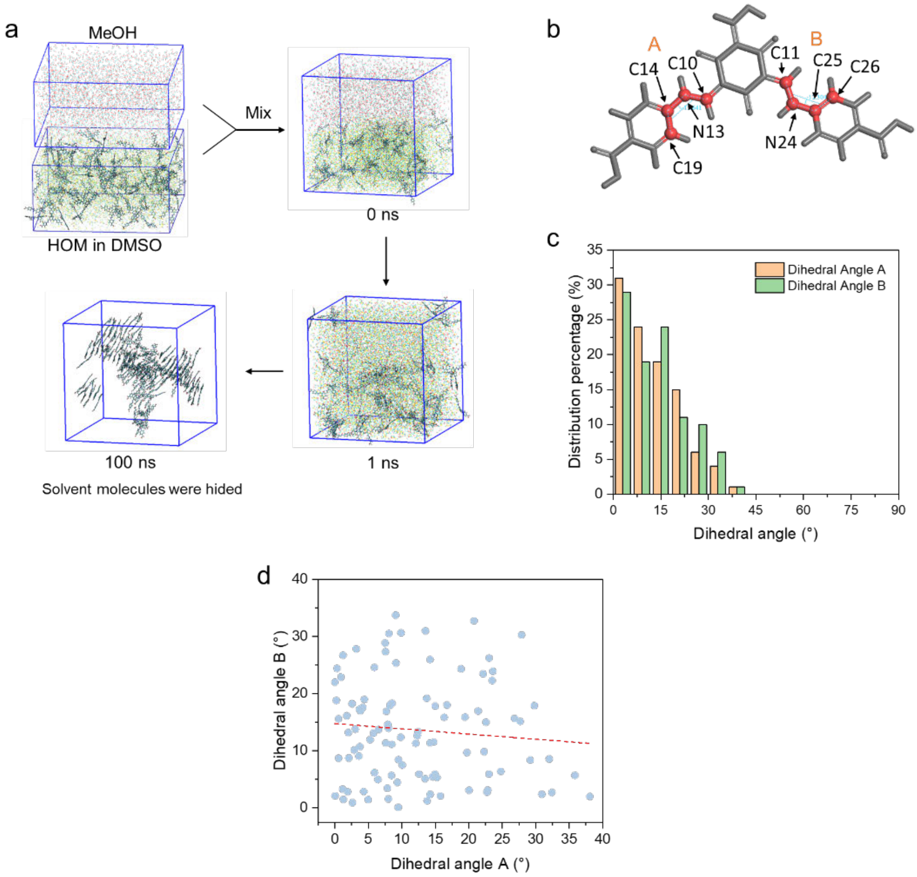

Supplementary Fig. 4 | a Snapshots from an MD simulation of 100 randomly placed BACT molecules in DMSO/methane (2978 molecules each). After 100 ns MD simulation, a tubular structure with distinct branching was formed. **b** Schematic diagram for dihedral angle calculation. The dihedral angle between the planes formed by C19, C14, and N13, as well as by C14, N13, and C10, was calculated. Acute angles were considered for statistical analysis. The same calculation method was applied to the other side, considering the dihedral angle between the planes formed by C11, N24, and C25 (dihedral angle A), and by N24, C25, and C26 (dihedral angle B). **c** Distribution percentage of the calculated dihedral angle. The frequencies (proportions) of the dihedral angles of 100 molecules in MD simulations falling into each grid, divided into 5° intervals from 0 to 90°, were calculated by MATLAB R2021a (Supplementary Code 1). For angles greater than 90°, their supplementary angle is referred to as the dihedral angle. Dihedral angles less than 20° are considered to indicate the planarity of the molecule. **d** The plot of all dihedral angles A/B within the same molecule. The coefficient of determination (R^2) is calculated to be 0.0083, indicating the independence of the two dihedral angles.

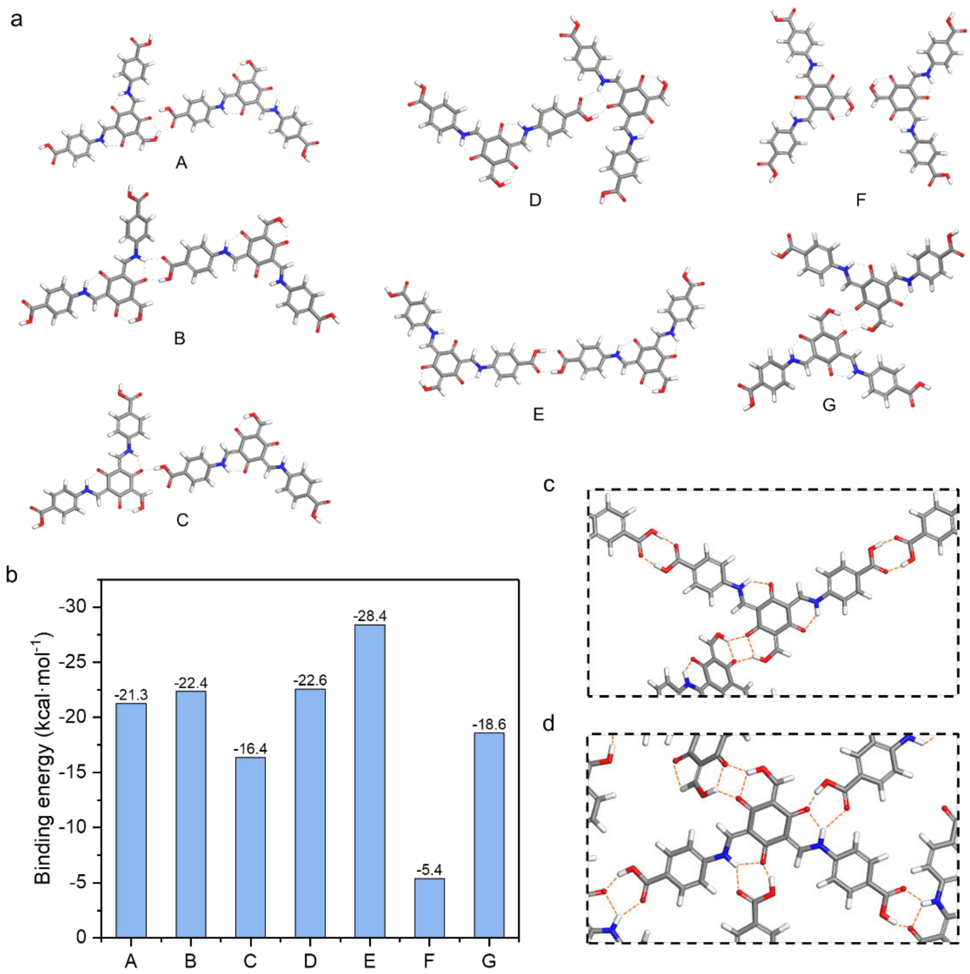

Supplementary Fig. 5 | a Bimolecular interaction mode within the plane of the BACT molecule. **b** The binding energy between two molecules in different binding modes. **c** A schematic diagram illustrating the interaction of one molecule with three surrounding molecules. **d** A schematic diagram illustrating the interaction of one molecule with five surrounding molecules. Hydrogen bonding interactions are shown in orange dashed line.

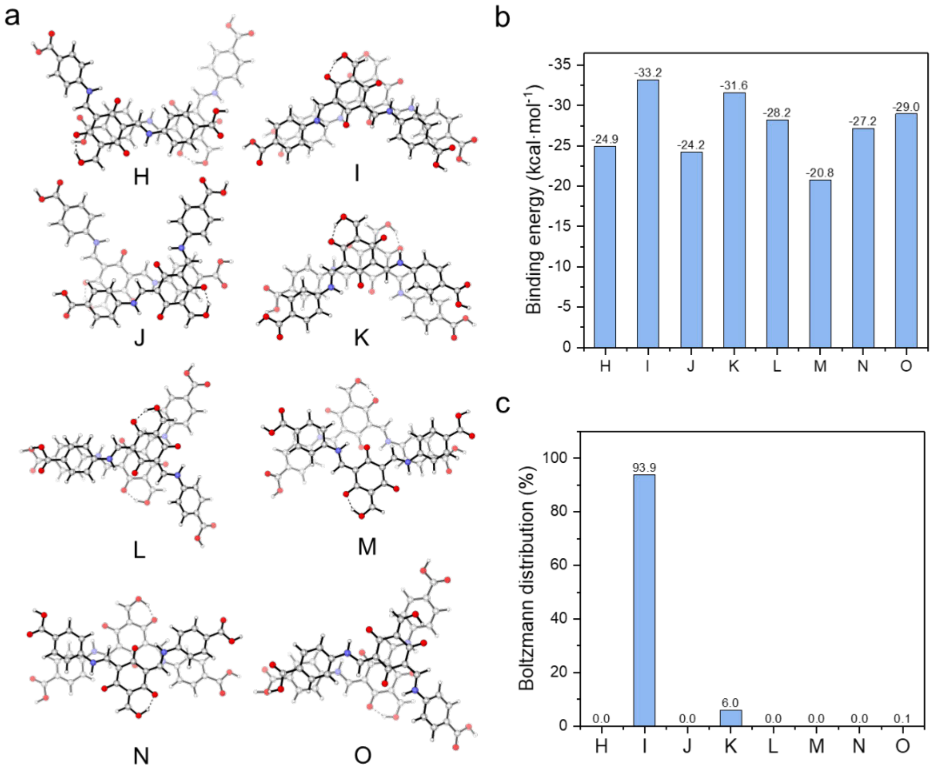

Supplementary Fig. 6 | a π-π stacking model of the BACT molecule. **b** The binding energy between two molecules in different stacking models. **c** The Boltzmann distributions of the π-π stacking models at 298 K

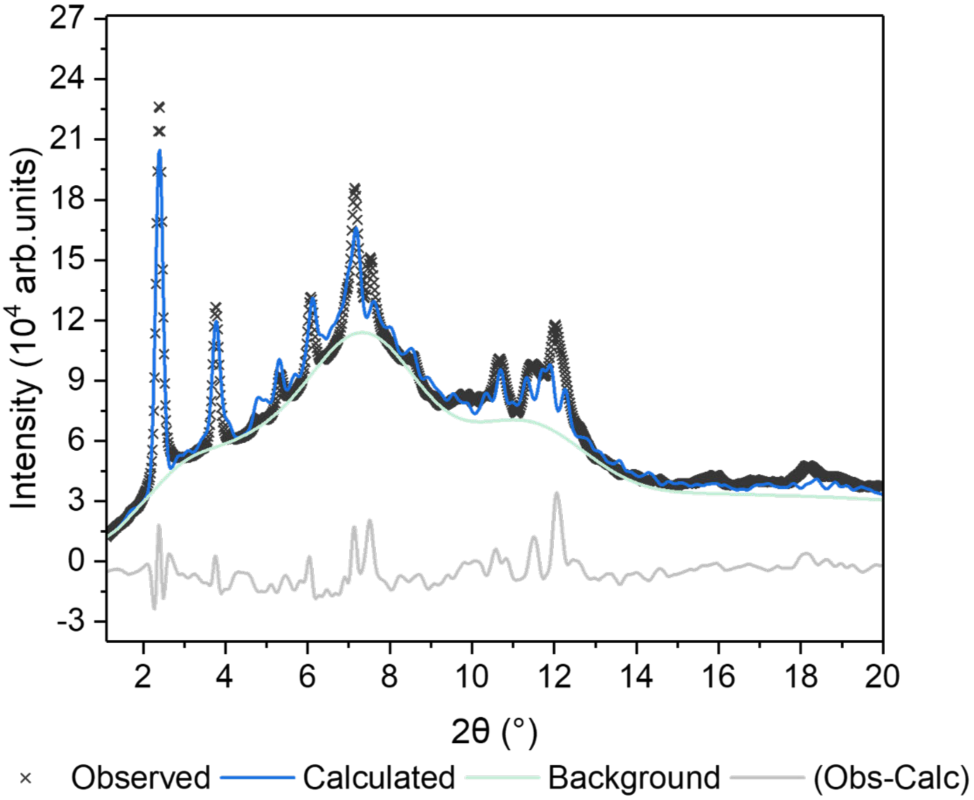

Supplementary Fig. 7 | The PXRD pattern for the HOM-1 sample (dark cross) and the ideal model (blue line).

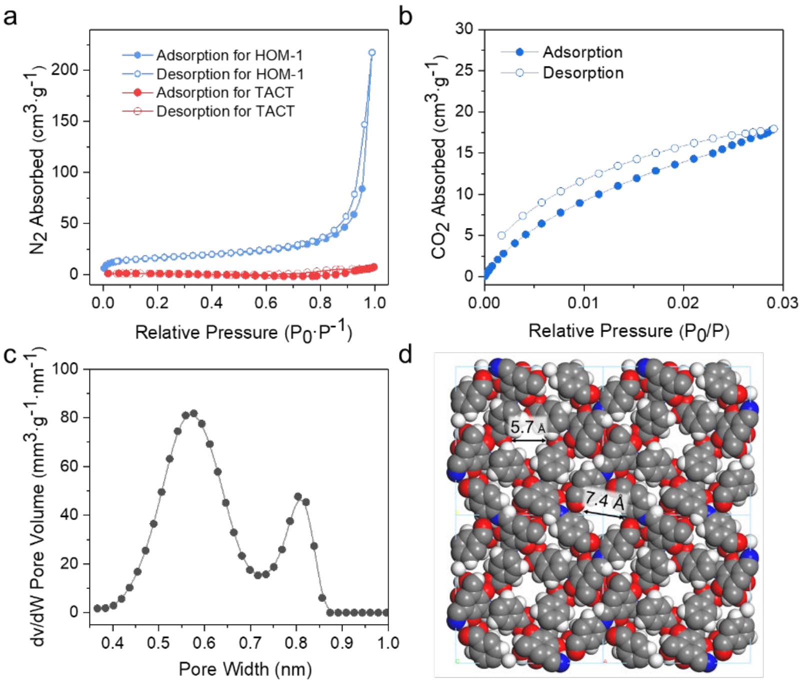

Supplementary Fig. 8 | a N_2 adsorption–desorption isotherms at 77 K of HOM-1 the precipitate of triple condensation product (TACT). From the isotherm, the surface area (S_{BET}) of the HOM-1 and TACT precipitate was calculated to be 55.8 $m^2\ g^{-1}$ and 3.0 $m^2\ g^{-1}$, respectively. Few pores in the TACT may have already deviated from the BET model. **b** CO_2 adsorption–desorption isotherms at 273 K of HOM-1. **c** Calculated pore size distribution plot of HOM-1 from CO_2 adsorption data at 273 K after DFT model fitting of adsorption branch data. **d** Schematic representation of the two main kinds of pore in the HOM-1 model.

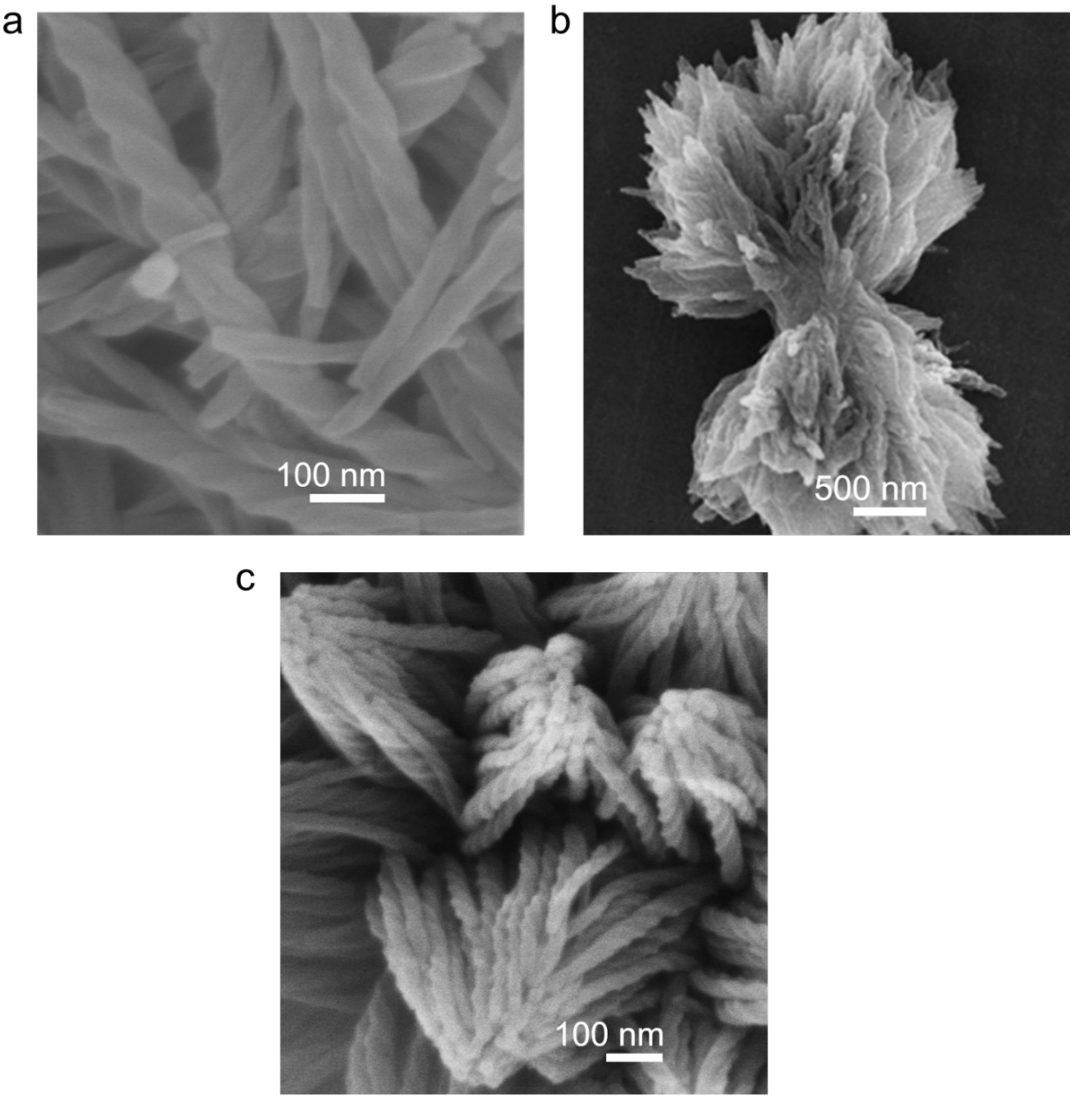

Supplementary Fig. 9 | Enlarged SEM images for several typical assembly morphologies, fibre (a), branching fibre (b), and tips (c).

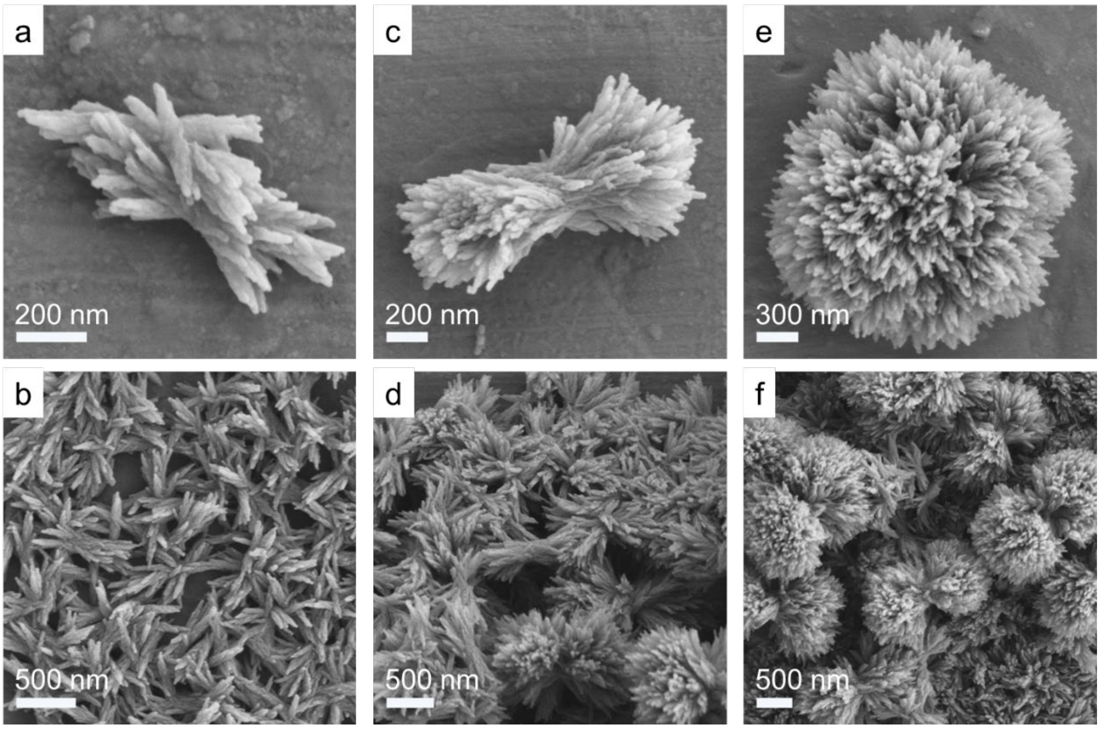

Supplementary Fig. 10 | SEM image of intermediates isolated at 90 s (a-b), 120 s (c-d), 150 s (e-f).

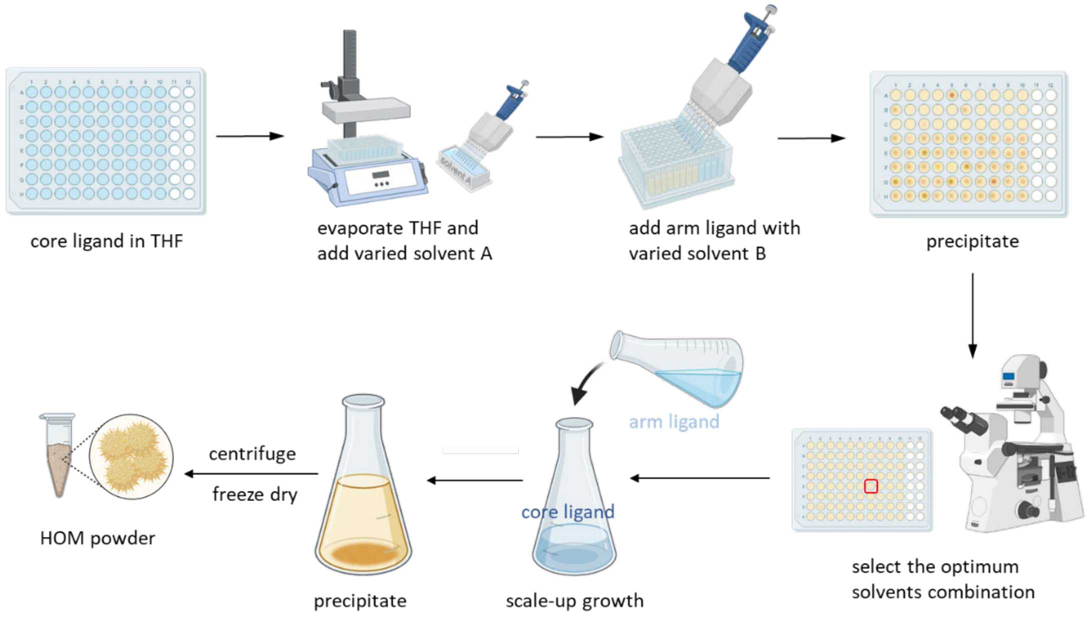

Supplementary Fig. 11 | The outline of the high-throughput screening scheme for optimizing the growth conditions of HOMs. The figure was created with BioRender.com released under a Creative Commons Attribution-NonCommercial-NoDerivs 4.0 International license.

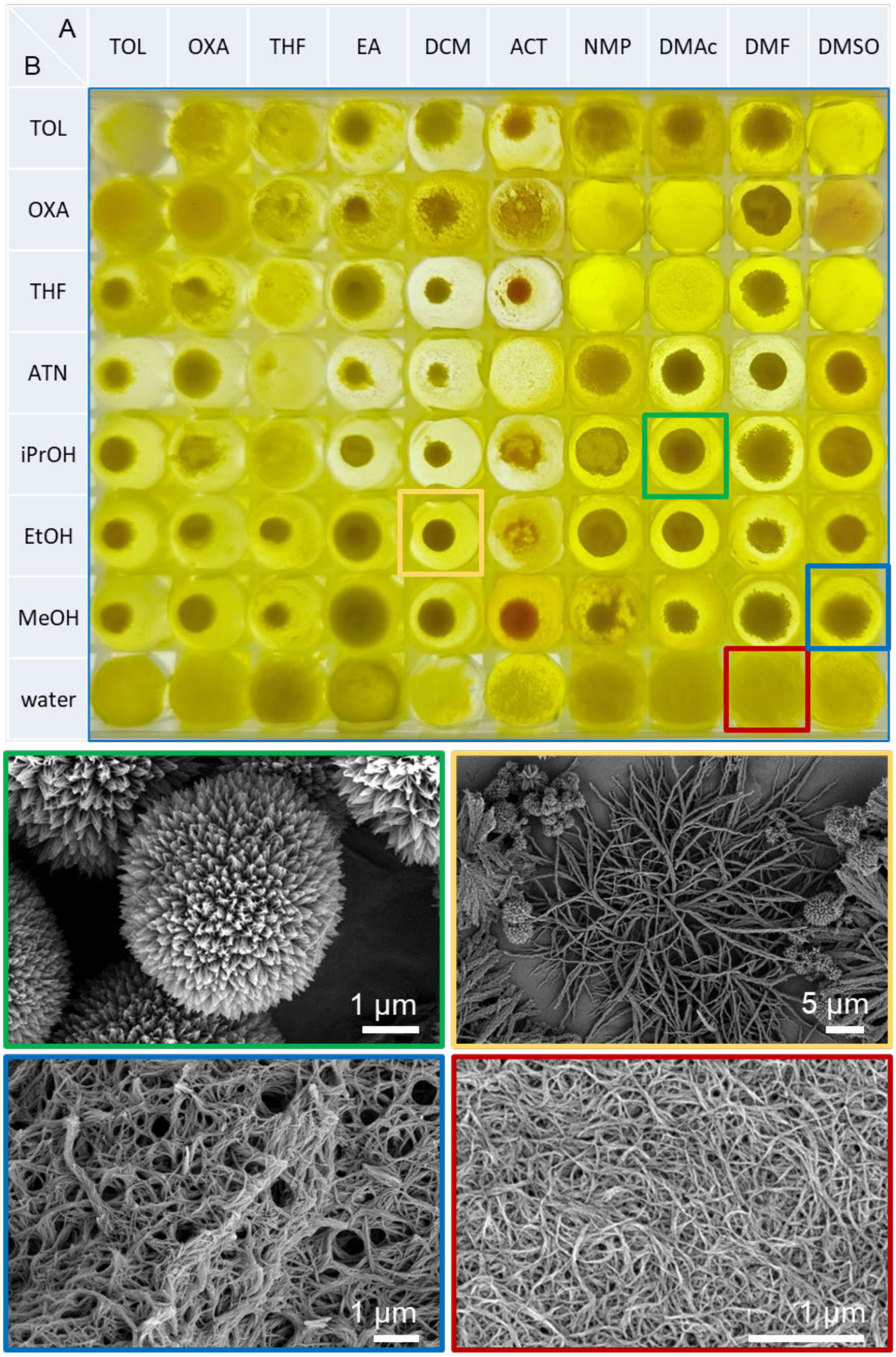

Supplementary Fig. 12 | The photo was taken during the high-throughput screening process for HOM-1. The dark sediment can be easily distinguished from the bright well with clear solutions. SEM images of selected samples are shown below. TP in different solvents A (20 mM, 100 μL) were mixed with ABA in different solvents B (40 mM, 100 μL). The photo was taken 30 minutes after mixing. Green frame: A = DMAc, B=iPrOH. Yellow frame: A = DCM, B = EtOH. Blue frame: A = DMSO, B = MeOH. Red frame: A = DMF, B = water.

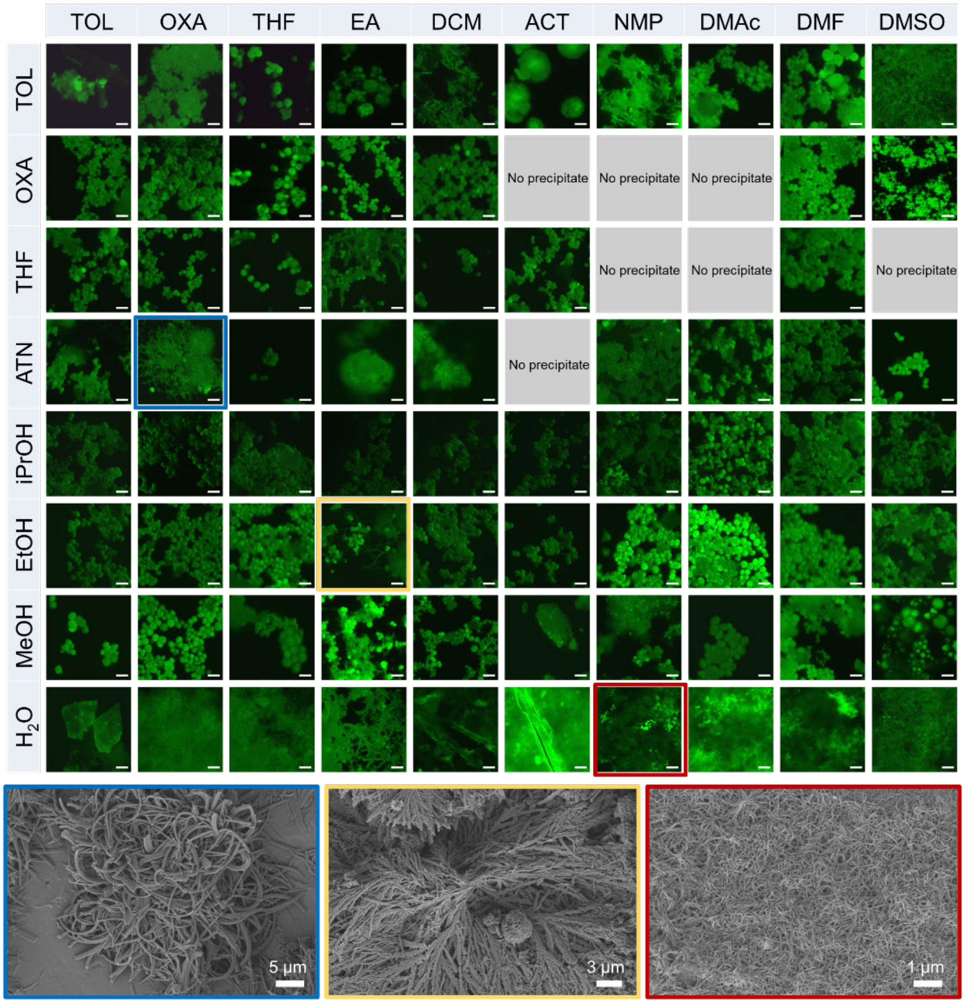

Supplementary Fig. 13 | The confocal images of HOM-1 grow in different solvent combinations. All samples were prepared by the above method: TP in solvents A (20 mM, 100μL) were mixed with ABA in solvents B (40 mM, 100μL). After 1 hr of reaction, the supernatant was removed by centrifugation, and the precipitate was deposited onto a glass slide. The sample was then observed using a laser scanning confocal microscope with a 20x objective lens (the scale bars are 100 μm, excitation wavelength: 488 nm and optical filter: 530nm/43, exposure time: 2.0 μs). Blue frame: A=1,4-dioxane, B=MeCN. Yellow frame: A=ethyl acetate, B=EtOH. Red frame: A=NMP, B=water.

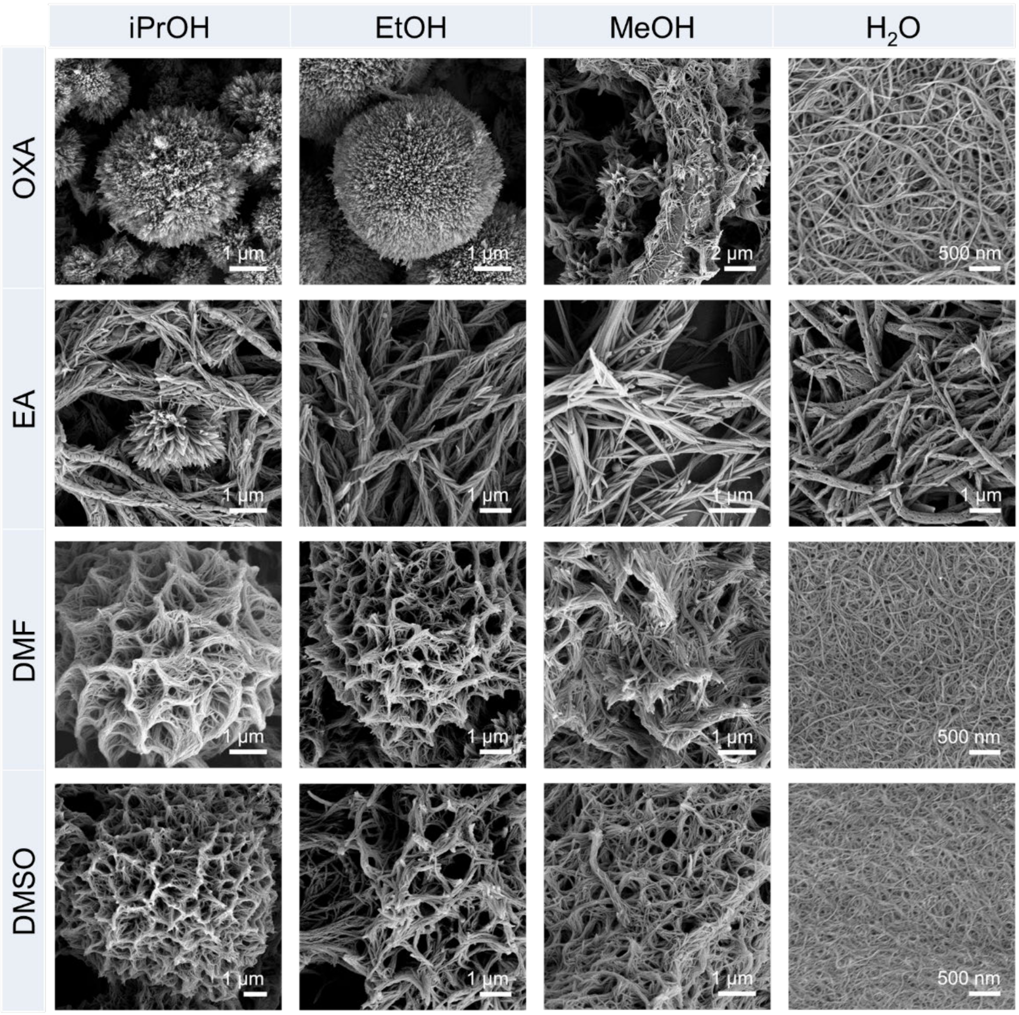

Supplementary Fig. 14 | The morphology of assembly of BACT precipitated in different solvent combinations. All samples were prepared by the above method: TP in different solvents A (20 mM, 100 μL) were mixed with ABA in different solvents B (40 mM, 100 μL). After 1 hr of reaction, the supernatant was removed by centrifugation, and the precipitate was washed by solvent B three times before observation.

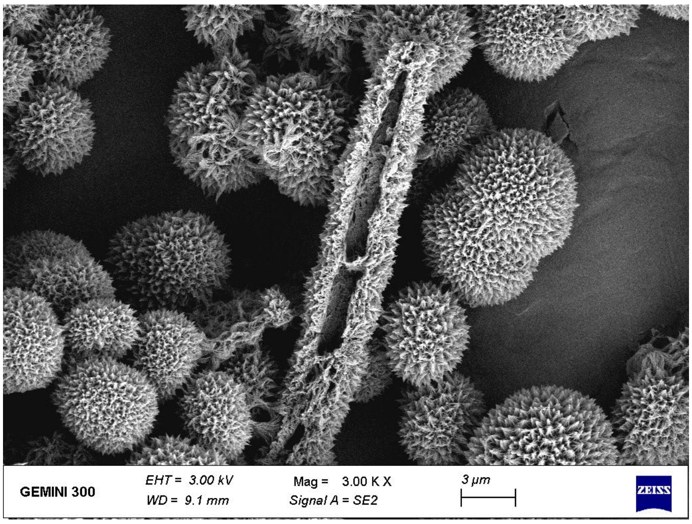

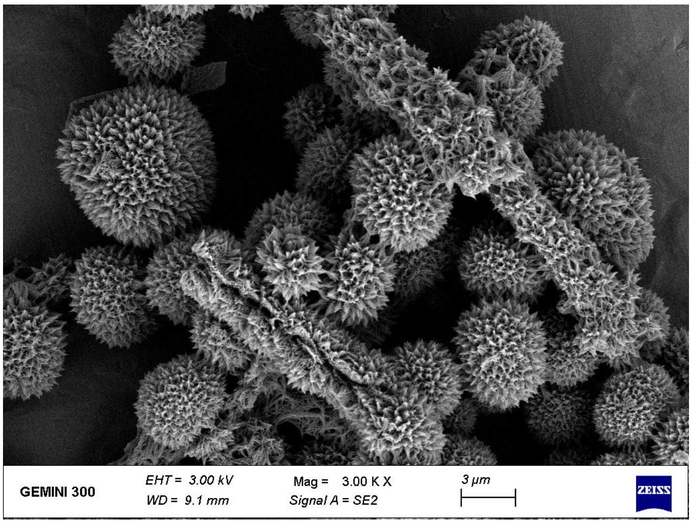

Supplementary Fig. 15 | SEM image of hierarchically assembled hollow tubes. TP in THF (20 mM, 100 μL) was mixed with ABA in MeOH (40 mM, 100 μL). After 1 hr of reaction, the supernatant was removed by centrifugation, and the precipitate was washed with MeOH three times before observation.

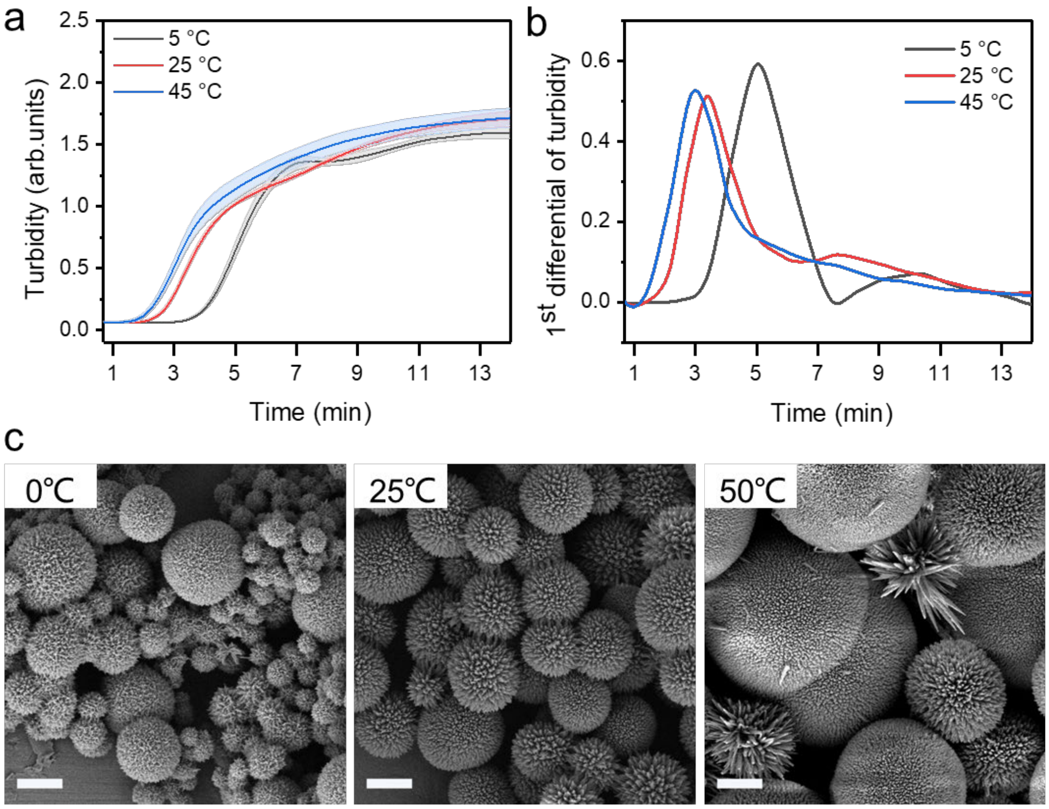

Supplementary Fig. 16 | a Effect of temperature on precipitation kinetics. There was an induction period for each of the three curves, featured by no increase in turbidity in the first few minutes, likely because that this stage is primarily dominated by the imine condensation reaction to yield the doubly condensed product. At 0°C, the induction period lasted for 4 minutes. As the reaction temperature increases, the duration of this process gradually shortens to 2.5 minutes at 25 °C and 2 minutes at 45 °C. In the assembly stage, the turbidity of the suspension increased rapidly. **b** The first order differential of turbidity recorded under different temperature. At 5 °C, the maximum rate of the assembly is higher than those of 25 and 45 °C, likely because that the building blocks are less soluble at lower temperature. **c** SEM image of HOM-1 synthesized under different temperature (the scale bar is 3 μm).

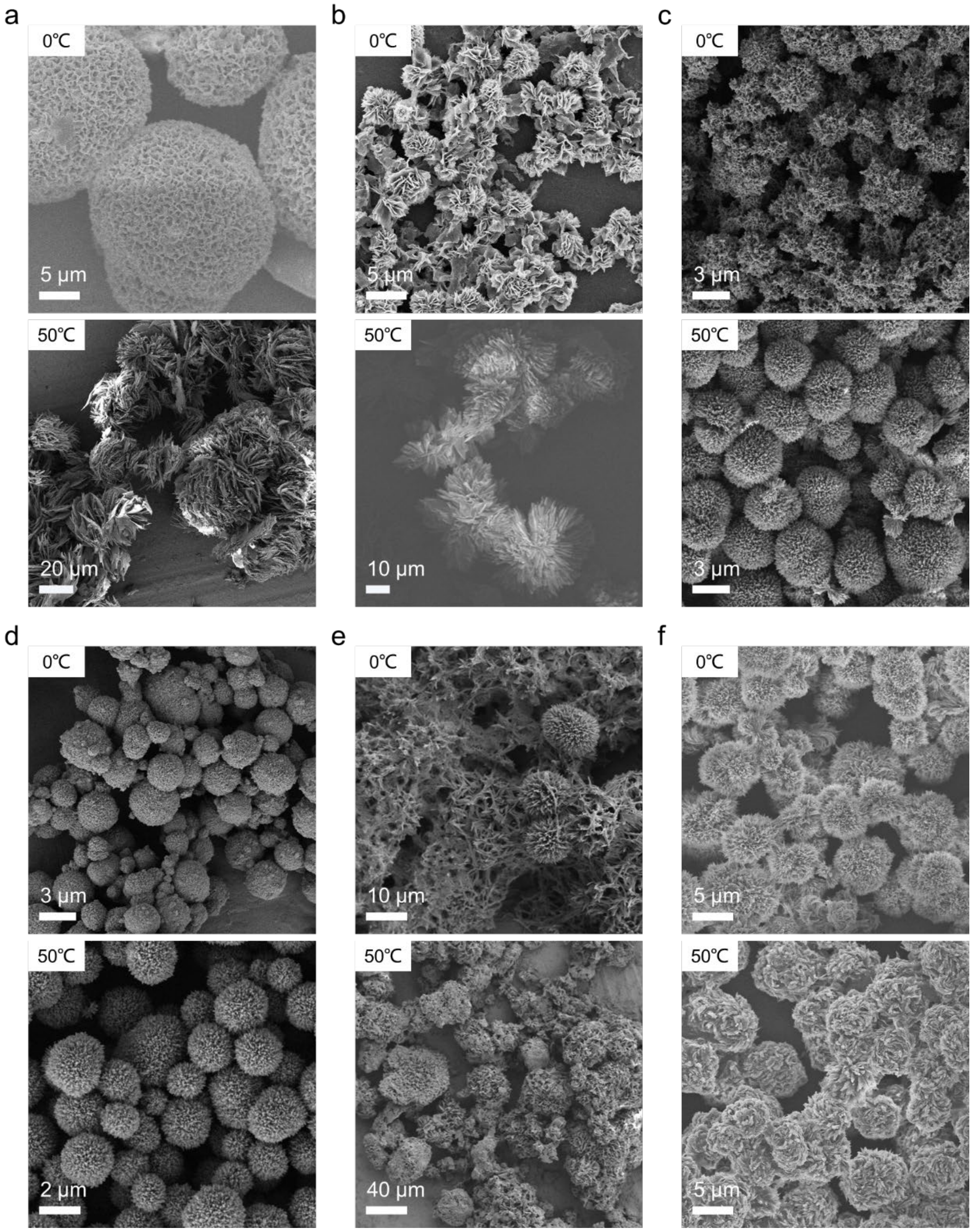
a
0℃
5 μm
50℃
20 μm
b
0℃
5 μm
50℃
10 μm
c
0℃
3 μm
50℃
3 μm
d
0℃
3 μm
50℃
2 μm
e
0℃
10 μm
50℃
40 μm
f
0℃
5 μm
50℃
5 μm

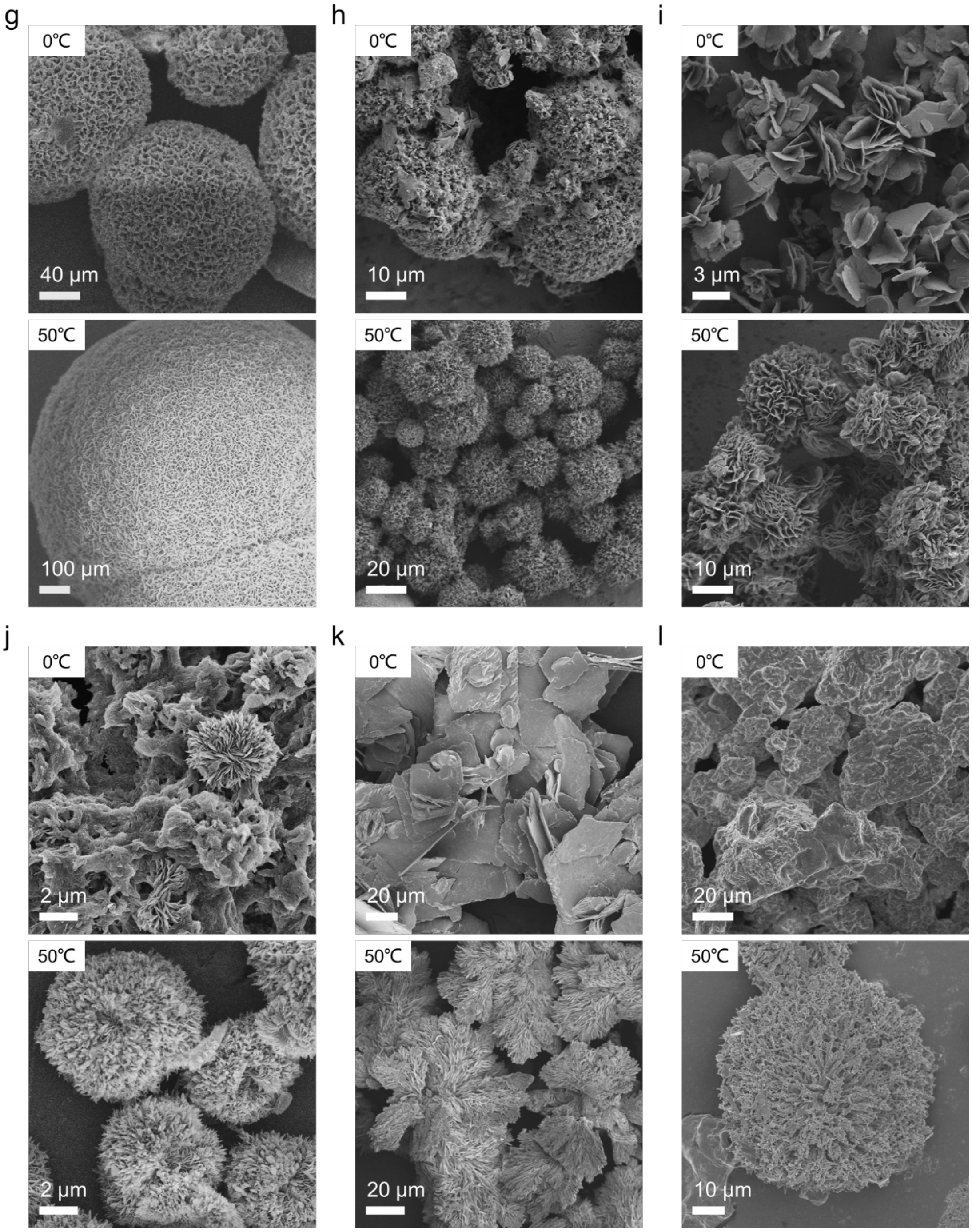
g
0℃
40 μm
50℃
100 μm
h
0℃
10 μm
50℃
20 μm
i
0℃
3 μm
50℃
10 μm
j
0℃
2 μm
50℃
2 μm
k
0℃
20 μm
50℃
20 μm
l
0℃
20 μm
50℃
10 μm

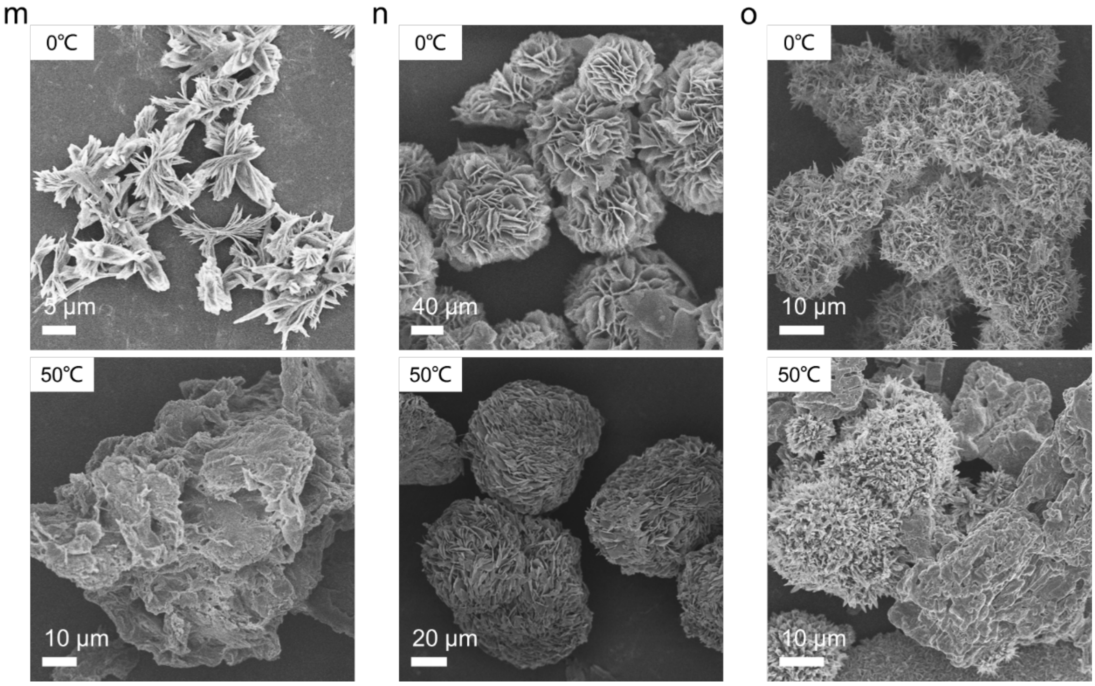

Supplementary Fig. 17 | a-o SEM image of HOM-2~16 synthesized under 0°C and 50 °C.

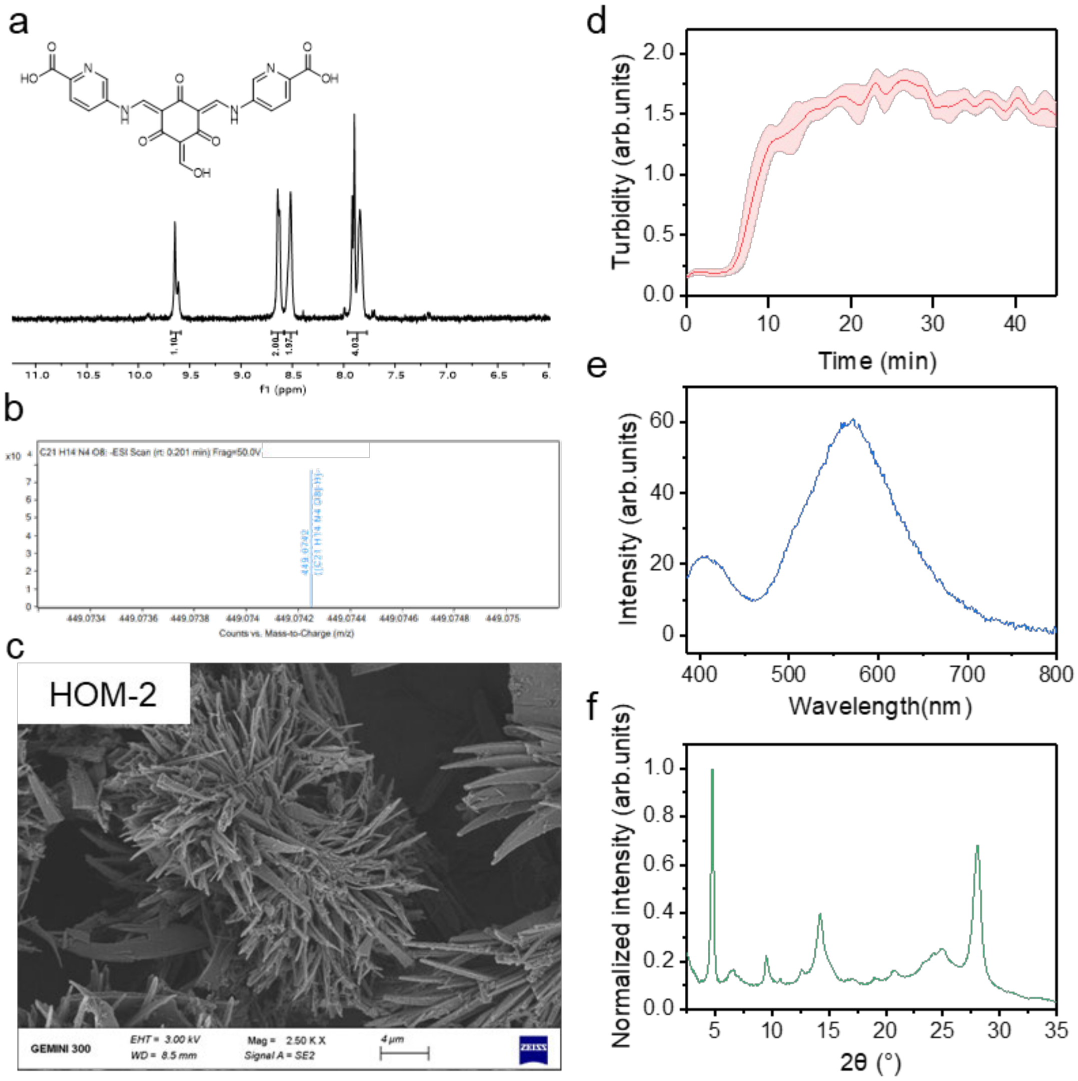

Supplementary Fig. 18 | a ^{1}H NMR of the subunit for HOM-2. **b** HRMS of the subunit for HOM-2. **c** SEM image of HOM-2. **d** The precipitation kinetics of HOM-2. **e,** The emission spectrum of HOM-2 (excited by 365nm). **f** The PXRD pattern for HOM-2.

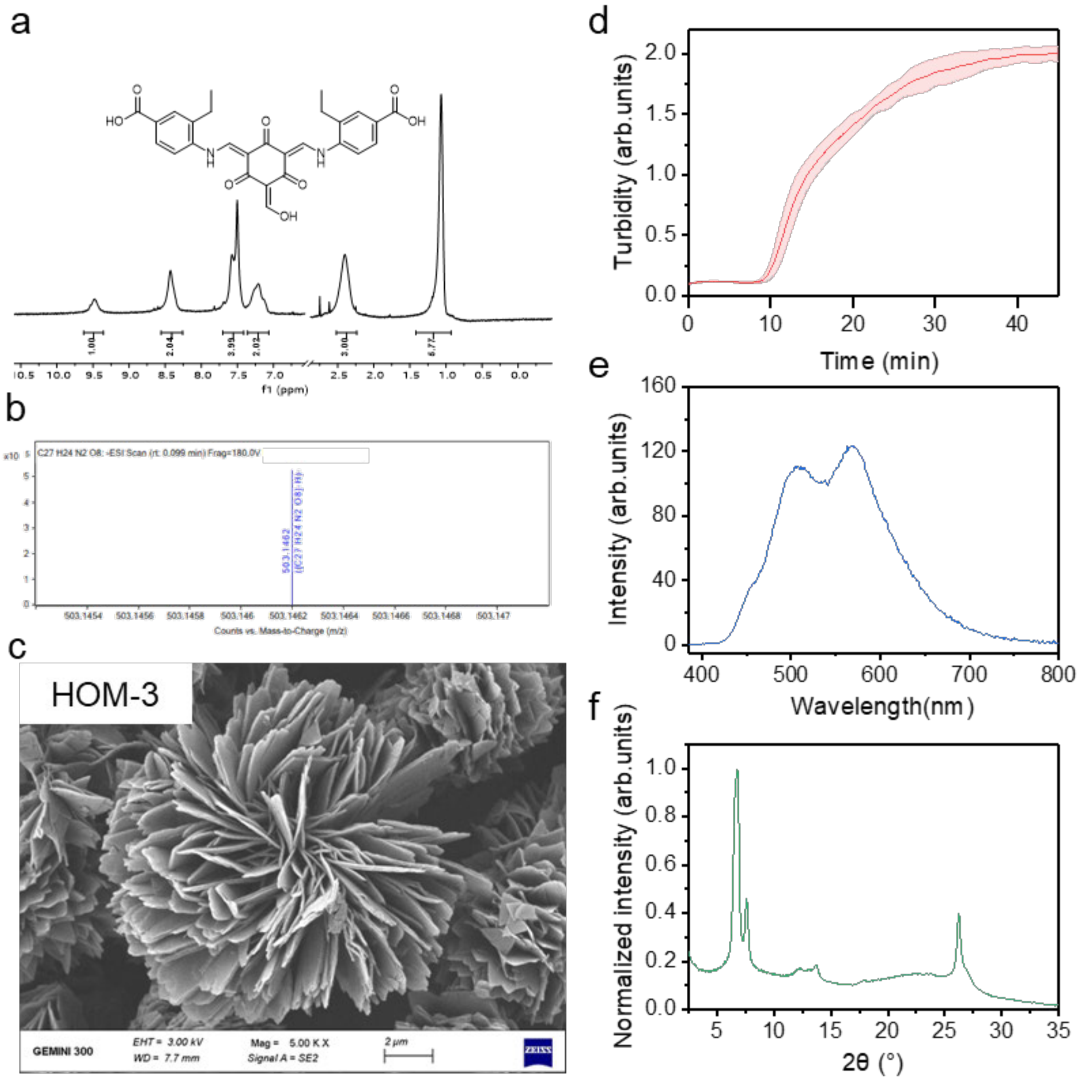

Supplementary Fig. 19 | a ^{1}H NMR of the subunit for HOM-3. **b** HRMS of the subunit for HOM-3. **c** SEM image of HOM-3. **d** The precipitation kinetics of HOM-3. **e** The emission spectrum of HOM-3 (excited by 365nm). **f** The PXRD pattern for HOM-3.

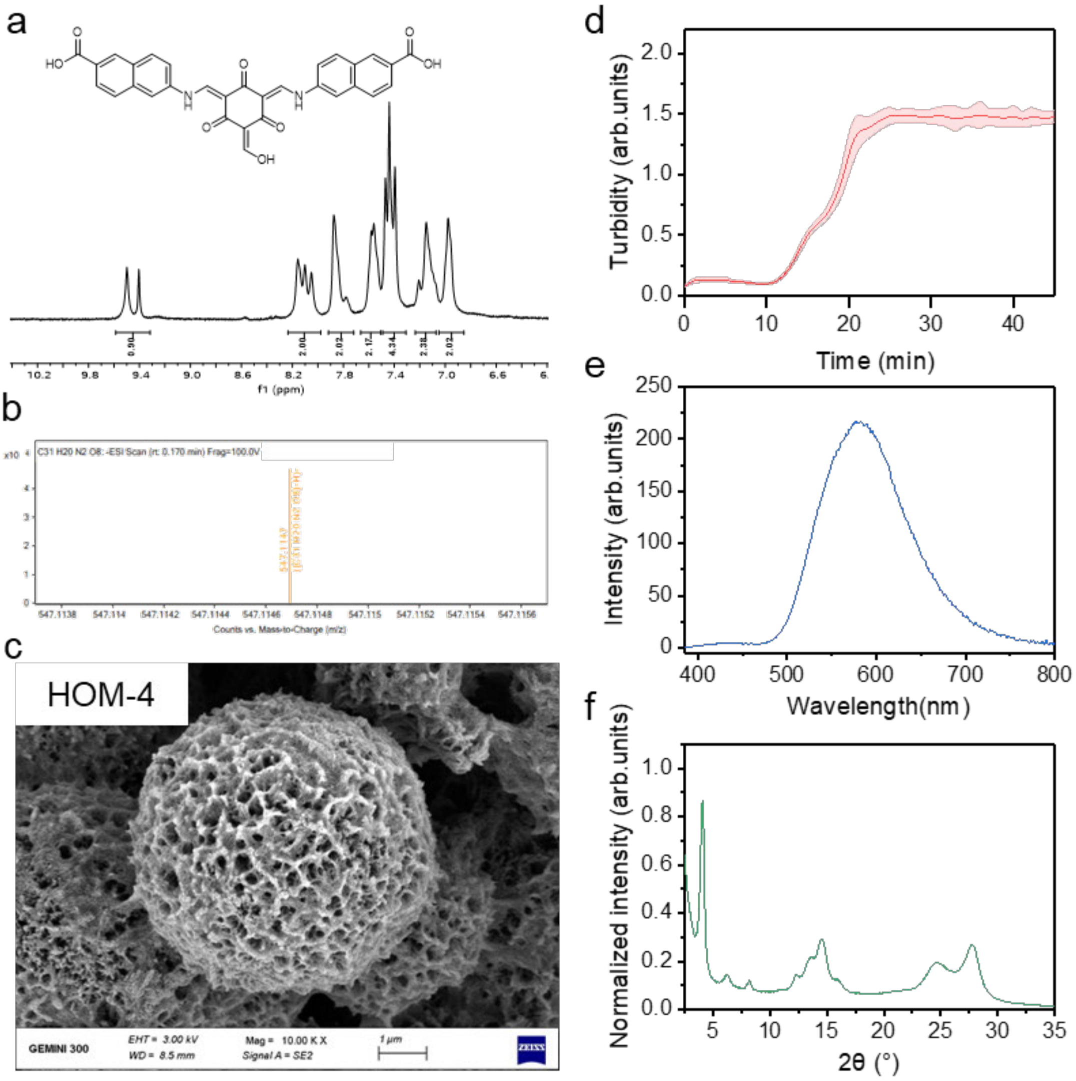

Supplementary Fig. 20 | a ^{1}H NMR of the subunit for HOM-4. **b** HRMS of the subunit for HOM-4. **c** SEM image of HOM-4. **d** The precipitation kinetics of HOM-4. **e** The emission spectrum of HOM-4 (excited by 365nm). **f** The PXRD pattern for HOM-4.

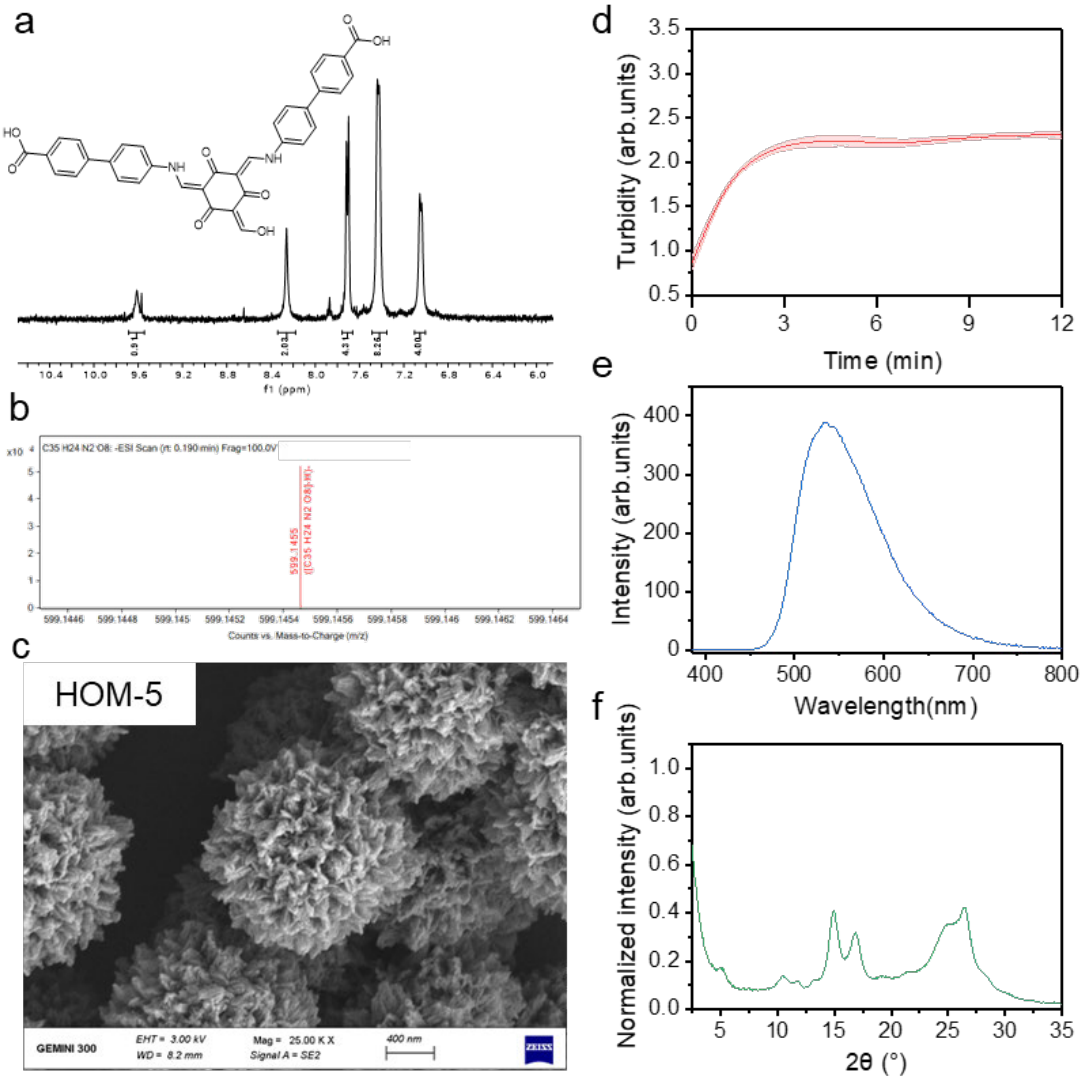

Supplementary Fig. 21 | a ^{1}H NMR of the subunit for HOM-5. **b** HRMS of the subunit for HOM-5. **c** SEM image of HOM-5. **d** The precipitation kinetics of HOM-5. **e** The emission spectrum of HOM-5 (excited by 365nm). **f** The PXRD pattern for HOM-5.

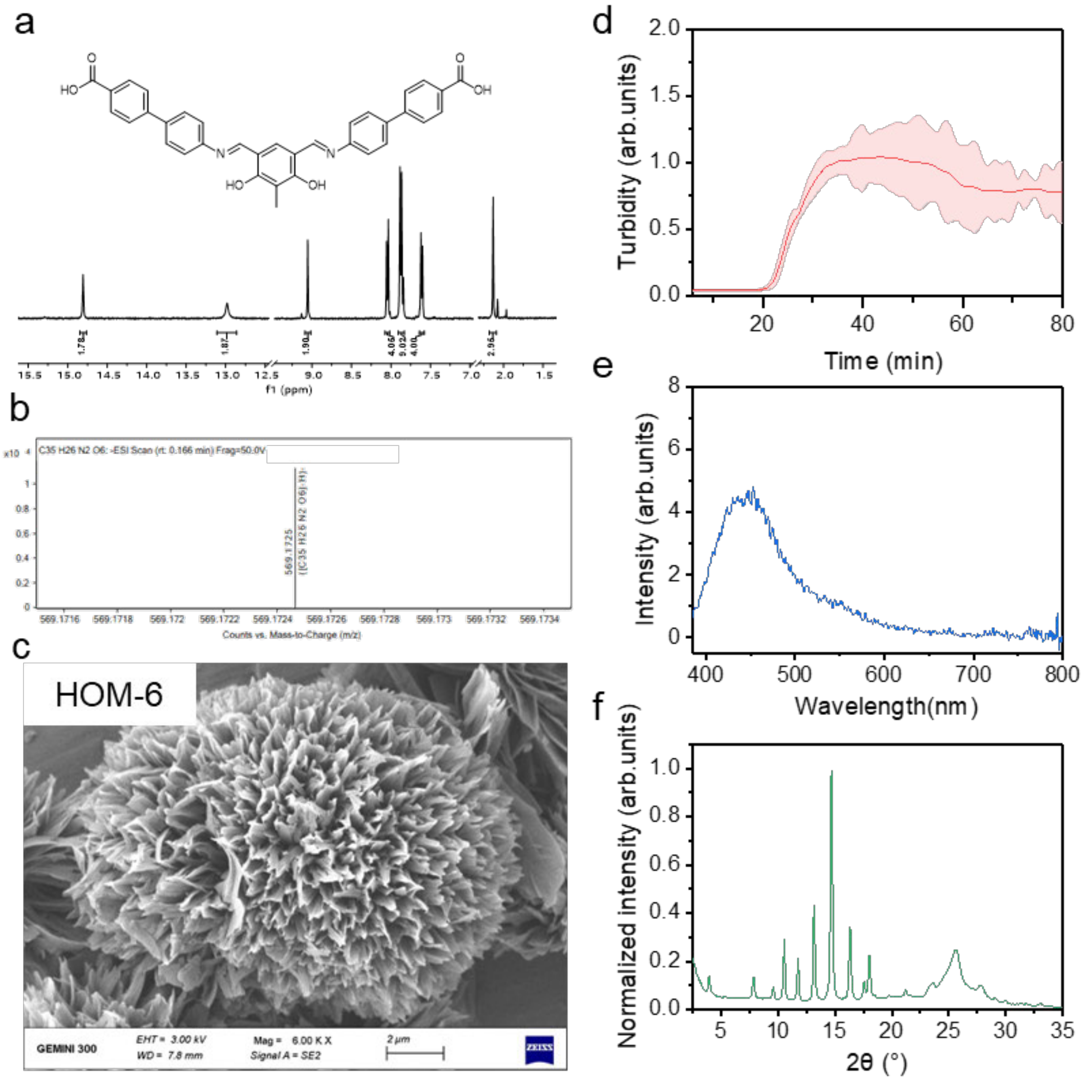

Supplementary Fig. 22 | a ^{1}H NMR of the subunit for HOM-6. **b** HRMS of the subunit for HOM-6. **c** SEM image of HOM-6. **d** The precipitation kinetics of HOM-6. **e** The emission spectrum of HOM-6 (excited by 365nm). **f** The PXRD pattern for HOM-6.

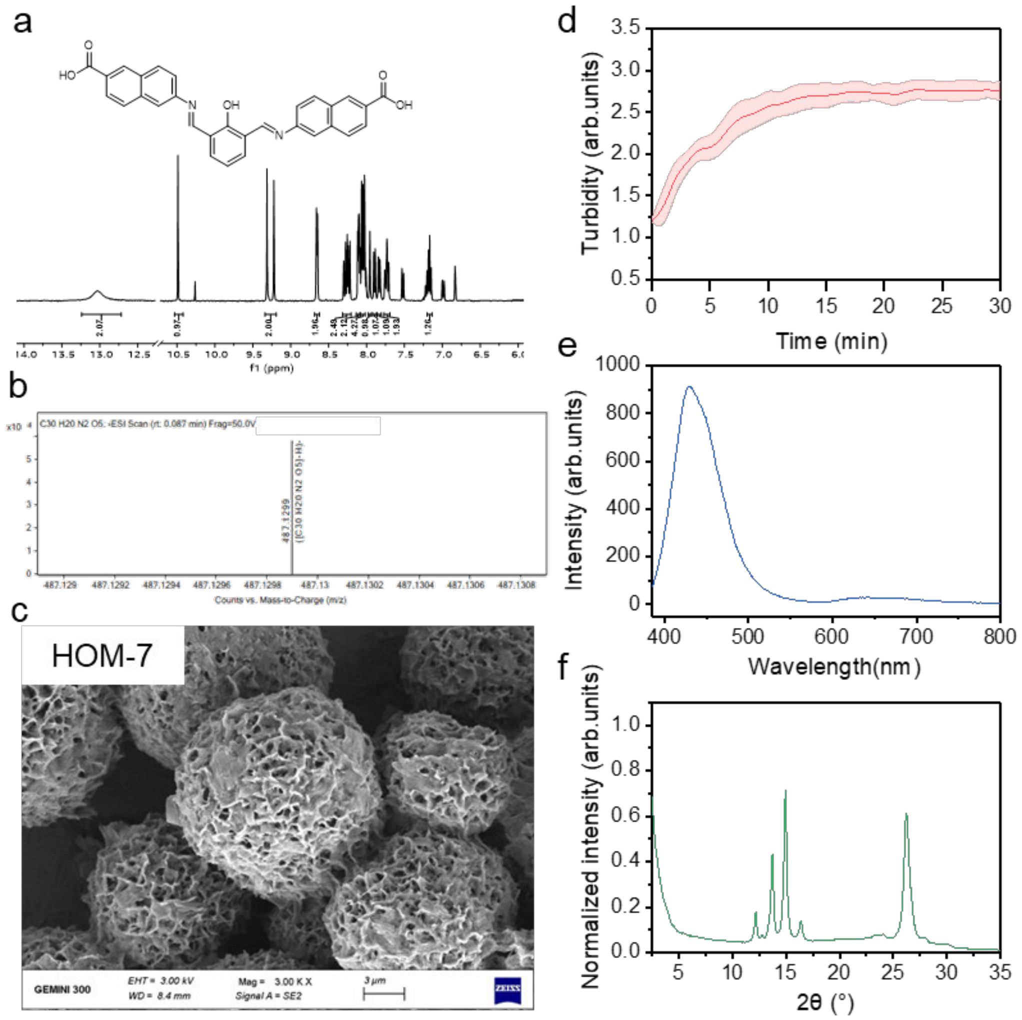

Supplementary Fig. 23 | a ^{1}H NMR of the subunit for HOM-7. **b** HRMS of the subunit for HOM-7. **c** SEM image of HOM-7. **d** The precipitation kinetics of HOM-7. **e** The emission spectrum of HOM-7 (excited by 365nm). **f** The PXRD pattern for HOM-7.

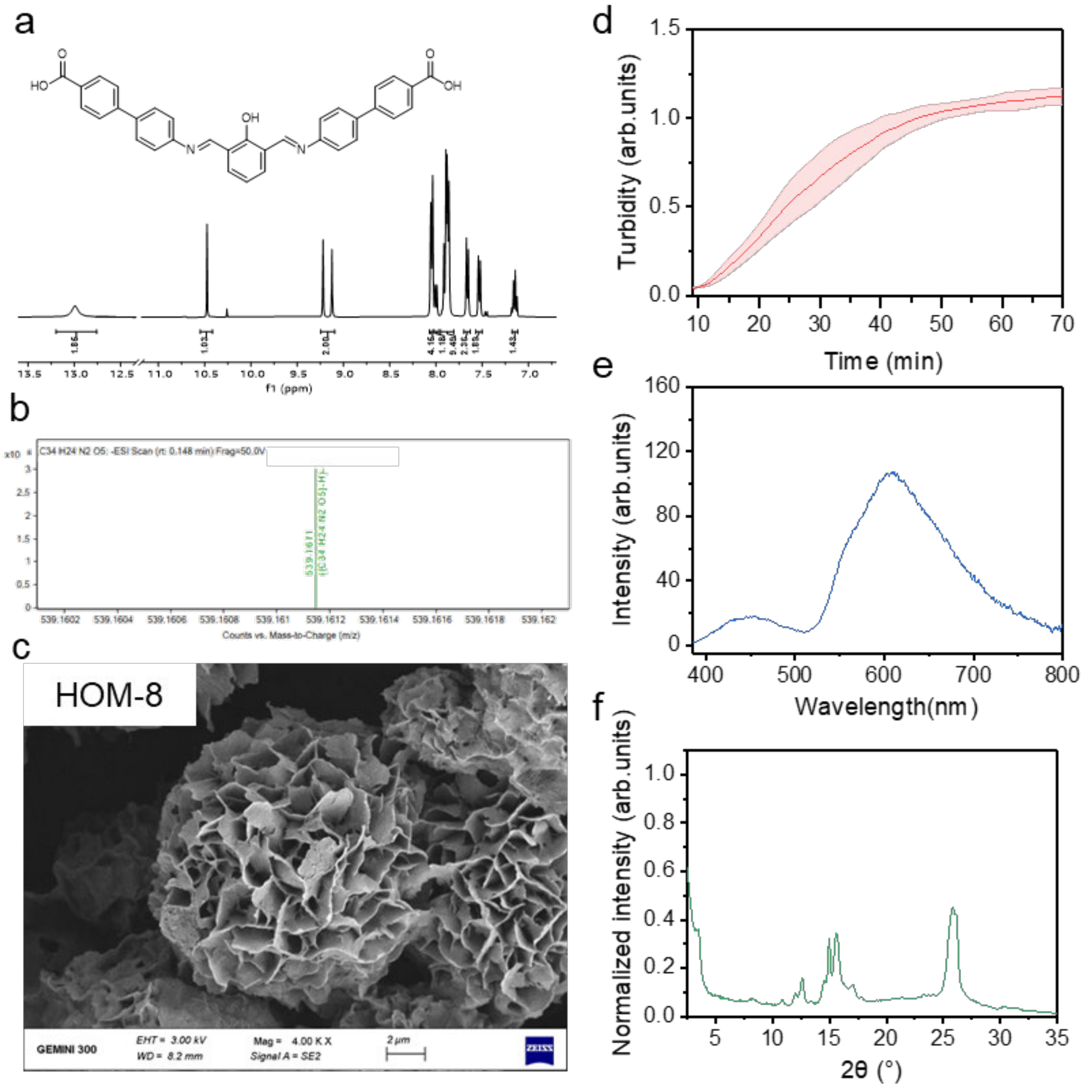

Supplementary Fig. 24 | a ^{1}H NMR of the subunit for HOM-8. **b** HRMS of the subunit for HOM-8. **c** SEM image of HOM-8. **d** The precipitation kinetics of HOM-8. **e** The emission spectrum of HOM-8 (excited by 365nm). **f** The PXRD pattern for HOM-8.

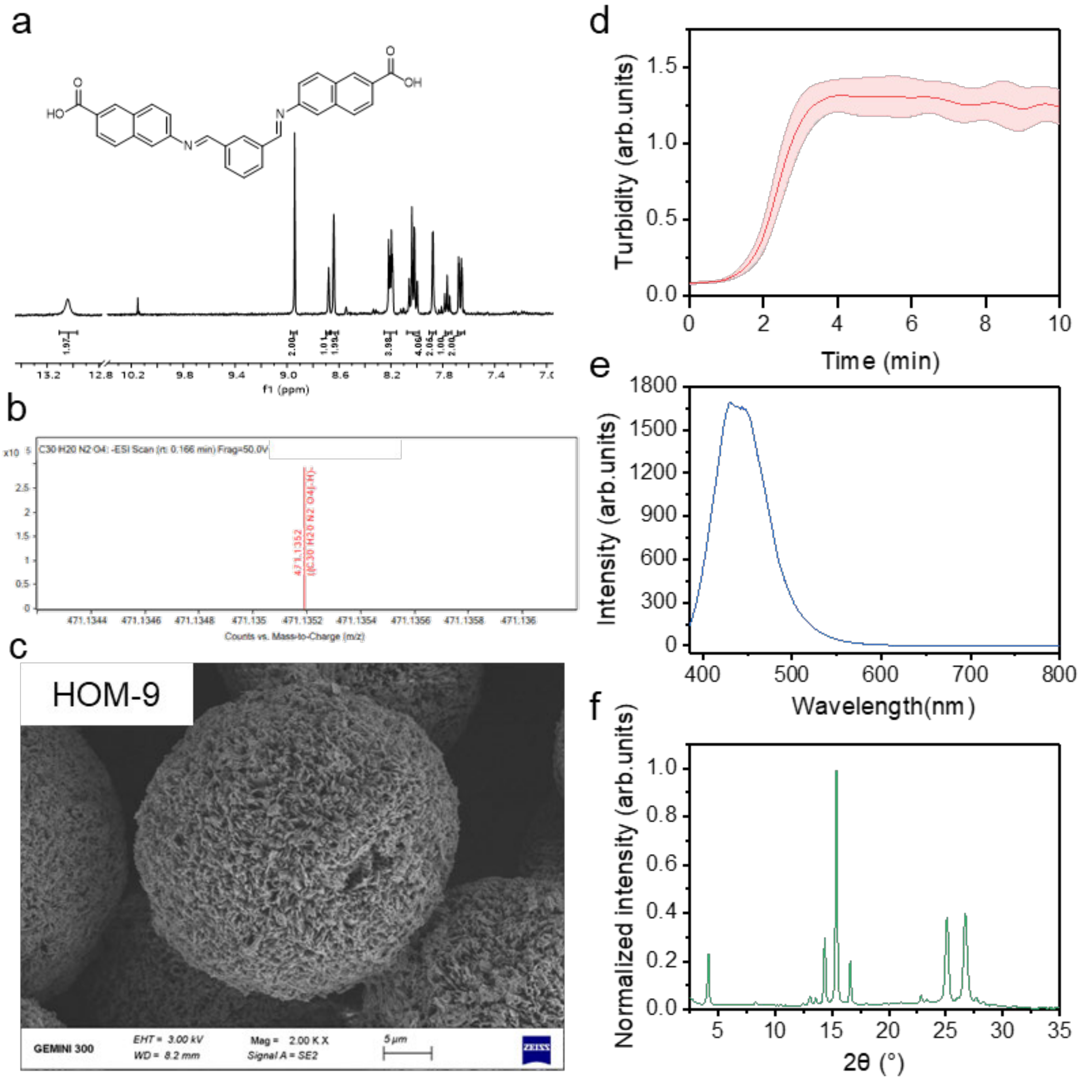

Supplementary Fig. 25 | a ^{1}H NMR of the subunit for HOM-9. **b** HRMS of the subunit for HOM-9. **c** SEM image of HOM-9. **d** The precipitation kinetics of HOM-9. **e** The emission spectrum of HOM-9 (excited by 365nm). **f** The PXRD pattern for HOM-9.

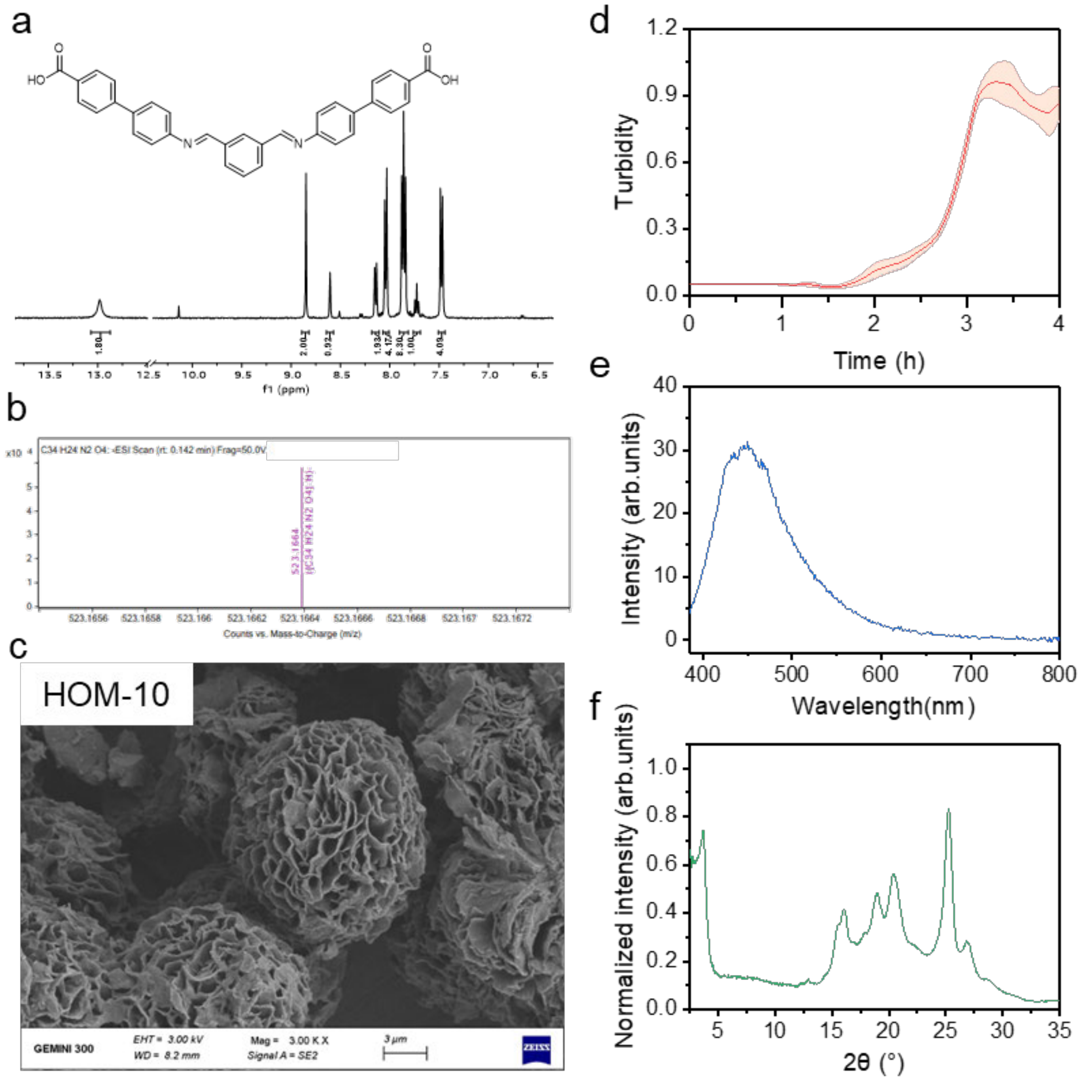

Supplementary Fig. 26 | a ^{1}H NMR of the subunit for HOM-10. **b** HRMS of the subunit for HOM-10. **c** SEM image of HOM-10. **d** The precipitation kinetics of HOM-10. **e** The emission spectrum of HOM-10 (excited by 365nm). **f** The PXRD pattern for HOM-10.

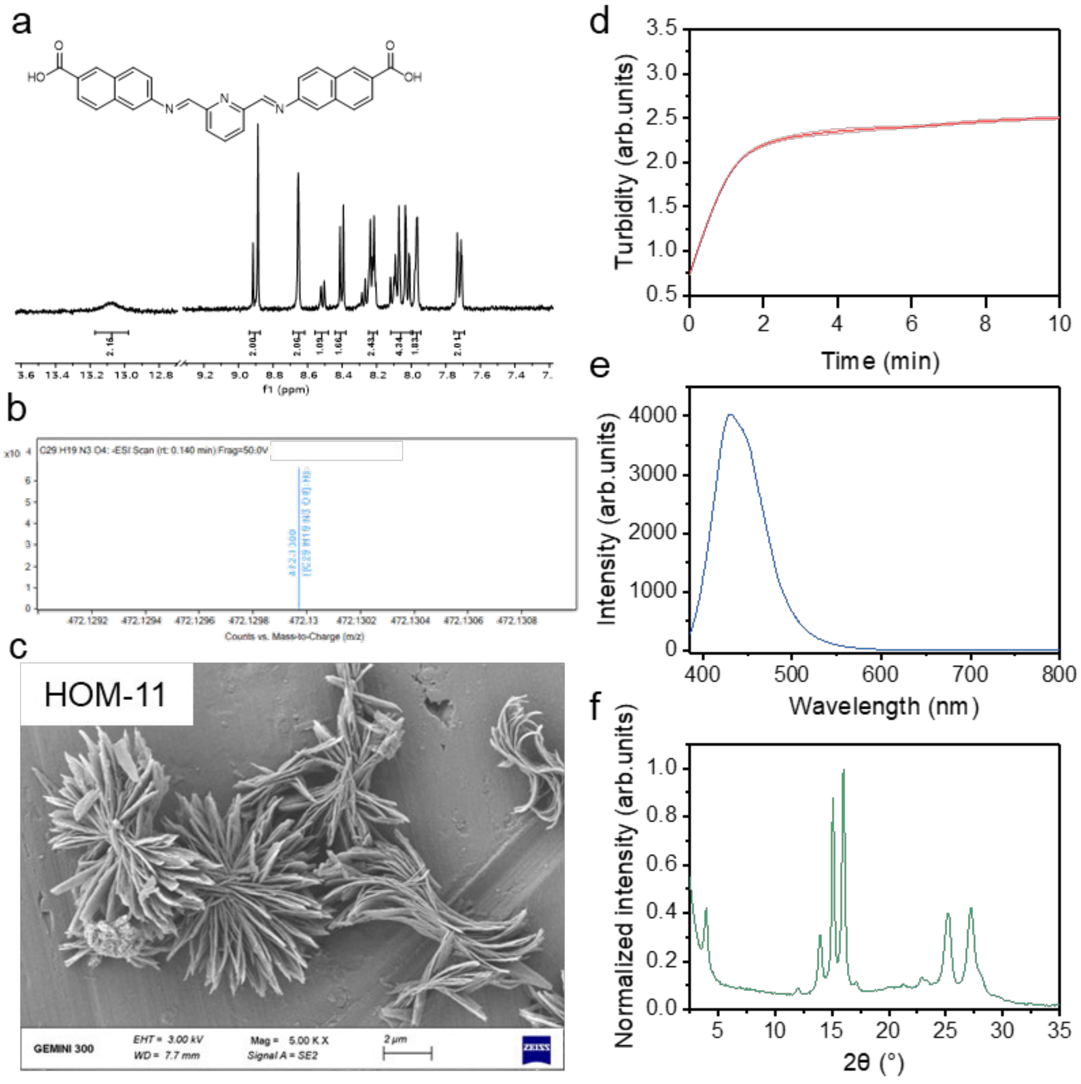

Supplementary Fig. 27 | a ^{1}H NMR of the subunit for HOM-11. **b** HRMS of the subunit for HOM-11. **c** SEM image of HOM-11. **d** The precipitation kinetics of HOM-11. **e** The emission spectrum of HOM-11 (excited by 365nm). **f** The PXRD pattern for HOM-11.

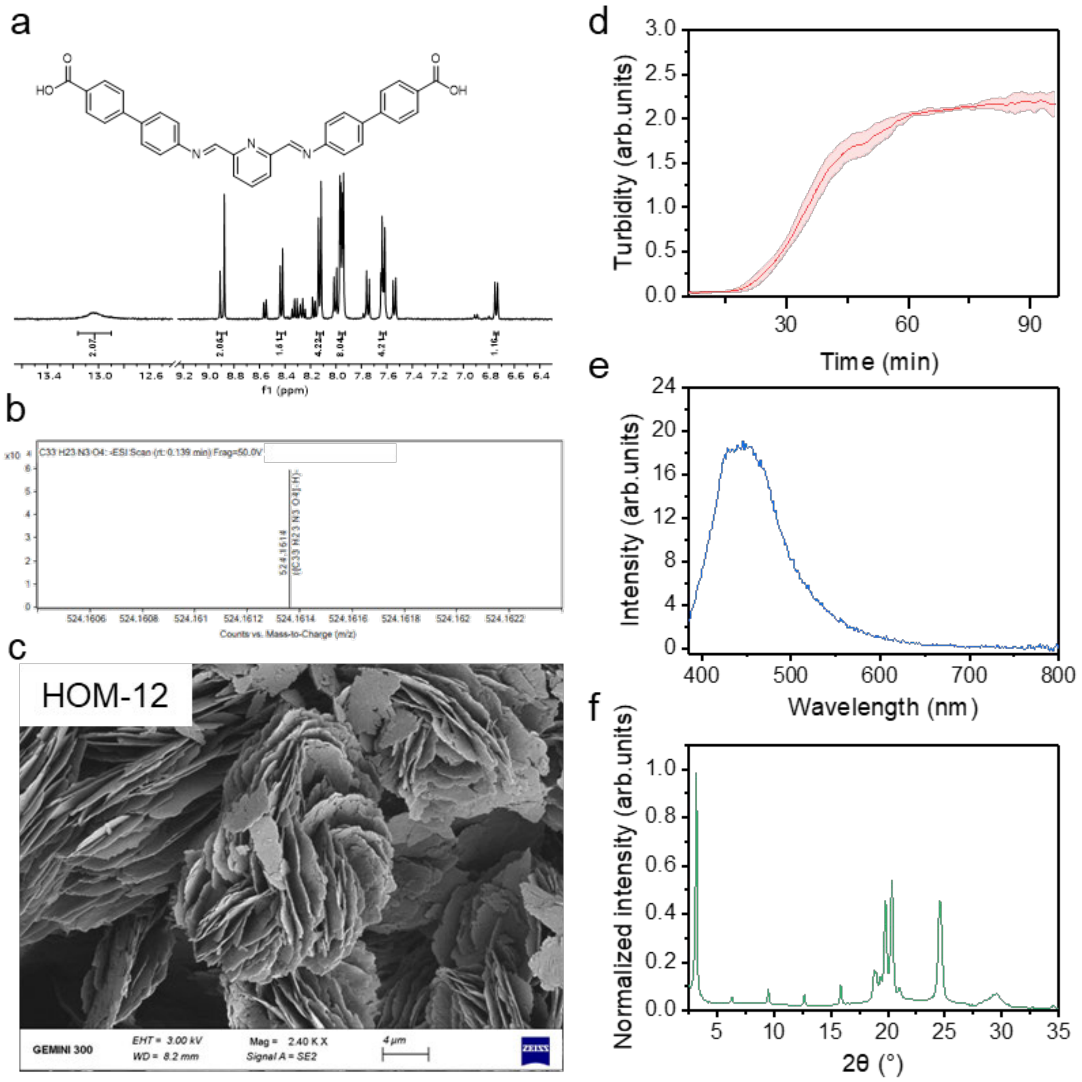

Supplementary Fig. 28 | a ^{1}H NMR of the subunit for HOM-12. **b** HRMS of the subunit for HOM-12. **c** SEM image of HOM-12. **d** The precipitation kinetics of HOM-12. **e** The emission spectrum of HOM-12 (excited by 365nm). **f** The PXRD pattern for HOM-12.

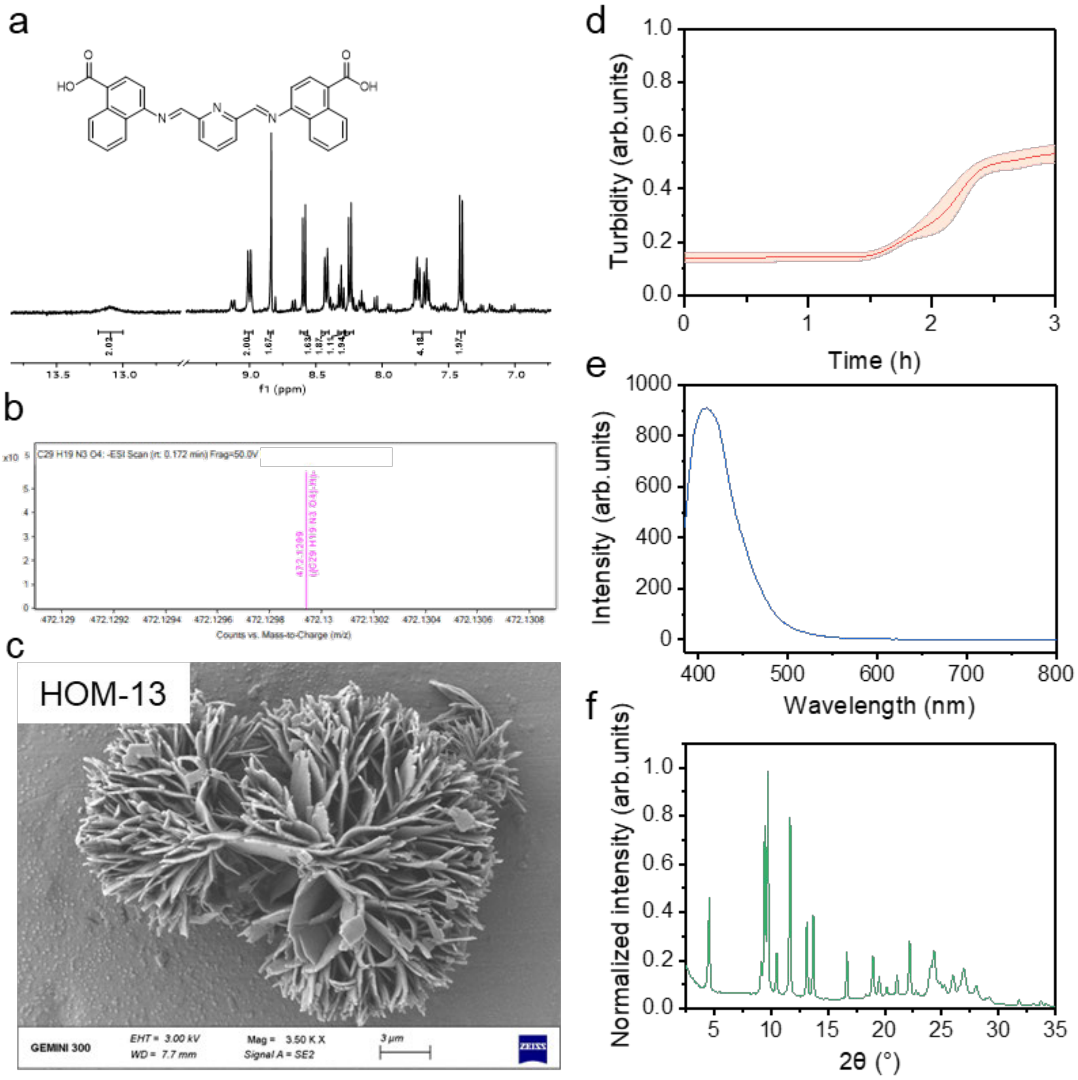

Supplementary Fig. 29 | a ^{1}H NMR of the subunit for HOM-13. **b** HRMS of the subunit for HOM-13. **c** SEM image of HOM-13. **d** The precipitation kinetics of HOM-13. **e** The emission spectrum of HOM-13 (excited by 365nm). **f** The PXRD pattern for HOM-13.

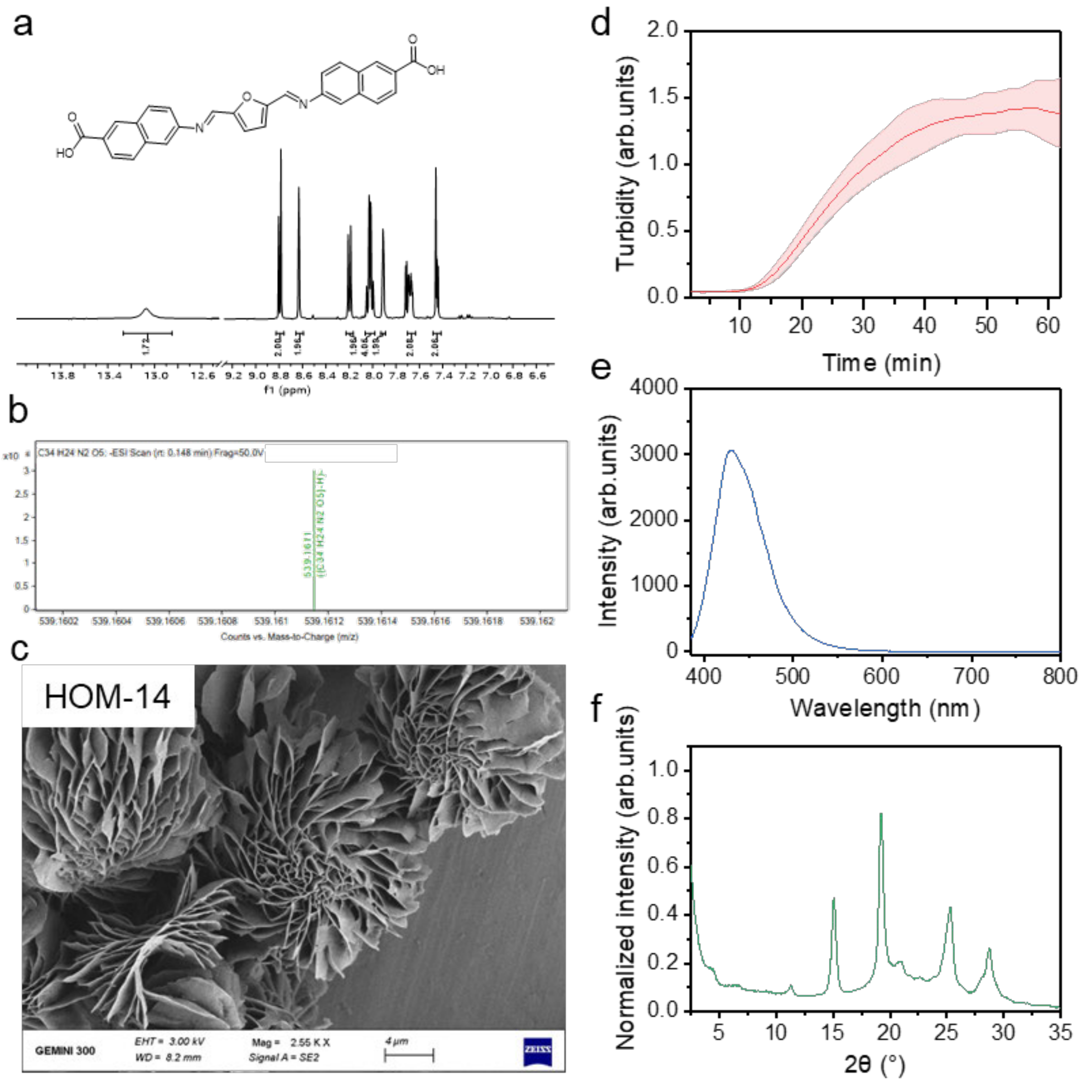

Supplementary Fig. 30 | a ^{1}H NMR of the subunit for HOM-14. **b** HRMS of the subunit for HOM-14. **c** SEM image of HOM-14. **d** The precipitation kinetics of HOM-14. **e** The emission spectrum of HOM-14 (excited by 365nm). **f** The PXRD pattern for HOM-14.

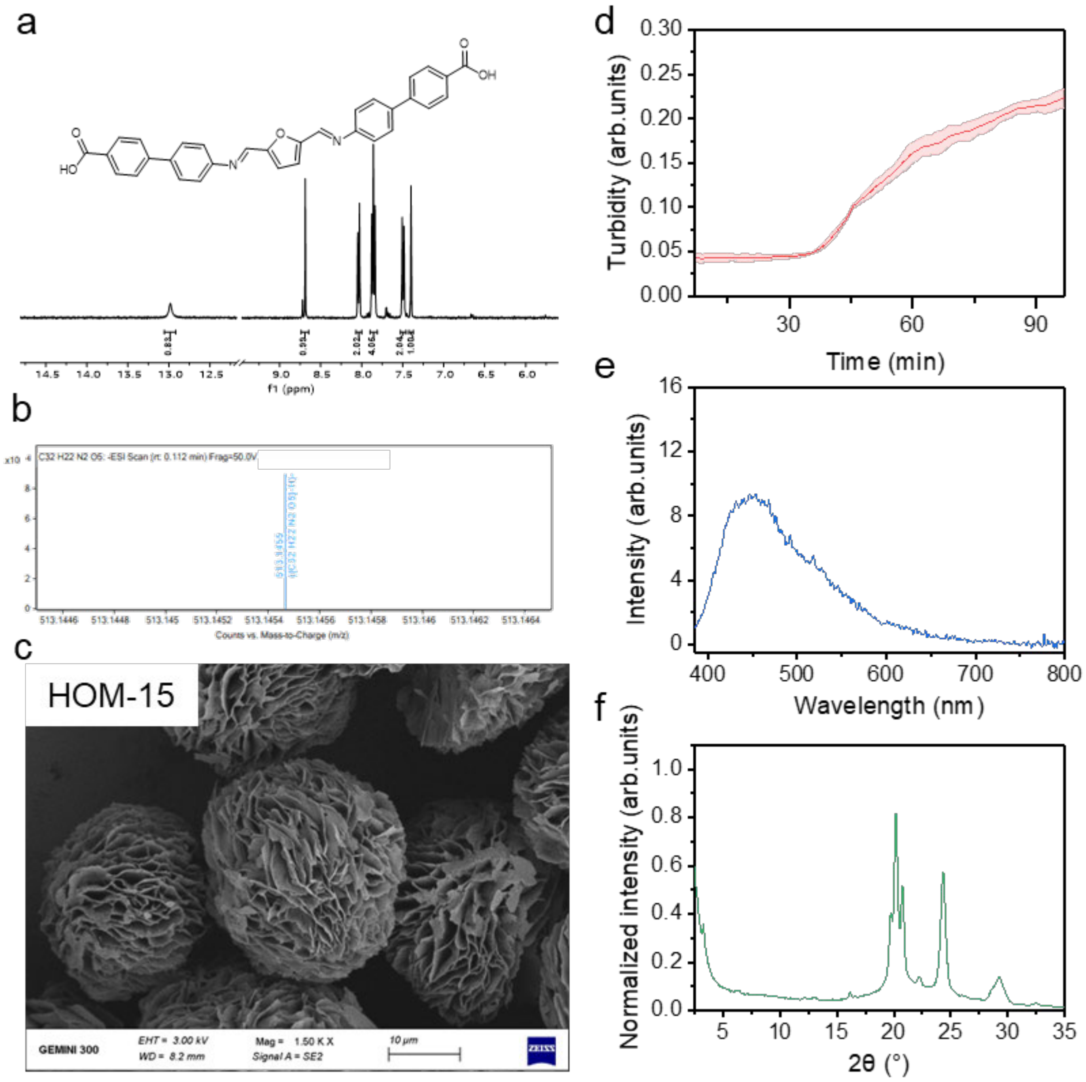

Supplementary Fig. 31 | a ^{1}H NMR of the subunit for HOM-15. **b** HRMS of the subunit for HOM-15. **c** SEM image of HOM-15. **d** The precipitation kinetics of HOM-15. **e** The emission spectrum of HOM-15 (excited by 365nm). **f** The PXRD pattern for HOM-15.

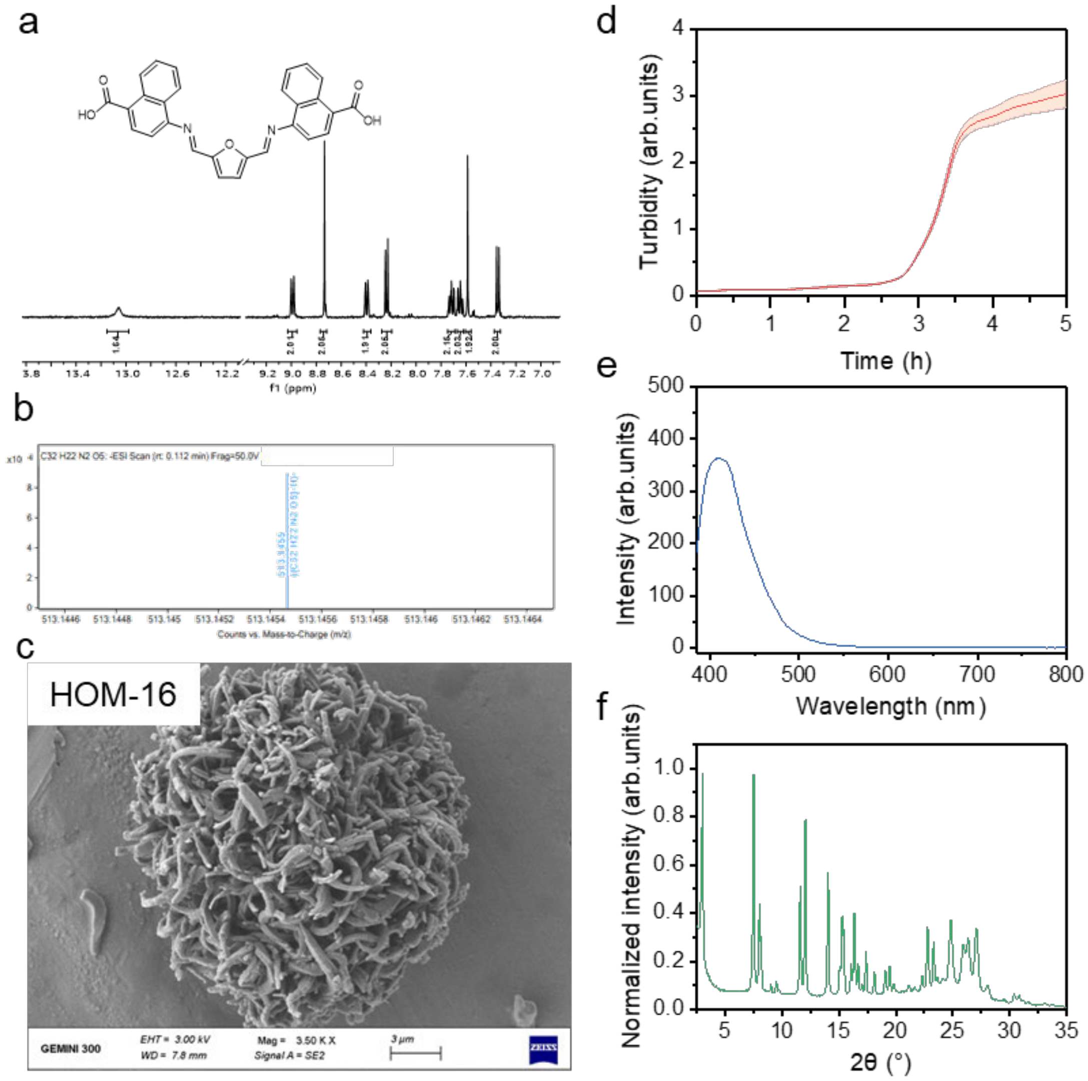

Supplementary Fig. 32 | a ^{1}H NMR of the subunit for HOM-16. **b** HRMS of the subunit for HOM-16. **c** SEM image of HOM-16. **d** The precipitation kinetics of HOM-16. **e** The emission spectrum of HOM-16 (excited by 365nm). **f** The PXRD pattern for HOM-16.

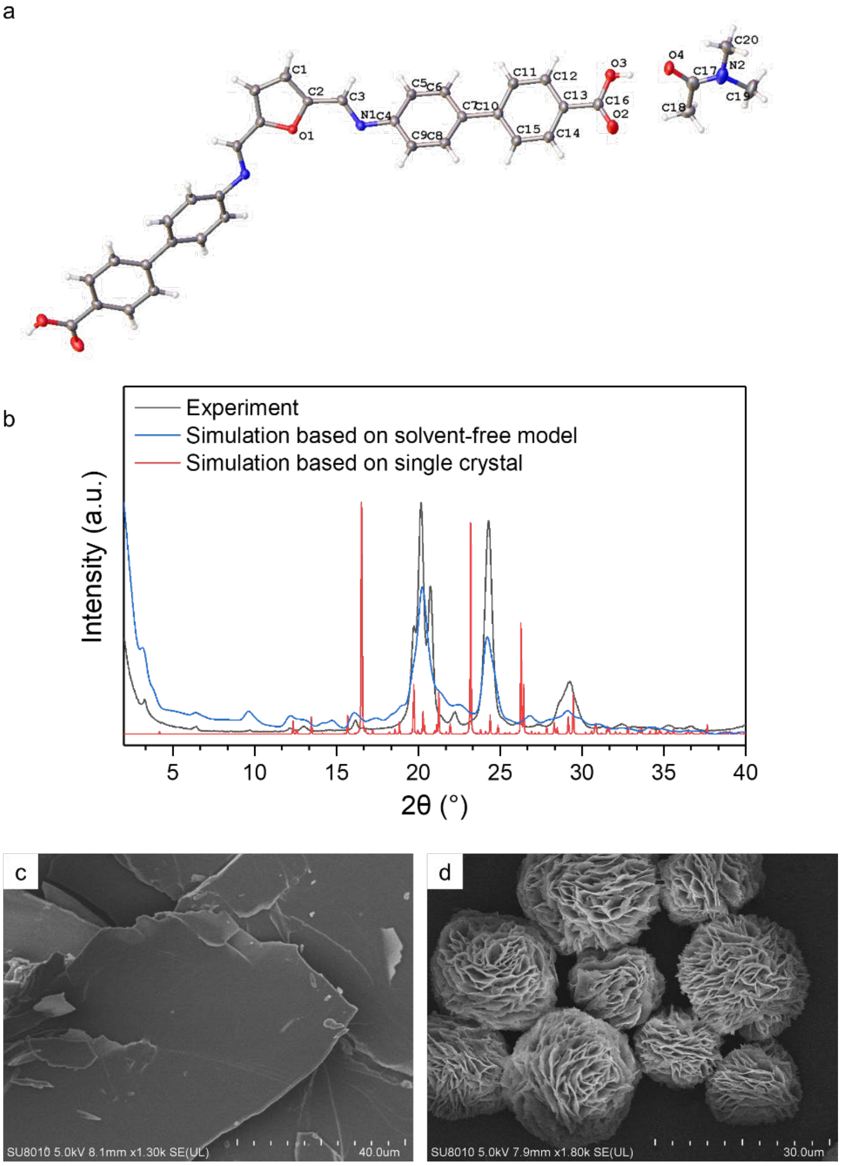

Supplementary Fig. 33 | a Single crystal structure of 4',4''-[furan-2,5-diylbis(methanylylideneazanylylidene)]bis([1,1'-biphenyl]-4-carboxylic acid) N,N-dimethylacetamide solvate displayed in ORTEP diagram. **b** Simulation PXRD pattern of the above single crystal structure, compared with the experimental PXRD pattern measured with HOM-15. 4',4''-[furan-2,5-diylbis(methanylylideneazanylylidene)]-bis([1,1'-biphenyl]-4-carboxylic acid) is the subunit for HOM-15. **c** SEM image of the plate-like single crystal which was synthesized in DMAc and iPrOH. **d** SEM image of the HOM-15.

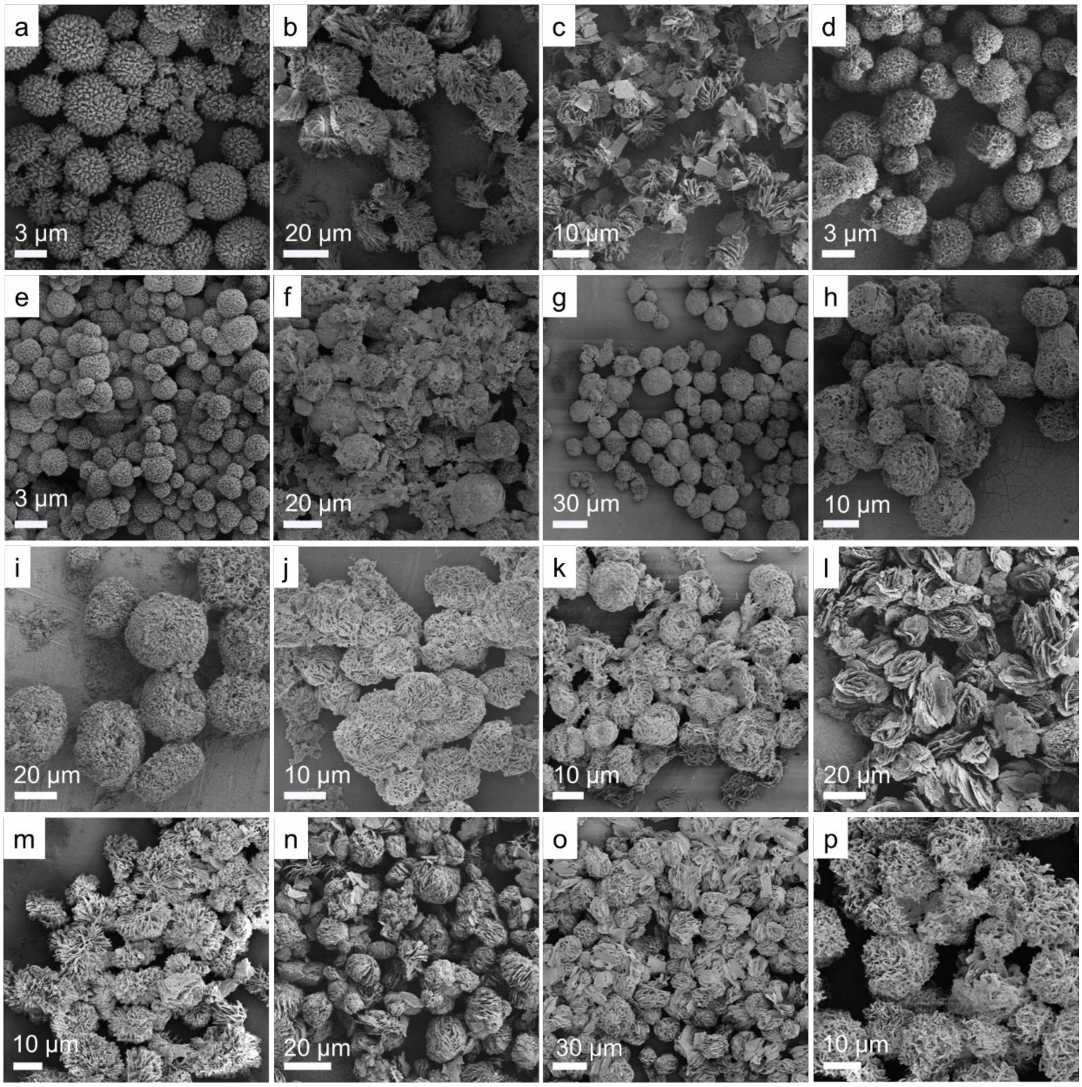

Supplementary Fig. 34 | a-p SEM image of HOM-1~16 which were stored in powder form at ambient condition for 1.

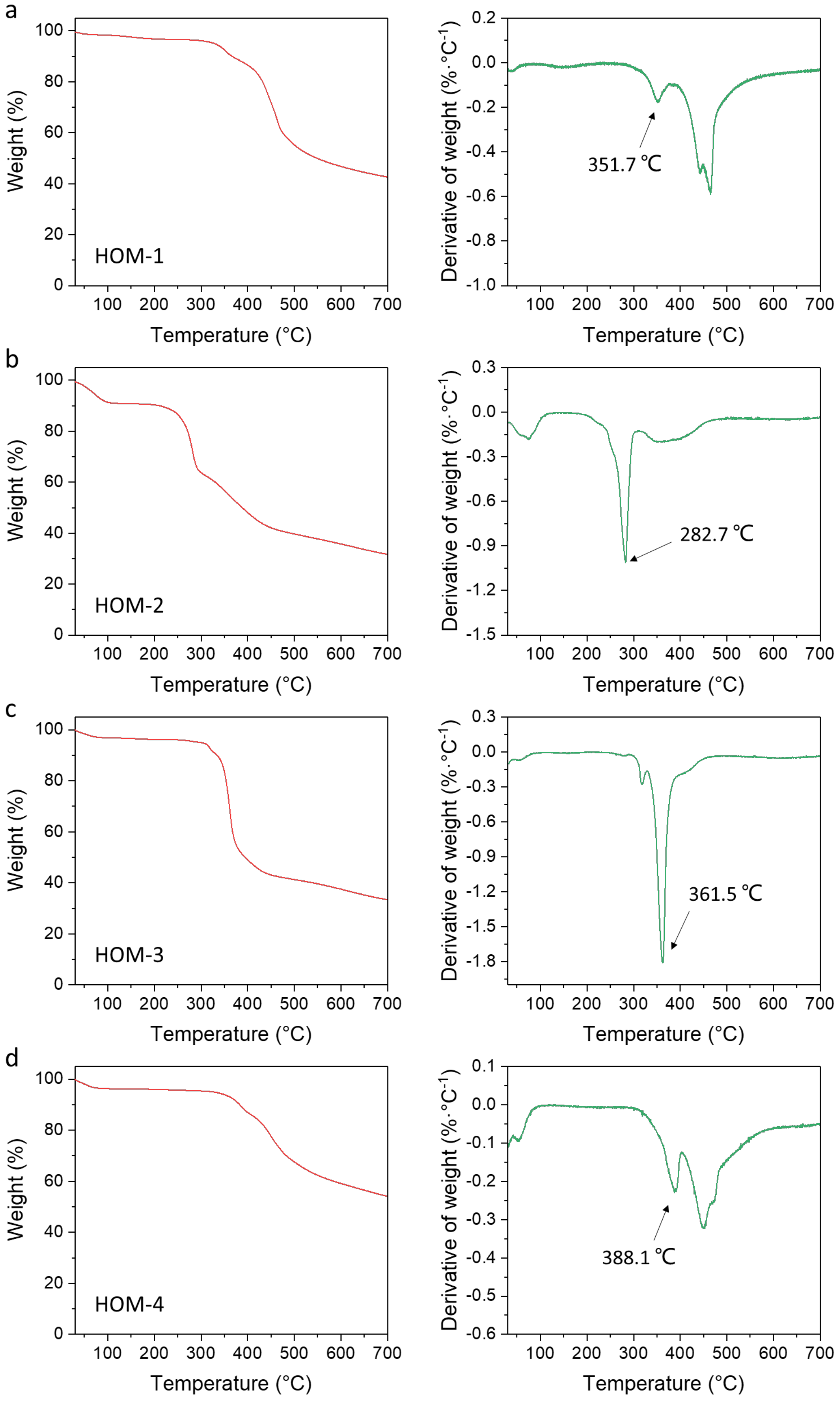
a
b
c
d
HOM-1
HOM-2
HOM-3
HOM-4
Weight (%)
Derivative of weight (%·°C⁻¹)
Temperature (°C)
351.7 ℃
282.7 ℃
361.5 ℃
388.1 ℃

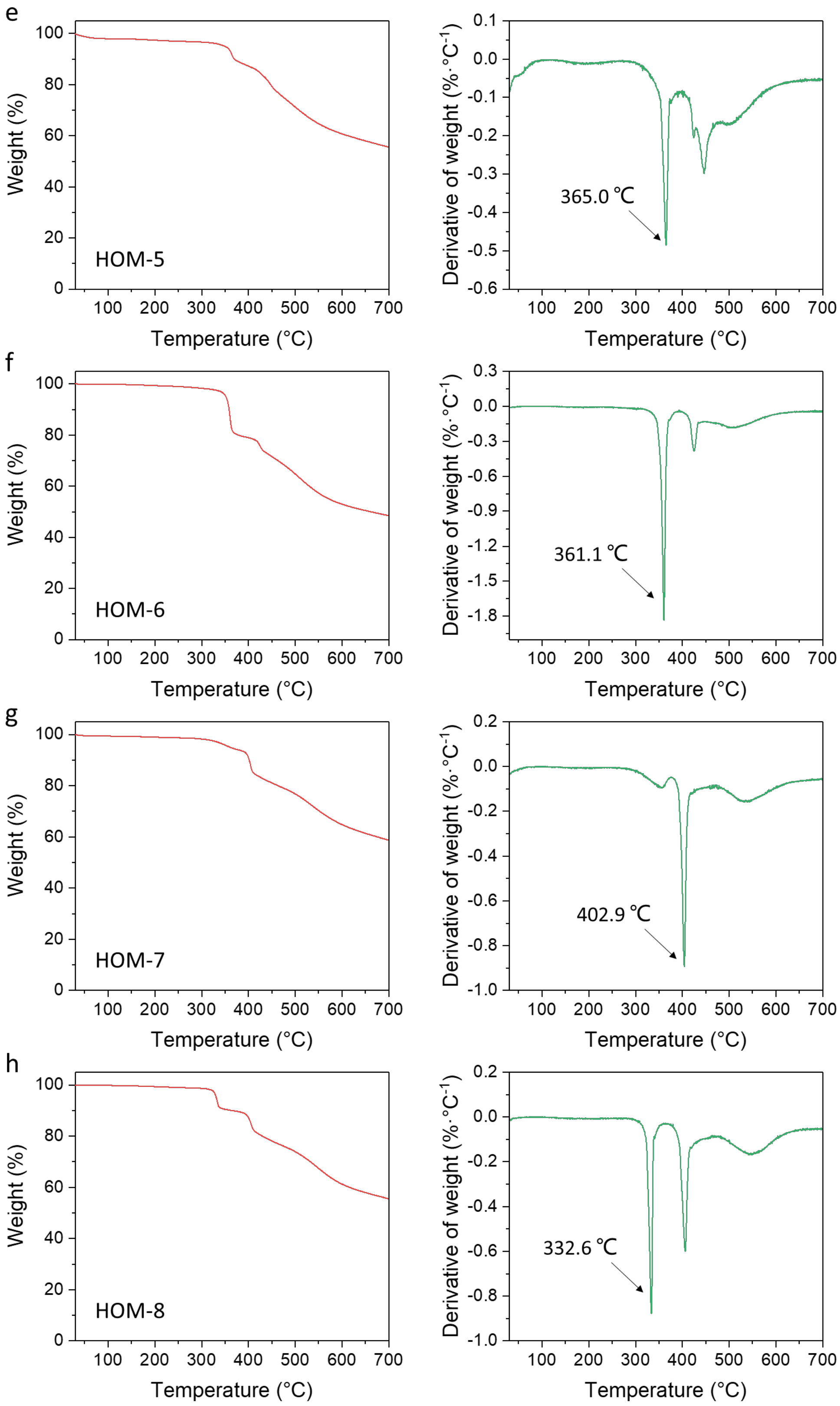
e
Weight (%)
Temperature (°C)
HOM-5
Derivative of weight (%·°C^{-1})
365.0 ℃
f
HOM-6
361.1 ℃
g
HOM-7
402.9 ℃
h
HOM-8
332.6 ℃

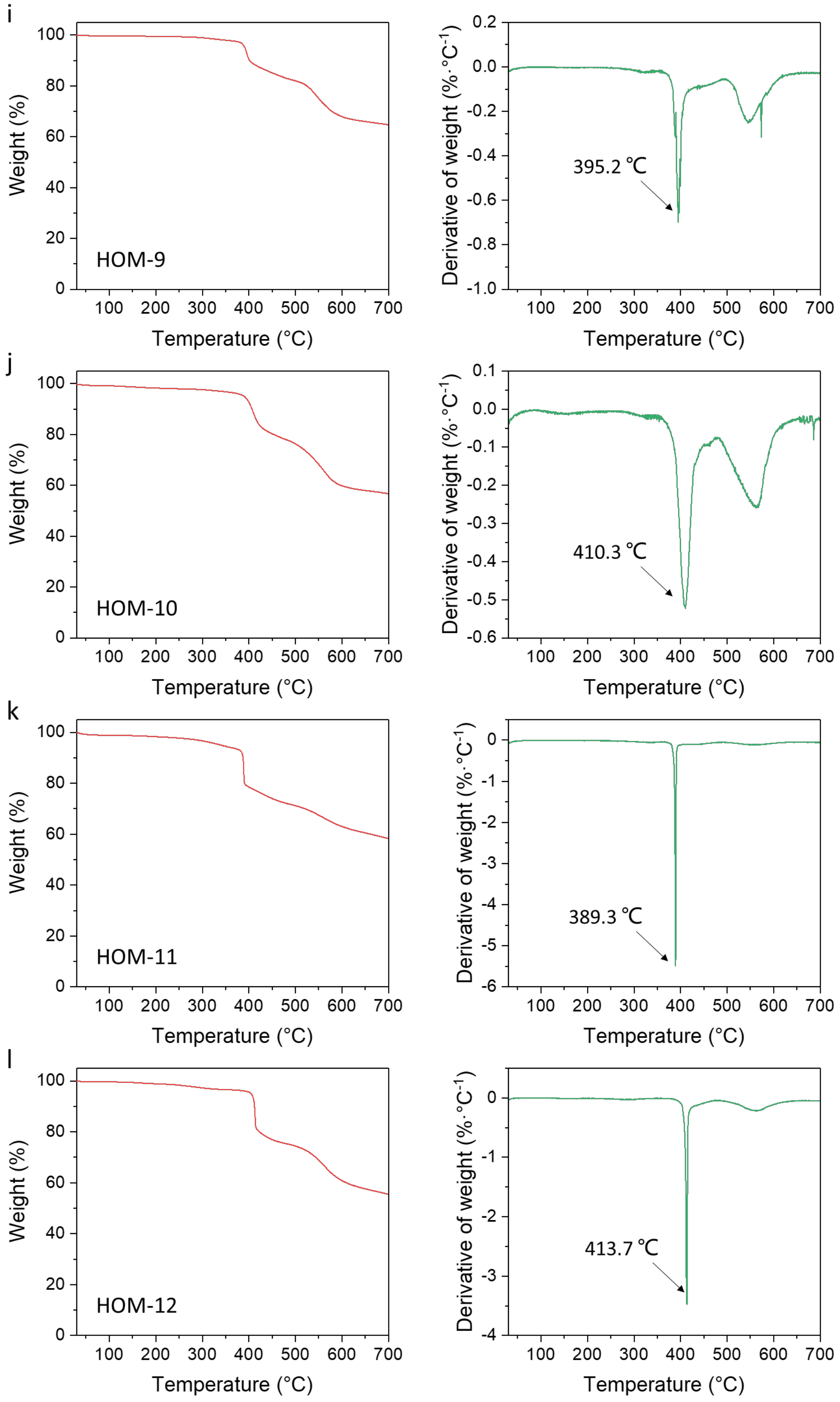
i
Weight (%)
HOM-9
Temperature (°C)
Derivative of weight (%·°C⁻¹)
395.2 ℃
j
Weight (%)
HOM-10
Temperature (°C)
Derivative of weight (%·°C⁻¹)
410.3 ℃
k
Weight (%)
HOM-11
Temperature (°C)
Derivative of weight (%·°C⁻¹)
389.3 ℃
l
Weight (%)
HOM-12
Temperature (°C)
Derivative of weight (%·°C⁻¹)
413.7 ℃

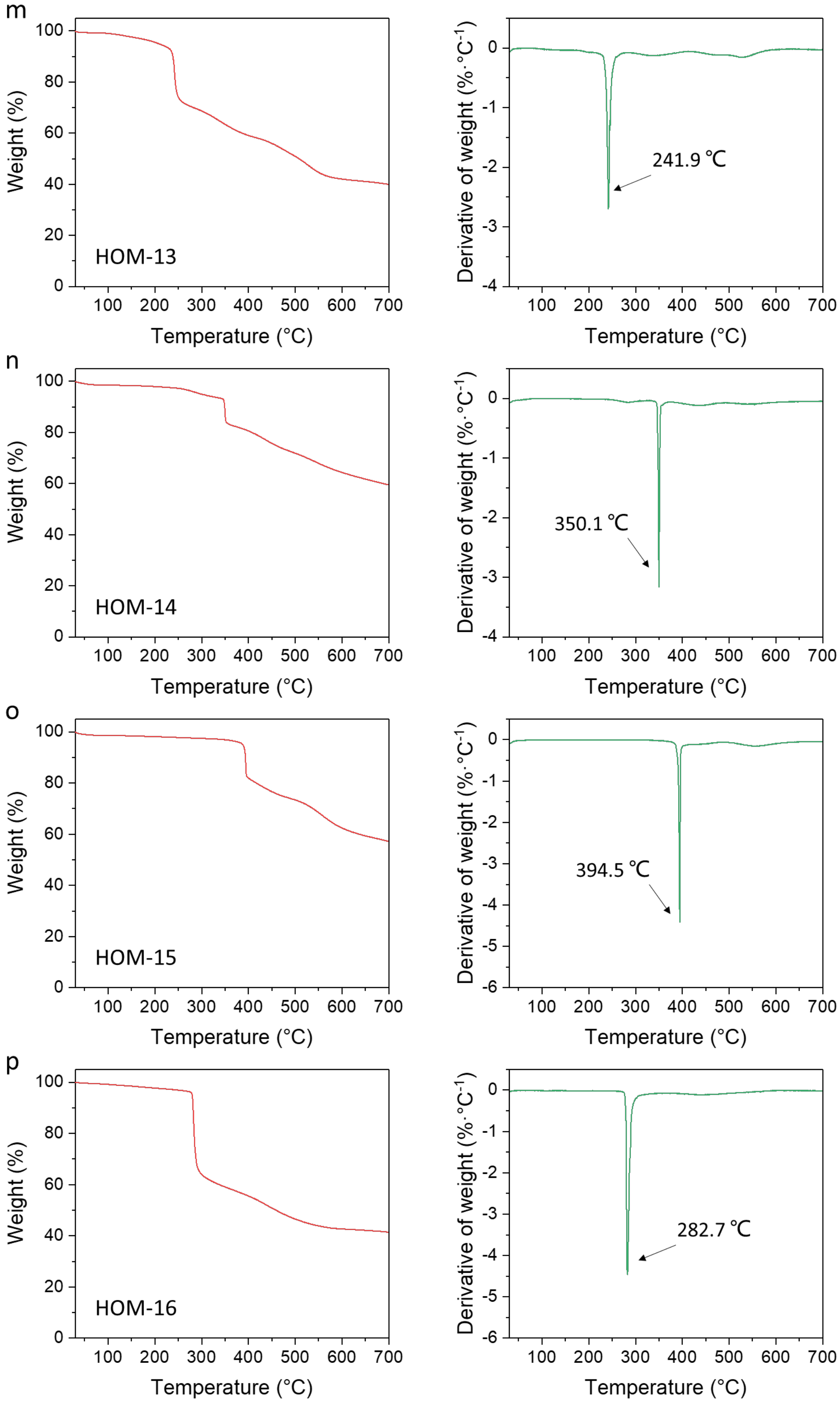

Supplementary Fig. 35 | a-p Left: Thermogravimetric analysis (TGA) of HOM-1~16. **Right:** The first derivative of the TGA curve.

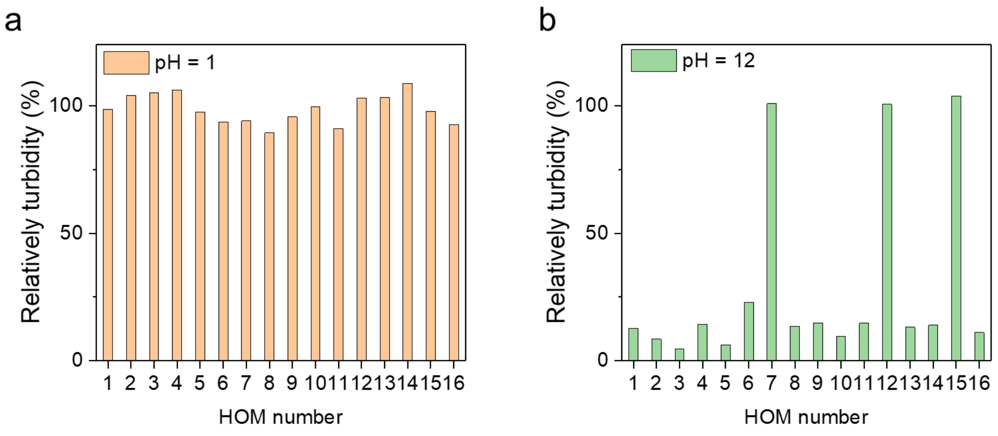

Supplementary Fig. 36 | Relatively turbidity of HOMs in HCl (a) or NaOH (b) solution. 1 mg HOMs were dispersed in 100 μL H_2O. 20 μL HOMs suspension was added into 180 μL HCl (pH = 1) or NaOH (pH=12) solution. The OD_{600} was measured as sample turbidity after 10 min vortex. Also, the reference turbidity was measured with HOMs dispersed in pure H_2O (1 mg mL^{-1}, 200 μL). The relative turbidity is the ratio of the sample turbidity in an HCl or NaOH solution to the reference turbidity.

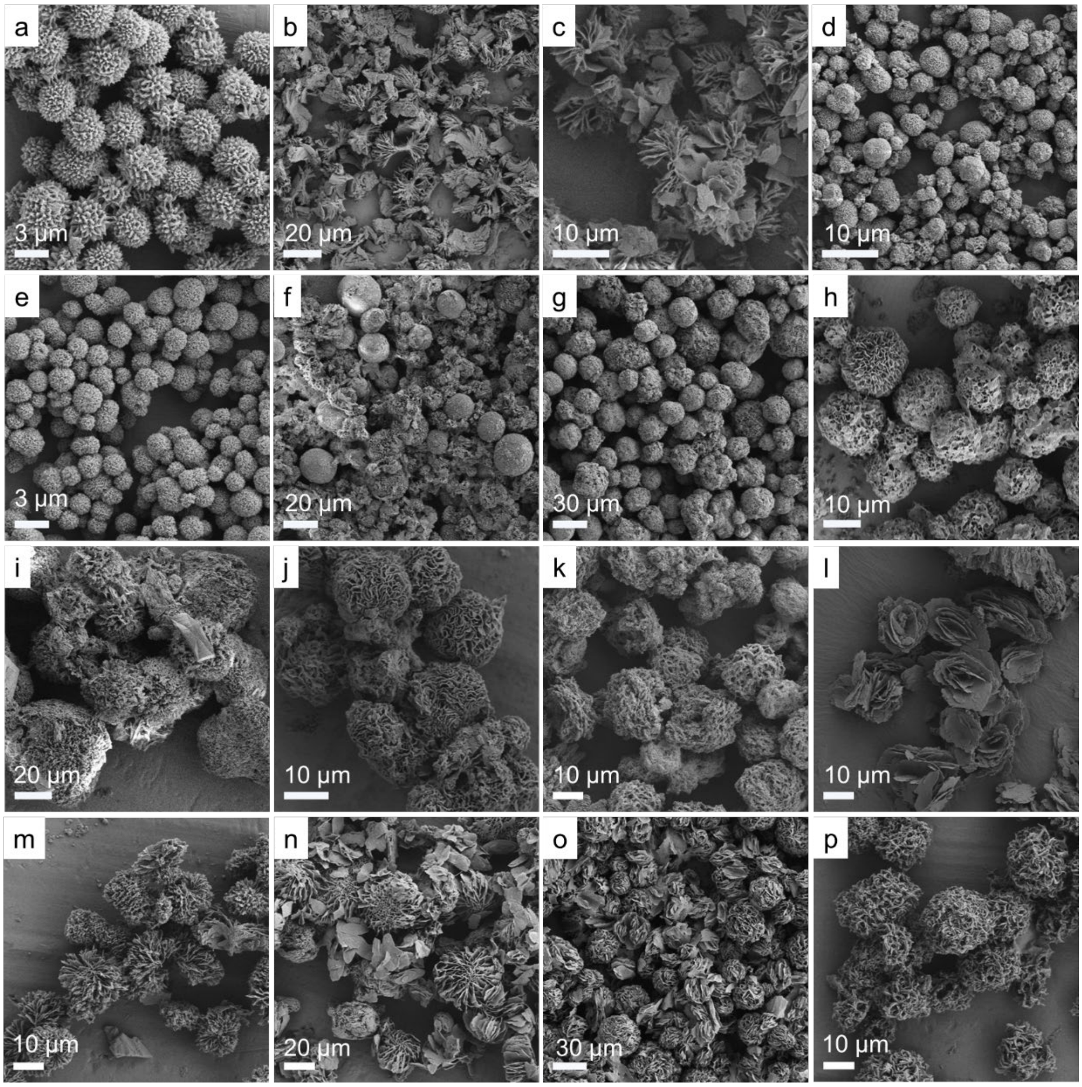

Supplementary Fig. 37 | a-p, SEM image of HOM-1~16 after being immersed in HCl solution (pH=1) for 12 hour.

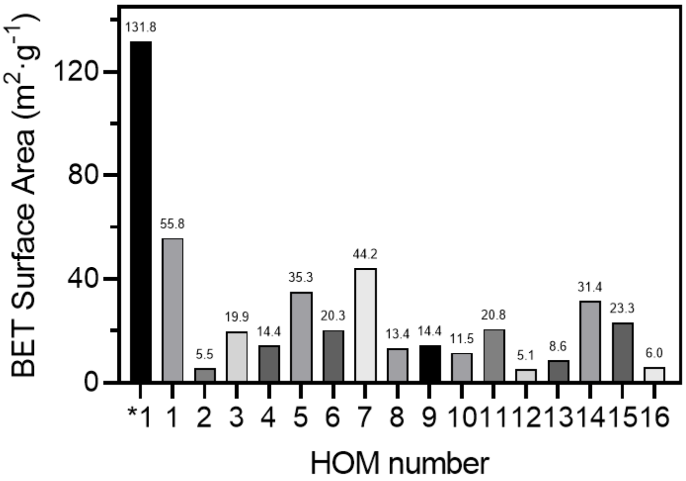

Supplementary Fig. 38 | The calculated surface area of HOMs based on N_2 isotherm adsorption curve (*1: fibrous HOM-1 synthesized in DMSO/MeOH combination).

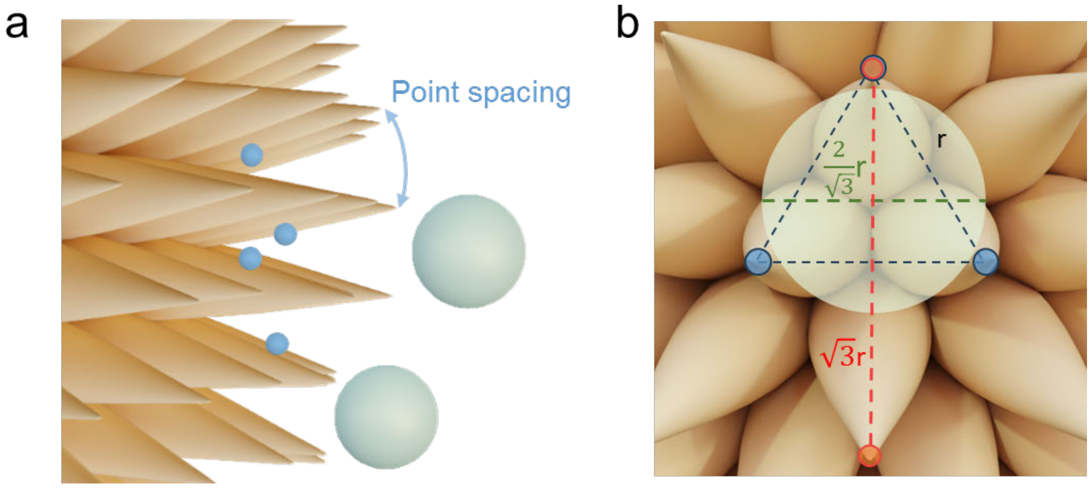

Supplementary Fig. 39 | a, Scheme of size selectivity on echinate HOMs surface. **b,** Scheme of the closest packing model of the surface tip.

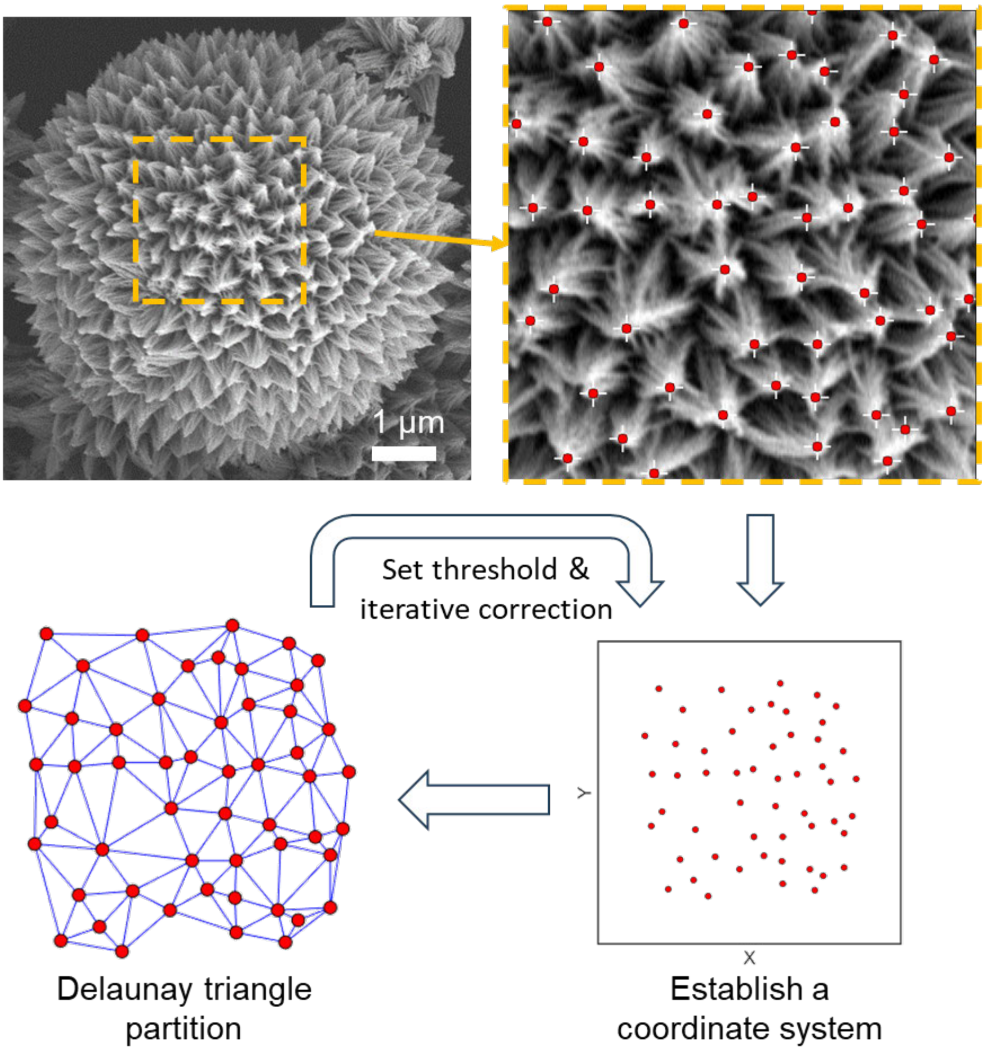

Supplementary Fig. 40 | Flowchart for the statistical analysis of point spacing based on the Delaunay triangle partition method.

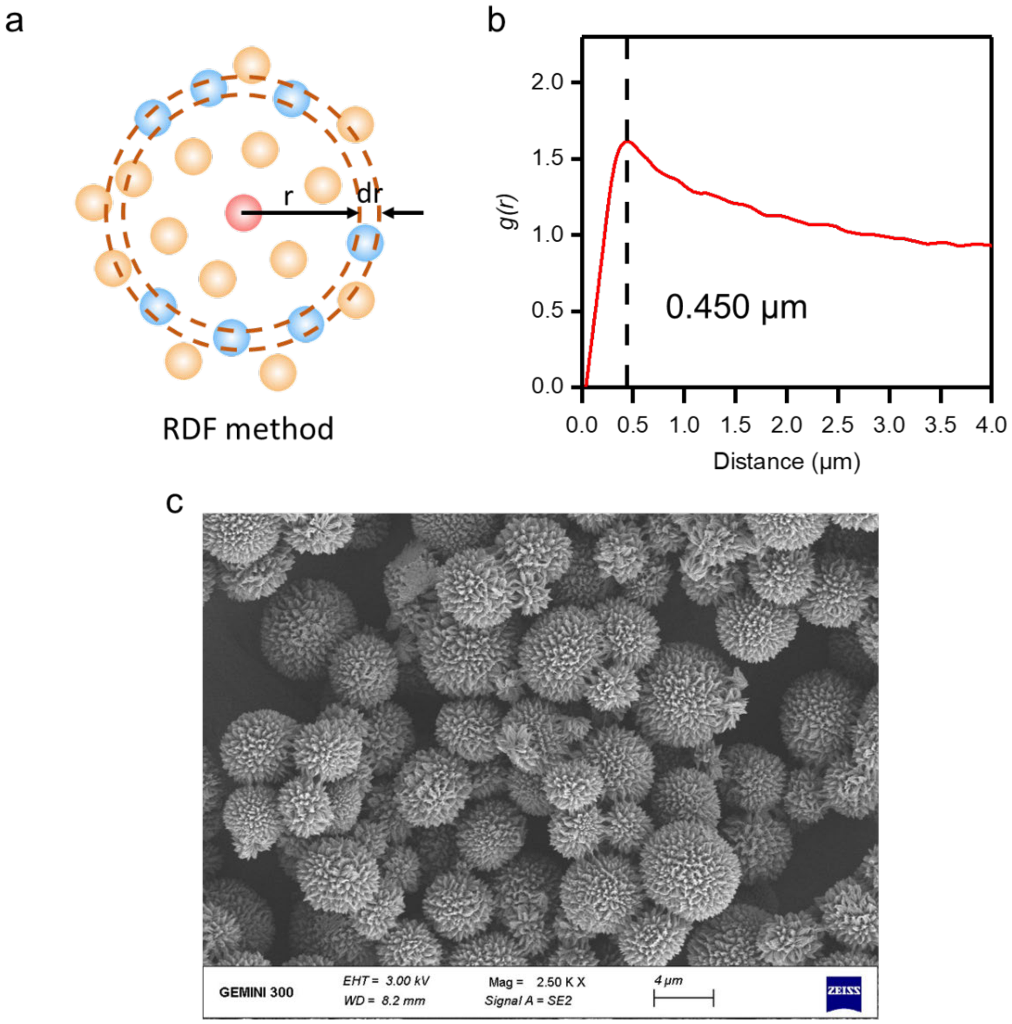

Supplementary Fig. 41 | a Schematic representation of radial distribution function calculation. **b** Statistical results of the RDF for multiple HOM-1 particles under a wide field of view. **c** The SEM image for the RDF analysis.

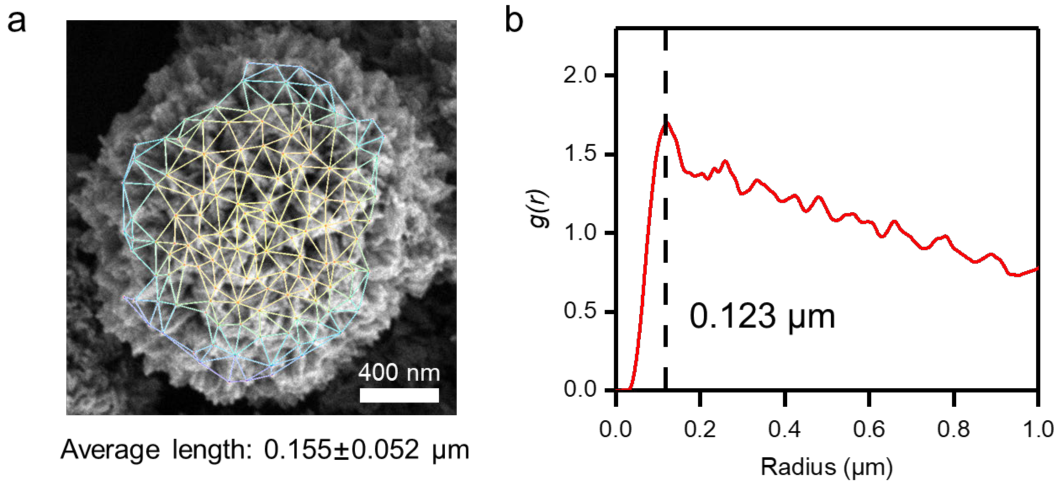

Supplementary Fig. 42 | a Results of Delaunay triangle partition for HOM-5 particle. **b** Statistical results of the RDF for HOM-5 particle.

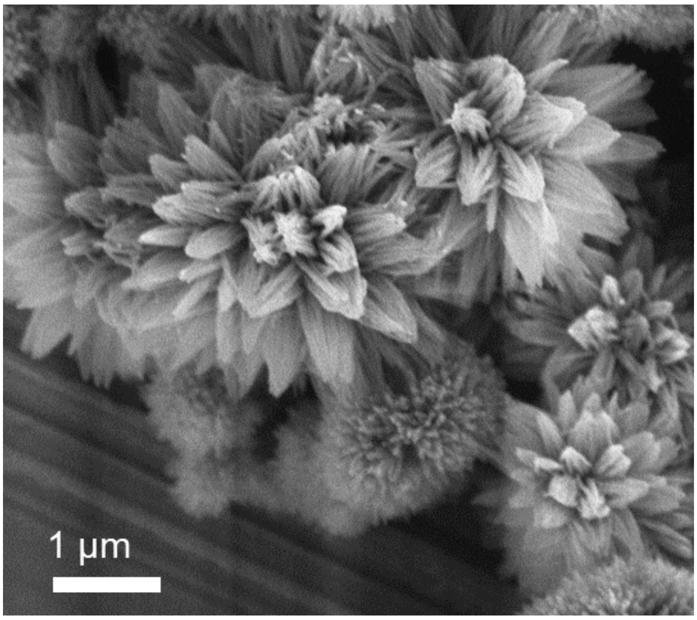

Supplementary Fig. 43 | SEM image of HOM-1 synthesized in NMP-EtOH. TP in NMP (20 mM, 100 μL) was mixed with ABA in EtOH (40 mM, 100 μL). After 1 hr of reaction, the supernatant was removed by centrifugation, and the precipitate was washed with EtOH three times before observation.

a

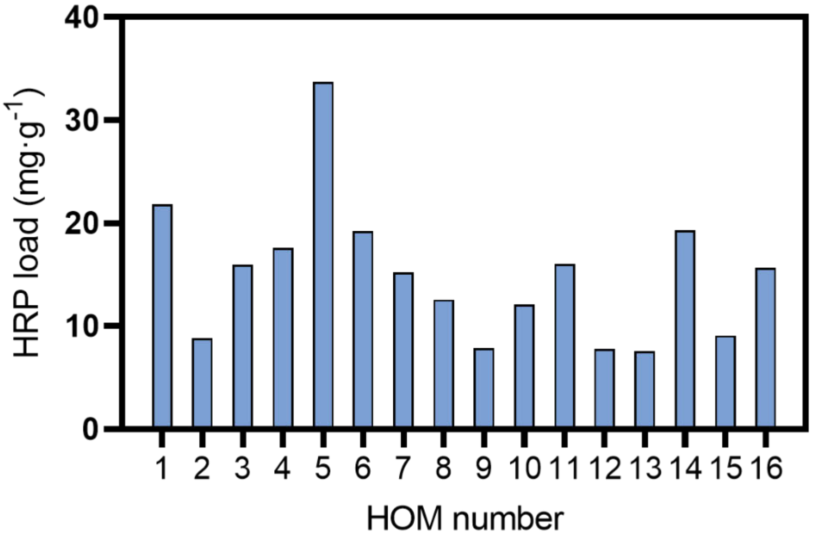

b

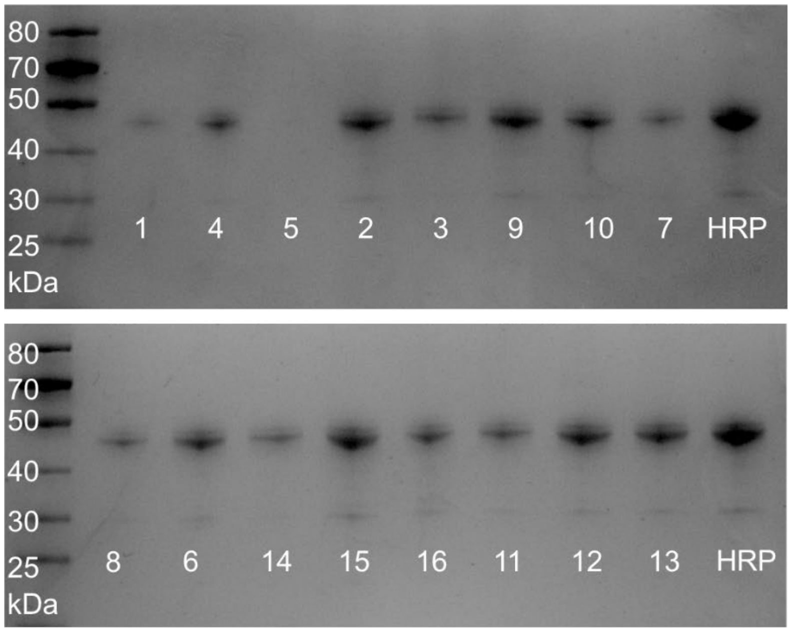

Supplementary Fig. 44 | a Loading capacity of HOMs for HRP. 10 mg ml^{-1} HOMs suspension was prepared by suspending HOMs in water. 1 mL suspension was taken and 4 μL of 10 mg mL^{-1} HRP solution was added. The samples were then oscillated at 10°C for 12 hrs. Subsequently, the samples were centrifuged at 10,000 x g for 3 minutes, and the supernatant was collected. SDS-PAGE analysis was performed. By comparing the difference in band intensity between HRP samples without or with the addition of HOMs, the adsorption amount of HRP on HOMs can be determined. **b** The photos of SDS-PAGE. Uncropped SDS-PAGE is shown in Supplementary Data 2a.

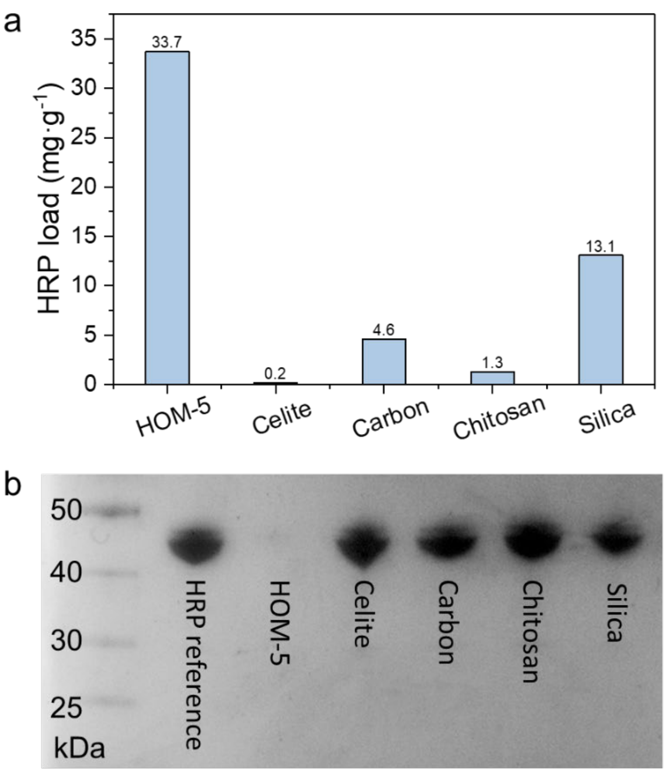

Supplementary Fig. 45 | a Loading capacity of HOM-5 for HRP, compared with classical carrier. 1 mL suspension was taken and 4 µL of 10 mg mL^{-1} HRP solution was added. The samples were then oscillated at 10°C for 12 hrs. Subsequently, the samples were centrifuged at 10,000 x g for 3 minutes, and the supernatant was collected. SDS-PAGE analysis was performed. By comparing the difference in band intensity between HRP samples without or with the addition of carrier, the adsorption amount of HRP can be determined. **b** The SDS-PAGE image of protein supernatant without treatment (the first lane) and after loading with various materials (the 2nd to the 6th lanes). Uncropped SDS-PAGE is shown in Supplementary Data 2b.

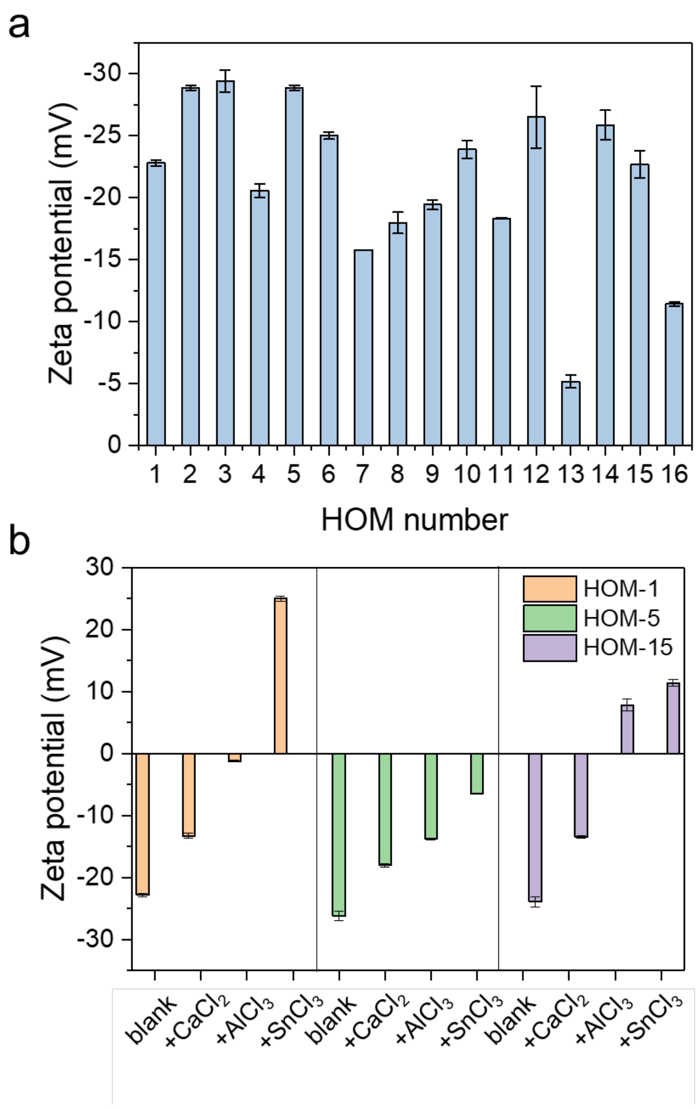

Supplementary Fig. 46 | a The Zeta potential of HOMs which were suspended in water (1 mg/mL). Error area represent standard deviation, n = 3. **b** The Zeta potential of HOM-1/5/15 after surface metallization with different metal ions.

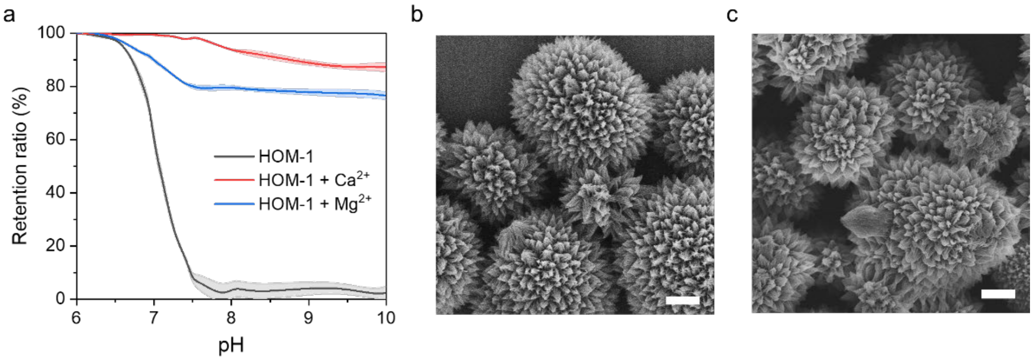

Supplementary Fig.47 | a The retention ratio curves of HOM-1 with or without metal ions, which were recorded by the absorbance of supernatant. **b** The SEM image of HOM-1 treated with Ca^{2+}. **c** The SEM image of HOM-1 treated with Mg^{2+}. The scale bars for (b) and (c) are both 1 μm.

a

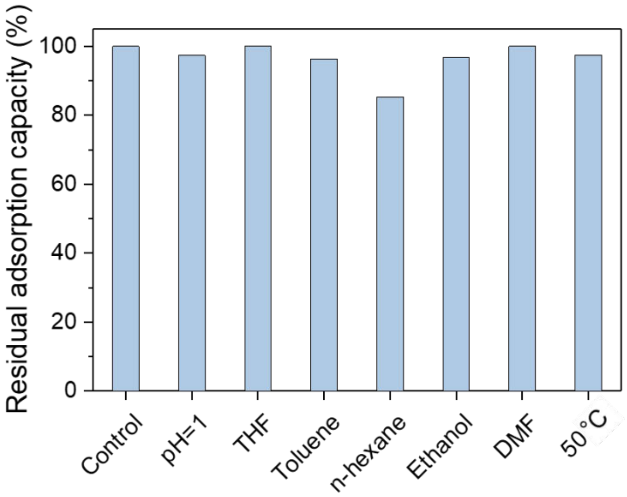

b

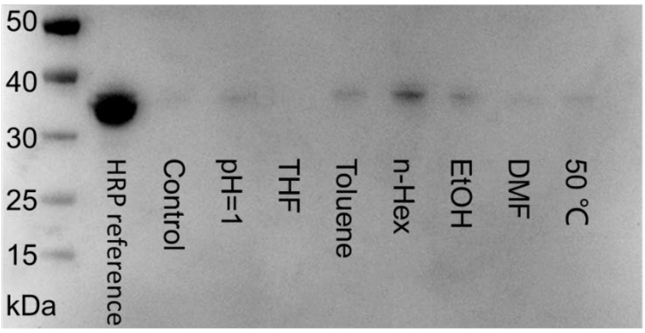

Supplementary Fig. 48 | a Residual HRP loading capacity of HOM-5 after treatment under different conditions. HOM-5 was suspended in organic solvents or aqueous solutions with varying pH, prepared as a 10 mg/mL suspension. After 14 hours, the original solvent was removed by centrifugation, and HOM-5 was washed three times with deionized water, and ultimately suspended in deionized water for testing its adsorption capacity for HRP. The 50°C group refers to heating the HOM-5 powder at 50°C for 14 hours, dispersing it in deionized water, and testing its adsorption capacity. **b** The SDS-PAGE image of protein supernatant without carrier (the first lane) and with HOM-5 after various treatment (the 2nd to the 9th lanes). Uncropped SDS-PAGE is shown in Supplementary Data 2d.

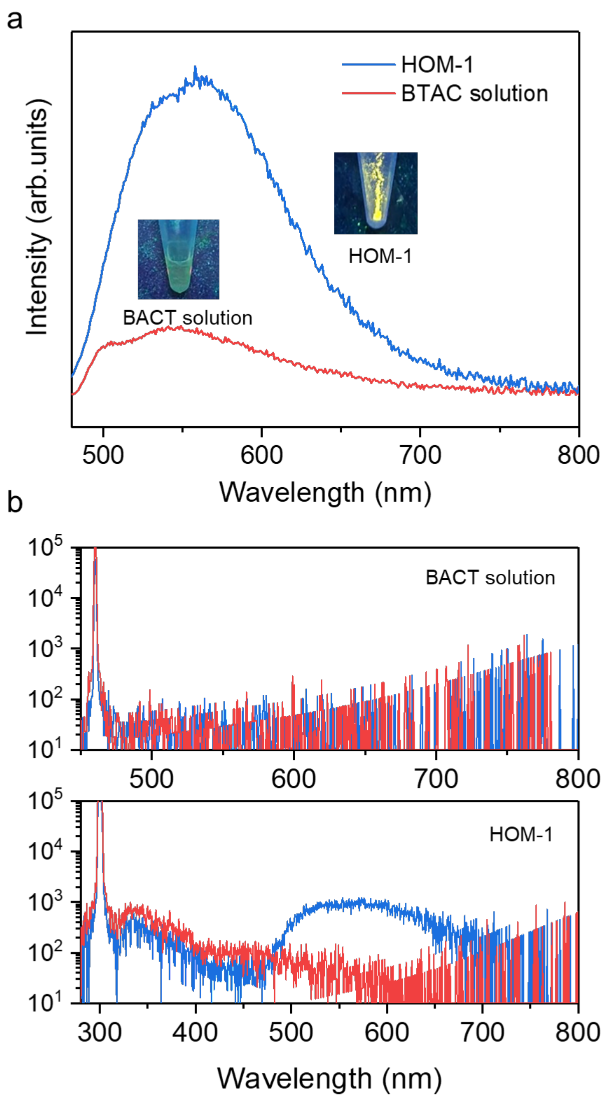

Supplementary Fig. 49 | a The emission spectrum of HOM-1 and BACT solution. **b** Emission spectrum and fluorescence quantum yield were measured using an Edinburgh FLS 1000 equipped with a xenon laser arc lamp and PMT-900 detector (Edinburgh, UK). (top) The fluorescence quantum yield (QY) of the solution of **3** was measured to be 0.47%. (bottom) QY of HOM-1, which was tested to be 15.62%. The analysis shows that HOM-1 has aggregation-induced emission (AIE) propriety.

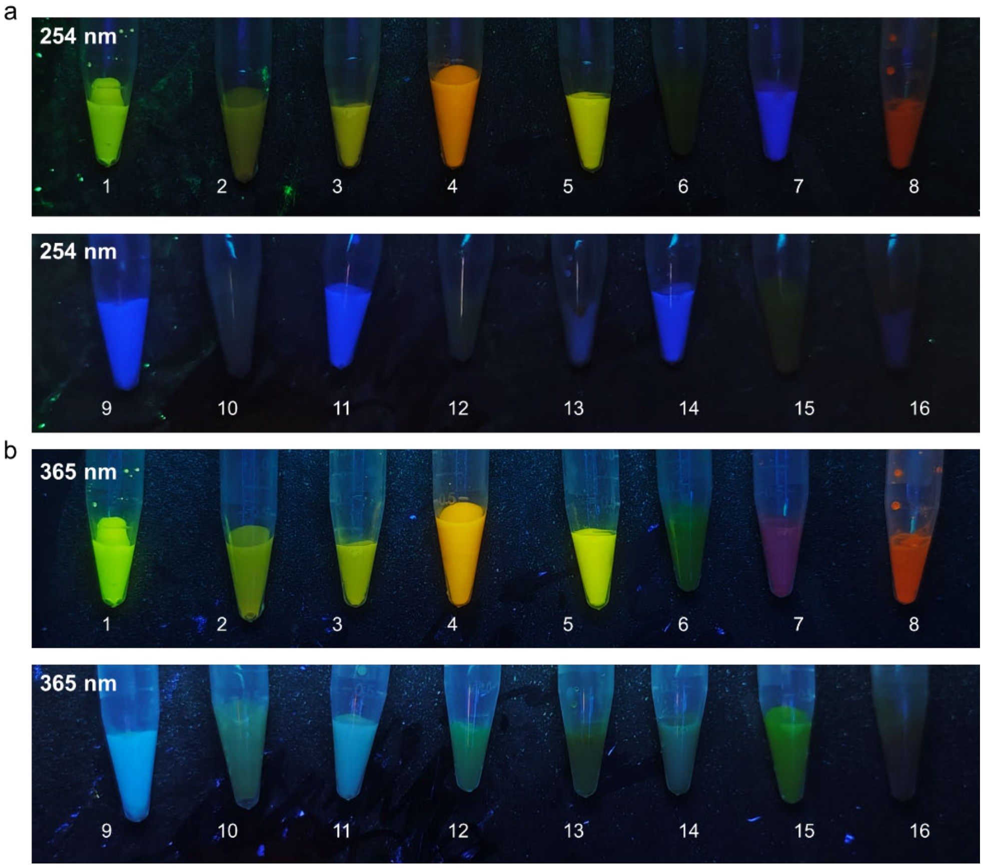

Supplementary Fig. 50 | a The photographs of the fluorescence of HOM-1~16 under excitation at a wavelength of 254 nm. **b** The photographs of the fluorescence of HOM-1~16 under excitation at a wavelength of 365 nm. All samples were 10 mg mL^{-1} in water.

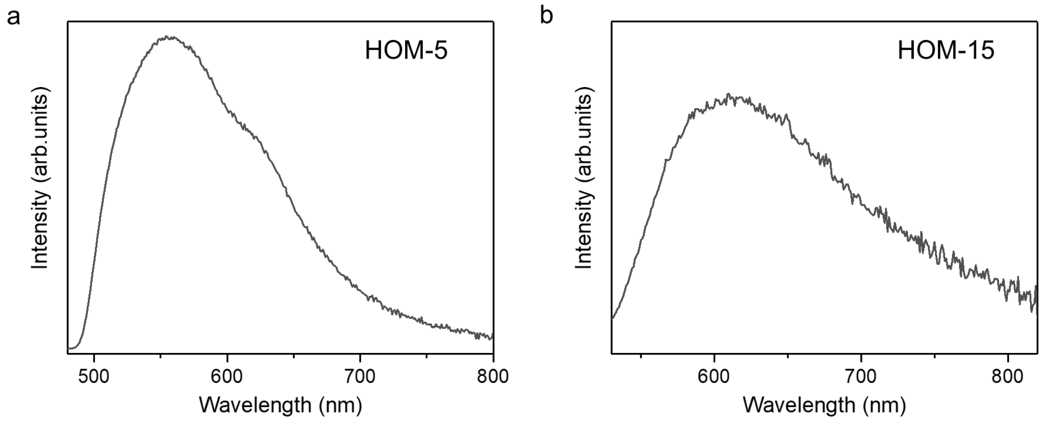

Supplementary Fig. 51 | The emission spectrum of HOM-5 (a) and HOM-15 (b). The spectra were recorded under excitation at a wavelength of 470 nm (a) and 520 nm (b), respectively.

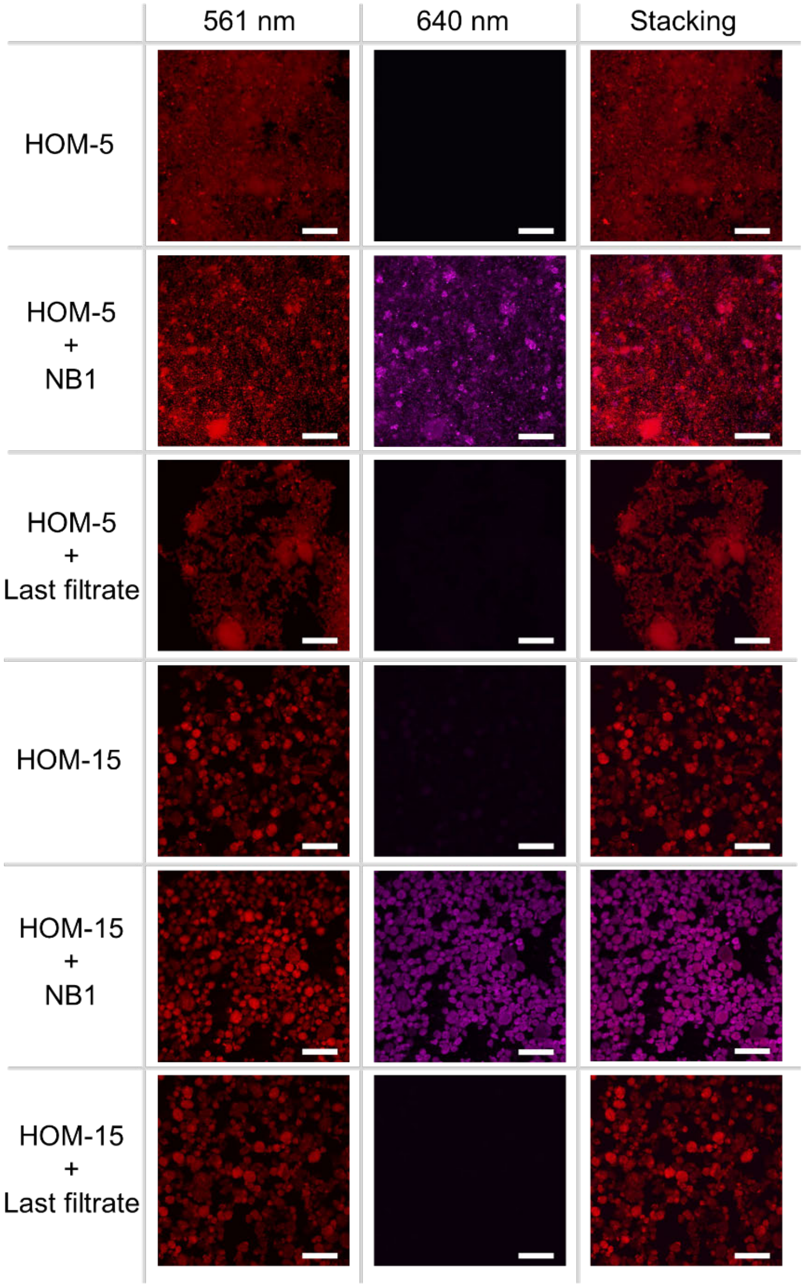

Supplementary Fig. 52 | Laser scanning confocal microscopy images of HOM-5 and HOM-15 loaded with Cy5-labeled bacteriophage (the scale bar is 40 μm). HOM-5/15 can be excited by a 561 nm laser. Phages capsid proteins stained with Cy5 can be excited by 640 nm laser, while HOMs did not generate signals at this wavelength. Therefore, when phages were adsorbed on HOMs, NB1@HOMs could be excited both at 561 nm and 640 nm. To exclude the influence of HOMs adsorbing the dye itself, we washed the bacteriophages by ultrafiltration. The HOMs immersed in the final filtrate did not exhibit any fluorescence signal at 640 nm, indicating that the aforementioned fluorescence indeed originated from the adsorbed phages. The images were taken using a laser scanning confocal microscope with a 10x objective lens (excitation wavelength: 561 nm and 640 nm, optical filter: 607 nm/36 and 685 nm/40, exposure time: 2.0 μs).

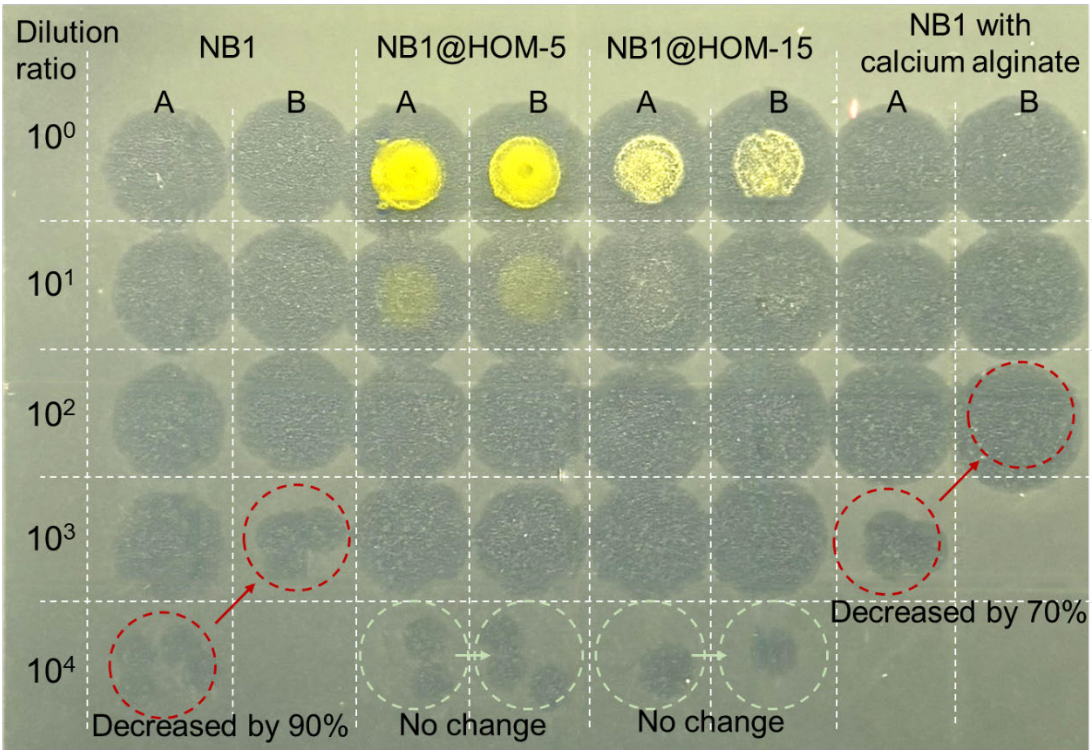

Supplementary Fig. 53 | Lysis plaques of phage with different carrier before (A column) and after (B column) heat treatment. The weight of the three carriers is controlled to be the same.

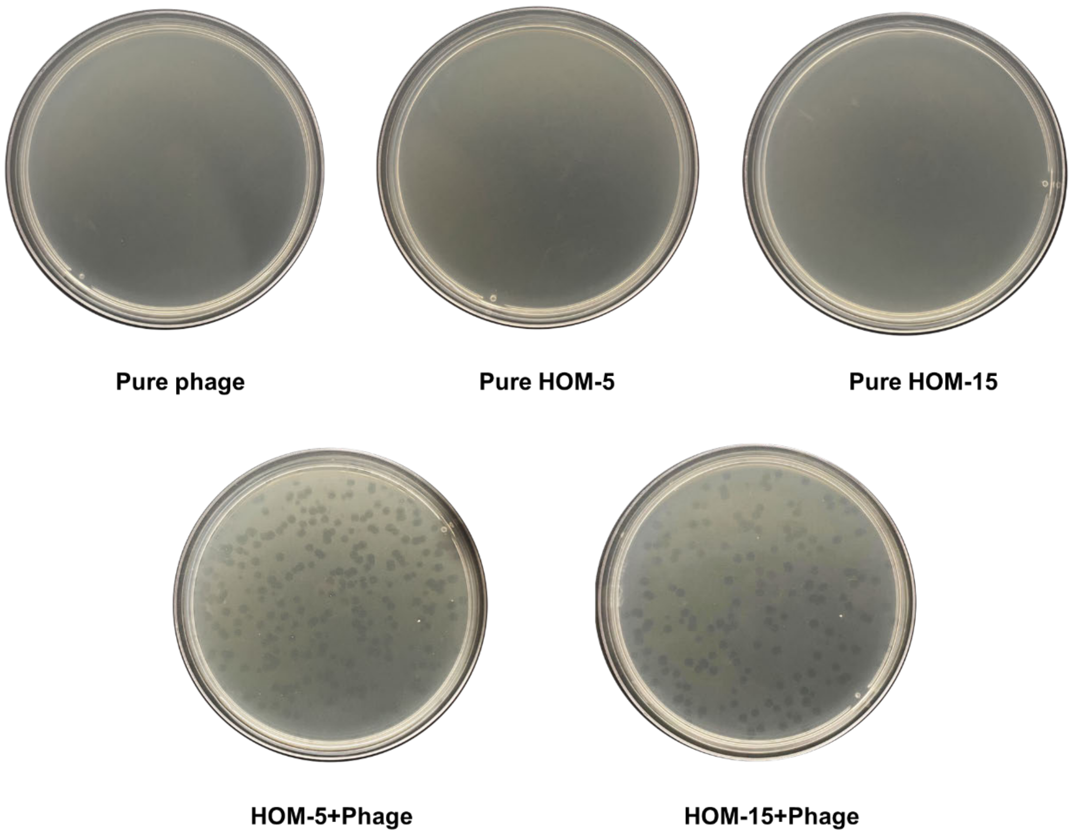

Supplementary Fig. 54 | Photo of the HOM-5 and -15 loaded phage forming lysis zones on the lawn of *Xoo* after heat treatment (60 °C for 1 h), compared with free phage and pure HOMs.

Supplementary Table

Supplementary Table 1: Synthetic conditions for HOMs

	Solvent A	Solvent B	Reaction time
HOM-1	DMAc	iPrOH	1 hr
HOM-2	DMF	Toluene	1 hr
HOM-3	DMF	iPrOH	1 hr
HOM-4	DMAc	1,4-dioxane	1 hr
HOM-5	NMP	iPrOH	1 hr
HOM-6	1,4-dioxane	MeOH	12 hrs
HOM-7	Toluene	MeOH	12 hrs
HOM-8	DMAc	iPrOH	12 hrs
HOM-9	Toluene	MeOH	24 hrs
HOM-10	Acetone	iPrOH	12 hrs
HOM-11	1,4-dioxane	MeCN	12 hrs
HOM-12	1,4-dioxane	MeOH	12 hrs
HOM-13	Toluene	MeCN	12 hrs
HOM-14	Toluene	Acetone	12 hrs
HOM-15	DMAc	MeOH	12 hrs
HOM-16	Toluene	iPrOH	12 hrs

Growth solvents and synthesis time for different HOMs. All the samples were synthesized by the following method. 15 mL 20mM aldehyde of A solution was mixed with 15 mL 40mM amine of B solution. After briefly shaking to mix, the reaction mixture was kept for a certain time (reaction time). Then, the precipitate was isolated by centrifugation (10000 x g, 5min), and washed by solvent B three times.

Supplementary Table 2: HOMs growth and characteristics data index

<table>
<tr><th>No.</th><th>NMR/MS</th><th>Growth kinetics</th><th>PXRD</th><th>Fluorescence</th><th>BET surface</th><th>Surface parameters</th><th>Zeta potential</th><th>Stability</th><th>Protein loading capacity</th></tr>
<tr><td>HOM-1</td><td>Fig. 2</td><td>Fig. 3</td><td>Fig. 2, S8</td><td>Fig. S49, S50</td><td>Fig. S8, S38</td><td>Fig. 6, S40,41</td><td rowspan="16">Fig. 46</td><td>Fig. S34, S35, S36, S37, S47</td><td rowspan="16">Fig. 44</td></tr>
<tr><td>HOM-2</td><td colspan="3">Fig. S18</td><td>Fig. S18, S50</td><td rowspan="15">Fig. 38</td><td rowspan="3">Fig. 6</td><td rowspan="15">Fig. S34, S35, S36, S37</td></tr>
<tr><td>HOM-3</td><td colspan="3">Fig. S19</td><td>Fig. S19, S50</td></tr>
<tr><td>HOM-4</td><td colspan="3">Fig. S20</td><td>Fig. S20, S50</td></tr>
<tr><td>HOM-5</td><td colspan="3">Fig. S21</td><td>Fig. S21, S50, S51</td><td>Fig. 6, S42</td></tr>
<tr><td>HOM-6</td><td colspan="3">Fig. S22</td><td>Fig. S24, S50</td><td rowspan="11">Fig. 6</td></tr>
<tr><td>HOM-7</td><td colspan="3">Fig. S23</td><td>Fig. S23, S50</td></tr>
<tr><td>HOM-8</td><td colspan="3">Fig. S24</td><td>Fig. S24, S50</td></tr>
<tr><td>HOM-9</td><td colspan="3">Fig. S25</td><td>Fig. S25, S50</td></tr>
<tr><td>HOM-10</td><td colspan="3">Fig. S26</td><td>Fig. S26, S50</td></tr>
<tr><td>HOM-11</td><td colspan="3">Fig. S27</td><td>Fig. S27, S50</td></tr>
<tr><td>HOM-12</td><td colspan="3">Fig. S28</td><td>Fig. S28, S50</td></tr>
<tr><td>HOM-13</td><td colspan="3">Fig. S29</td><td>Fig. S29, S50</td></tr>
<tr><td>HOM-14</td><td colspan="3">Fig. S30</td><td>Fig. S30, S50</td></tr>
<tr><td>HOM-15</td><td colspan="3">Fig. S31</td><td>Fig. S31, S50, S51</td></tr>
<tr><td>HOM-16</td><td colspan="3">Fig. S32</td><td>Fig. S32, S50</td></tr>
</table>

Supplementary Fig. is abbreviated as Fig. S

Supplementary Table 3: Comparison of HRP loading capacity in this work to other reported carriers

Carrier	Loading Capacity	Reference
Chitosan–halloysite hybrid-nanotubes	21.5 mg/g	9
poly(GMA-MMA)	3.35 mg/g	10
Magnetic biochar	65 mg/g	11
SOM-ZIF-8	71.2 mg/g	12
Calcium alginate	8.9 mg/g	13
Silica nanoparticles	8.06 mg/g	14
poly (Pro-Glu) modified silica gel	16.8 mg/g	15
multi-walled-carbon-nanotube/cordierite composite	1.34 mg/g	16
HOM-5	33.7 mg/g	This work

Supplementary References

1 Chen, Y. *et al.* Self-Assembly of a Purely Covalent Cage with Homochirality by Imine Formation in Water. *Angew. Chem. Int. Ed.* **60**, 18815-18820 (2021).

2 Zhao, Y. & Truhlar, D. G. Density functionals with broad applicability in chemistry. *Acc. Chem. Res.* **41**, 157-167 (2008).

3 Angelerou, M. G. F. *et al.* Supramolecular Nucleoside-Based Gel: Molecular Dynamics Simulation and Characterization of Its Nanoarchitecture and Self-Assembly Mechanism. *Langmuir* **34**, 6912-6921 (2018).

4 Markvardsen, A., David, W., Johnson, J. & Shankland, K. A probabilistic approach to space-group determination from powder diffraction data. *Acta Crystallographica Section A: Foundations of Crystallography* **57**, 47-54 (2001).

5 Kumar, P. *et al.* Photonically active bowtie nanoassemblies with chirality continuum. *Nature* **615**, 418-424 (2023).

6 Engel, G., Wilke, S., König, O., Harris, K. & Leusen, F. PowderSolve–a complete package for crystal structure solution from powder diffraction patterns. *J. Appl. Crystallogr.* **32**, 1169-1179 (1999).

7 Toby, B. H. & Von Dreele, R. B. GSAS-II: the genesis of a modern open-source all purpose crystallography software package. *J. Appl. Crystallogr.* **46**, 544-549 (2013).

8 Jiang, S. & Jiang, W. Reliable image matching via photometric and geometric constraints structured by Delaunay triangulation. *ISPRS Journal of Photogrammetry and Remote Sensing* **153**, 1-20 (2019).

9 Zhai, R. *et al.* Chitosan–halloysite hybrid-nanotubes: Horseradish peroxidase immobilization and applications in phenol removal. *Chem. Eng. J.* **214**, 304-309 (2013).

10 Bayramoğlu, G. & Arıca, M. Y. Enzymatic removal of phenol and p-chlorophenol in enzyme reactor: Horseradish peroxidase immobilized on magnetic beads. *J. Hazard. Mater.* **156**, 148-155 (2008).

11 Zhang, H. & Hay, A. G. Magnetic biochar derived from biosolids via hydrothermal carbonization: Enzyme immobilization, immobilized-enzyme kinetics, environmental toxicity. *J. Hazard. Mater.* **384**, 121272 (2020).

12 Li, S.-F., Zhai, X.-J., Zhang, C., Mo, H.-L. & Zang, S.-Q. Enzyme immobilization in highly ordered macro–microporous metal–organic frameworks for rapid biodegradation of hazardous dyes. *Inorganic Chemistry Frontiers* **7**, 3146-3153 (2020).

13 Jonović, M. *et al.* Immobilization of Horseradish Peroxidase on Magnetite-Alginate Beads to Enable Effective Strong Binding and Enzyme Recycling during Anthraquinone Dyes' Degradation. *Polymers* **14** (2022).

14 Sha, Y. *et al.* Construction of co-immobilized multienzyme systems using DNA-directed immobilization technology and multifunctionalized nanoparticles. *Colloids and Surfaces B: Biointerfaces* **229**, 113443 (2023).

15 Zheng, X. *et al.* Enrichment of IgG and HRP glycoprotein by dipeptide-based

polymeric material. *Talanta* **241**, 123223 (2022).
16 Li, Z. L. *et al.* Preparation of a novel multi-walled-carbon-nanotube/cordierite composite support and its immobilization effect on horseradish peroxidase. *Process Saf. Environ. Prot.* **107**, 463-467 (2017).
17 Li, Q. *et al.* Shear Stress Triggers Ultrathin-Nanosheet Carbon Nitride Assembly for Photocatalytic H2O2 Production Coupled with Selective Alcohol Oxidation. *J. Am. Chem. Soc.* (2023).
18 Li, T. *et al.* Large-Scale Self-Assembly of 3D Flower-like Hierarchical Ni/Co-LDHs Microspheres for High-Performance Flexible Asymmetric Supercapacitors. *ACS Appl. Mater. Interfaces* **8**, 2562-2572 (2016).
19 Xu, Z. *et al.* Nitrogen-Doped Porous Carbon Superstructures Derived from Hierarchical Assembly of Polyimide Nanosheets. *Adv. Mater.* **28**, 1981-1987 (2016).
20 Zhang, F., Bao, Y., Ma, S., Liu, L. & Shi, X. Hierarchical flower-like nickel phenylphosphonate microspheres and their calcined derivatives for supercapacitor electrodes. *J. Mater. Chem. A* **5**, 7474-7481 (2017).
21 Li, Q. *et al.* Hierarchical MoS2/NiCo2S4@C urchin-like hollow microspheres for asymmetric supercapacitors. *Chem. Eng. J.* **380**, 122544 (2020).
