# Supplementary material for: Hierarchical organic microspheres from diverse molecular building blocks
Source: Nat Commun. 2024 Jun 13;15:5041. doi: 10.1038/s41467-024-49379-7 (PMC11176358; doi:10.1038/s41467-024-49379-7)
Supplement: Supplementary file 3 — Description of Additional Supplementary Files [file 41467_2024_49379_MOESM3_ESM.pdf]

Description of Additional Supplementary Files

File Name: Supplementary Data 1
Description: Delaunay triangulation data.

File Name: Supplementary Data 2
Description: Uncropped copies of all gel images.

File Name: Supplementary Code 1-4
Description:

Supplementary Code 1: Dihedral angle statistics.

Supplementary Code 2: Estimation of the Z-coordinate value.

Supplementary Code 3: Delaunay triangulation.

Supplementary Code 4: Radial distribution function.
