# Supplementary material for: Hierarchical organic microspheres from diverse molecular building blocks
Source: Nat Commun. 2024 Jun 13;15:5041. doi: 10.1038/s41467-024-49379-7 (PMC11176358; doi:10.1038/s41467-024-49379-7)
Supplement: Supplementary file 5 — Supplementary Data 1 [file 41467_2024_49379_MOESM5_ESM.pdf]

Supplementary Data 1

HOM-1-1

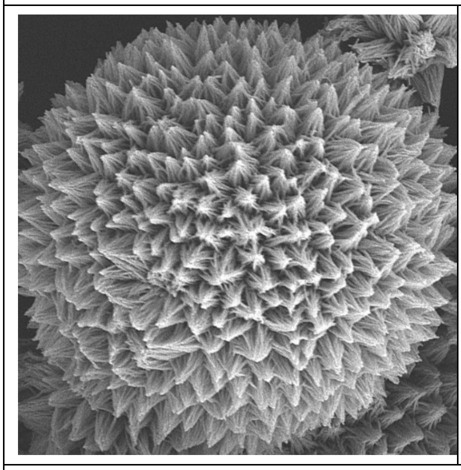

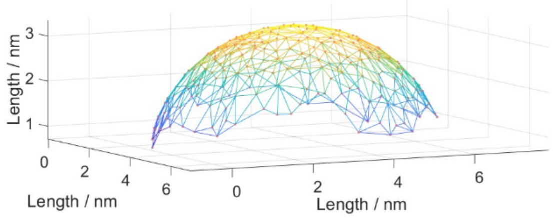

Average length = 0.493±0.167μm

Modelling function: $\frac{(x-a)^2}{A}+\frac{(y-b)^2}{B}+\frac{z^2}{C}=1$ (a=3.41; b=3.39; A=C=3.36; B=3.33)

x	y	z
4.400	6.267	1.377
4.867	6.144	1.205
4.300	5.744	2.205
2.811	5.900	2.131
4.733	5.950	1.695
3.433	5.900	2.212
1.989	5.889	1.715
2.222	5.867	1.913
3.078	5.678	2.422
5.178	5.778	1.536
4.644	5.711	2.070
2.467	5.711	2.222
3.878	5.644	2.431
4.967	5.456	2.126
3.400	5.589	2.526
5.467	5.111	2.008
4.600	5.011	2.683
5.006	5.644	1.889
1.956	5.344	2.305
1.517	5.483	1.810
3.744	5.333	2.710
3.011	5.211	2.787
2.267	5.144	2.621
1.489	5.167	2.100
4.333	5.056	2.759
6.067	4.867	1.411
3.278	5.000	2.940
1.967	5.044	2.538
5.844	4.989	1.658
2.744	4.911	2.916
5.667	4.911	1.956
4.200	4.856	2.912
1.844	4.856	2.583
3.100	4.800	3.030
2.211	4.778	2.812
0.933	4.789	1.786
3.678	4.656	3.097
1.544	4.733	2.448
5.394	4.633	2.402
5.867	4.656	1.898
4.200	4.556	3.046
2.900	4.444	3.147
3.356	4.478	3.176
1.422	4.433	2.500
5.456	4.311	2.495
5.011	4.211	2.833
1.211	4.244	2.394
4.567	4.122	3.065
3.867	4.222	3.221
6.289	4.133	1.554
3.200	4.067	3.284
2.544	4.078	3.173
5.911	4.139	2.108
1.278	4.000	2.527
2.244	3.956	3.101
5.156	3.933	2.815
4.522	3.900	3.127
2.678	3.878	3.243
4.144	3.822	3.249
3.617	3.400	3.353
6.411	3.744	1.459
5.717	4.489	2.173
0.711	3.772	1.970
6.256	3.656	1.758
1.344	3.700	2.634
1.811	3.678	2.943
5.867	3.700	2.265
2.100	3.656	3.084
4.844	3.439	3.036
2.933	3.456	3.326
5.378	3.289	2.718
2.411	3.489	3.208
0.956	3.489	2.297
4.444	3.411	3.195
3.233	3.400	3.355
2.533	3.378	3.244
0.844	3.389	2.174
6.389	3.333	1.543
1.889	3.311	2.997
1.411	3.278	2.701

6.000	3.044	2.105
2.078	3.189	3.079
1.478	3.178	2.743
5.033	3.111	2.925
6.556	3.144	1.139
0.778	3.133	2.077
3.211	3.100	3.341
4.267	2.944	3.216
2.867	3.011	3.294
3.978	3.022	3.289
3.578	2.867	3.313
2.478	2.889	3.189
4.744	2.756	3.014
5.456	2.889	2.612
1.267	2.900	2.543
6.378	2.878	1.477
5.333	2.722	2.667
5.883	2.633	2.136
2.978	2.589	3.232
1.789	2.678	2.856
3.844	2.578	3.228
4.278	2.567	3.136
4.933	2.489	2.850
4.100	2.478	3.155
1.600	2.533	2.697
4.733	2.378	2.912
3.422	2.300	3.174
2.611	2.233	3.048
2.333	2.211	2.952
1.311	2.211	2.341
2.922	2.189	3.095
4.156	2.133	3.018
5.544	2.078	2.226
6.117	2.128	1.519
3.422	2.056	3.077
5.311	2.067	2.423
4.700	2.011	2.769
4.356	2.000	2.900
3.944	1.789	2.895
2.900	1.822	2.919
3.278	1.767	2.930
5.289	1.778	2.256
6.000	1.767	1.367
5.278	1.633	2.152

6.067	1.567	0.902
3.622	1.522	2.772
4.678	1.489	2.446
4.200	1.444	2.607
3.333	1.433	2.716
5.544	1.322	1.534
3.633	1.322	2.622
5.189	5.511	1.882
1.156	4.811	2.045
2.367	1.744	2.728
5.767	1.700	1.672
3.267	1.567	2.807
3.144	6.256	1.697
5.067	4.022	2.850
1.133	3.711	2.453
2.100	1.733	2.604
3.000	5.400	2.650
4.067	5.211	2.736
0.922	4.556	1.934
5.111	1.578	2.243
1.611	2.878	2.792
0.489	2.644	1.487
0.922	1.933	1.718
3.933	1.289	2.551
4.867	1.122	1.977
3.956	6.344	1.458
2.056	2.178	2.822
2.389	0.800	1.849
0.750	4.367	1.808
0.378	3.011	1.405
1.267	1.678	1.930
4.656	6.156	1.399
3.567	5.100	2.880
0.706	2.661	1.859
2.278	2.644	3.073
2.556	2.067	2.963
1.700	1.767	2.386
2.967	1.556	2.768
2.278	1.156	2.220
4.256	0.644	1.697
1.156	3.322	2.494
2.011	0.944	1.802
0.967	5.167	1.462
2.833	1.878	2.937

1.156	1.533	1.646
5.444	1.089	1.317
4.644	1.044	2.036
1.833	2.133	2.683
2.211	0.867	1.837
4.178	6.411	1.194
0.856	4.944	1.529
6.067	2.889	1.986
1.178	2.744	2.428
1.722	5.733	1.698
0.344	3.833	1.310
5.856	3.444	2.298
0.511	2.967	1.651
4.622	1.344	2.354
6.306	2.361	1.338
2.689	1.311	2.523
1.789	1.133	1.866
3.767	1.022	2.333
4.178	1.056	2.267
2.800	1.311	2.552
1.933	0.967	1.771
0.911	3.256	2.246
0.778	2.111	1.647
1.311	2.022	2.234
3.444	1.056	2.394
1.622	4.444	2.642
3.367	6.467	1.293
5.933	4.344	1.994
0.500	3.389	1.687
1.733	4.000	2.849
3.233	0.744	2.031
0.411	4.278	1.234
2.967	0.967	2.261
1.789	3.122	2.932
1.389	1.067	1.312
4.111	5.722	2.296
1.089	3.989	2.358
5.144	0.967	1.511
3.700	0.744	2.017
0.300	2.844	1.156
0.722	1.678	1.047
0.656	4.900	1.190
2.778	1.644	2.790
2.456	1.256	2.397

0.400	2.378	1.099
2.122	0.744	1.583
0.756	1.811	1.313
2.389	0.511	1.345
2.978	0.567	1.728
0.844	1.611	1.224
5.000	0.744	1.269
1.578	0.756	0.937
2.789	0.956	2.207
3.846	6.089	1.923

0.233	2.556	0.714
1.133	1.100	0.882
0.633	4.700	1.364
2.022	4.056	2.987
3.222	0.422	1.510
1.733	1.033	1.682
5.056	4.611	2.656
4.683	4.861	2.732
5.150	5.044	2.339
4.567	4.572	2.919

4.250	4.222	3.142
6.289	4.528	1.289
5.656	5.278	1.614
0.794	1.439	0.765
3.672	0.517	1.676
5.539	3.156	2.584
5.556	4.028	2.500
3.683	3.778	3.325
4.306	6.028	1.847
5.894	2.456	2.050

HOM-1-2

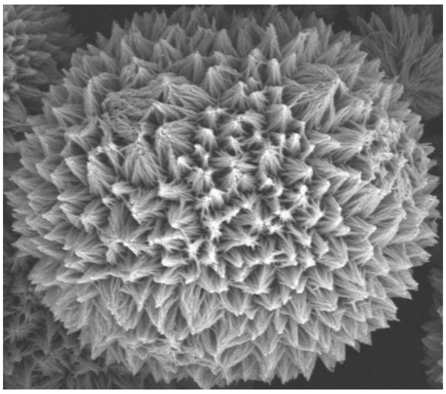

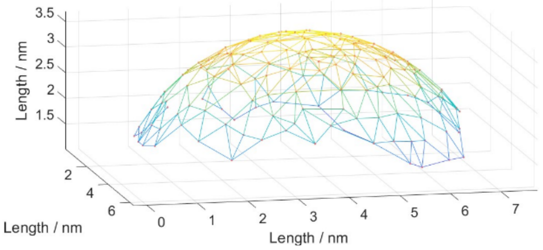

Average length = 0.547±0.167 μm

Modelling function: $\frac{(x-a)^2}{A^2}+\frac{(y-b)^2}{B^2}+\frac{z^2}{C^2}=1$ (a=3.75; b=3.30; A=C=3.70; B=3.24)

x	y	z
5.156	5.411	2.437
4.578	5.211	2.877
5.100	4.911	2.916
4.389	4.911	3.150
3.733	4.878	3.236
5.622	4.856	2.654
5.022	4.778	3.040
3.178	4.744	3.268
2.711	4.744	3.151
3.767	4.644	3.370
6.567	4.544	1.933
5.822	4.467	2.762
4.044	4.467	3.442
5.244	4.411	3.140
2.822	4.433	3.344
4.333	4.411	3.429
4.533	4.378	3.402
3.578	4.322	3.510
4.967	4.311	3.299
5.556	4.200	3.062
3.233	4.167	3.530
6.322	4.167	2.468
2.100	4.100	3.187
4.200	4.078	3.565
5.222	4.067	3.280
2.778	4.000	3.482
3.678	3.756	3.664
5.833	3.844	2.993
4.633	3.833	3.541
4.389	3.711	3.614
5.744	3.722	3.078
5.422	3.600	3.282
4.789	3.578	3.536
5.767	3.500	3.091
4.167	3.433	3.673
3.600	3.244	3.697
5.150	3.269	3.423
4.744	3.189	3.560
2.722	3.211	3.554
1.911	3.167	3.209
5.578	3.044	3.201
4.556	3.011	3.595
6.111	3.022	2.827
3.967	2.867	3.659
3.500	2.878	3.660
5.822	2.889	3.026
5.244	2.844	3.342
3.033	2.656	3.554
3.467	2.622	3.606
4.522	2.544	3.512
6.122	2.444	2.661
5.300	2.444	3.211
3.311	2.422	3.534
5.011	2.267	3.269
3.978	2.333	3.522
4.789	2.289	3.355
3.533	2.178	3.463
4.822	1.733	3.052
4.078	5.278	2.919
2.656	5.144	2.847
2.222	4.678	2.986
3.644	4.533	3.424
6.367	3.833	2.541
2.256	3.600	3.370
3.933	2.733	3.637
4.000	2.022	3.389
6.211	5.000	1.970
6.500	3.233	2.470
2.700	2.222	3.327
4.289	5.711	2.423
5.667	5.211	2.297
4.089	4.722	3.311
3.544	1.833	3.291
1.089	4.478	2.201
5.756	2.378	2.921
5.589	2.267	2.982
4.344	2.256	3.450
4.133	1.933	3.330
4.311	1.522	3.039
5.156	2.022	3.092
2.667	1.744	3.059
3.750	5.831	2.323
4.744	5.611	2.405
5.678	5.511	1.906
1.511	4.678	2.499
1.489	2.744	2.861
3.189	5.256	2.904
4.833	5.067	2.911
3.367	2.856	3.645

1.644	2.500	2.903
1.867	2.444	3.032
1.378	3.667	2.813
6.578	2.544	2.217
2.878	5.767	2.249
3.256	5.556	2.620
2.356	5.111	2.742
1.289	4.278	2.534
5.744	1.289	2.100
5.378	5.678	1.925
2.678	5.511	2.495
1.811	5.189	2.309
2.200	4.033	3.258
0.689	3.611	2.055
1.011	3.456	2.486
0.956	3.311	2.430
6.556	4.989	1.452
2.878	2.311	3.413
4.994	5.824	1.971
3.056	5.722	2.371
3.400	6.011	2.013
5.300	5.922	1.540
6.167	1.678	2.094
4.078	1.356	2.939
6.189	5.367	1.482
6.489	2.022	2.006

4.656	0.989	2.426
1.722	3.333	3.097
1.078	4.178	2.363
3.556	1.100	2.707
6.143	4.735	2.299
4.567	5.956	1.970
2.357	2.639	3.344
4.756	1.444	2.857
2.411	5.722	2.077
1.144	2.689	2.535
7.000	2.467	1.479
1.311	5.033	1.970
2.800	1.322	2.771
5.000	0.867	2.094
1.167	2.233	2.354
0.833	2.911	2.237
0.767	4.000	2.047
2.944	1.522	2.985
1.831	5.654	1.688
1.467	1.444	1.997
0.633	4.156	1.749
4.267	0.889	2.413
0.633	2.267	1.611
5.156	0.511	1.243
4.267	1.278	2.841
0.467	3.422	1.709

0.489	3.722	1.690
2.356	1.078	2.303
2.267	0.933	2.045
0.878	1.800	1.587
3.322	0.856	2.389
1.831	4.061	3.046
4.046	5.602	2.596
4.644	0.556	1.747
2.722	0.600	1.767
2.091	5.513	2.148
6.765	3.646	2.104
2.972	3.646	3.597
5.706	0.698	1.003
2.217	1.580	2.735
2.009	2.002	2.909
2.350	2.061	3.119
0.617	2.787	1.884
5.550	5.839	1.445
6.646	3.883	2.201
6.787	3.254	2.107
5.491	1.135	2.126
5.165	1.483	2.712
2.224	3.424	3.370
3.765	0.498	1.855
3.424	0.461	1.750
3.009	0.417	1.515

HOM-1-3

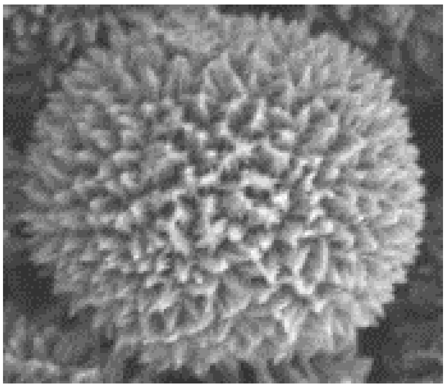

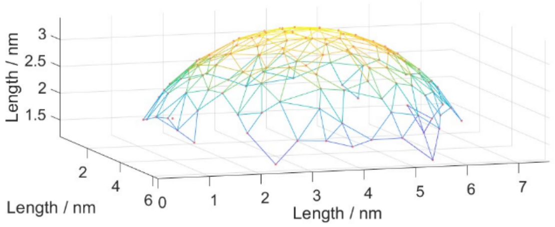

Average length = 0.568±0.154 μm

Modelling function: $\frac{(x-a)^2}{A^2}+\frac{(y-b)^2}{B^2}+\frac{z^2}{C^2}=1$ (a=3.77; b=3.18; A=C=3.43; B=3.05)

x	y	z
4.821	5.399	2.103
5.582	5.538	1.200
5.407	5.187	1.996
5.099	4.835	2.555
4.615	4.703	2.847
5.714	4.659	2.284
3.780	4.484	3.099
4.308	4.308	3.139
4.484	4.132	3.178
4.000	3.956	3.308
5.231	3.912	2.991
5.582	3.824	2.820
6.154	3.648	2.409
4.484	3.516	3.332
5.319	3.604	3.022
2.989	3.604	3.303
4.791	3.297	3.271
5.363	3.297	3.034
5.626	3.253	2.883
3.473	2.989	3.409
5.407	2.637	2.951
4.396	1.890	3.043
6.593	1.802	1.182
4.176	3.033	3.401
5.143	2.681	3.092
3.385	2.242	3.239
5.011	4.527	2.815
4.088	3.297	3.411
6.374	4.132	1.959
3.648	3.868	3.338
6.725	3.692	1.645
5.231	2.989	3.095
5.670	2.857	2.832
3.165	2.857	3.355
4.176	2.462	3.308
4.835	2.198	3.067
4.484	5.011	2.646
3.077	4.659	2.916
5.626	2.110	2.621
5.978	4.308	2.298
3.516	3.473	3.403
5.187	1.802	2.712
3.560	2.549	3.348
2.681	4.484	2.900
3.780	2.154	3.229
3.956	1.714	3.000
2.901	5.714	1.693
4.747	2.549	3.210
6.637	2.154	1.489
5.451	4.659	2.483
5.978	4.044	2.438
6.637	2.813	1.838
2.286	4.308	2.817
3.516	4.923	2.800
6.022	3.297	2.584
6.286	1.758	1.697
2.286	3.560	3.060
2.725	5.187	2.357
3.780	5.670	1.976
2.813	2.418	3.178
3.516	5.187	2.567
1.978	3.648	2.873
2.945	3.253	3.326
6.154	2.901	2.446
3.429	1.582	2.899
2.505	4.747	2.653
3.385	4.703	2.944
2.374	4.484	2.765
3.165	2.066	3.133
4.703	1.802	2.913
5.231	1.099	2.037
2.681	4.088	3.085
5.451	1.670	2.460
3.956	5.231	2.529
1.846	4.440	2.456
4.923	1.363	2.501
2.066	2.945	2.962
1.670	4.132	2.487
2.462	1.758	2.735
4.308	5.802	1.662
5.538	1.099	1.777
1.714	3.648	2.691
1.934	2.593	2.818
2.462	5.758	1.274
4.352	0.879	2.173
1.890	1.758	2.378
1.670	2.901	2.690
3.912	5.890	1.561
3.407	5.465	2.238

1.978	4.967	2.119
2.066	1.978	2.649
2.549	1.275	2.381
3.473	0.747	2.044
1.802	3.077	2.803
2.110	2.198	2.788
1.538	3.385	2.590
3.077	0.923	2.197
2.066	5.275	1.812
1.319	2.549	2.287
2.374	5.187	2.168
1.407	4.308	2.132
1.538	2.198	2.354
1.978	0.967	1.529

1.011	3.736	1.933
0.923	3.297	1.902
0.923	2.505	1.749
1.055	4.176	1.764
1.231	4.659	1.589
0.879	2.857	1.803
2.586	2.806	3.189
2.945	1.839	2.966
2.813	1.597	2.769
5.538	4.337	2.635
5.971	4.777	1.921
5.861	5.055	1.715
3.121	4.110	3.199
4.681	5.253	2.343

4.960	5.634	1.649
6.337	3.443	2.256
6.073	2.337	2.359
4.015	1.319	2.705
3.538	1.311	2.698
3.744	0.938	2.323
4.315	1.172	2.522
1.377	3.692	2.384
1.231	3.048	2.296
1.377	2.037	2.089
4.535	3.055	3.340
3.810	2.667	3.380
3.421	5.817	1.681

HOM-1-4

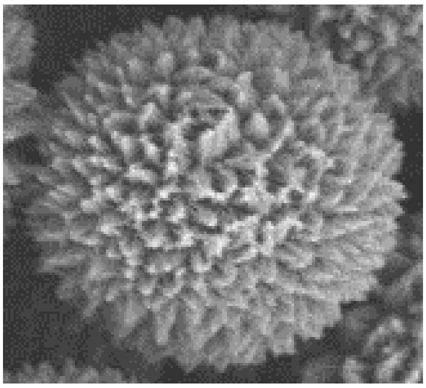

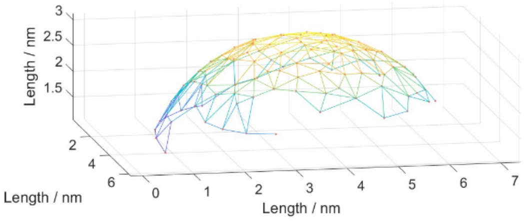

Average length = 0.558±0.154 μm

Modelling function: $\frac{(x-a)^2}{A^2}+\frac{(y-b)^2}{B^2}+\frac{z^2}{C^2}=1$ (a=3.72; b=3.21; A=C=3.12; B=3.01)

x	y	z
3.341	4.484	2.805
4.264	4.176	2.908
3.692	4.088	2.988
3.121	4.044	2.942
2.593	4.044	2.783
3.956	3.780	3.059
3.560	3.736	3.072
4.835	3.692	2.875
2.286	3.560	2.753
2.989	3.560	3.017
2.549	3.560	2.875
4.132	3.297	3.096
5.231	3.209	2.734
3.648	3.253	3.123
2.725	3.209	2.963
4.308	3.033	3.063
5.890	2.989	2.234
2.198	2.813	2.699
2.857	2.769	2.969
2.857	2.549	2.925
3.516	2.198	2.936
4.527	3.956	2.917
4.791	3.165	2.934
2.505	2.462	2.773
2.945	2.066	2.786
4.659	2.374	2.851
4.403	2.740	3.009
5.275	2.505	2.608
4.220	3.912	2.996
3.824	1.890	2.807
2.769	4.484	2.667
1.802	3.165	2.468
4.835	2.110	2.686
1.714	3.824	2.311
5.846	3.473	2.271
2.066	2.330	2.491
2.989	4.571	2.690
4.396	1.934	2.748
4.571	4.659	2.602
5.319	3.956	2.568
2.198	3.385	2.724
1.758	2.725	2.381
2.154	4.396	2.409
5.978	3.780	2.074
5.495	4.615	2.115
5.670	3.297	2.438
1.538	2.330	2.045
2.505	1.714	2.426
3.297	4.835	2.596
3.736	4.747	2.686
5.055	4.440	2.518
1.890	2.198	2.307
4.044	5.099	2.410
5.407	4.967	1.892
2.418	4.879	2.252
4.220	4.791	2.611
4.044	1.582	2.609
2.681	1.451	2.315
2.110	4.571	2.276
2.505	4.527	2.534
1.582	3.209	2.281
1.538	2.989	2.227
6.330	2.945	1.692
2.813	4.923	2.404
4.879	5.363	1.849
5.714	4.308	2.116
1.802	4.264	2.212
1.451	4.044	1.968
2.110	1.582	2.080
5.978	4.176	1.909
4.615	1.582	2.471
3.165	1.495	2.509
3.736	0.967	2.086
3.604	5.495	2.030
4.352	5.231	2.227
3.209	5.099	2.379
5.319	1.934	2.334
1.758	1.978	2.072
5.495	1.626	1.976
3.253	0.835	1.865
1.231	2.681	1.811
2.813	0.879	1.760
1.538	4.571	1.736
4.659	1.099	2.020
4.938	4.791	2.362
1.055	3.121	1.633
1.099	2.198	1.344
3.473	1.143	2.260
1.055	3.604	1.583

0.835	3.209	1.206
1.802	4.967	1.663
5.099	1.670	2.303
2.066	1.407	1.881
2.154	0.967	1.380
1.670	1.363	1.379
2.725	5.582	1.647

2.110	4.791	2.117
0.923	2.637	1.265
0.879	3.956	1.051
2.198	5.319	1.631
1.275	1.846	1.340
1.495	1.978	1.786
1.978	1.099	1.394

4.176	2.520	3.007
3.839	2.872	3.103
3.311	2.996	3.090
3.949	4.557	2.784
3.773	5.209	2.335
5.619	2.769	2.437
4.212	1.209	2.283

HOM-1-5

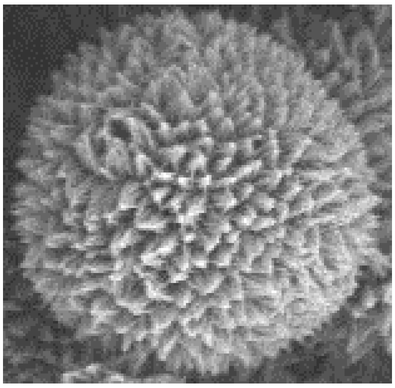

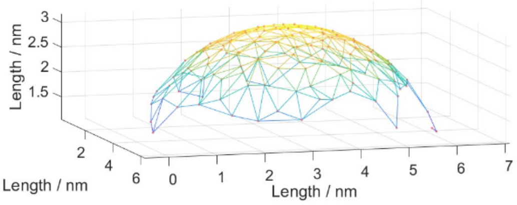

Average length = 0.532±0.160 μm

Modelling function: $\frac{(x-a)^2}{A^2}+\frac{(y-b)^2}{B^2}+\frac{z^2}{C^2}=1$ (a=3.31; b=3.31; A=C=3.18; B=3.16)

x	y	z
4.527	5.538	1.899
3.165	5.538	2.252
3.868	5.275	2.429
2.637	5.055	2.568
4.747	5.011	2.264
3.033	4.791	2.799
1.978	4.791	2.478
3.165	4.571	2.915
3.473	4.527	2.933
2.813	4.264	2.994
2.066	4.176	2.798
3.209	4.088	3.084
3.912	3.912	3.066
2.593	3.868	3.051
4.220	3.780	3.013
3.253	3.648	3.164
2.066	3.516	2.923
4.000	3.473	3.103
2.681	3.341	3.121
5.143	3.209	2.599
3.121	3.077	3.169
4.000	3.033	3.094
3.560	3.033	3.161
2.813	2.989	3.127
4.264	2.857	3.001
5.231	4.308	2.329
3.033	3.516	3.164
3.297	5.758	2.012
4.132	5.099	2.491

3.604	4.220	3.034
2.418	2.637	2.979
2.813	2.110	2.902
3.824	4.527	2.892
6.154	3.648	1.385
4.659	4.571	2.587
3.429	2.681	3.117
3.560	2.330	3.015
3.165	5.187	2.557
1.978	4.615	2.576
4.044	4.044	3.007
1.846	3.692	2.801
3.560	5.758	1.996
4.835	2.945	2.768
3.077	2.505	3.069
2.857	5.582	2.166
4.571	3.824	2.876
5.978	4.571	1.179
5.714	3.692	2.048
4.659	3.473	2.878
4.000	2.462	2.987
4.923	3.604	2.727
5.363	3.516	2.422
5.846	3.253	1.920
3.560	5.011	2.671
5.319	2.374	2.279
4.879	4.000	2.680
5.670	2.989	2.109
2.857	5.978	1.644

2.418	5.758	1.805
4.264	4.615	2.737
6.198	4.132	1.047
3.912	2.725	3.069
4.747	2.549	2.733
4.308	3.209	3.020
2.242	2.066	2.724
1.582	5.363	1.696
2.286	5.275	2.274
3.516	2.154	2.954
1.451	4.967	1.974
1.275	4.220	2.272
5.890	2.593	1.715
2.022	5.714	1.616
4.923	5.670	1.368
3.648	1.538	2.611
2.330	4.659	2.707
1.582	3.692	2.647
1.582	4.615	2.329
1.495	3.912	2.545
4.440	2.022	2.676
4.308	2.462	2.898
3.209	2.066	2.923
3.165	1.538	2.630
0.791	4.176	1.743
1.714	2.901	2.724
1.978	1.934	2.537
2.593	1.846	2.727
3.956	2.066	2.852
5.363	1.890	1.963

1.231	3.429	2.409
1.934	2.593	2.778
3.736	1.802	2.763
1.758	2.242	2.563
1.099	4.659	1.846
4.484	2.286	2.771
1.451	2.462	2.439
4.527	1.714	2.460
4.923	1.538	2.080
4.923	1.802	2.282
4.220	1.670	2.562
4.000	1.451	2.477
1.231	3.648	2.388
1.407	1.758	2.016
3.253	0.659	1.726
3.736	0.615	1.600

0.615	2.901	1.646
2.505	3.121	3.074
0.396	3.780	1.195
1.495	4.352	2.397
0.879	3.297	2.057
1.670	1.538	2.062
0.703	3.429	1.825
0.747	2.593	1.746
2.286	0.967	1.871
0.923	2.242	1.811
0.440	3.473	1.371
3.509	3.502	3.171
2.462	1.700	2.603
2.637	1.341	2.395
2.894	1.194	2.325
3.465	1.231	2.389

3.875	1.238	2.333
4.388	1.392	2.285
5.355	3.802	2.387
5.216	4.073	2.429
4.864	4.381	2.559
4.491	4.227	2.807
1.993	5.114	2.258
1.707	1.114	1.631
2.330	0.667	1.438
3.377	1.062	2.233
4.212	1.143	2.130
5.487	4.432	2.027
5.158	4.718	2.168
5.121	5.077	1.918
4.095	5.392	2.261
3.985	5.568	2.121

HOM-4-1

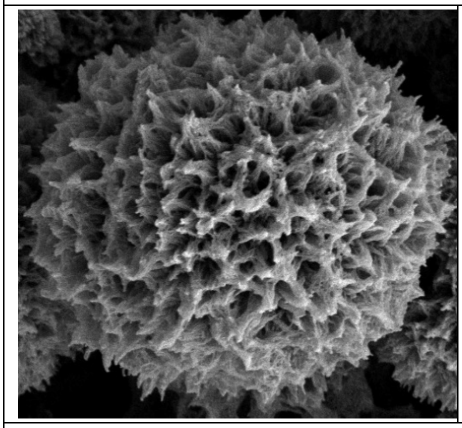

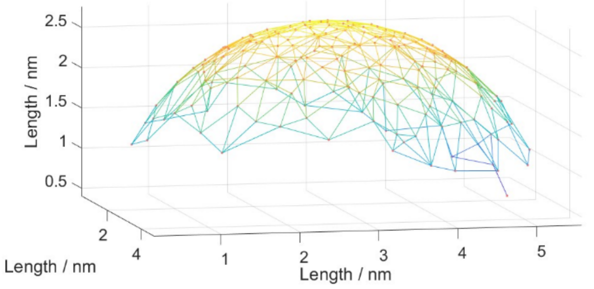

Average length = 0.406±0.157μm

Modelling function: $\frac{(x-a)^2}{A}+\frac{(y-b)^2}{B}+\frac{z^2}{C}=1$ (a=2.84; b=2.59; A=C=2.76; B=2.51)

x	y	z
2.978	4.533	1.732
3.878	4.378	1.625
1.833	4.356	1.674
2.856	4.256	2.057
4.156	4.200	1.648
2.744	4.189	2.118
4.356	4.033	1.663
4.678	3.956	1.395
2.211	3.978	2.205
3.889	3.956	2.056
3.211	3.856	2.348
3.711	3.778	2.263
2.678	3.767	2.426
2.322	3.656	2.439
2.878	3.556	2.542
4.678	3.578	1.739
4.144	3.467	2.226
4.556	3.533	1.888
3.478	3.444	2.510
2.967	3.478	2.573
4.900	3.467	1.552
3.233	3.411	2.573
2.211	3.333	2.555
2.767	3.311	2.638
4.544	3.289	2.023
3.344	3.222	2.618
2.044	3.233	2.542
5.233	3.222	1.172
2.189	3.156	2.604
2.667	3.167	2.676
2.522	3.156	2.665
3.956	3.122	2.449
4.267	3.111	2.285
4.211	3.033	2.339
3.000	2.933	2.725
4.222	2.800	2.372
3.200	2.900	2.710
1.844	2.900	2.547
3.489	2.867	2.660
2.344	2.878	2.692
4.533	2.789	2.162
2.133	2.856	2.647
4.989	2.689	1.719
2.500	2.711	2.731
1.800	2.756	2.546
3.411	2.733	2.691
2.122	2.733	2.656
3.778	2.656	2.589
2.733	2.578	2.754
3.078	2.544	2.745
1.878	2.544	2.582
5.200	2.322	1.389
5.000	2.256	1.669
3.444	2.233	2.660
4.044	2.056	2.408
4.844	2.200	1.841
2.156	2.089	2.613
3.150	1.944	2.645
2.944	1.956	2.664
3.278	1.811	2.582
3.967	1.744	2.336
3.233	1.656	2.527
4.433	1.444	1.862
2.222	1.478	2.392
4.433	1.200	1.649
3.633	4.067	2.078
3.133	1.656	2.541
3.322	4.344	1.906
3.000	1.811	2.615
4.478	2.367	2.201
3.333	4.656	1.476
3.022	4.300	2.004
4.578	1.944	2.016
1.844	2.100	2.513
4.656	1.367	1.576
3.256	4.611	1.572
2.667	3.533	2.546
2.822	0.989	2.121
4.011	2.556	2.493
5.156	1.433	0.779
4.744	0.944	0.831
3.689	2.989	2.584
3.922	1.633	2.305
4.611	4.256	1.043
5.122	3.633	1.029
1.967	2.022	2.539
2.733	3.967	2.299
3.778	3.678	2.297
4.878	2.933	1.814

5.089	1.622	1.183
3.144	4.078	2.195
3.878	0.911	1.763
3.667	4.722	1.184
3.178	4.733	1.384
2.633	2.378	2.738
3.800	1.900	2.469
3.800	1.711	2.395
2.333	2.633	2.708
2.289	1.578	2.461
3.878	1.589	2.303
2.967	1.100	2.213
3.500	4.578	1.539
2.156	4.400	1.777
1.478	3.478	2.187
3.011	1.189	2.279
4.589	1.011	1.232
3.678	1.011	1.969
2.389	4.633	1.528
3.489	1.333	2.294
2.178	3.656	2.404
3.989	4.633	1.102
3.678	4.344	1.778
1.911	2.233	2.565
3.322	1.067	2.135
5.311	1.544	0.400
1.700	1.189	1.982
3.689	0.556	1.369
3.756	4.233	1.866
4.089	1.111	1.841
3.756	2.156	2.554

1.611	1.522	2.170
3.622	1.656	2.435
1.678	1.056	1.845
2.522	1.033	2.137
1.567	4.167	1.721
1.778	3.778	2.181
2.956	1.400	2.423
0.956	3.756	1.549
3.467	1.711	2.503
4.989	1.333	1.030
3.989	0.556	1.128
4.133	2.633	2.432
2.022	1.667	2.429
0.411	2.222	1.240
2.900	2.189	2.720
3.311	0.622	1.641
1.144	4.144	1.340
0.722	2.222	1.718
1.256	1.933	2.138
3.467	0.711	1.713
2.589	0.622	1.689
1.567	2.578	2.445
1.589	2.256	2.429
2.533	2.878	2.720
4.156	1.489	2.096
1.600	2.089	2.399
4.289	2.000	2.252
1.611	1.922	2.356
2.722	0.867	1.999
1.244	2.533	2.247
4.633	2.411	2.082

1.689	0.733	1.452
0.600	2.633	1.606
1.978	3.467	2.433
1.578	2.444	2.445
2.700	1.833	2.624
0.689	1.733	1.445
0.767	3.111	1.724
1.456	2.778	2.375
1.667	2.956	2.461
2.611	2.078	2.688
2.100	1.044	2.040
1.900	0.911	1.819
2.978	0.467	1.459
1.533	1.122	1.813
1.044	1.189	1.416
0.467	2.878	1.367
2.472	3.867	2.341
1.322	2.328	2.283
0.989	2.228	2.004
1.044	1.767	1.886
1.406	1.517	2.038
1.178	3.317	2.048
1.161	3.094	2.114
1.194	2.844	2.193
3.278	1.939	2.625
4.350	1.767	2.119
2.033	4.094	2.050
1.400	3.867	1.883
0.939	3.306	1.834

HOM-4-2

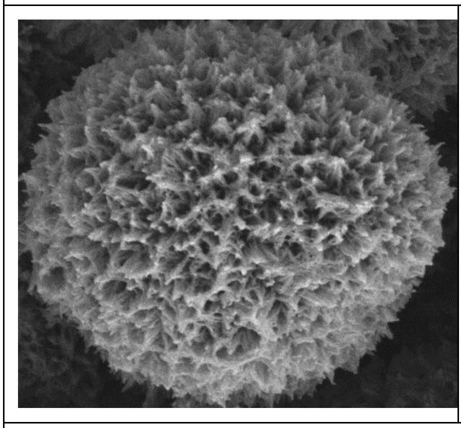

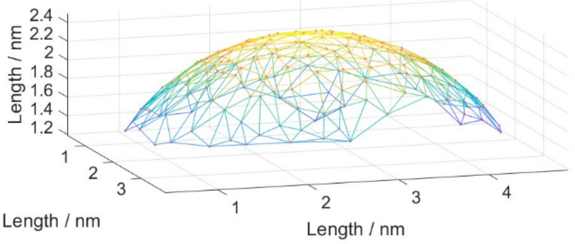

Average length = 0.324±0.112μm

Modelling function: $\frac{(x-a)^2}{A} + \frac{(y-b)^2}{B} + \frac{z^2}{C} = 1$ (a=2.55; b=2.22; A=C=2.49; B=2.20)

x	y	z
2.756	3.522	1.991
1.844	3.756	1.629
2.333	3.133	2.250
3.233	3.356	2.015
3.489	3.300	1.951
2.822	3.300	2.147
1.822	3.233	2.080
3.350	3.044	2.161
4.033	3.044	1.765
3.189	2.956	2.254
1.556	2.911	2.138
2.922	2.933	2.322
2.789	2.822	2.379
2.322	2.833	2.375
3.956	2.800	1.944
2.556	2.744	2.414
3.522	2.667	2.232
2.389	2.667	2.428
2.056	2.567	2.404
1.456	2.578	2.193
3.733	2.522	2.161
3.289	2.567	2.341
1.300	2.567	2.110
3.544	2.533	2.252
4.356	2.522	1.677
4.056	2.500	1.954
3.011	2.456	2.429
2.300	2.478	2.455
3.811	2.433	2.130
2.611	2.411	2.476
3.489	2.411	2.293
2.889	2.411	2.453
3.278	2.367	2.372
1.344	2.378	2.165
1.889	2.356	2.391
4.344	2.333	1.719
4.011	2.178	2.013
2.100	2.211	2.444
2.478	2.178	2.485
3.811	2.156	2.143
1.689	2.133	2.329
2.833	2.067	2.464
1.689	1.911	2.305
2.189	1.889	2.431
3.456	1.867	2.282
1.956	1.822	2.372
2.311	1.789	2.427
4.211	1.767	1.781
2.533	1.733	2.425
3.711	1.656	2.107
3.356	1.644	2.262
2.644	1.633	2.395
1.489	1.500	2.096
2.744	1.233	2.215
1.411	3.567	1.597
1.278	3.439	1.627
1.978	3.700	1.744
3.811	3.100	1.898
1.811	3.000	2.202
0.967	3.044	1.671
3.056	2.311	2.432
3.522	1.611	2.185
1.078	3.333	1.554
3.528	3.028	2.095
0.844	2.522	1.772
3.178	2.078	2.401
2.944	0.822	1.882
0.656	2.744	1.492
4.289	2.711	1.690
1.633	2.678	2.250
1.144	2.067	2.041
2.522	3.411	2.088
3.756	2.956	2.009
0.778	2.183	1.740
2.322	1.622	2.382
2.378	3.772	1.750
1.678	3.211	2.038
1.056	1.717	1.903
1.644	1.422	2.132
2.444	3.589	1.940
1.311	3.122	1.895
0.778	2.928	1.544
1.578	2.400	2.278
3.067	2.122	2.430
1.422	3.356	1.801
2.944	3.078	2.254
3.144	1.133	2.081
2.078	1.511	2.306
1.100	1.922	1.989

3.689	3.278	1.858
3.833	1.244	1.826
1.589	3.156	2.031
1.411	1.389	2.000
2.056	2.967	2.284
2.767	2.233	2.477
4.289	2.156	1.778
2.467	3.878	1.628
1.778	3.589	1.782
3.367	2.889	2.223
2.911	1.600	2.359
2.000	1.456	2.266
4.456	1.533	1.401
0.933	2.300	1.883
3.989	1.933	2.004
2.344	1.222	2.207
2.022	2.744	2.355
4.600	2.311	1.407
1.822	2.644	2.327
0.789	1.978	1.731
2.844	1.944	2.450
4.567	1.811	1.383
3.056	1.000	2.009
0.633	2.278	1.578
1.422	2.100	2.210
4.067	1.611	1.849
1.544	2.756	2.189
2.767	1.889	2.449
3.178	1.589	2.299
1.133	2.322	2.037

2.067	1.333	2.224
2.189	3.822	1.661
2.667	2.011	2.473
2.256	3.622	1.890
4.006	1.439	1.816
1.556	3.567	1.691
4.667	2.156	1.306
1.722	1.567	2.225
3.222	1.300	2.158
4.478	1.967	1.548
2.022	1.722	2.363
1.878	1.211	2.105
4.222	1.283	1.510
2.756	0.911	1.990
3.556	0.911	1.731
4.333	2.022	1.721
4.389	1.711	1.576
1.622	0.778	1.634
1.111	2.567	1.986
1.289	1.167	1.782
2.622	2.922	2.354
0.789	1.256	1.375
4.022	1.189	1.635
1.856	0.722	1.685
4.467	1.367	1.263
2.478	0.633	1.724
1.744	0.544	1.397
2.300	0.967	2.030
3.100	1.922	2.402
1.922	0.900	1.889

2.567	1.500	2.350
1.000	1.278	1.626
1.311	0.789	1.426
0.811	1.744	1.691
1.578	1.011	1.836
2.311	0.578	1.641
1.478	1.756	2.180
3.478	1.444	2.136
1.522	2.322	2.259
2.500	0.989	2.062
1.944	0.556	1.512
2.078	2.011	2.429
2.622	0.656	1.750
3.244	0.600	1.538
1.467	1.656	2.145
3.167	0.367	1.197
0.678	1.878	1.587
4.289	1.444	1.550
3.433	0.556	1.372
2.122	0.922	1.963
3.278	0.856	1.814
1.833	1.056	1.985
2.750	0.456	1.477
2.950	3.644	1.850
1.244	2.911	1.963
2.617	3.133	2.259
2.006	3.283	2.105
2.006	3.500	1.944
1.117	2.783	1.926
3.578	2.128	2.263

HOM-4-3

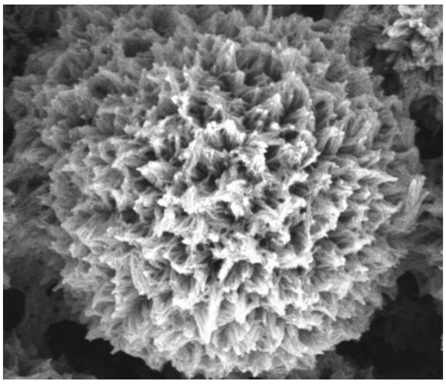

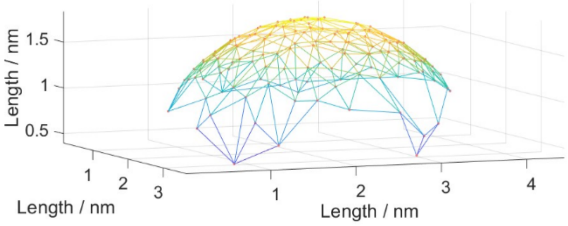

Average length = 0.319±0.118μm

Modelling function: $\frac{(x-a)^2}{A}+\frac{(y-b)^2}{B}+\frac{z^2}{C}=1$ (a=2.26; b=1.85; A=C=1.84; B=1.73)

x	y	z
2.007	2.948	1.396
2.933	3.333	0.661
2.667	3.296	0.921
2.594	2.475	1.677
1.526	3.074	1.073
1.111	2.948	0.838
1.333	2.785	1.238
3.170	2.719	1.297
2.244	2.467	1.714
2.341	2.622	1.640
1.378	2.593	1.406
3.526	2.563	1.087
2.889	2.459	1.596
3.244	2.489	1.389
1.037	2.504	1.184
1.422	2.415	1.521
3.444	2.326	1.303
1.867	2.252	1.742
2.673	2.026	1.777
2.044	2.259	1.770
1.548	2.156	1.661
3.244	1.993	1.538
1.993	1.911	1.815
1.319	1.896	1.577
3.548	1.830	1.302
2.837	1.681	1.731
0.896	1.756	1.228
2.481	1.474	1.776
3.119	1.667	1.607
1.674	1.659	1.728
1.444	1.563	1.617
3.533	1.615	1.293
2.993	1.504	1.639
2.904	1.363	1.636
2.052	1.222	1.697
1.489	1.326	1.571
2.437	1.252	1.712
3.200	1.222	1.425
2.911	1.222	1.579
2.637	1.163	1.640
1.541	1.148	1.517
1.733	0.919	1.455
2.956	1.052	1.469
2.481	1.015	1.591
2.096	0.978	1.576
2.667	0.896	1.474
1.600	0.719	1.224
2.815	0.667	1.216
2.215	0.422	1.035
1.022	2.178	1.313
1.119	1.874	1.440
1.074	1.563	1.370
3.281	1.244	1.379
1.778	0.674	1.258
2.741	0.467	0.989
3.467	2.067	1.358
1.814	1.389	1.712
3.281	1.719	1.515
2.800	1.185	1.604
2.067	3.304	0.978
2.393	1.807	1.829
0.837	1.444	1.081
1.378	1.163	1.437
2.274	1.422	1.777
3.474	1.304	1.243
1.763	0.607	1.177
3.793	1.985	0.991
2.800	3.444	0.464
0.815	2.259	1.050
3.170	3.163	0.770
1.615	1.267	1.604
1.981	3.122	1.214
1.533	2.748	1.392
3.630	1.489	1.153
1.059	1.326	1.275
3.296	0.948	1.169
2.970	0.926	1.376
0.785	2.459	0.887
3.452	1.763	1.388
1.422	0.704	1.093
2.674	0.378	0.868
2.526	3.059	1.285
2.956	2.689	1.444
1.281	3.200	0.608
2.880	1.927	1.723
3.533	1.133	1.075
3.384	1.502	1.398
0.889	1.585	1.191
2.615	0.452	1.019

3.215	1.556	1.532
2.496	1.141	1.656
3.644	1.852	1.199
0.659	1.563	0.851
3.156	0.859	1.204
2.230	1.644	1.821
0.667	2.363	0.740
2.459	0.807	1.449
2.311	0.659	1.329
2.296	0.919	1.546
1.519	0.874	1.323
2.785	2.681	1.520
3.067	0.630	1.016
0.874	2.926	0.396
0.933	1.363	1.162
1.415	1.037	1.384

2.459	0.385	0.954
1.809	1.102	1.593
1.763	0.422	0.911
3.207	1.067	1.331
2.001	1.428	1.761
1.296	0.919	1.212
2.044	0.600	1.250
2.000	0.733	1.377
2.144	1.448	1.781
2.293	3.043	1.329
2.214	1.764	1.832
1.542	1.898	1.689
1.784	1.907	1.772
1.690	2.535	1.587
1.932	2.604	1.620
1.764	2.860	1.407

2.135	2.599	1.650
2.219	2.796	1.536
2.520	2.786	1.521
2.421	2.431	1.720
2.732	2.253	1.719
2.243	1.981	1.829
3.083	2.238	1.585
3.601	2.184	1.196
3.730	1.695	1.079
2.174	1.157	1.679
1.098	2.707	1.096
1.221	2.233	1.460
1.749	3.167	1.078
1.211	1.226	1.355

HOM-4-4

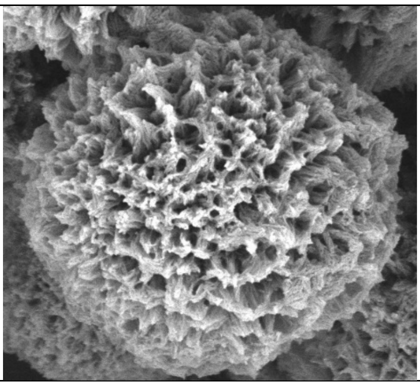

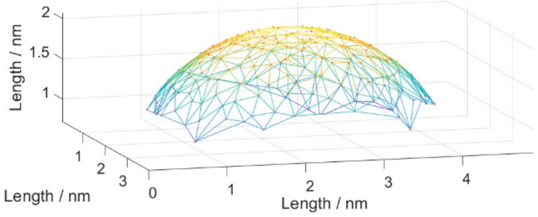

Average length = 0.296±0.112μm

Modelling function: $\frac{(x-a)^2}{A}+\frac{(y-b)^2}{B}+\frac{z^2}{C}=1$ (a=2.35; b=2.12; A=C=2.09; B=2.01)

x	y	z
1.932	3.676	1.257
2.032	3.368	1.608
1.716	3.572	1.300
2.600	3.420	1.575
3.456	3.528	1.002
1.352	3.472	1.182
3.080	3.440	1.398
3.312	3.416	1.276
1.576	3.336	1.474
2.468	3.032	1.859
2.976	3.008	1.767
1.824	3.008	1.800
3.400	3.064	1.517
2.016	2.992	1.853
0.968	2.912	1.334
2.104	2.752	1.968
3.120	2.800	1.810
2.800	2.720	1.943
1.208	2.792	1.605
1.632	2.736	1.855
1.360	2.764	1.714
0.896	2.752	1.350
3.144	2.640	1.856
2.480	2.696	1.998
1.824	2.664	1.942
1.008	2.656	1.502
0.904	2.600	1.424
2.336	2.624	2.023
2.440	2.608	2.025
1.352	2.432	1.807
2.680	2.528	2.019
2.040	2.488	2.031
1.712	2.440	1.962
1.016	2.496	1.560
2.280	2.320	2.078
1.104	2.456	1.641
3.688	2.432	1.572
3.528	2.384	1.704
0.992	2.328	1.574
0.608	2.376	1.123
3.280	2.320	1.860
3.032	2.304	1.966
0.808	2.296	1.399
1.592	2.240	1.944
2.904	2.272	2.009
1.688	2.224	1.979
1.432	2.144	1.877
2.048	2.128	2.068
4.080	2.184	1.171
3.336	2.144	1.843
2.632	2.072	2.070
0.592	2.136	1.130
2.472	2.120	2.086
1.296	2.096	1.805
1.880	2.064	2.036
1.072	2.016	1.650
0.656	2.016	1.220
3.168	2.032	1.921
2.336	2.024	2.088
1.744	2.016	1.997
2.464	2.040	2.085
0.992	1.920	1.575
1.568	1.880	1.922
2.904	1.840	1.995
1.328	1.840	1.800
2.200	1.792	2.057
2.560	1.832	2.058
3.304	1.808	1.832
1.432	1.824	1.853
1.152	1.792	1.679
1.936	1.800	2.022
3.384	1.760	1.778
1.936	1.624	1.983
3.128	1.584	1.859
2.672	1.656	2.009
1.784	1.688	1.962
2.328	1.576	2.013
3.600	1.600	1.586
2.776	1.480	1.936
0.936	1.552	1.423
3.816	1.520	1.354
1.496	1.432	1.770
1.840	1.176	1.776
3.240	1.320	1.700
1.424	1.224	1.628
2.584	1.272	1.882
3.376	1.256	1.586
3.048	1.208	1.729
2.392	1.176	1.847
2.808	1.080	1.732
2.496	0.992	1.727
2.200	0.912	1.667

2.672	0.920	1.649
1.648	0.688	1.294
3.408	3.176	1.429
3.208	3.232	1.515
0.896	3.056	1.143
1.168	3.312	1.198
3.608	3.128	1.299
0.760	2.912	1.078
2.344	1.840	2.070
1.552	3.648	1.100
3.224	3.096	1.604
2.832	2.000	2.030
1.472	1.976	1.891
0.864	1.792	1.430
0.856	1.488	1.307
1.304	3.224	1.399
3.980	2.680	1.171
1.384	1.440	1.715
3.784	2.912	1.278
1.136	2.976	1.450
1.080	1.512	1.536
3.836	2.256	1.463
3.320	3.512	1.155
1.376	0.944	1.391
2.856	0.824	1.520
3.952	2.160	1.342
2.696	1.800	2.035
0.816	3.168	0.910
1.872	2.528	1.990
1.160	2.160	1.718
1.800	1.384	1.867
1.168	3.160	1.342
4.040	2.560	1.141
3.016	2.416	1.957
2.992	0.920	1.553
1.448	3.480	1.248
1.128	2.680	1.592

1.648	2.096	1.968
3.784	1.360	1.301
2.816	0.912	1.608
1.720	3.272	1.593
2.064	1.624	2.006
2.920	0.688	1.357
2.896	1.600	1.945
2.104	0.648	1.407
2.768	1.856	2.030
1.624	1.728	1.918
2.616	0.664	1.422
4.144	1.824	1.028
2.704	0.792	1.533
2.928	1.336	1.837
0.992	1.264	1.319
3.372	1.136	1.512
2.288	1.120	1.814
4.088	1.972	1.151
3.472	1.960	1.756
3.676	1.892	1.598
1.488	1.592	1.824
3.552	0.976	1.232
0.984	0.856	0.887
1.296	2.000	1.801
3.808	1.760	1.451
3.028	1.728	1.935
3.656	1.112	1.254
2.992	3.288	1.575
3.872	2.056	1.431
2.648	0.408	1.063
0.728	1.320	1.025
3.088	0.920	1.509
3.400	0.904	1.296
2.240	1.176	1.844
3.368	0.736	1.129
3.544	0.720	0.915
3.656	2.200	1.630

3.736	0.904	0.927
0.696	1.816	1.239
3.928	1.072	0.837
1.280	0.768	1.123
2.456	0.832	1.605
3.192	0.400	0.693
1.684	0.956	1.572
3.056	0.376	0.776
1.256	1.116	1.446
2.560	2.312	2.070
2.380	2.264	2.084
2.432	2.404	2.067
2.184	2.432	2.058
3.400	2.652	1.720
1.976	3.240	1.695
2.384	3.312	1.683
2.796	3.068	1.788
3.936	1.908	1.344
3.952	1.428	1.135
2.300	2.832	1.954
1.636	2.928	1.775
0.844	2.076	1.449
1.416	3.072	1.586
1.616	3.104	1.668
1.916	3.452	1.504
2.216	3.520	1.495
2.400	3.572	1.445
2.704	3.672	1.282
2.576	3.112	1.804
3.064	0.648	1.237
2.864	0.516	1.158
2.772	0.364	0.936
2.244	0.432	1.138
1.132	1.000	1.240
0.748	1.636	1.246
0.720	2.600	1.209
2.796	3.492	1.462

HOM-4-5

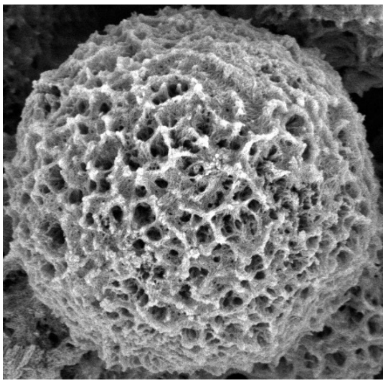

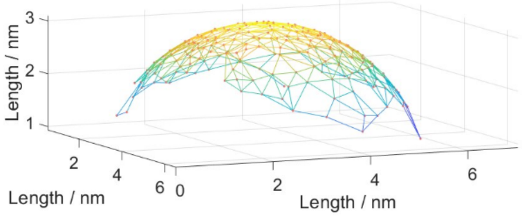

Average length = 0.414±0.135μm

Modelling function: $\frac{(x-a)^2}{A}+\frac{(y-b)^2}{B}+\frac{z^2}{C}=1$ (a=3.25; b=3.21; A=C=3.10; B=3.10)

x	y	z
2.622	5.522	1.968
3.211	5.356	2.238
3.067	5.156	2.408
2.611	5.089	2.384
3.478	5.056	2.482
4.567	5.011	2.153
4.278	5.000	2.314
4.056	4.911	2.465
4.600	4.756	2.324
4.078	4.667	2.610
3.244	4.300	2.906
3.024	4.520	2.804
2.200	4.511	2.615
4.511	4.478	2.534
2.656	4.356	2.823
2.222	4.300	2.719
5.267	4.278	2.099
4.867	4.178	2.463
2.489	4.156	2.857
2.772	4.098	2.936
3.469	3.639	3.066
2.733	3.711	3.020
2.378	3.844	2.912
2.144	3.878	2.823
3.180	3.772	3.052
4.589	3.733	2.749
4.778	3.700	2.655
2.089	3.667	2.843
1.411	3.344	2.499
4.456	3.333	2.856
4.928	3.283	2.608
1.711	3.400	2.691
2.044	3.367	2.857
3.289	3.311	3.102
1.878	3.133	2.784
3.478	3.167	3.095
3.944	3.111	3.022
2.333	2.889	2.948
3.922	2.856	3.008
5.400	2.978	2.222
1.522	2.867	2.557
3.378	2.944	3.089
1.844	2.933	2.754
4.767	2.878	2.685
2.122	2.900	2.876
3.511	2.867	3.073
3.011	2.878	3.076
3.009	2.587	3.030
1.967	2.678	2.776
0.967	2.689	2.039
3.639	2.624	3.021
5.111	2.644	2.414
4.522	2.300	2.677
5.778	2.611	1.691
3.787	2.483	2.967
5.522	2.456	1.969
0.589	2.433	1.399
1.533	2.444	2.470
3.422	2.333	2.970
2.289	2.344	2.820
1.800	2.156	2.533
3.967	2.078	2.796
2.289	2.067	2.719
1.167	1.956	1.928
5.444	2.033	1.844
4.067	1.733	2.599
4.667	1.689	2.297
5.089	1.800	2.056
2.478	1.711	2.602
2.711	1.767	2.691
5.144	1.656	1.895
3.000	1.556	2.609
1.609	1.543	2.035
4.311	1.356	2.242
3.000	1.367	2.478
2.733	1.367	2.437
1.456	1.222	1.560
1.644	1.200	1.727
3.756	0.978	2.084
1.978	1.022	1.786
2.467	0.933	1.947
3.200	0.800	1.941
3.656	5.600	1.931
4.133	5.389	2.020
3.756	5.278	2.254
2.411	4.311	2.779
2.067	4.178	2.702
5.144	4.000	2.325
1.656	3.856	2.586

1.489	3.800	2.489
1.356	3.644	2.423
4.267	3.544	2.912
2.678	3.411	3.045
2.900	3.211	3.084
0.556	2.967	1.527
4.378	2.500	2.800
4.167	0.967	1.925
4.678	0.956	1.567
5.478	4.667	1.589
3.511	4.244	2.914
1.878	3.867	2.707
1.056	3.667	2.151
3.367	1.756	2.735
2.933	0.778	1.888
4.789	4.967	2.038
4.111	4.389	2.737
1.356	3.944	2.349
4.244	5.678	1.587
5.056	4.789	1.964
5.244	4.756	1.801
3.506	4.698	2.710
4.756	3.978	2.601
3.389	5.844	1.626
4.567	5.544	1.555
1.978	4.867	2.295
3.744	1.644	2.628
1.061	3.424	2.193
4.756	2.544	2.627
4.111	5.878	1.317
3.631	4.483	2.804
2.811	5.378	2.173
4.822	4.522	2.329
5.011	3.844	2.473

5.700	2.922	1.878
3.033	0.978	2.135
5.306	3.535	2.299
5.411	3.422	2.214
2.189	3.411	2.911
4.900	5.233	1.670
4.400	3.889	2.800
5.261	2.187	2.125
2.756	5.711	1.763
5.044	4.222	2.319
5.711	3.711	1.819
1.289	2.878	2.385
5.867	3.933	1.498
1.261	2.313	2.208
4.278	1.989	2.657
3.300	1.056	2.224
1.189	2.622	2.247
2.928	4.713	2.695
5.678	4.011	1.755
5.900	4.522	0.930
1.656	1.878	2.304
2.400	0.789	1.732
5.033	3.567	2.513
3.433	1.156	2.310
3.750	4.276	2.871
5.311	3.233	2.317
3.372	2.646	3.048
4.667	3.144	2.759
3.031	3.935	3.010
3.091	4.209	2.934
3.239	3.550	3.085
3.698	3.543	3.053
3.639	3.898	3.001
3.869	3.306	3.039

3.943	3.498	3.011
3.943	3.735	2.979
4.039	3.957	2.906
4.165	3.794	2.907
4.276	3.261	2.927
2.750	2.180	2.883
2.513	2.639	2.960
2.017	2.380	2.724
3.780	4.898	2.547
4.150	5.157	2.238
2.543	1.283	2.321
2.446	1.417	2.397
2.646	1.550	2.547
1.965	1.594	2.313
2.024	1.876	2.517
3.106	1.987	2.846
1.580	3.594	2.590
1.861	3.594	2.751
2.513	3.246	3.015
2.572	2.928	3.016
4.794	2.098	2.447
4.994	2.372	2.422
5.787	4.172	1.501
5.624	4.380	1.615
5.535	4.224	1.835
5.402	3.920	2.117
4.209	2.320	2.811
1.787	4.743	2.268
2.002	4.639	2.457
2.231	5.046	2.284
1.965	5.098	2.101
2.291	5.328	2.053
1.498	2.113	2.314
1.106	3.039	2.240

HOM-5-1

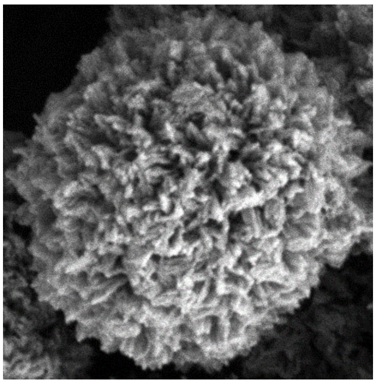

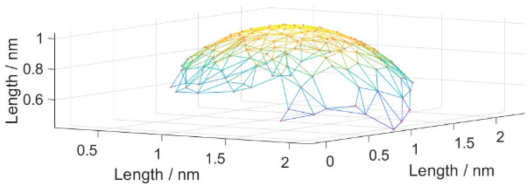

Average length = 0.155±0.052μm

Modelling function: $\frac{(x-a)^2}{A}+\frac{(y-b)^2}{B}+\frac{z^2}{C}=1$ (a=1.12; b=1.12; A=C=1.04; B=1.06)

x	y	z
1.344	1.922	0.632
0.967	1.917	0.660
1.422	1.833	0.699
1.111	1.811	0.781
1.444	1.728	0.779
1.317	1.706	0.836
1.106	1.661	0.887
1.500	1.667	0.797
1.622	1.594	0.774
1.428	1.606	0.864
1.378	1.539	0.913
0.856	1.533	0.915
1.511	1.528	0.869
1.417	1.489	0.922
1.033	1.478	0.969
1.094	1.478	0.972
1.644	1.433	0.836
0.928	1.439	0.967
0.678	1.400	0.895
1.611	1.383	0.872
1.533	1.383	0.912
1.339	1.350	0.985
1.483	1.178	0.967
1.067	1.244	1.026
0.761	1.239	0.964
0.978	1.183	1.023
1.217	1.167	1.029
1.733	1.150	0.832
1.139	1.150	1.034
1.389	1.122	0.999
1.672	1.044	0.871
1.767	1.011	0.800
1.739	0.950	0.812
0.844	0.917	0.979
1.656	0.856	0.847
1.778	0.781	0.727
1.000	0.744	0.962
1.342	0.839	0.973
1.172	1.978	0.599
1.594	0.911	0.897
1.211	0.911	1.011
1.161	1.044	1.032
1.244	1.439	0.978
1.622	1.144	0.904
0.828	1.606	0.871
1.589	1.039	0.919
1.817	0.667	0.624
0.933	2.067	0.416
1.178	2.028	0.523
1.333	1.856	0.709
0.394	1.600	0.569
1.661	0.728	0.795
0.767	1.956	0.524
0.883	1.222	1.003
0.378	1.133	0.723
1.128	1.089	1.035
0.839	1.372	0.965
0.556	1.789	0.568
0.800	1.672	0.822
0.767	1.117	0.974
1.483	1.028	0.965
1.394	0.986	0.989
1.272	1.789	0.784
0.700	1.289	0.932
1.022	1.106	1.031
1.283	0.861	0.991
0.872	1.744	0.796
1.000	1.739	0.829
1.400	0.761	0.933
1.661	0.583	0.710
1.328	1.467	0.954
0.867	0.833	0.965
1.239	0.767	0.969
1.267	1.572	0.922
1.489	1.394	0.927
0.789	0.983	0.973
1.539	0.533	0.754
0.722	1.011	0.951
1.033	2.061	0.459
1.572	1.789	0.658
0.756	1.411	0.926
1.039	0.878	1.005
1.539	0.622	0.813
0.333	1.211	0.668
0.578	0.889	0.854
0.733	1.900	0.580
0.789	0.767	0.920
0.678	0.733	0.858
0.383	1.256	0.716

0.861	1.006	0.997
1.172	0.489	0.832
1.439	0.383	0.674
0.333	1.583	0.497
0.517	1.461	0.772
0.589	1.456	0.825
0.878	1.300	0.991
0.478	1.744	0.534
1.750	0.572	0.623
1.039	0.250	0.589
1.506	0.850	0.923
1.339	0.272	0.584
0.378	1.317	0.697
1.256	0.361	0.712
0.594	1.339	0.866
1.289	0.661	0.918
1.400	0.594	0.855
0.633	1.628	0.765
0.856	0.500	0.800

0.783	0.578	0.826
0.644	0.683	0.817
1.200	0.261	0.604
0.983	1.617	0.902
1.333	0.628	0.893
0.517	0.944	0.826
1.300	0.483	0.809
0.622	1.278	0.895
1.128	0.722	0.960
0.389	0.800	0.666
0.961	0.861	0.992
1.144	1.322	1.015
0.628	1.117	0.912
1.506	0.367	0.619
0.400	0.717	0.635
1.350	0.561	0.850
1.011	0.350	0.706
0.328	0.933	0.644
0.928	0.589	0.877

1.389	0.478	0.780
0.683	0.572	0.774
1.050	0.528	0.858
1.122	0.581	0.893
0.983	1.053	1.024
0.981	1.311	1.008
1.086	1.344	1.010
1.289	1.247	1.013
1.369	1.208	1.000
1.300	0.975	1.009
1.661	1.253	0.871
1.672	1.658	0.696
0.842	1.856	0.687
0.528	1.608	0.702
0.514	1.269	0.827
0.567	0.681	0.766
1.731	1.436	0.773
1.153	1.536	0.950

HOM-5-2

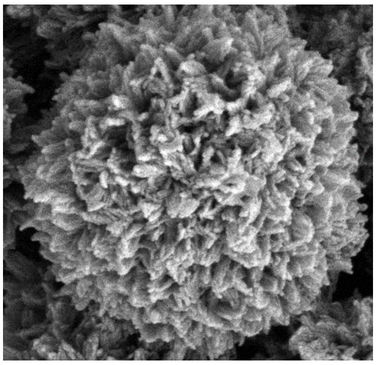

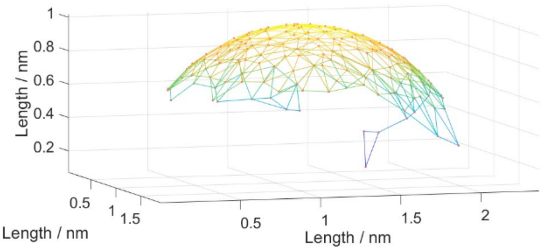

Average length = 0.155±0.052μm

Modelling function: $\frac{(x-a)^2}{A}+\frac{(y-b)^2}{B}+\frac{z^2}{C}=1$ (a=1.15; b=1.06; A=C=1.02; B=1.04)

x	y	z
1.550	1.822	0.564
1.039	1.644	0.835
0.733	1.628	0.744
1.539	1.561	0.802
0.750	1.478	0.842
1.472	1.422	0.898
1.122	1.475	0.933
1.861	1.406	0.643
1.539	1.378	0.887
1.089	1.378	0.967
1.689	1.372	0.807
1.722	1.239	0.823
1.511	1.261	0.931
1.117	1.278	0.995
0.883	1.267	0.961
1.217	1.267	0.995
1.411	1.128	0.981
1.317	1.183	0.997
1.094	1.161	1.012
0.800	1.128	0.954
1.861	1.111	0.726
1.789	1.094	0.791
1.283	1.089	1.009
0.611	1.078	0.864
1.528	1.056	0.945
1.356	1.033	0.996
0.822	1.006	0.963
1.822	1.006	0.762
0.983	0.906	0.993
1.978	0.956	0.582
1.422	0.939	0.974
1.633	0.911	0.884
0.856	0.933	0.967
1.528	0.906	0.933
0.544	0.894	0.803
1.872	0.844	0.685
0.967	0.811	0.972
1.394	0.756	0.943
1.917	0.767	0.604
0.656	0.717	0.826
1.617	0.672	0.821
1.297	0.642	0.921
1.389	0.603	0.883
0.683	0.589	0.780
1.503	0.525	0.799
1.117	0.472	0.841
1.100	0.383	0.773
1.644	0.350	0.558
1.372	0.228	0.572
0.906	1.733	0.737
1.878	0.594	0.547
1.711	0.489	0.641
1.517	0.450	0.740
1.183	0.822	0.991
0.928	1.778	0.702
1.989	0.894	0.552
1.878	0.689	0.612
1.978	0.644	0.430
1.339	1.722	0.761
1.125	1.561	0.891
1.700	1.650	0.631
1.028	1.283	0.987
0.578	1.233	0.825
1.983	1.194	0.569
1.906	0.961	0.674
1.844	0.356	0.286
1.928	1.133	0.651
0.911	1.644	0.808
0.694	1.383	0.854
1.700	1.017	0.855
1.900	1.500	0.535
1.256	1.456	0.935
1.089	0.928	1.008
1.100	0.561	0.893
1.800	1.622	0.556
0.661	1.367	0.841
1.856	0.550	0.538
1.311	1.772	0.724
1.383	1.306	0.961
1.678	1.156	0.865
0.669	0.969	0.893
0.978	1.539	0.887
1.506	1.756	0.666
0.550	1.444	0.732
1.239	1.578	0.878
1.144	0.978	1.015
1.089	1.650	0.836
0.625	1.533	0.739
1.028	0.711	0.952
1.722	1.422	0.763
0.928	1.383	0.942
1.756	0.839	0.789

0.911	0.811	0.960
0.917	0.400	0.754
1.956	1.600	0.324
1.611	1.472	0.812
0.544	0.800	0.779
1.144	1.761	0.752
1.228	1.144	1.012
0.689	1.064	0.908
0.939	1.017	0.995
0.789	0.472	0.760
1.417	0.144	0.407
1.078	1.817	0.694
1.306	0.450	0.811
1.239	0.928	1.006
1.722	0.572	0.694
1.700	1.056	0.856
0.961	0.378	0.747
1.772	0.289	0.287
1.772	1.772	0.402
1.739	1.322	0.789
1.650	1.700	0.626
1.628	1.217	0.885
0.800	0.811	0.925
0.450	1.086	0.740
0.506	0.850	0.762
1.469	0.667	0.887
1.150	0.900	1.006

0.894	0.178	0.480
1.694	0.800	0.822
1.667	0.689	0.798
1.956	0.561	0.386
0.794	1.728	0.694
0.500	1.300	0.748
1.767	1.128	0.806
1.794	0.256	0.071
1.233	0.150	0.491
0.667	1.161	0.891
1.956	1.022	0.620
0.850	0.678	0.899
1.017	0.272	0.654
1.339	0.128	0.416
1.661	1.572	0.723
0.739	1.272	0.908
1.233	1.689	0.806
1.517	1.656	0.748
1.011	0.772	0.969
0.856	0.250	0.571
1.300	1.367	0.961
1.756	0.906	0.804
1.011	0.494	0.844
1.606	1.144	0.906
0.750	1.039	0.936
0.961	0.606	0.897
1.439	0.361	0.698

0.467	0.600	0.609
0.431	0.944	0.713
0.411	0.739	0.628
0.917	0.589	0.879
0.739	0.439	0.708
0.461	1.478	0.628
1.117	0.144	0.486
1.228	0.789	0.980
1.389	1.806	0.667
1.094	1.072	1.016
1.289	0.800	0.976
0.900	1.511	0.882
0.922	0.511	0.836
0.522	0.439	0.526
0.756	1.189	0.930
1.211	0.300	0.695
0.378	0.839	0.629
1.281	0.558	0.883
1.172	0.658	0.939
1.942	1.297	0.594
1.769	1.478	0.696
1.403	1.650	0.798
0.683	0.381	0.617
0.539	0.703	0.737
1.856	1.317	0.687

HOM-5-3

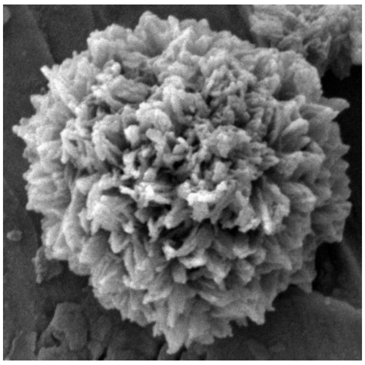

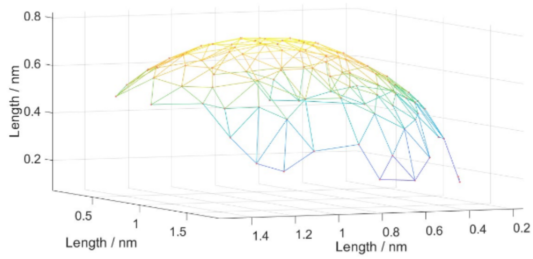

Average length = 0.151±0.050μm

Modelling function: $\frac{(x-a)^2}{A}+\frac{(y-b)^2}{B}+\frac{z^2}{C}=1$ (a=0.892; b=0.936; A=C=0.755; B=0.865)

x	y	z
1.414	1.180	0.502
0.494	1.043	0.635
1.026	1.006	0.741
0.760	0.975	0.743
0.631	0.965	0.708
1.132	0.951	0.716
0.392	0.944	0.566
0.689	0.883	0.726
0.644	0.818	0.706
0.331	0.661	0.445
0.920	0.706	0.727
0.484	0.672	0.592
0.651	0.661	0.674
0.757	0.528	0.652
0.382	0.549	0.443
0.498	0.453	0.487
0.856	0.440	0.618
0.621	0.387	0.517
0.573	0.331	0.435
0.886	0.327	0.536
1.087	0.276	0.447
0.948	0.153	0.316
0.866	1.289	0.689
1.012	1.343	0.655
0.747	0.886	0.740
0.938	0.256	0.464
0.736	0.774	0.725
1.105	0.358	0.520
0.648	1.180	0.682
0.862	1.152	0.730
0.951	0.951	0.753
1.162	0.762	0.689
0.644	0.539	0.623
1.036	0.286	0.477
0.644	1.452	0.553
0.706	1.381	0.620
1.040	0.770	0.726
0.750	1.548	0.514
1.057	0.939	0.737
0.999	0.893	0.746
1.531	0.617	0.290
1.228	0.423	0.507
1.043	0.170	0.317
0.787	0.702	0.719
1.306	1.139	0.606
1.524	0.511	0.182
0.648	1.360	0.611
0.576	1.282	0.616
0.841	1.016	0.750
0.770	1.166	0.718
1.266	0.698	0.622
1.332	0.426	0.422
0.416	0.805	0.575
1.050	0.634	0.690
1.122	0.839	0.714
1.220	0.181	0.168
1.422	0.515	0.393
1.551	0.781	0.343
0.876	0.675	0.720
1.193	0.368	0.483
1.139	1.415	0.578
1.483	1.081	0.452
1.343	0.607	0.533
1.278	1.019	0.645
1.275	0.873	0.648
1.573	0.996	0.322
0.965	0.801	0.742
0.539	0.594	0.597
0.637	0.460	0.577
1.244	1.319	0.578
1.418	0.805	0.529
1.111	0.460	0.591
1.098	1.537	0.502
1.316	0.810	0.615
1.285	1.381	0.515
1.173	0.539	0.609
0.880	0.805	0.746
1.353	0.736	0.572
1.183	0.481	0.572
0.770	1.312	0.669
1.353	0.539	0.487
1.592	0.702	0.196
0.938	1.456	0.602
0.808	1.387	0.639
0.702	1.101	0.716
1.357	1.312	0.496
1.272	0.573	0.570
0.814	0.560	0.675
0.807	0.301	0.506

1.210	0.169	0.144
1.428	0.973	0.531
1.585	1.139	0.242
1.135	1.273	0.652
1.023	1.121	0.726

1.273	0.389	0.444
1.260	0.485	0.529
1.419	0.398	0.268
1.439	0.660	0.461
1.541	1.246	0.275

1.448	1.326	0.381
0.546	0.939	0.671
0.412	0.678	0.538
1.110	1.123	0.704
1.146	0.664	0.670

HOM-5-4

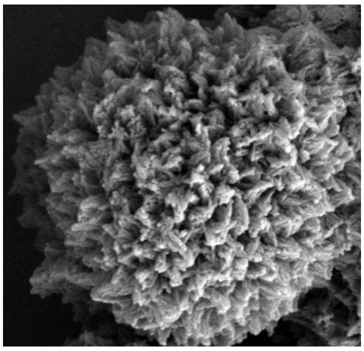

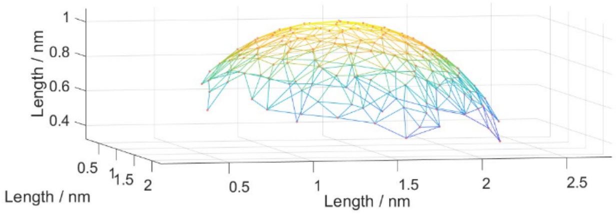

Average length = 0.163±0.050µm

Modelling function: $\frac{(x-a)^2}{A}+\frac{(y-b)^2}{B}+\frac{z^2}{C}=1$ (a=1.35; b=1.21; A=C=1.06; B=1.17)

x	y	z
1.250	2.132	0.639
1.698	2.105	0.581
1.560	2.127	0.618
0.962	2.083	0.584
1.711	2.052	0.635
1.489	1.919	0.827
1.197	1.959	0.795
1.875	1.910	0.662
1.698	1.813	0.835
0.966	1.901	0.760
1.826	1.875	0.725
1.316	1.799	0.911
1.582	1.773	0.896
1.804	1.782	0.801
2.043	1.746	0.632
0.953	1.733	0.857
1.932	1.733	0.743
1.112	1.693	0.932
1.480	1.671	0.962
1.245	1.680	0.961
1.268	1.613	0.988
2.017	1.520	0.769
1.856	1.534	0.880
1.702	1.565	0.943
1.569	1.511	0.997
1.254	1.489	1.021
1.431	1.452	1.030
2.270	1.428	0.480
1.591	1.467	1.002
2.198	1.378	0.611
1.540	1.348	1.032
1.321	1.365	1.047
1.800	1.252	0.955
1.409	1.276	1.053
2.063	1.239	0.779
1.374	1.228	1.056
1.640	1.206	1.016
2.278	1.197	0.504
1.383	1.143	1.054
1.529	1.130	1.039
2.034	1.104	0.799
2.345	1.108	0.341
1.565	1.068	1.026
1.994	1.011	0.817
2.243	0.975	0.522
2.079	0.842	0.687
1.822	0.926	0.909
1.345	0.807	0.991
2.057	0.678	0.619
2.114	1.028	0.710
1.671	0.980	0.984
1.924	1.266	0.885
2.083	0.572	0.493
2.158	1.274	0.678
0.988	1.972	0.713
1.847	1.419	0.913
1.416	0.902	1.017
1.791	1.573	0.902
2.249	1.088	0.543
1.746	1.897	0.756
1.671	1.706	0.901
1.112	1.861	0.844
1.330	1.494	1.025
1.011	0.589	0.827
0.980	1.454	0.965
0.975	1.635	0.910
1.609	0.691	0.909
1.901	1.126	0.898
1.879	0.705	0.791
1.312	2.154	0.619
1.777	2.043	0.603
1.879	0.403	0.548
1.549	2.243	0.448
0.802	1.702	0.786
1.640	1.330	1.010
1.746	1.081	0.972
1.370	2.216	0.534
1.596	1.999	0.738
1.019	1.516	0.965
1.922	1.468	0.857
1.183	1.121	1.040
1.064	1.290	1.015
1.751	1.170	0.977
1.401	1.008	1.039
1.755	0.647	0.831
1.192	1.396	1.031
1.609	0.603	0.863
1.627	0.900	0.980
1.192	0.762	0.962
1.658	1.042	0.999
1.511	1.006	1.028
2.203	0.767	0.476

2.175	0.917	0.603
1.183	0.607	0.888
1.538	0.594	0.877
1.773	2.123	0.503
1.915	1.671	0.789
1.099	1.542	0.981
0.824	1.529	0.870
0.993	1.183	0.994
1.064	2.070	0.654
1.932	0.652	0.722
1.817	0.301	0.467
1.861	1.206	0.924
0.789	1.941	0.603
1.768	1.673	0.875
0.917	1.067	0.955
0.884	0.716	0.836
1.011	0.669	0.872
0.558	1.418	0.674
1.977	1.343	0.841
1.046	1.073	1.004
0.656	0.740	0.673
0.727	1.813	0.656
1.197	0.452	0.788
1.609	0.439	0.748
2.039	1.383	0.785
0.966	1.263	0.983
1.082	1.215	1.022
0.718	0.674	0.693
0.519	1.547	0.577

1.197	0.847	0.992
0.793	1.206	0.898
1.223	0.541	0.856
1.271	0.281	0.633
1.242	0.657	0.924
1.640	0.372	0.674
0.731	1.578	0.789
0.793	0.838	0.832
1.316	1.977	0.795
1.707	0.817	0.928
1.242	0.988	1.032
1.109	0.934	0.998
1.844	0.545	0.713
0.674	0.878	0.754
1.334	0.674	0.938
1.347	0.133	0.403
0.767	1.392	0.866
0.851	1.374	0.920
0.842	0.576	0.727
1.392	0.425	0.779
1.449	0.370	0.725
0.881	1.682	0.845
1.516	0.847	0.990
1.440	1.773	0.921
0.955	0.831	0.918
1.387	0.603	0.901
0.687	1.427	0.799
1.604	0.301	0.610
1.135	0.343	0.672

1.972	1.918	0.563
0.825	0.943	0.884
0.934	0.926	0.937
0.926	0.500	0.723
1.407	0.278	0.631
1.972	0.878	0.799
1.008	1.381	0.988
0.713	1.251	0.842
0.837	1.780	0.766
1.389	2.007	0.770
2.302	0.988	0.410
0.586	1.257	0.729
0.683	1.118	0.815
1.484	0.751	0.962
1.366	0.494	0.833
0.645	1.517	0.737
1.490	2.066	0.704
1.644	2.181	0.505
1.159	2.172	0.566
1.671	1.910	0.782
1.709	1.403	0.978
1.750	1.329	0.972
1.956	1.178	0.865
2.107	1.570	0.660
2.124	1.665	0.589
2.048	1.673	0.673
1.991	1.604	0.760

HOM-5-5

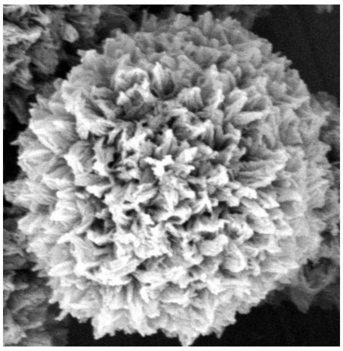

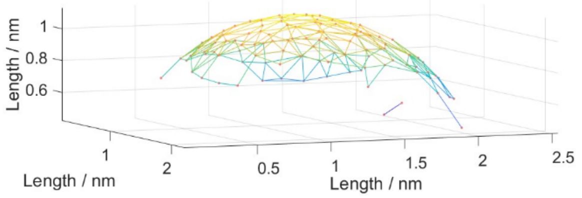

Average length = 0.182±0.049μm

Modelling function: $\frac{(x-a)^2}{A}+\frac{(y-b)^2}{B}+\frac{z^2}{C}=1$ (a=1.19; b=1.26; A=C=1.13; B=1.19)

x	y	z
1.281	2.141	0.753
2.003	1.932	0.453
1.724	1.875	0.806
1.848	1.915	0.674
1.578	1.711	0.972
1.877	1.525	0.862
1.582	1.600	1.011
1.223	1.449	1.118
1.458	1.432	1.088
1.768	1.370	0.967
2.141	1.401	0.596
0.652	1.339	0.996
1.596	1.250	1.057
1.928	1.250	0.857
0.940	1.219	1.105
0.496	1.135	0.890
0.696	1.183	1.019
1.352	1.183	1.119
1.343	1.081	1.110
0.962	0.984	1.079
1.489	1.059	1.075
1.197	0.935	1.090
0.811	0.917	1.018
1.440	0.895	1.049
0.558	0.820	0.845
1.303	0.767	1.025
1.560	0.674	0.914
0.860	0.656	0.921
1.024	0.589	0.922
1.405	0.607	0.923

0.966	0.474	0.823
1.520	0.492	0.800
1.104	0.470	0.844
1.737	0.669	0.816
0.554	1.378	0.933
1.844	0.798	0.812
0.550	1.454	0.918
1.706	0.931	0.958
1.671	1.024	1.000
0.683	1.516	0.985
0.714	0.736	0.901
2.083	0.762	0.507
1.316	0.381	0.754
1.629	0.515	0.766
2.030	0.598	0.421
1.702	0.457	0.660
1.161	0.381	0.765
0.740	1.591	0.992
1.449	0.320	0.645
1.584	0.361	0.629
1.679	0.630	0.827
1.242	0.527	0.892
1.236	1.242	1.132
1.008	1.384	1.112
0.884	1.369	1.087
0.775	1.416	1.045
0.837	1.460	1.060
0.855	1.549	1.047
1.035	1.549	1.088
1.209	1.540	1.101
1.313	1.534	1.095

1.387	1.676	1.042
1.449	1.617	1.048
1.307	1.785	1.009
1.147	1.661	1.065
1.159	1.753	1.030
1.035	1.723	1.032
0.923	1.700	1.018
0.840	1.644	1.014
0.701	1.700	0.933
0.858	1.777	0.965
0.934	1.874	0.935
1.044	1.821	0.988
0.459	1.685	0.767
0.411	1.519	0.787
1.082	1.969	0.902
1.212	1.924	0.938
1.389	1.859	0.957
1.543	1.780	0.954
1.765	1.661	0.896
1.945	1.437	0.825
2.013	1.168	0.771
1.741	1.064	0.971
1.856	0.970	0.872
0.680	0.621	0.811
0.408	0.861	0.731
0.695	0.843	0.941
0.533	1.268	0.925
0.406	1.375	0.813
0.683	1.026	0.990
0.858	0.985	1.053
1.543	0.781	0.975

1.853	0.651	0.711
1.806	0.580	0.695
1.336	0.284	0.633
0.840	0.550	0.842
0.533	1.812	0.760
0.796	1.871	0.889

1.310	2.019	0.862
1.469	1.963	0.868
1.555	1.886	0.889
1.853	1.744	0.791
1.951	1.632	0.757
2.016	1.522	0.730

2.081	1.617	0.606
2.075	1.248	0.704
1.992	1.061	0.775
1.206	1.112	1.124
1.097	1.135	1.123

HOM-6-1

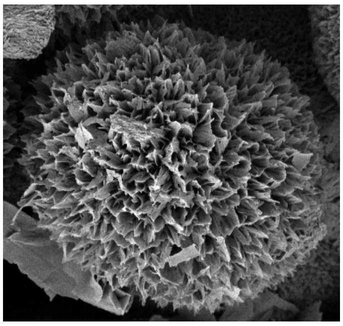

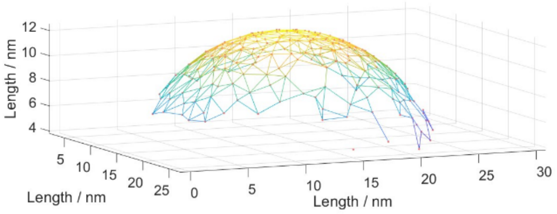

Average length = 1.59±0.492μm

Modelling function: $\frac{(x-a)^2}{A}+\frac{(y-b)^2}{B}+\frac{z^2}{C}=1$ (a=14.4; b=13.6; A=C=12.1; B=12.3)

x	y	z
18.578	24.044	4.766
17.600	23.111	7.057
12.978	22.444	8.414
17.511	22.311	8.122
12.133	22.000	8.707
19.067	21.067	8.692
16.978	21.600	9.039
8.400	21.556	7.243
20.222	21.289	7.741
11.333	20.356	9.910
12.926	19.148	11.023
8.400	20.000	8.742
10.089	19.511	10.021
11.156	19.467	10.446
14.978	18.889	11.248
20.044	18.578	9.940
17.556	18.578	10.978
12.756	18.356	11.390
9.600	17.911	10.654
11.333	17.733	11.353
6.756	17.511	9.025
5.244	18.133	7.091
10.311	17.378	11.159
15.200	16.622	12.104
16.978	16.978	11.755
6.578	16.889	9.138
8.756	16.711	10.688
17.644	16.622	11.696
7.867	16.444	10.250
21.289	16.356	10.090
13.556	16.267	12.186

12.489	16.178	12.082
10.044	15.111	11.628
5.867	16.133	8.751
14.437	13.785	12.531
11.733	15.956	11.986
13.067	15.378	12.316
7.244	15.644	10.036
22.356	15.422	9.523
16.533	15.156	12.246
18.437	15.000	11.784
6.356	14.978	9.468
20.800	14.756	10.727
11.778	13.733	12.246
6.756	13.644	9.903
8.178	13.244	10.853
15.422	13.244	12.490
4.489	13.333	7.620
14.356	12.533	12.487
17.489	12.452	12.101
10.044	13.022	11.725
12.844	12.622	12.393
7.289	11.111	9.978
7.956	12.444	10.665
10.311	11.022	11.540
9.378	11.644	11.294
13.607	9.281	11.697
9.200	9.822	10.711
17.733	8.622	10.962
9.637	8.126	10.124
16.400	7.689	10.789
8.311	7.156	8.703
20.489	22.089	6.549

14.941	20.304	10.408
22.444	19.689	7.275
11.022	16.711	11.612
10.267	14.044	11.808
4.844	12.533	7.995
7.244	8.400	8.769
16.037	19.593	10.757
22.000	18.089	8.833
5.156	16.667	7.788
13.156	8.756	11.436
20.000	13.467	11.230
15.911	12.444	12.392
4.578	11.422	7.415
12.711	10.889	12.102
19.030	11.326	11.430
14.844	6.533	10.207
6.311	19.644	7.182
4.244	16.244	6.746
22.667	21.333	4.992
6.222	12.267	9.370
17.689	9.911	11.501
12.844	9.600	11.738
10.178	21.822	8.134
24.756	18.133	5.321
16.711	16.178	12.025
20.215	15.474	10.945
16.178	9.067	11.510
23.956	13.556	8.153
9.844	8.807	10.576
21.067	13.911	10.630
9.956	20.444	9.299
13.904	18.526	11.424

7.422	19.778	8.164
11.296	15.000	12.042
6.000	14.267	9.239
18.311	13.511	11.919
21.911	21.289	6.126
17.644	15.778	11.898
13.778	6.489	10.161
5.111	18.711	6.467
17.333	17.644	11.442
19.415	15.948	11.235
14.978	5.867	9.674
13.022	23.689	6.758
22.711	16.267	8.990
16.489	13.467	12.363
6.933	7.244	7.626
25.022	16.489	5.992
23.600	15.022	8.417
8.533	15.022	10.952
21.156	11.911	10.441
20.800	11.778	10.639
15.156	24.889	4.437
18.667	7.111	9.735
3.822	13.600	6.663
8.489	8.178	9.527
21.556	20.444	7.475
20.044	16.933	10.655
4.667	15.644	7.550
21.644	14.489	10.209
20.533	9.556	10.138
11.563	7.741	10.614
15.689	5.111	8.904
21.911	22.578	3.808
12.311	9.556	11.637
8.622	18.267	9.983
23.822	20.533	4.143
10.052	7.148	9.692
25.422	15.689	5.620
12.622	6.844	10.277

25.378	17.556	4.512
22.489	12.978	9.585
15.511	17.911	11.651
22.667	11.067	9.094
26.178	12.711	4.295
13.511	10.356	12.052
12.311	8.178	11.028
9.689	5.644	8.223
25.156	14.444	6.431
8.356	14.444	10.921
20.533	20.356	8.407
18.933	17.867	10.821
17.333	6.978	10.120
12.489	6.267	9.823
24.044	19.200	5.557
13.644	4.400	8.177
21.200	8.444	9.131
11.422	6.356	9.621
23.911	11.111	7.804
10.881	9.756	11.356
12.311	11.956	12.239
23.378	9.778	7.864
23.022	8.844	7.721
10.800	4.622	7.652
11.000	9.104	11.138
9.104	7.889	9.710
10.733	8.274	10.654
14.141	9.489	11.802
14.378	10.170	12.033
15.148	10.289	12.047
14.674	11.326	12.316
16.244	10.763	12.060
16.007	11.563	12.261
16.778	12.096	12.219
18.230	11.207	11.695
18.526	9.815	11.197
18.585	9.044	10.866
19.119	8.185	10.212

19.800	10.763	10.952
19.859	12.570	11.253
19.326	13.756	11.538
17.400	13.578	12.178
15.356	15.770	12.290
16.363	18.111	11.458
17.430	19.178	10.699
16.867	19.622	10.583
18.407	19.919	9.910
13.281	23.178	7.556
15.148	23.681	6.880
15.770	23.000	7.764
14.081	21.874	9.124
6.052	17.726	8.263
7.652	18.378	9.290
4.363	17.222	6.427
5.044	14.556	8.234
3.770	11.622	6.267
5.637	10.763	8.442
5.252	9.667	7.518
22.881	14.763	9.178
22.319	14.022	9.733
13.578	5.311	9.148
12.007	5.222	8.771
20.926	21.074	7.401
23.296	16.807	8.208
23.030	17.874	7.963
19.652	7.000	9.161
19.652	7.593	9.584
20.541	7.119	8.690
22.052	7.593	7.814
23.207	7.711	6.605
24.807	12.304	6.917
23.919	12.600	8.135
10.970	13.489	12.044
11.978	15.059	12.192
15.089	14.378	12.488

HOM-6-2

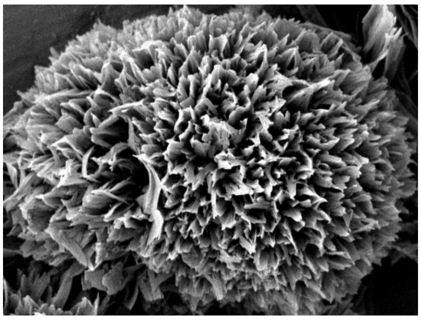

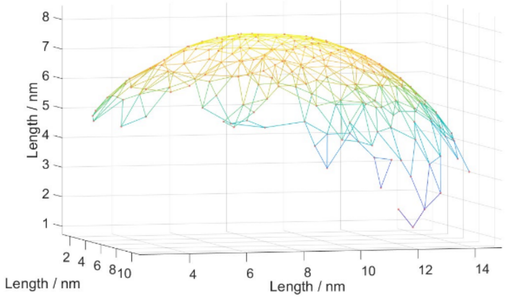

Average length = 0.896±0.301μm

Modelling function: $\frac{(x-a)^2}{A}+\frac{(y-b)^2}{B}+\frac{z^2}{C}=1$ (a=7.97; b=5.91; A=C=7.97; B=5.60)

x	y	z
10.604	10.226	4.205
9.890	9.991	4.973
6.566	9.830	5.378
5.528	9.774	5.076
9.698	9.509	5.707
12.358	9.340	4.313
10.509	9.340	5.604
11.208	9.189	5.415
6.019	7.264	7.292
8.981	9.038	6.375
7.434	9.000	6.467
10.057	8.981	6.164
13.340	8.962	3.708
8.604	8.642	6.763
7.208	8.717	6.689
9.491	8.415	6.791
4.717	8.340	6.208
7.962	8.377	6.985
12.623	8.283	5.295
11.472	8.340	6.074
4.113	8.151	5.996
3.472	7.302	6.045
8.245	7.849	7.294
7.434	8.057	7.166
10.660	8.038	6.676
7.736	7.755	7.342
6.396	7.679	7.212
12.355	7.588	5.990
11.868	7.623	6.300
7.038	7.547	7.381

9.887	7.566	7.181
10.679	7.453	6.972
11.245	7.415	6.741
10.245	7.170	7.232
13.208	7.396	5.371
9.245	6.717	7.593
7.097	6.808	7.629
12.981	7.132	5.706
8.170	6.811	7.676
13.717	7.019	5.015
12.604	6.811	6.124
11.906	6.377	6.681
10.717	6.585	7.219
3.170	6.509	6.064
10.028	6.500	7.458
13.906	6.264	5.007
10.151	5.943	7.469
3.962	6.075	6.663
13.075	6.075	5.868
9.604	6.094	7.603
6.453	5.491	7.608
2.245	5.321	5.202
10.943	5.547	7.173
9.849	5.358	7.511
13.604	5.283	5.297
7.358	5.264	7.704
12.981	5.283	5.889
7.000	5.000	7.615
11.038	5.075	7.056
5.528	5.075	7.295
7.792	5.075	7.691

14.113	4.943	4.585
10.396	4.264	7.031
3.443	4.877	6.162
5.981	4.849	7.376
8.057	4.566	7.553
6.943	4.321	7.390
12.491	4.774	6.134
5.094	4.585	6.991
7.472	4.660	7.568
9.434	4.509	7.390
13.679	4.509	4.917
6.057	4.189	7.153
3.557	4.330	6.019
11.934	4.274	6.299
10.774	4.208	6.863
4.679	4.132	6.603
12.491	4.113	5.822
4.943	3.906	6.605
2.613	3.708	4.741
8.019	2.962	6.617
8.962	3.660	7.057
9.566	3.830	7.047
6.623	3.811	7.087
7.097	3.752	7.127
3.764	3.255	5.408
6.792	3.226	6.728
12.321	3.057	5.093
7.283	2.981	6.598
5.877	3.349	6.596
5.575	3.104	6.294
10.019	3.189	6.487

9.453	3.170	6.624
8.396	3.038	6.668
4.887	2.057	4.734
10.057	2.377	5.669
7.811	2.396	6.059
6.472	2.528	6.021
5.566	2.547	5.741
10.623	2.377	5.427
6.981	1.660	4.975
11.321	1.755	4.007
8.623	1.358	4.493
8.075	1.283	4.390
10.981	1.434	3.589
9.264	1.302	4.237
8.774	9.792	5.552
8.642	7.566	7.402
12.019	2.113	4.047
3.123	4.255	5.634
10.547	1.698	4.441
13.245	8.019	4.914
8.679	3.113	6.705
8.566	9.208	6.261
6.321	8.736	6.512
12.208	5.642	6.515
3.443	5.311	6.271
12.811	9.509	3.483
11.321	7.792	6.518
12.264	5.302	6.434
13.283	3.811	4.882
2.434	4.245	4.952
13.358	2.566	3.159
13.377	6.849	5.442
11.981	10.151	3.126
11.434	2.453	5.052
13.151	9.679	2.513
10.415	8.811	6.191

5.830	9.981	4.897
12.434	10.264	2.016
11.660	10.075	3.671
11.000	9.283	5.424
12.849	2.208	3.216
11.434	1.321	2.824
10.104	10.292	4.354
9.453	2.340	5.813
9.208	4.887	7.549
12.547	3.396	5.237
14.189	7.434	4.171
14.434	6.358	4.288
9.513	10.381	4.428
6.528	8.113	7.006
7.585	9.283	6.200
12.651	6.349	6.186
14.519	5.255	4.104
14.943	5.283	3.343
13.170	3.509	4.734
5.585	4.415	7.108
13.132	2.019	2.177
6.264	9.585	5.618
7.642	6.396	7.744
11.925	10.623	1.440
11.566	5.283	6.845
6.000	5.245	7.469
11.509	4.226	6.524
14.651	5.896	3.990
11.396	10.717	2.062
13.642	7.849	4.596
12.921	4.783	5.796
10.887	9.887	4.640
6.802	8.726	6.623
7.110	5.802	7.731
11.472	6.132	6.941
6.708	6.544	7.626

6.431	6.267	7.610
8.607	6.255	7.739
9.324	7.123	7.474
9.336	7.135	7.468
9.135	8.104	7.063
5.437	5.613	7.344
7.764	3.676	7.133
9.110	4.079	7.265
9.890	4.192	7.153
10.179	4.858	7.316
9.538	5.638	7.612
11.035	5.978	7.151
11.752	5.840	6.799
10.657	3.777	6.675
10.947	3.097	6.036
11.903	3.261	5.618
5.299	8.217	6.566
4.280	7.160	6.624
4.443	6.066	6.930
2.764	4.915	5.613
3.443	3.708	5.539
4.431	2.934	5.562
7.047	2.318	5.901
7.399	1.890	5.392
8.355	2.456	6.116
8.796	2.192	5.764
9.802	2.808	6.217
7.462	7.110	7.582
7.664	8.531	6.869
7.097	9.588	5.803
11.437	9.575	4.756
12.456	8.846	4.878
11.877	8.607	5.590
14.708	7.638	3.065
14.142	5.965	4.739
3.953	2.330	4.437

HOM-6-3

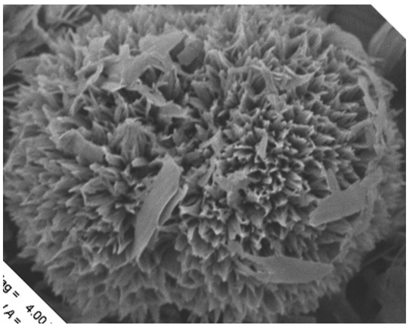

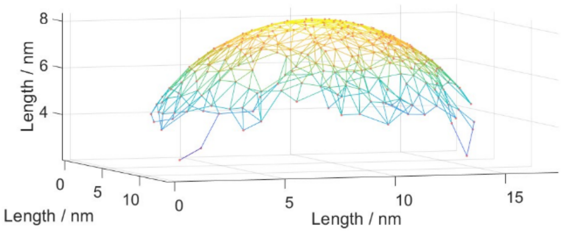

Average length = 0.857±0.337μm

Modelling function: $\frac{(x-a)^2}{A} + \frac{(y-b)^2}{B} + \frac{z^2}{C} = 1$ (a=8.63; b=6.76; A=C=8.35; B=6.48)

x	y	z
10.182	6.102	8.164
4.341	6.205	7.132
5.818	3.261	6.446
10.989	8.273	7.770
13.205	4.761	6.498
4.920	3.602	6.282
10.295	9.920	7.095
11.489	7.591	7.774
11.932	6.057	7.619
11.909	7.398	7.637
11.614	7.091	7.790
7.784	8.898	7.837
9.170	9.795	7.356
9.955	6.466	8.238
2.420	4.898	5.046
12.273	2.807	5.529
12.761	7.489	7.198
5.455	1.091	2.516
8.580	8.989	7.840
11.080	2.443	5.731
8.977	1.989	5.645
4.386	1.409	2.054
7.807	1.125	4.047
12.716	7.807	7.158
9.148	6.784	8.336
8.034	1.409	4.678
12.841	6.864	7.212
12.125	4.534	7.025
5.341	1.750	4.157
11.193	9.920	6.823
12.023	9.443	6.800

11.636	8.034	7.617
13.648	6.966	6.672
12.080	5.636	7.469
6.170	1.398	3.998
7.898	7.875	8.194
2.466	5.693	5.467
2.500	3.545	3.879
3.693	3.318	5.073
13.307	5.420	6.703
11.364	5.034	7.574
3.705	2.489	3.904
4.534	2.295	4.461
11.568	1.875	4.640
8.727	1.716	5.247
5.318	4.977	7.317
11.898	7.932	7.536
10.443	7.409	8.110
10.955	4.602	7.527
5.273	4.352	6.992
9.364	2.295	6.012
8.773	7.818	8.238
10.341	7.170	8.158
10.955	6.364	8.007
10.239	5.364	7.997
6.273	4.136	7.266
5.443	2.273	5.118
14.023	5.727	6.239
13.795	3.091	4.557
3.807	6.932	6.815
9.670	6.648	8.286
12.830	3.114	5.485
8.080	10.000	7.208

10.864	6.727	8.048
12.534	6.273	7.358
2.023	5.307	4.756
9.057	8.795	7.916
9.705	8.170	8.080
13.386	6.136	6.820
12.386	5.034	7.123
12.852	4.398	6.535
13.568	2.727	4.290
13.330	2.602	4.358
11.386	8.295	7.631
3.614	7.705	6.565
9.466	6.420	8.299
9.182	5.773	8.237
9.261	9.182	7.719
11.341	8.886	7.408
7.307	6.807	8.247
12.068	5.057	7.291
9.045	4.432	7.786
7.239	8.227	8.014
6.943	4.284	7.534
10.352	3.943	7.325
12.227	3.091	5.874
9.341	7.398	8.281
12.568	5.205	7.090
3.966	11.148	3.991
10.659	7.136	8.088
13.955	6.170	6.391
12.898	5.795	7.072
10.955	5.364	7.819
11.841	4.716	7.249
10.920	10.239	6.659

13.318	9.443	5.982
11.216	7.170	7.924
9.932	3.489	7.094
3.011	4.318	5.320
5.602	6.750	7.784
11.295	6.682	7.916
10.182	6.580	8.204
13.545	3.966	5.716
9.182	10.295	6.974
5.545	8.875	7.265
3.125	7.182	6.257
10.625	1.864	5.099
5.273	7.216	7.625
2.614	6.909	5.790
4.534	5.557	7.113
7.795	4.795	7.917
11.659	3.966	6.903
2.932	7.966	5.904
5.784	7.784	7.740
12.205	6.170	7.511
10.648	5.500	7.942
1.932	4.807	4.311
11.841	3.193	6.193
9.716	2.818	6.543
7.295	2.636	6.306
12.068	8.500	7.272
10.489	5.125	7.867
15.000	4.716	4.720
8.227	10.693	6.620
4.955	10.625	5.600
10.500	6.580	8.137
9.795	6.080	8.225
14.909	5.477	5.256
10.205	4.727	7.775
11.170	4.545	7.429
15.045	4.318	4.329
10.409	9.364	7.436
4.636	7.136	7.319
3.670	4.477	6.044
9.739	3.773	7.331
14.205	3.000	3.904
6.250	2.659	6.016
11.636	2.330	5.307
11.386	8.034	7.711

7.875	3.943	7.486
14.284	3.352	4.306
8.182	3.318	7.065
5.744	10.983	5.632
8.557	9.943	7.271
12.307	6.000	7.436
7.500	3.239	6.923
9.523	11.818	5.134
13.818	7.318	6.506
14.011	5.057	6.002
7.580	4.420	7.720
6.920	3.386	6.925
12.580	9.193	6.655
8.682	8.080	8.176
13.989	7.909	6.232
13.114	7.898	6.892
13.159	6.568	7.014
3.489	5.364	6.333
14.761	3.568	3.910
4.886	8.682	7.041
2.273	7.773	5.256
10.295	5.750	8.082
7.114	5.705	8.101
13.864	3.795	5.273
8.750	10.864	6.456
12.148	7.091	7.563
8.455	6.273	8.327
14.909	6.261	5.472
13.966	4.409	5.669
14.386	3.898	4.802
6.466	3.477	6.871
10.585	2.347	5.799
9.432	10.864	6.408
9.511	5.443	8.132
9.420	2.875	6.641
11.830	2.784	5.771
4.102	10.636	4.921
11.000	9.432	7.228
4.170	8.955	6.467
8.017	7.415	8.286
14.295	6.830	6.138
13.795	5.148	6.229
7.091	2.943	6.575
6.625	2.659	6.151

14.330	6.455	6.093
4.000	5.136	6.630
10.261	3.432	6.981
3.636	10.330	4.855
2.795	8.489	5.542
13.295	8.352	6.616
12.625	8.170	7.105
4.830	7.750	7.326
1.625	7.284	4.497
8.716	6.261	8.328
15.693	4.989	3.834
12.602	2.466	4.837
11.114	1.466	4.132
12.511	5.659	7.260
11.750	6.784	7.748
1.614	6.761	4.531
8.455	7.091	8.339
7.716	6.000	8.245
4.784	10.591	5.523
6.648	9.545	7.273
1.875	8.125	4.584
14.352	7.102	6.069
6.955	4.784	7.778
9.341	4.682	7.881
8.807	2.670	6.479
10.148	2.125	5.640
4.875	9.670	6.445
4.125	9.648	5.963
2.034	6.727	5.123
8.352	5.227	8.112
4.182	4.341	6.347
3.034	9.636	4.965
5.625	6.216	7.762
5.739	4.182	7.099
8.307	2.875	6.680
9.886	10.727	6.478
3.182	6.557	6.326
14.420	6.091	5.959
5.557	7.670	7.676
14.773	6.898	5.657
1.898	6.420	4.925
7.375	5.432	8.079
10.830	4.034	7.253
8.909	12.136	4.642

11.000	11.477	5.204
5.784	8.545	7.506
11.080	4.818	7.584
14.455	4.659	5.342
9.489	10.216	7.008
7.773	9.409	7.571
11.625	5.534	7.637
11.455	11.500	4.935
1.364	7.886	3.852
12.182	6.602	7.557
7.784	11.398	5.763
15.523	7.511	4.616
6.148	8.159	7.767
10.432	11.352	5.603
14.898	7.841	5.341
13.136	5.966	6.959
10.148	3.261	6.867
11.966	10.023	6.394
14.716	10.125	3.722

14.932	7.148	5.459
14.932	9.659	4.005
9.330	8.432	8.038
4.523	10.261	5.697
7.477	10.477	6.738
8.295	4.273	7.707
10.159	12.239	4.175
8.080	12.136	4.618
6.273	11.489	5.192
14.080	11.205	2.674
12.898	9.648	6.135
2.841	8.898	5.349
4.432	5.068	6.885
3.625	9.034	6.006
10.091	11.773	5.077
11.466	9.307	7.134
10.727	9.727	7.120
10.966	8.727	7.606
2.216	8.773	4.675

12.932	10.330	5.479
11.625	9.852	6.697
13.455	9.943	5.440
9.750	7.057	8.268
3.733	8.585	6.341
3.403	8.017	6.308
4.688	6.562	7.359
6.585	6.722	8.098
6.290	5.449	7.839
9.097	11.494	5.676
8.540	11.403	5.817
8.074	11.642	5.455
6.653	11.040	5.944
6.392	10.517	6.421
4.699	11.358	4.369
13.494	10.460	4.826
13.460	10.994	4.071
14.062	10.153	4.590
13.153	9.938	5.698

HOM-6-4

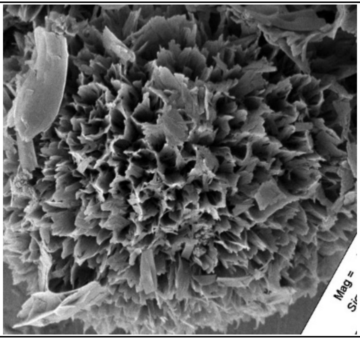

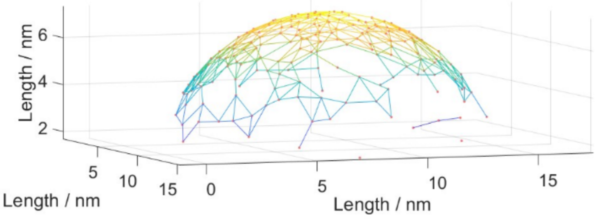

Average length = 1.04±0.309μm

Modelling function: $\frac{(x-a)^2}{A}+\frac{(y-b)^2}{B}+\frac{z^2}{C}=1$ (a=8.32; b=7.57; A=C=7.35; B=7.01)

x	y	z
6.167	8.917	6.886
7.722	8.167	7.301
4.500	8.472	6.210
6.306	9.500	6.775
8.611	7.472	7.345
4.917	4.556	5.698
3.167	6.778	5.177
4.139	7.361	6.043
5.000	6.583	6.477
11.111	11.028	5.755
4.444	12.111	4.046
3.611	5.889	5.363
3.389	11.667	3.361
2.333	10.417	3.050
9.722	9.528	6.919
1.889	7.500	3.562
3.083	10.500	4.147
3.282	9.875	4.779
2.444	7.472	4.417
9.111	7.083	7.291
7.028	13.361	3.941
5.694	7.417	6.865
8.750	6.500	7.252
2.139	8.972	3.700
4.750	11.333	5.074
6.250	11.167	5.962
5.083	7.889	6.592
6.153	10.208	6.458
3.694	9.417	5.376
9.056	9.306	7.085
7.306	14.333	1.661
5.301	4.060	5.602
9.333	9.417	7.020
9.222	3.917	6.209
6.861	2.556	4.926
9.028	13.111	4.451
5.889	10.750	6.085
6.167	6.611	6.957
7.333	8.806	7.169
5.167	12.500	4.172
1.361	8.639	2.091
8.167	3.222	5.764
2.417	8.000	4.359
7.389	6.361	7.181
2.611	5.861	4.271
5.764	5.116	6.394
4.667	4.111	5.248
5.394	3.134	4.883
7.861	12.722	4.967
9.972	10.806	6.310
6.778	10.250	6.617
8.222	9.194	7.151
6.806	7.611	7.194
3.500	3.750	3.842
3.917	3.611	4.174
9.667	11.694	5.792
8.472	5.778	7.105
6.500	4.000	6.059
12.361	13.056	2.155
11.889	10.972	5.347
3.722	8.472	5.658
7.333	4.444	6.505
4.889	10.778	5.565
6.444	8.083	7.088
6.361	5.528	6.754
4.389	10.833	5.187
9.000	5.722	7.058
6.056	12.944	4.146
11.500	12.444	4.223
4.389	9.944	5.693
2.167	6.917	3.965
6.417	6.194	6.953
11.139	5.833	6.540
8.278	3.611	6.066
4.472	9.194	6.029
3.639	9.056	5.451
8.917	13.806	3.310
8.361	13.472	3.971
3.333	8.750	5.259
10.722	10.472	6.247
7.750	10.111	6.829
7.111	8.361	7.204
2.889	6.167	4.731
2.750	11.056	3.111
7.167	9.611	6.939
6.944	6.806	7.177
4.889	13.444	2.089
6.806	10.778	6.360
10.250	9.556	6.782
14.194	8.056	4.391
6.750	3.389	5.688
11.083	11.889	5.091
1.833	9.278	2.961
11.639	5.917	6.326
3.083	5.250	4.550

4.778	6.111	6.258
7.000	5.278	6.821
7.528	10.861	6.444
11.306	9.722	6.328
14.556	7.028	3.850
8.028	6.778	7.299
4.417	4.389	5.261
8.856	3.394	5.880
5.389	12.111	4.775
8.861	11.556	6.025
9.250	10.056	6.811
14.667	9.806	2.875
8.111	6.361	7.238
3.306	4.611	4.390
6.250	13.528	3.280
3.139	11.944	2.488
13.222	5.583	5.066
10.333	5.083	6.571
3.667	4.639	4.790
5.528	13.306	3.178
10.444	7.111	7.021
7.472	3.889	6.198
7.028	2.167	4.502
8.889	11.250	6.233
9.944	10.278	6.584
8.417	10.917	6.460
11.028	4.889	6.229
1.528	8.083	2.763

13.917	6.472	4.624
8.000	4.750	6.722
10.556	8.389	6.950
13.306	10.778	4.228
12.856	8.801	5.639
1.694	6.806	3.083
13.500	6.722	5.139
4.250	5.194	5.592
9.750	4.000	6.162
8.889	8.528	7.261
12.579	6.505	5.887
7.056	12.222	5.355
11.694	9.306	6.273
2.611	5.167	3.886
14.944	5.722	2.530
4.764	2.856	4.118
14.444	4.389	2.324
9.227	4.912	6.741
9.500	2.833	5.288
8.778	4.528	6.607
12.167	4.833	5.568
12.333	2.944	3.794
4.412	11.690	4.487
13.838	5.560	4.375
12.500	7.639	6.047
11.889	7.167	6.413
13.778	10.333	3.983
8.500	2.000	4.460

3.500	8.028	5.531
8.778	10.528	6.650
5.333	11.194	5.541
13.722	3.194	1.947
11.833	3.889	5.176
13.653	9.394	4.685
10.583	3.583	5.607
3.944	11.889	3.796
6.333	1.889	3.821
9.778	2.222	4.523
5.944	3.528	5.516
10.167	1.667	3.508
13.116	9.616	5.142
12.542	10.097	5.404
12.838	10.931	4.607
12.986	11.671	3.714
12.690	3.560	4.154
12.968	4.468	4.674
13.412	4.653	4.330
2.245	6.301	3.921
11.005	9.042	6.668
11.153	7.579	6.784
7.319	7.819	7.279
7.690	9.394	7.071
5.505	5.986	6.585
11.634	8.486	6.492

HOM-6-5

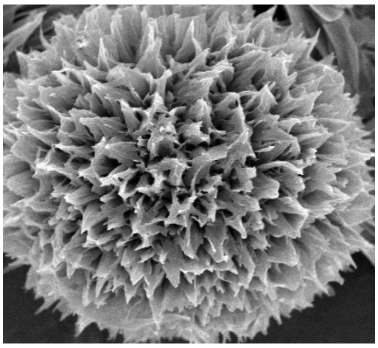

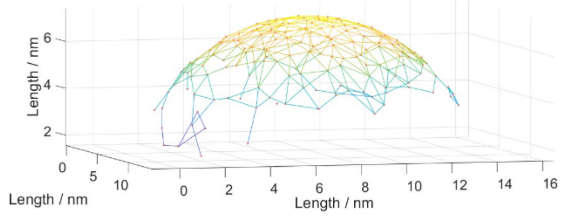

Average length =1.05±0.327μm

Modelling function: $\frac{(x-a)^2}{A}+\frac{(y-b)^2}{B}+\frac{z^2}{C}=1$ (a=7.54; b=6.97; A=C=7.43; B=6.76)

x	y	z
5.109	12.158	4.100
5.960	11.960	4.759
7.129	11.564	5.434
5.485	11.386	5.237
10.772	11.347	4.644
7.960	11.050	5.907
9.584	11.010	5.591
7.485	10.970	5.987
8.594	10.911	5.941
1.743	10.851	1.855
6.594	10.614	6.185
5.149	10.594	5.798
9.168	10.376	6.204
7.703	10.356	6.426
8.673	10.040	6.518
5.901	9.485	6.697
3.624	9.644	5.588
9.941	9.564	6.422
3.267	9.327	5.498
5.446	9.347	6.631
7.525	9.089	7.052
12.356	8.436	5.413
7.287	8.475	7.235
5.465	8.475	6.937
10.535	8.337	6.625
13.545	8.396	4.070
5.545	8.337	6.995
2.614	7.921	5.462
13.525	7.624	4.329
7.743	7.941	7.346

11.188	7.881	6.387
4.871	7.663	6.890
5.644	7.584	7.149
12.376	7.505	5.599
6.356	7.307	7.322
4.436	7.129	6.746
12.188	7.079	5.785
3.228	6.931	6.049
11.832	6.723	6.049
10.317	6.891	6.884
1.703	6.653	4.584
7.228	6.515	7.402
12.792	6.218	5.178
10.198	6.139	6.871
4.218	5.822	6.523
2.356	5.842	5.176
11.069	5.822	6.407
10.020	5.307	6.755
8.238	5.485	7.210
6.594	5.089	7.070
8.000	4.990	7.085
12.356	4.752	5.095
6.139	4.911	6.934
2.079	4.297	4.094
9.842	4.653	6.583
3.248	4.653	5.504
5.960	4.475	6.719
6.990	4.356	6.826
5.030	4.119	6.250
8.257	4.000	6.632
12.733	4.099	4.263

6.337	3.762	6.426
1.980	3.683	3.355
10.812	3.465	5.439
9.465	3.624	6.157
3.327	2.832	4.097
8.277	3.050	6.005
6.733	2.733	5.731
8.851	2.931	5.808
4.911	2.871	5.291
9.257	2.683	5.478
11.604	2.337	3.563
4.099	1.644	3.021
10.337	1.901	4.037
8.376	1.861	4.791
6.119	1.149	3.503
9.386	1.426	3.827
3.267	1.624	1.569
7.822	0.891	3.239
9.604	11.861	4.692
10.198	11.228	5.118
13.881	6.733	3.846
6.158	5.683	7.159
10.178	1.624	3.699
5.960	11.465	5.320
8.871	7.089	7.303
12.950	5.307	4.741
7.861	12.257	4.619
12.040	10.614	4.340
4.455	9.149	6.319
5.960	9.010	6.903
1.941	9.010	4.340

10.158	7.663	6.905
8.554	6.356	7.324
2.396	5.228	5.007
5.743	10.554	6.037
12.040	5.584	5.703
6.139	5.564	7.128
2.772	4.337	4.909
8.535	12.238	4.548
3.446	12.158	2.445
2.970	10.614	4.278
10.653	9.109	6.317
11.287	4.653	5.881
8.693	8.574	7.120
6.178	6.792	7.298
12.257	6.515	5.708
8.198	6.099	7.334
4.178	11.188	4.736
8.733	7.901	7.257
8.614	7.584	7.316
3.683	7.050	6.348
7.089	5.584	7.254
4.535	11.723	4.348
4.911	9.802	6.212
11.267	9.505	5.785

6.851	8.970	7.061
8.891	12.733	3.642
7.743	11.366	5.639
10.772	9.901	5.857
1.604	9.901	3.101
14.059	7.327	3.524
5.347	3.426	5.933
7.624	9.822	6.733
9.564	7.644	7.104
4.238	3.683	5.589
6.950	2.317	5.356
3.950	11.693	3.924
2.584	8.802	5.156
4.158	8.000	6.516
2.535	6.554	5.471
8.851	5.228	7.053
9.386	12.416	3.995
8.950	11.644	5.177
8.931	11.208	5.616
8.099	8.891	7.097
5.663	5.545	7.013
2.752	5.366	5.400
4.178	4.911	6.226
7.188	7.545	7.391

5.426	6.396	7.092
12.574	9.109	4.922
11.010	8.772	6.257
4.673	10.297	5.797
10.564	4.802	6.348
3.050	3.901	4.865
4.178	8.990	6.241
4.297	10.079	5.745
6.337	8.178	7.207
6.950	3.743	6.499
6.139	7.960	7.212
6.970	9.426	6.896
5.911	2.782	5.599
1.218	5.238	3.409
6.446	12.644	3.892
7.277	12.386	4.440
11.950	3.733	4.796
12.089	2.881	3.772
6.366	1.713	4.522
2.208	2.822	2.453
2.505	10.168	4.185
2.129	7.792	5.011
4.584	1.119	2.269
2.426	2.307	1.679

HOM-7-1

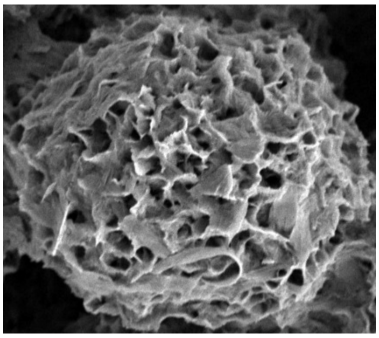

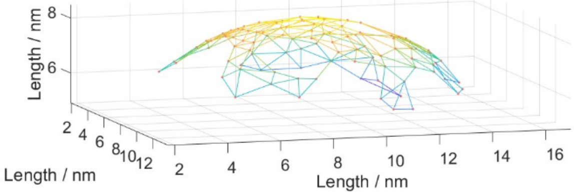

Average length =1.06±0.316μm

Modelling function: $\frac{(x-a)^2}{A}+\frac{(y-b)^2}{B}+\frac{z^2}{C}=1$ (a=8.82; b=7.89; A=C=8.48; B=7.35)

x	y	z
7.296	7.000	8.281
6.981	6.167	8.039
6.463	6.259	7.929
6.463	7.130	8.102
7.130	5.481	7.835
6.981	4.796	7.473
6.463	5.463	7.653
5.852	5.407	7.414
6.130	4.593	7.090
5.463	6.463	7.616
6.519	8.185	8.158
7.611	8.111	8.392
8.556	8.130	8.473
8.852	6.963	8.414
8.537	5.815	8.132
7.889	4.759	7.619
8.759	4.907	7.752
8.407	4.333	7.412
9.352	5.796	8.113
9.944	6.519	8.256
9.463	7.204	8.420
9.407	8.074	8.458
10.407	7.722	8.328
11.000	7.407	8.176
10.463	6.685	8.203
10.130	5.481	7.905
11.481	5.222	7.440
10.463	4.648	7.432
9.926	3.574	6.777
9.444	4.074	7.223
10.389	3.963	6.996
12.000	4.667	6.926
12.426	4.296	6.459
11.130	3.352	6.259
12.407	6.648	7.549
13.315	6.667	7.049
12.944	7.130	7.356
12.611	7.722	7.582
12.056	8.648	7.789
13.315	8.204	7.180
13.148	8.944	7.189
12.222	9.685	7.485
11.426	10.278	7.584
11.963	10.574	7.241
12.741	10.759	6.750
11.667	9.259	7.830
10.648	11.537	7.132
11.944	11.056	6.985
11.241	11.981	6.615
10.648	11.019	7.452
9.796	10.852	7.700
9.519	11.500	7.354
9.037	11.056	7.651
9.000	10.463	7.943
9.704	10.019	8.069
9.111	9.759	8.197
8.315	10.111	8.069
8.500	9.556	8.255
7.741	8.870	8.337
7.407	9.537	8.145
8.778	8.889	8.403
9.778	9.093	8.311
10.315	9.556	8.123
6.852	8.889	8.170
6.333	9.444	7.910
6.074	9.000	7.924
5.704	9.148	7.756
6.056	9.870	7.688
5.463	10.630	7.121
5.241	9.907	7.331
4.815	10.519	6.836
5.815	7.722	7.931
5.389	7.315	7.731
6.019	6.685	7.886
4.926	7.019	7.471
4.037	7.370	6.982
3.796	6.074	6.509
4.574	5.389	6.755
5.056	5.111	6.895
8.148	3.333	6.623
7.852	2.926	6.183
8.704	2.630	5.926
10.167	2.796	5.965
9.444	3.333	6.627
12.852	3.519	5.497
12.296	3.222	5.552
13.685	3.630	4.907
14.611	6.019	5.804
14.074	6.630	6.493
14.907	6.796	5.764
14.019	7.463	6.679
13.537	11.130	5.971

13.870	10.593	6.054
12.870	10.241	6.937
10.093	12.833	6.146
11.296	12.870	5.724

10.500	13.074	5.772
7.241	11.963	6.883
7.870	12.130	6.863
7.093	12.593	6.287

6.519	11.426	7.072
6.259	11.815	6.700
4.981	11.593	6.244

HOM-7-2	
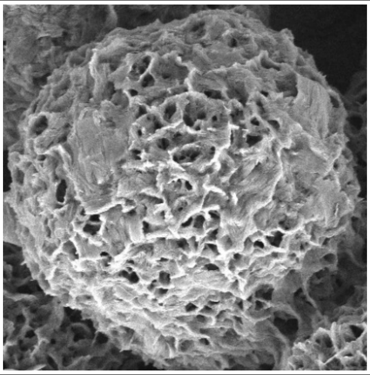	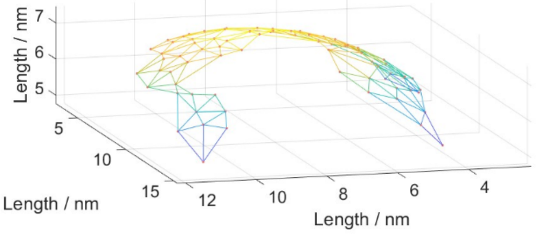<br>Average length =0.901±0.291μm

Modelling function: $\frac{(x-a)^2}{A}+\frac{(y-b)^2}{B}+\frac{z^2}{C}=1$ (a=7.85; b=8.05; A=C=7.46; B=7.51)

x	y	z
7.630	9.685	7.280
8.111	9.759	7.262
8.167	9.222	7.364
8.593	8.981	7.368
8.944	9.278	7.281
8.815	9.741	7.207
9.537	9.407	7.144
9.444	8.796	7.253
8.296	7.981	7.450
7.907	8.148	7.462
7.889	7.481	7.442
7.259	7.759	7.434
7.019	8.611	7.395
6.204	10.000	7.015
6.167	9.389	7.147
6.685	9.685	7.189
6.537	9.056	7.277
7.204	10.130	7.141
8.037	10.667	6.991
8.500	10.741	6.936
8.778	10.352	7.042
9.333	10.296	6.965
9.481	10.815	6.743
9.130	11.019	6.733
8.574	11.519	6.578

9.630	11.500	6.384
10.167	10.852	6.524
10.556	10.907	6.349
10.852	10.574	6.355
11.500	10.630	5.983
11.204	10.019	6.373
11.852	9.907	6.023
12.333	10.000	5.643
11.963	10.556	5.708
7.352	6.574	7.301
7.889	6.463	7.295
7.704	6.926	7.378
8.648	6.407	7.239
9.296	6.296	7.112
8.370	5.574	7.028
8.630	5.185	6.856
8.130	5.444	6.995
7.778	5.056	6.845
7.815	4.333	6.487
8.667	4.500	6.528
9.407	4.926	6.608
6.648	5.722	6.994
7.315	5.574	7.026
6.556	4.704	6.556
9.426	3.741	5.909
9.241	4.148	6.226

8.481	3.704	6.056
10.481	5.463	6.496
11.204	5.685	6.241
10.593	6.333	6.729
10.963	4.815	5.975
5.537	6.222	6.859
6.167	6.407	7.085
6.000	6.981	7.152
5.426	6.889	6.963
5.778	5.259	6.612
5.963	4.167	6.104
5.370	3.630	5.502
5.963	3.648	5.746
6.315	2.944	5.256
6.704	3.444	5.784
6.037	8.296	7.235
6.944	10.685	6.928
10.796	3.981	5.541
10.037	4.185	6.016
10.389	4.667	6.163
11.667	3.722	4.762
11.519	4.296	5.325
11.759	11.222	5.520
12.833	10.796	4.839

HOM-7-3

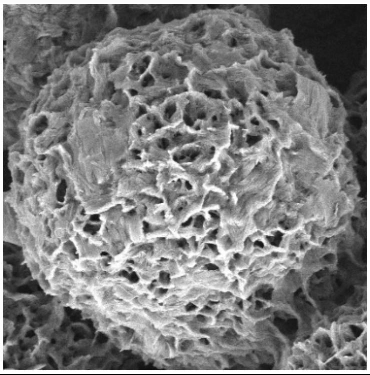

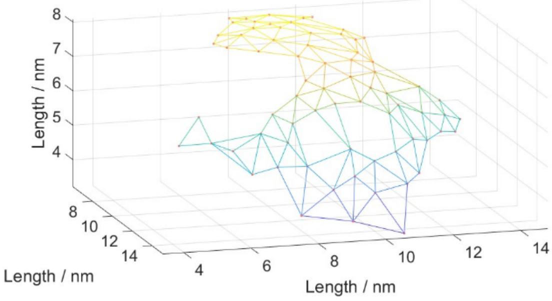

Average length =0.957±0.296μm

Modelling function: $\frac{(x-a)^2}{A}+\frac{(y-b)^2}{B}+\frac{z^2}{C}=1$ (a=8.70; b=7.83; A=C=8.09; B=7.56)

x	y	z
7.005	8.441	7.884
6.734	8.920	7.761
6.989	9.383	7.731
7.580	8.824	7.942
8.043	8.473	8.034
8.601	8.266	8.076
8.793	8.633	8.044
8.170	7.340	8.057
7.707	7.548	8.024
8.266	7.867	8.079
9.000	6.830	8.014
7.851	6.718	7.958
9.830	7.452	8.001
9.255	8.154	8.064
9.559	9.096	7.929
8.346	9.479	7.887
9.239	9.878	7.768
8.793	10.436	7.592
7.755	9.973	7.700
9.495	10.755	7.416
10.356	10.468	7.396
11.027	10.340	7.265
10.676	10.005	7.490
10.947	10.819	7.080
10.612	11.521	6.793
10.101	11.234	7.084
9.160	11.729	6.913
8.713	11.441	7.105
8.713	12.399	6.441
8.298	11.920	6.789
10.340	6.622	7.816
10.723	8.426	7.806
10.516	8.904	7.798
10.883	9.128	7.664
11.250	8.761	7.612
11.330	11.856	6.318
10.963	12.223	6.178
11.074	11.569	6.614
10.149	12.064	6.540
9.351	12.207	6.560
7.468	12.319	6.389
7.963	12.670	6.167
7.005	12.702	5.945
7.261	13.516	5.127
7.691	13.085	5.723
9.479	13.340	5.477
10.133	13.516	5.127
9.989	14.090	4.338
9.447	13.867	4.803
8.745	14.106	4.500
9.191	14.553	3.653
7.707	14.394	3.877
8.106	13.644	5.130
11.043	13.739	4.459
10.723	14.505	3.197
10.867	13.181	5.280
11.489	13.420	4.668
11.521	12.702	5.498
12.543	12.032	5.513
12.064	11.761	6.031
11.984	12.543	5.399
13.069	11.697	5.401
12.862	11.346	5.823
13.404	11.043	5.607
12.862	10.835	6.143
12.287	10.723	6.554
6.144	12.814	5.516
6.527	13.452	4.947
5.633	12.383	5.681
5.505	11.553	6.273
4.803	11.936	5.562

HOM-7-4	

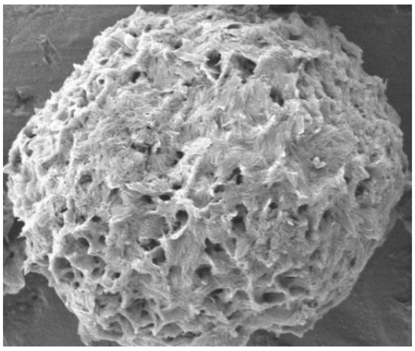

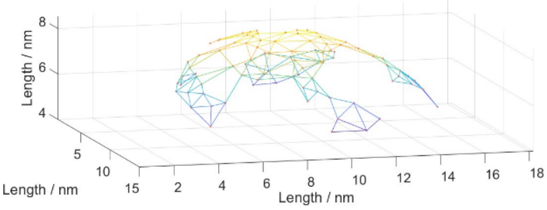

Average length =0.990±0.323μm

Modelling function: $\frac{(x-a)^2}{A}+\frac{(y-b)^2}{B}+\frac{z^2}{C}=1$ (a=8.80; b=7.51; A=C=8.56; B=7.11)

x	y	z
10.116	6.994	8.431
9.523	7.483	8.525
10.343	7.709	8.412
9.366	8.198	8.496
8.860	8.023	8.533
7.709	7.674	8.483
7.273	7.517	8.418
7.395	8.006	8.418
6.523	8.198	8.205
6.924	8.651	8.233
7.552	8.703	8.341
8.110	9.052	8.323
7.256	9.279	8.141
7.622	9.907	7.967
5.773	8.424	7.926
6.767	10.029	7.737
5.965	10.047	7.472
6.174	10.727	7.162
7.081	11.023	7.235
6.767	11.215	7.011
8.599	10.587	7.709
9.872	10.674	7.585
8.616	9.994	8.013
10.029	9.994	7.921
10.448	9.733	7.957
10.395	9.227	8.147
9.680	9.087	8.295
10.326	8.442	8.343
11.477	8.355	8.062

11.076	9.000	8.049
11.965	8.721	7.813
9.140	11.547	7.032
8.843	11.041	7.424
8.302	11.424	7.123
8.634	11.930	6.697
8.703	12.436	6.165
9.227	12.087	6.530
9.733	12.279	6.273
7.378	6.331	8.317
7.587	6.837	8.431
7.971	6.471	8.423
5.477	2.895	5.596
5.459	3.314	6.045
6.122	3.035	6.085
9.000	1.936	5.307
8.424	2.791	6.388
9.558	2.477	5.995
9.157	3.331	6.913
10.343	3.279	6.700
10.884	2.564	5.782
10.012	2.076	5.382
11.616	3.506	6.485
11.250	4.064	7.071
12.000	4.448	7.027
12.070	4.012	6.692
13.116	8.477	7.294
12.576	8.948	7.479
13.134	9.174	7.099
13.081	10.343	6.575

14.198	9.349	6.257
13.866	8.651	6.755
13.552	9.593	6.657
14.703	7.919	6.173
14.983	7.517	5.913
14.599	7.256	6.283
15.750	7.866	4.970
14.983	8.320	5.832
6.453	6.000	8.025
6.035	5.843	7.844
6.244	6.471	8.069
5.006	7.849	7.658
4.500	7.866	7.384
4.395	8.547	7.227
3.733	9.017	6.651
3.558	9.419	6.359
4.465	9.488	6.981
4.535	10.081	6.740
4.483	10.570	6.402
3.942	9.994	6.376
4.657	11.285	5.947
4.465	11.895	5.151
5.233	11.512	6.104
3.890	10.727	5.838
9.558	13.291	4.917
9.924	12.924	5.426
11.215	12.994	4.875
11.948	12.453	5.278
10.814	12.297	5.993

HOM-7-5

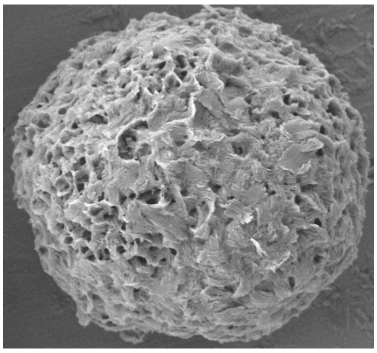

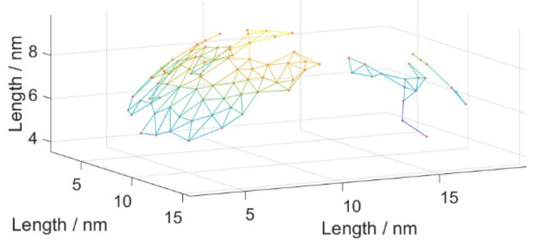

Average length =1.05±0.2635μm

Modelling function: $\frac{(x-a)^2}{A}+\frac{(y-b)^2}{B}+\frac{z^2}{C}=1$ (a=10.6; b=9.75; A=C=10.0; B=9.11)

x	y	z
8.899	10.346	9.856
7.686	10.984	9.489
8.835	11.282	9.721
6.793	10.707	9.204
6.410	11.324	8.929
7.048	12.197	8.970
6.324	12.388	8.577
6.835	12.856	8.628
7.005	13.601	8.332
6.410	13.261	8.233
6.473	14.090	7.773
7.410	14.176	8.150
8.133	14.410	8.245
7.729	14.750	7.862
8.367	13.473	8.867
7.516	13.090	8.794
8.963	12.920	9.252
9.154	13.686	8.922
9.686	14.027	8.803
8.878	14.133	8.614
9.707	11.303	9.839
8.410	9.367	9.770
8.154	9.963	9.715
5.665	10.431	8.679
5.048	11.218	8.171
5.112	12.261	7.902
5.835	12.941	8.074
5.154	13.261	7.456
5.835	13.686	7.665
5.218	13.856	7.127
5.495	14.452	6.881
4.559	14.218	6.281
4.452	13.452	6.762
4.495	12.495	7.331
4.580	11.686	7.706
4.239	10.644	7.662
5.069	10.027	8.337
5.750	9.346	8.748
5.176	8.963	8.369
4.154	9.622	7.653
4.133	8.750	7.556
4.686	8.388	7.934
3.303	9.644	6.842
3.048	10.495	6.508
3.495	11.154	6.871
3.495	12.069	6.564
3.750	12.814	6.465
3.324	13.303	5.641
2.984	12.133	5.929
2.367	10.410	5.630
2.601	11.473	5.695
3.388	8.431	6.779
3.090	7.793	6.246
2.835	8.601	6.176
3.963	6.601	6.636
4.622	7.303	7.561
5.899	6.793	8.219
5.771	5.984	7.728
4.963	6.452	7.431
7.750	6.516	8.921
7.133	6.878	8.850
8.154	7.154	9.288
7.580	5.452	8.297
7.771	4.622	7.777
7.154	4.941	7.773
6.516	4.793	7.336
7.090	4.154	7.073
8.644	4.303	7.787
8.495	5.090	8.347
9.367	4.601	8.174
10.090	4.452	8.139
10.537	3.835	7.625
9.644	3.707	7.437
9.282	3.112	6.731
8.665	3.324	6.828
7.239	2.984	5.790
6.516	3.367	5.848
17.920	8.048	6.634
17.261	8.388	7.379
18.005	8.580	6.680
17.941	6.665	5.973
17.750	7.069	6.423
18.261	7.154	5.854
16.835	5.559	6.391
16.324	5.835	7.045
17.027	6.090	6.595
16.112	4.963	6.542
15.644	5.537	7.347
18.431	5.495	4.226
19.239	6.346	3.538
18.176	6.090	5.242
16.729	10.559	7.916

16.133	10.941	8.285
16.601	11.388	7.858

17.814	11.963	6.567
18.005	12.537	6.071

17.410	12.303	6.840

HOM-8-1

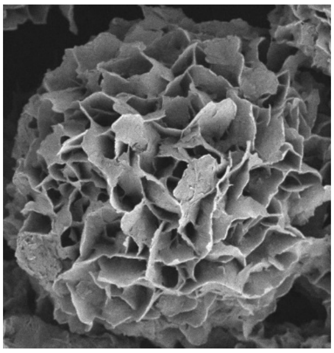

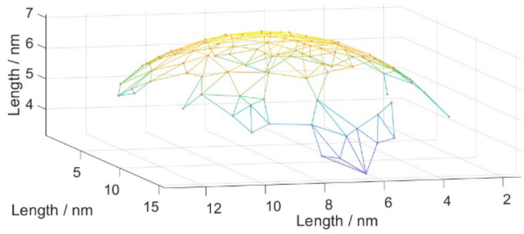

Average length =1.03±0.322μm

Modelling function: $\frac{(x-a)^2}{A}+\frac{(y-b)^2}{B}+\frac{z^2}{C}=1$ (a=7.40; b=7.78; A=C=7.12; B=7.53)

x	y	z
6.674	5.367	6.701
8.064	5.642	6.791
7.817	6.537	7.005
7.885	7.060	7.066
6.688	6.798	7.018
7.528	7.651	7.112
7.266	7.885	7.112
7.472	8.573	7.074
8.106	8.009	7.076
8.395	7.390	7.036
6.647	7.720	7.074
6.179	6.963	6.966
5.573	6.372	6.746
5.601	7.252	6.865
5.188	7.046	6.726
5.092	6.096	6.539
4.225	5.917	6.118
4.899	5.615	6.338
4.486	6.936	6.440
4.968	8.271	6.668
5.518	8.436	6.832
4.954	9.096	6.562
5.766	9.372	6.757
6.385	8.862	6.966
8.050	9.564	6.881
7.225	9.798	6.851
6.977	9.000	7.007
9.771	7.183	6.686
8.972	6.895	6.889
8.546	6.537	6.924

9.165	6.372	6.764
10.335	6.647	6.394
10.761	6.578	6.170
10.087	5.560	6.247
8.739	5.450	6.633
9.096	4.734	6.284
8.587	3.798	5.923
8.326	3.083	5.486
7.142	4.197	6.254
6.950	5.216	6.675
8.422	4.376	6.265
5.972	4.128	6.057
5.408	4.321	5.998
6.234	5.050	6.528
7.514	3.509	5.860
7.073	3.275	5.693
6.853	2.725	5.247
7.569	2.972	5.475
8.711	2.780	5.159
8.546	1.913	4.314
9.812	8.009	6.691
9.688	8.408	6.711
8.862	10.101	6.607
8.670	10.885	6.354
7.913	10.349	6.666
9.427	10.954	6.124
7.005	10.514	6.615
6.812	11.532	6.137
7.830	11.725	6.042
8.601	11.972	5.784
8.587	12.509	5.404

8.147	12.482	5.502
7.803	12.839	5.249
6.757	12.495	5.505
10.005	10.610	6.056
10.266	9.812	6.222
10.789	8.573	6.212
11.147	7.404	6.040
5.601	10.486	6.388
6.151	11.050	6.283
6.138	11.463	6.072
5.546	11.436	5.933
5.009	10.706	6.100
4.569	10.087	6.149
4.170	8.794	6.264
4.101	7.995	6.298
3.564	7.982	5.986
3.537	8.463	5.937
2.945	7.266	5.523
3.894	7.142	6.160
3.550	6.550	5.867
2.752	6.564	5.260
3.771	6.000	5.883
4.101	5.408	5.891
5.092	4.830	6.126
4.032	4.665	5.532
11.986	7.252	5.420
12.743	7.638	4.700
11.794	9.000	5.477
11.876	9.468	5.297
11.450	9.716	5.557
10.761	10.830	5.569

10.872	11.477	5.133
11.986	10.101	4.979
12.633	9.633	4.493
12.564	9.028	4.752
13.445	7.486	3.747

13.445	6.881	3.661
12.936	6.523	4.314
12.826	5.450	4.047
12.661	6.096	4.523
13.569	6.028	3.142

11.229	4.555	5.169
11.972	5.202	4.882
12.440	4.775	4.148
4.528	2.931	4.624
5.573	3.317	5.432

HOM-8-2	
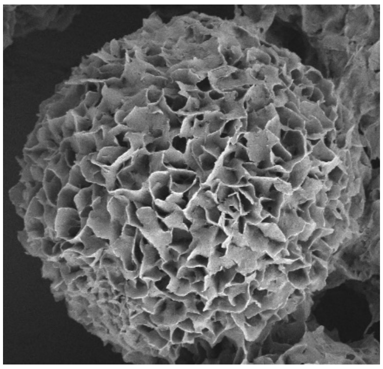	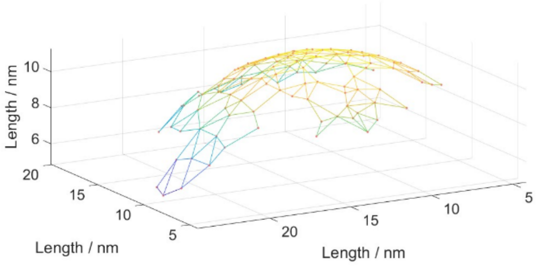 Average length =1.43±0.383μm

Modelling function: $\frac{(x-a)^2}{A}+\frac{(y-b)^2}{B}+\frac{z^2}{C}=1$ (a=12.38; b=11.6; A=C=11.3; B=10.9)

x	y	z
6.674	5.367	6.701
8.064	5.642	6.791
7.817	6.537	7.005
7.885	7.060	7.066
6.688	6.798	7.018
7.528	7.651	7.112
7.266	7.885	7.112
7.472	8.573	7.074
8.106	8.009	7.076
8.395	7.390	7.036
6.647	7.720	7.074
6.179	6.963	6.966
5.573	6.372	6.746
5.601	7.252	6.865
5.188	7.046	6.726
5.092	6.096	6.539
4.225	5.917	6.118
4.899	5.615	6.338
4.486	6.936	6.440
4.968	8.271	6.668
5.518	8.436	6.832
4.954	9.096	6.562
5.766	9.372	6.757
6.385	8.862	6.966
8.050	9.564	6.881
7.225	9.798	6.851
6.977	9.000	7.007
9.771	7.183	6.686
8.972	6.895	6.889
8.546	6.537	6.924
9.165	6.372	6.764
10.335	6.647	6.394
10.761	6.578	6.170
10.087	5.560	6.247
8.739	5.450	6.633
9.096	4.734	6.284
8.587	3.798	5.923
8.326	3.083	5.486
7.142	4.197	6.254
6.950	5.216	6.675
8.422	4.376	6.265
5.972	4.128	6.057
5.408	4.321	5.998
6.234	5.050	6.528
7.514	3.509	5.860
7.073	3.275	5.693
6.853	2.725	5.247
7.569	2.972	5.475
8.711	2.780	5.159
8.546	1.913	4.314
9.812	8.009	6.691
9.688	8.408	6.711
8.862	10.101	6.607
8.670	10.885	6.354
7.913	10.349	6.666
9.427	10.954	6.124
7.005	10.514	6.615
6.812	11.532	6.137
7.830	11.725	6.042
8.601	11.972	5.784
8.587	12.509	5.404
8.147	12.482	5.502
7.803	12.839	5.249
6.757	12.495	5.505
10.005	10.610	6.056
10.266	9.812	6.222
10.789	8.573	6.212
11.147	7.404	6.040
5.601	10.486	6.388
6.151	11.050	6.283
6.138	11.463	6.072
5.546	11.436	5.933
5.009	10.706	6.100
4.569	10.087	6.149
4.170	8.794	6.264
4.101	7.995	6.298
3.564	7.982	5.986
3.537	8.463	5.937
2.945	7.266	5.523
3.894	7.142	6.160
3.550	6.550	5.867
2.752	6.564	5.260
3.771	6.000	5.883
4.101	5.408	5.891
5.092	4.830	6.126
4.032	4.665	5.532
11.986	7.252	5.420
12.743	7.638	4.700
11.794	9.000	5.477
11.876	9.468	5.297
11.450	9.716	5.557
10.761	10.830	5.569

10.872	11.477	5.133
11.986	10.101	4.979
12.633	9.633	4.493
12.564	9.028	4.752
13.445	7.486	3.747

13.445	6.881	3.661
12.936	6.523	4.314
12.826	5.450	4.047
12.661	6.096	4.523
13.569	6.028	3.142

11.229	4.555	5.169
11.972	5.202	4.882
12.440	4.775	4.148
4.528	2.931	4.624
5.573	3.317	5.432

HOM-8-3

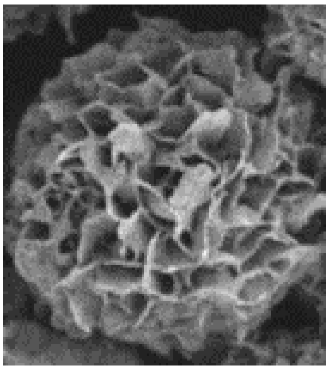

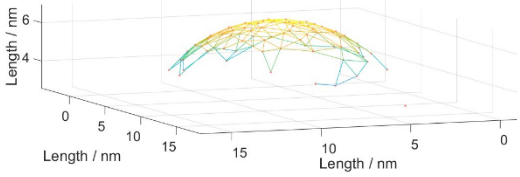

Average length =1.30±0.352μm

Modelling function: $\frac{(x-a)^2}{A}+\frac{(y-b)^2}{B}+\frac{z^2}{C}=1$ (a=7.20; b=7.98; A=C=6.96; B=7.32)

x	y	z
6.630	5.685	6.586
7.963	5.815	6.607
9.185	5.278	6.159
8.259	4.648	6.107
7.074	4.667	6.206
8.463	3.241	5.151
8.611	2.407	4.283
7.315	3.574	5.556
10.222	4.648	5.413
10.556	4.278	4.982
10.519	2.148	2.580
10.278	7.926	6.248
8.963	6.704	6.627
9.778	7.370	6.444
7.981	7.037	6.861
7.111	6.315	6.779
6.889	7.370	6.932
6.222	8.093	6.893
7.056	8.704	6.927
7.593	8.315	6.945
7.519	9.185	6.861
7.944	7.611	6.915
9.556	8.556	6.531
8.389	9.926	6.607
9.111	10.685	6.183
8.870	11.630	5.800
7.981	10.870	6.350
7.222	10.481	6.544
6.389	9.444	6.774
5.648	10.056	6.493
4.944	8.630	6.557
5.185	6.759	6.562
5.056	7.444	6.604
4.093	7.611	6.219
4.148	6.074	5.987
4.870	6.185	6.333
5.481	5.611	6.358
3.444	4.926	5.088
4.667	3.815	5.130
5.056	4.907	5.942
6.444	4.556	6.105
6.704	3.204	5.249
6.000	2.704	4.668
11.148	7.556	5.724
10.407	5.685	5.783
12.000	5.444	4.433
11.370	5.963	5.238
3.241	6.741	5.602
2.463	7.444	5.074
3.167	7.722	5.668
3.537	8.611	5.889
4.167	8.593	6.239
4.074	9.815	5.970
5.037	9.685	6.415
6.389	11.500	6.050
5.630	11.093	6.102
5.204	11.778	5.606
6.000	12.037	5.668
6.944	12.111	5.741
3.685	10.500	5.509
2.796	9.778	5.112
2.185	8.296	4.817
1.741	8.648	4.270
3.426	11.741	4.626
2.463	11.333	3.978
4.407	11.889	5.179
4.593	10.852	5.848
8.037	12.241	5.599
10.556	10.130	5.750
9.778	9.722	6.254
4.056	12.778	4.210
6.296	13.000	4.984
7.741	13.648	4.370
11.778	10.167	4.820
10.722	9.204	5.895
12.741	8.056	4.221
12.759	6.944	4.080

HOM-8-4	
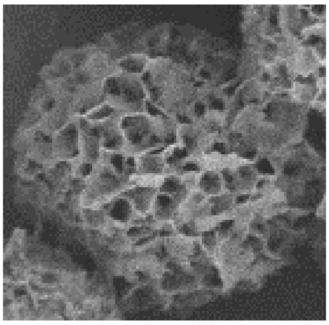	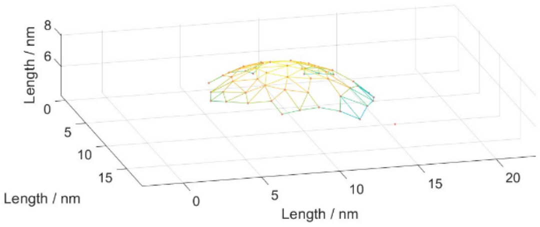 Average length =1.48±0.319μm

Modelling function: $\frac{(x-a)^2}{A}+\frac{(y-b)^2}{B}+\frac{z^2}{C}=1$ (a=8.87; b=9.18; A=C=8.13; B=8.14)

x	y	z
7.131	6.237	7.374
6.632	7.268	7.576
8.230	6.357	7.594
7.302	8.574	7.952
8.471	7.646	7.971
10.309	8.076	7.921
9.175	9.158	8.121
7.646	9.914	8.002
5.756	8.591	7.485
6.942	10.412	7.800
4.742	11.151	6.722
4.845	10.017	7.013
4.330	9.021	6.741
4.055	10.034	6.495
5.636	9.381	7.455
7.182	11.357	7.649
7.199	12.835	7.069
5.842	12.285	6.878

8.230	12.182	7.528
8.729	10.911	7.941
10.155	11.100	7.793
9.605	12.560	7.357
9.966	9.742	8.033
11.014	8.763	7.827
11.529	9.605	7.667
12.371	7.955	7.229
9.931	7.079	7.778
8.540	5.567	7.272
11.186	6.718	7.389
10.619	6.014	7.277
11.357	5.172	6.617
11.959	6.186	6.893
12.904	6.186	6.386
13.471	8.849	6.688
14.605	8.265	5.681
14.639	9.364	5.717
13.557	9.639	6.621

14.261	10.653	5.898
12.732	10.945	6.929
11.357	10.722	7.582
11.735	12.388	6.897
13.540	12.010	6.019
15.842	10.017	4.086
12.234	5.172	6.216
11.512	4.364	5.989
8.385	3.832	6.102
7.749	4.416	6.490
6.409	5.498	6.816
4.914	6.271	6.478
5.636	6.976	7.124
5.945	11.340	7.272
7.595	13.900	6.499
8.746	13.849	6.657
10.017	13.660	6.688
11.375	13.625	6.330
12.663	13.574	5.692

HOM-8-5

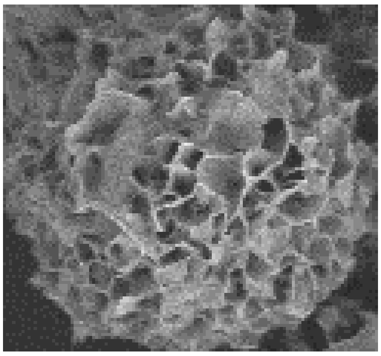

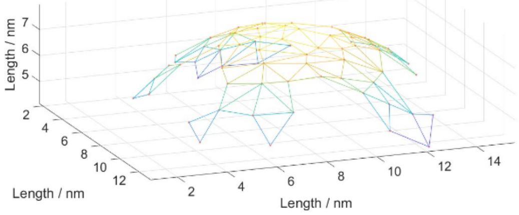

Average length =1.31±0.326μm

Modelling function: $\frac{(x-a)^2}{A}+\frac{(y-b)^2}{B}+\frac{z^2}{C}=1$ (a=8.40; b=7.80; A=C=7.85; B=7.58)

x	y	z
12.148	5.000	6.261
11.306	6.065	7.070
12.216	6.649	6.759
12.612	6.065	6.379
10.704	6.753	7.428
10.997	7.715	7.410
11.701	7.595	7.122
12.423	8.471	6.708
13.643	7.096	5.801
12.698	7.440	6.562
11.753	8.986	6.994
10.842	8.402	7.437
10.653	9.296	7.361
9.948	9.759	7.426
9.897	8.643	7.659
8.986	9.777	7.558
9.742	10.515	7.207
8.918	7.938	7.834
9.055	6.838	7.761
8.591	7.371	7.837
7.560	7.148	7.777
8.213	6.237	7.681
7.904	8.832	7.763
7.268	8.196	7.759
6.838	9.227	7.551
5.808	7.663	7.410
6.649	6.701	7.569
7.388	5.636	7.456
8.454	5.344	7.428
8.368	10.172	7.458
8.162	10.670	7.263
6.667	10.120	7.271
7.165	11.340	6.832
8.471	11.392	6.914
9.227	11.512	6.796
10.533	11.512	6.506
9.897	12.079	6.306
10.859	12.285	5.833
11.168	10.790	6.664
12.955	9.674	6.096
4.227	6.581	6.529
3.076	7.491	5.761
4.399	7.320	6.737
5.292	9.347	7.030
6.392	8.316	7.572
7.784	2.732	5.804
7.869	4.021	6.785
9.055	3.058	6.089
9.570	2.010	4.928
10.447	3.093	5.803
5.498	4.003	6.143
6.890	3.368	6.186
6.770	4.330	6.787
7.285	1.684	4.498
6.873	2.457	5.353
8.162	1.907	4.930
10.533	13.213	5.065
12.165	13.041	4.243
12.852	11.684	5.065
11.804	12.268	5.353
5.498	12.010	5.848
6.924	12.526	5.958
5.773	13.110	4.947
4.502	11.065	5.916
5.155	10.275	6.674
6.237	11.031	6.765
3.574	11.495	4.867
3.488	10.567	5.412
2.835	6.478	5.365
3.608	6.684	6.110
5.808	3.144	5.625

HOM-9-1

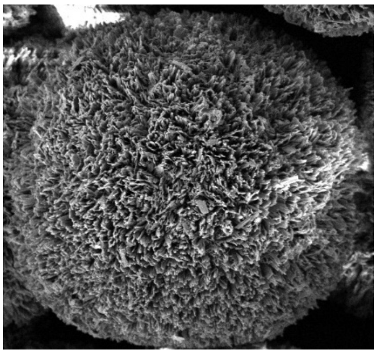

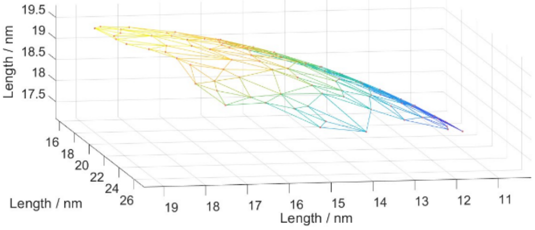

Average length =0.958±0.359μm

Modelling function: $\frac{(x-a)^2}{A}+\frac{(y-b)^2}{B}+\frac{z^2}{C}=1$ (a=20.4; b=20.0; A=C=19.8; B=19.1)

x	y	z
19.41	13.85	18.73
19.16	13.34	18.52
19.10	13.76	18.67
19.89	12.98	18.41
20.34	13.01	18.43
19.29	12.40	18.13
18.83	13.22	18.45
18.35	13.04	18.32
17.81	13.25	18.34
17.99	12.73	18.15
18.53	12.13	17.94
19.07	11.86	17.86
19.56	11.62	17.77
20.58	11.86	17.91
21.22	12.22	18.07
20.89	12.95	18.40
21.28	13.73	18.69
21.85	13.91	18.72
20.46	14.61	19.00
20.04	14.30	18.90
20.31	14.94	19.10
20.13	15.72	19.31
19.74	15.75	19.30
20.79	16.27	19.43
20.58	16.42	19.46
21.34	15.84	19.32
21.94	15.75	19.26
22.03	15.09	19.08
22.64	15.57	19.14
22.82	15.00	18.97
23.21	14.73	18.83
23.72	14.58	18.70
23.39	14.00	18.57
22.82	13.94	18.63
22.30	13.19	18.41
22.46	12.70	18.19
22.03	13.10	18.39
22.09	11.89	17.85
21.97	11.25	17.53
21.49	11.19	17.53
21.19	11.50	17.71
20.64	11.25	17.60
20.16	11.25	17.60
19.47	10.86	17.36
19.38	10.44	17.10
18.80	11.41	17.61
18.20	11.44	17.56
17.87	11.83	17.71
17.32	12.04	17.73
17.32	13.37	18.31
17.50	13.85	18.53
17.32	14.27	18.64
17.59	14.58	18.78
18.23	15.24	19.06
18.65	14.82	18.98
19.95	16.36	19.44
19.65	16.66	19.49
19.13	16.24	19.38
18.56	16.09	19.30
20.55	17.23	19.61
21.46	17.29	19.59
21.58	16.87	19.51
22.43	16.51	19.38
23.51	16.90	19.31
23.96	16.60	19.17
24.63	15.72	18.84
25.02	15.15	18.60
25.41	15.54	18.61
25.44	16.24	18.76
25.26	16.57	18.88
25.57	13.85	18.03
25.72	14.85	18.32
26.17	15.06	18.25
23.69	13.40	18.29
23.42	12.55	17.99
20.13	17.53	19.65
19.50	17.53	19.63
18.41	17.14	19.49
17.78	16.05	19.21
17.50	15.75	19.10
16.87	15.72	18.98
16.69	15.03	18.76
16.69	14.52	18.60
20.49	18.08	19.72
21.79	17.96	19.66
22.52	18.11	19.61
23.03	17.93	19.53
23.48	17.69	19.43
24.87	17.81	19.18
24.54	17.29	19.18
25.17	17.35	19.04
22.33	18.47	19.66

21.88	18.92	19.73
21.34	19.07	19.77
20.89	19.04	19.79

20.37	18.80	19.78
18.35	18.23	19.62
18.89	19.01	19.73

19.50	19.26	19.78
19.59	18.65	19.75
25.99	17.29	18.81

HOM-9-2	
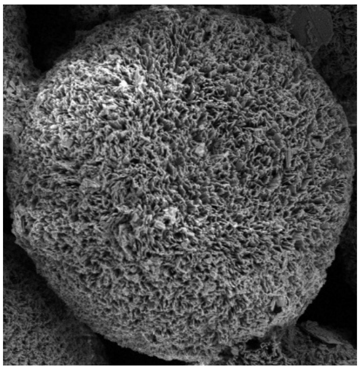	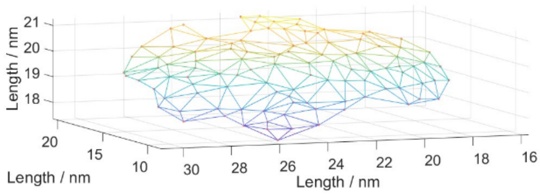 Average length =1.12±0.377μm

Modelling function: $\frac{(x-a)^2}{A}+\frac{(y-b)^2}{B}+\frac{z^2}{C}=1$ (a=22.1; b=22.3; A=C=21.6; B=21.8)

x	y	z
18.86	14.63	19.92
19.07	13.84	19.63
19.10	13.39	19.44
19.72	13.56	19.60
20.48	13.25	19.54
21.24	13.35	19.64
20.96	12.60	19.27
21.79	12.56	19.28
22.48	12.67	19.33
23.03	13.08	19.51
22.79	13.91	19.88
21.75	13.97	19.92
22.03	14.49	20.13
21.03	14.46	20.09
20.55	14.63	20.12
21.38	15.52	20.48
22.31	15.52	20.49
22.89	15.35	20.42
23.27	14.90	20.25
23.68	14.77	20.17
23.96	14.63	20.10
24.13	14.11	19.88
25.16	14.01	19.70
25.20	14.87	20.03
24.82	15.35	20.26
25.30	15.83	20.34
25.09	16.28	20.51
24.68	16.38	20.60
23.99	17.14	20.87
23.23	17.21	20.94

22.65	17.00	20.91
21.93	16.66	20.83
21.48	16.45	20.76
21.07	16.83	20.85
21.96	17.45	21.02
22.75	17.90	21.11
23.48	18.04	21.11
25.82	17.18	20.63
26.61	16.28	20.23
26.85	15.56	19.95
27.06	15.08	19.73
26.37	14.22	19.57
25.78	13.84	19.53
26.40	13.29	19.15
24.89	12.98	19.29
24.71	12.32	18.99
24.03	12.29	19.05
24.34	11.94	18.83
24.30	11.56	18.63
20.27	12.12	18.97
19.65	12.25	18.97
18.52	12.77	19.05
18.42	14.01	19.59
17.80	14.53	19.67
18.38	15.59	20.17
19.31	15.42	20.27
19.90	15.94	20.50
21.62	18.07	21.15
23.03	18.72	21.25
23.75	18.90	21.24
27.16	17.21	20.35

27.95	16.76	20.02
28.26	15.97	19.70
27.88	15.97	19.81
28.12	15.08	19.44
27.54	14.63	19.44
27.54	14.08	19.22
27.57	13.46	18.93
27.19	12.22	18.42
26.37	11.81	18.41
25.58	11.43	18.36
24.89	11.01	18.23
25.64	10.84	17.99
24.85	10.26	17.75
25.58	9.88	17.36
26.57	10.98	17.87
25.78	10.50	17.74
27.09	11.74	18.18
28.60	12.22	17.97
28.54	12.80	18.31
29.16	13.53	18.44
28.78	13.87	18.73
28.91	14.66	19.01
29.19	15.63	19.27
29.47	16.32	19.39
29.43	16.62	19.49
28.74	17.59	19.99
28.12	18.17	20.31
26.57	19.10	20.86
26.02	19.17	20.98
19.17	16.90	20.68
19.55	16.59	20.65

17.97	16.59	20.39
17.14	15.66	19.93
17.14	14.46	19.49
17.18	13.42	19.06
17.86	13.15	19.10

18.17	11.87	18.51
19.03	11.67	18.56
19.65	11.25	18.42
23.75	10.22	17.86
23.58	11.05	18.40

23.79	11.43	18.60
21.34	11.56	18.74
22.24	11.32	18.62
22.44	10.88	18.35

HOM-9-3

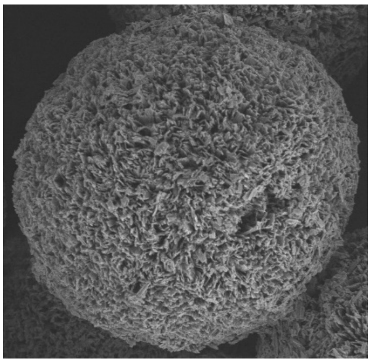

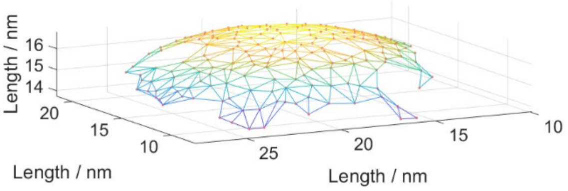

Average length =1.06±0.363μm

Modelling function: $\frac{(x-a)^2}{A} + \frac{(y-b)^2}{B} + \frac{z^2}{C} = 1$ (a=17.9; b=17.3; A=C=16.8; B=16.4)

x	y	z
17.37	15.16	16.66
17.89	14.50	16.56
18.49	14.48	16.55
17.73	13.83	16.43
17.79	13.49	16.35
18.46	13.46	16.33
19.11	13.88	16.39
19.64	14.19	16.41
19.84	14.50	16.45
19.04	15.08	16.62
20.21	14.79	16.45
20.23	15.49	16.54
19.27	15.83	16.69
18.54	16.46	16.78
17.34	15.73	16.72
16.51	15.00	16.58
15.75	15.08	16.51
16.15	15.75	16.64
16.54	16.22	16.72
16.90	16.67	16.77
17.16	17.37	16.79
18.33	17.19	16.80
18.67	17.58	16.79
18.18	18.18	16.79
18.54	18.54	16.75
19.04	18.88	16.70
19.90	18.25	16.66
20.21	17.76	16.65
20.60	17.16	16.59
19.37	16.87	16.74

20.03	16.59	16.66
20.73	16.46	16.55
21.07	15.96	16.45
21.38	15.81	16.37
21.09	15.08	16.35
21.20	14.56	16.24
20.39	13.91	16.26
19.97	13.38	16.19
19.30	13.07	16.18
19.56	12.79	16.07
21.07	13.07	15.93
20.83	13.36	16.05
21.43	13.59	15.99
21.38	13.96	16.08
22.16	14.37	15.98
22.50	13.88	15.78
22.71	14.19	15.79
22.27	12.99	15.62
21.22	11.95	15.54
20.34	11.51	15.54
19.82	11.67	15.67
19.24	11.98	15.84
18.31	11.85	15.85
18.02	12.24	15.99
17.24	12.42	16.03
16.98	12.06	15.90
16.15	12.40	15.94
16.72	12.76	16.11
15.68	13.46	16.19
14.74	13.44	16.03
14.95	12.71	15.86

15.18	12.34	15.79
16.07	11.51	15.62
17.60	10.29	15.19
18.10	10.52	15.30
18.98	10.52	15.27
19.84	10.81	15.31
21.07	11.12	15.24
21.74	11.20	15.12
22.55	11.82	15.15
23.62	11.95	14.83
23.93	12.00	14.72
23.83	13.41	15.22
23.20	13.02	15.34
23.98	14.14	15.33
23.41	14.30	15.58
23.02	14.77	15.80
23.62	14.95	15.62
23.62	15.49	15.70
22.79	15.68	16.00
22.66	16.30	16.09
23.59	16.61	15.80
24.37	16.30	15.48
24.58	15.65	15.33
25.05	14.95	15.02
25.08	14.37	14.90
24.61	14.11	15.06
25.13	13.80	14.75
24.84	13.23	14.73
25.99	14.11	14.37
25.89	14.63	14.54
25.91	14.97	14.58

25.65	15.44	14.79
25.86	15.86	14.73
25.34	16.48	15.05
26.02	16.67	14.71
25.47	17.21	15.01
25.21	17.86	15.13
24.53	17.60	15.45
21.85	17.55	16.34
21.07	17.99	16.50
21.09	18.44	16.47
21.48	19.04	16.33
20.86	18.80	16.48
19.71	19.43	16.57
21.93	19.64	16.15
20.73	19.95	16.35
17.99	18.93	16.73
18.93	19.97	16.56
18.62	20.21	16.53
17.00	18.78	16.72
16.67	17.86	16.76
16.12	18.57	16.67
15.96	16.80	16.69
15.29	16.30	16.57
15.16	15.52	16.48
14.79	15.31	16.39
14.30	14.27	16.12
14.06	15.05	16.20
13.49	14.22	15.91

13.85	15.68	16.23
14.22	16.33	16.37
14.45	16.80	16.44
14.92	17.60	16.54
13.83	17.24	16.31
13.46	16.54	16.19
12.68	15.94	15.92
13.02	15.44	15.97
12.66	15.10	15.81
12.47	14.50	15.65
16.87	9.48	14.74
16.80	10.05	15.04
18.44	9.19	14.60
16.56	8.12	13.87
16.35	8.88	14.34
15.68	8.23	13.83
14.43	10.34	14.82
13.07	11.64	15.02
13.44	12.03	15.28
13.23	12.99	15.53
13.83	13.31	15.78
23.46	10.86	14.42
22.79	10.47	14.48
24.22	10.55	13.96
24.53	11.17	14.11
23.72	9.74	13.73
23.10	9.43	13.80
21.85	10.49	14.78

21.54	10.29	14.75
21.15	9.97	14.69
20.39	10.13	14.91
19.66	9.45	14.66
20.89	9.17	14.29
18.88	21.07	16.34
19.92	20.68	16.33
20.16	20.99	16.23
21.09	20.57	16.17
21.17	21.04	16.04
21.69	20.94	15.95
22.53	20.94	15.73
22.99	19.97	15.79
23.36	20.52	15.56
24.69	19.61	15.20
25.16	19.40	15.02
11.59	17.29	15.58
11.67	16.48	15.59
12.42	17.00	15.89
13.18	17.08	16.13
13.67	18.36	16.23
12.53	18.36	15.89
12.27	17.92	15.83
14.17	19.27	16.27
14.97	19.22	16.44
15.10	18.44	16.54

HOM-9-4

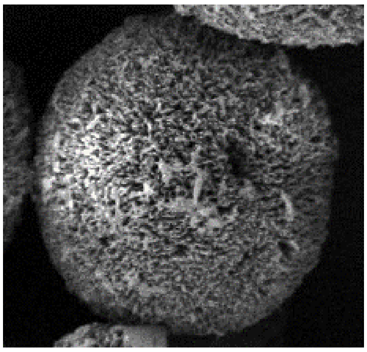

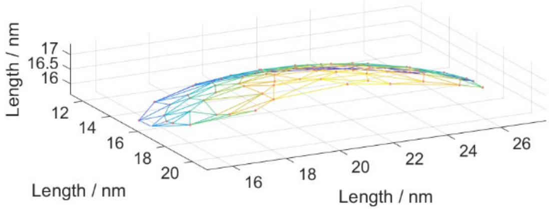

Average length =1.27±0.447μm

Modelling function: $\frac{(x-a)^2}{A}+\frac{(y-b)^2}{B}+\frac{z^2}{C}=1$ (a=21.1; b=19.9; A=C=17.4; B=18.6)

x	y	z
21.57	17.75	17.25
22.22	17.70	17.22
22.31	18.49	17.28
22.83	17.61	17.16
23.20	18.26	17.18
23.20	17.00	17.04
21.20	17.05	17.17
21.52	16.21	17.03
21.06	15.75	16.94
19.94	16.44	17.03
19.89	15.93	16.93
21.34	15.23	16.82
20.40	15.23	16.81
20.08	14.86	16.70
19.33	15.33	16.75
22.55	15.47	16.81
22.69	16.40	16.99
23.34	15.47	16.73
24.83	16.72	16.71
24.69	17.56	16.86
24.41	18.59	17.01
26.18	18.21	16.54
25.62	18.87	16.75
25.95	19.85	16.69
25.02	19.98	16.93
23.76	19.80	17.17
22.41	20.03	17.33
21.89	19.43	17.35
21.06	19.57	17.37
20.64	18.45	17.32
20.12	17.94	17.25
19.43	17.47	17.15
19.15	17.94	17.17
18.54	16.96	16.97
18.68	16.35	16.89
17.70	16.54	16.75
18.26	15.42	16.62
18.63	14.49	16.44
19.29	13.84	16.33
20.64	14.12	16.51
20.92	13.46	16.30
21.76	14.39	16.59
23.01	13.84	16.32
22.64	13.56	16.26
24.13	15.05	16.50
25.34	15.51	16.35
26.46	15.14	15.92
26.32	16.30	16.23
27.67	15.79	15.62
25.34	13.93	15.90
26.27	13.93	15.62
24.46	14.02	16.14
24.46	12.76	15.69
23.67	12.72	15.82
22.45	12.44	15.86
21.71	12.16	15.79
20.12	12.90	16.07
19.01	12.58	15.84
18.73	13.09	16.00
18.40	13.51	16.09
16.77	13.93	15.88
17.28	15.33	16.41
17.05	15.93	16.49
16.44	15.65	16.26
17.05	16.96	16.67
16.26	17.47	16.53
18.45	18.49	17.12
18.40	19.38	17.16
17.52	19.80	17.00
16.82	19.29	16.83
16.54	18.87	16.74
15.65	18.68	16.46
15.47	17.80	16.32
15.47	17.33	16.26
15.05	17.05	16.07
15.75	16.63	16.25
15.28	15.70	15.90
21.57	17.75	17.25
22.22	17.70	17.22
22.31	18.49	17.28
22.83	17.61	17.16
23.20	18.26	17.18
23.20	17.00	17.04
21.20	17.05	17.17
21.52	16.21	17.03
21.06	15.75	16.94
19.94	16.44	17.03
19.89	15.93	16.93
21.34	15.23	16.82
20.40	15.23	16.81
20.08	14.86	16.70
19.33	15.33	16.75

22.55	15.47	16.81
22.69	16.40	16.99
23.34	15.47	16.73
24.83	16.72	16.71
24.69	17.56	16.86
24.41	18.59	17.01
26.18	18.21	16.54
25.62	18.87	16.75
25.95	19.85	16.69
25.02	19.98	16.93
23.76	19.80	17.17
22.41	20.03	17.33
21.89	19.43	17.35
21.06	19.57	17.37
20.64	18.45	17.32
20.12	17.94	17.25

19.43	17.47	17.15
19.15	17.94	17.17
18.54	16.96	16.97
18.68	16.35	16.89
17.70	16.54	16.75
18.26	15.42	16.62
18.63	14.49	16.44
19.29	13.84	16.33
20.64	14.12	16.51
20.92	13.46	16.30
21.76	14.39	16.59
23.01	13.84	16.32
22.64	13.56	16.26
24.13	15.05	16.50
25.34	15.51	16.35
26.46	15.14	15.92

26.32	16.30	16.23
27.67	15.79	15.62
25.34	13.93	15.90
26.27	13.93	15.62
24.46	14.02	16.14
24.46	12.76	15.69
23.67	12.72	15.82
22.45	12.44	15.86
21.71	12.16	15.79
20.12	12.90	16.07
19.01	12.58	15.84
18.73	13.09	16.00
18.40	13.51	16.09
16.77	13.93	15.88
17.28	15.33	16.41
17.05	15.93	16.49

HOM-9-5

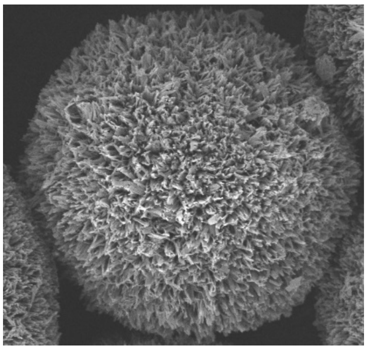

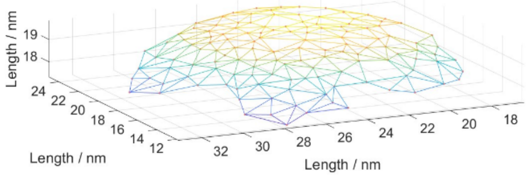

Average length =1.16±0.349μm

Modelling function: $\frac{(x-a)^2}{A}+\frac{(y-b)^2}{B}+\frac{z^2}{C}=1$ (a=22.8; b=20.9; A=C=19.9; B=19.9)

x	y	z
23.36	19.31	19.78
24.27	19.09	19.72
23.67	18.65	19.71
22.82	18.96	19.76
22.79	19.53	19.81
23.20	20.10	19.83
24.15	19.82	19.78
24.81	19.69	19.71
24.90	20.32	19.73
25.41	19.69	19.64
25.85	20.10	19.60
25.70	19.18	19.57
26.07	18.96	19.49
26.45	18.33	19.35
25.85	17.44	19.32
24.11	17.89	19.59
24.72	17.25	19.43
23.77	16.59	19.37
22.60	16.91	19.46
22.47	17.63	19.59
21.93	18.27	19.67
20.86	17.29	19.44
21.90	18.90	19.74
21.36	19.21	19.73
20.80	18.58	19.62
20.04	18.96	19.57
20.16	17.67	19.42
20.92	16.43	19.27
21.87	16.40	19.33
22.53	15.86	19.22
23.07	16.24	19.31
24.05	16.05	19.23
24.94	15.36	18.96
25.16	16.15	19.14
25.92	16.21	19.05
26.07	16.69	19.13
26.80	17.22	19.10
27.15	16.75	18.93
27.50	16.18	18.72
26.74	15.74	18.78
27.56	15.39	18.49
26.20	14.95	18.65
27.40	17.76	19.06
27.02	18.96	19.30
27.69	18.58	19.10
28.26	18.36	18.92
28.48	17.79	18.77
28.10	16.75	18.69
28.70	16.27	18.39
29.65	17.19	18.27
29.65	18.20	18.44
29.05	18.96	18.75
27.72	19.34	19.17
28.13	20.13	19.11
27.53	20.42	19.27
26.83	20.10	19.42
26.68	20.86	19.47
27.18	21.05	19.36
28.13	21.02	19.12
29.36	20.26	18.72
29.80	19.69	18.53
30.63	18.68	18.11
31.04	18.14	17.85
26.86	21.90	19.40
28.03	22.00	19.11
29.68	21.43	18.61
30.59	21.21	18.25
31.20	20.64	17.98
30.75	19.75	18.15
31.80	19.63	17.65
25.92	21.17	19.60
25.09	21.40	19.71
25.47	21.87	19.64
26.23	22.47	19.49
23.67	22.00	19.80
22.79	21.33	19.85
22.15	20.61	19.84
20.61	21.40	19.73
20.76	20.04	19.73
19.56	19.72	19.56
19.09	20.32	19.50
19.63	20.95	19.60
18.58	19.78	19.37
17.76	18.36	19.04
19.50	16.18	19.02
18.20	16.84	18.90
19.18	16.97	19.14
18.77	15.93	18.81
18.99	15.26	18.67
19.97	15.33	18.87
20.67	15.74	19.07
21.40	15.20	18.99

22.76	15.29	19.06
23.89	14.66	18.84
25.06	14.63	18.72
25.60	14.76	18.69
26.58	14.35	18.38
27.50	14.19	18.11
28.70	14.95	18.02
28.48	15.67	18.31
28.92	13.94	17.58
26.77	13.24	17.91
26.55	12.64	17.69
25.25	13.37	18.23
24.68	13.75	18.45

23.17	13.65	18.51
20.57	14.35	18.63
21.11	13.90	18.53
17.92	15.64	18.53
18.05	14.63	18.25
18.58	13.87	18.11
19.56	13.37	18.11
24.30	23.01	19.68
23.29	23.10	19.72
20.92	22.53	19.69
21.84	23.29	19.68
22.50	23.77	19.64
23.32	24.53	19.51

21.27	12.70	18.05
22.38	12.96	18.22
24.72	12.26	17.81
25.22	12.77	17.98
27.62	12.39	17.31
27.97	13.27	17.61
24.56	24.30	19.47
25.28	23.86	19.47
26.45	24.11	19.24
26.99	23.20	19.26
27.75	23.17	19.08
28.57	22.60	18.91
29.52	22.85	18.57

HOM-10-1

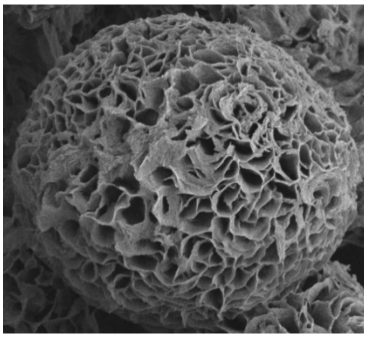

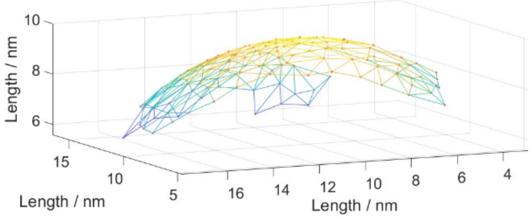

Average length =1.146±0.345μm

Modelling function: $\frac{(x-a)^2}{A}+\frac{(y-b)^2}{B}+\frac{z^2}{C}=1$ (a=10.3; b=9.31; A=C=9.85; B=8.83)

x	y	z
8.843	9.138	9.744
8.422	9.953	9.648
9.082	10.458	9.694
10.220	10.234	9.797
9.714	9.728	9.824
10.416	8.605	9.820
11.020	9.223	9.824
11.666	9.995	9.724
13.267	8.801	9.372
12.074	9.447	9.687
12.453	9.742	9.598
13.337	9.546	9.363
12.734	8.562	9.506
11.807	7.523	9.528
11.020	7.650	9.649
12.102	8.296	9.617
11.638	8.661	9.732
13.998	7.734	8.955
14.194	8.394	8.985
13.801	9.096	9.200
14.489	9.419	8.908
15.276	8.731	8.469
15.247	8.071	8.398
16.722	8.619	7.417
16.694	10.093	7.429
15.486	9.433	8.365
15.290	9.897	8.459
14.700	10.304	8.736
13.660	10.557	9.150
12.200	10.669	9.544
12.874	11.175	9.274
13.660	11.456	8.940
12.930	11.989	9.006
11.947	11.596	9.369
10.992	10.767	9.691
11.104	11.708	9.446
10.346	12.158	9.325
10.135	11.596	9.514
9.939	10.795	9.705
9.265	10.922	9.632
8.394	11.610	9.322
9.279	12.284	9.221
8.001	12.368	8.956
7.818	10.922	9.367
6.315	12.270	8.390
6.877	10.810	9.091
5.824	10.192	8.730
5.122	11.989	7.841
5.360	9.742	8.520
5.248	10.585	8.347
3.914	10.739	7.345
4.307	10.079	7.785
4.897	8.928	8.239
7.565	6.681	9.005
8.745	6.175	9.083
8.492	7.046	9.354
9.574	7.678	9.657
10.290	7.706	9.689
10.767	6.835	9.446
9.630	6.624	9.364
10.894	5.852	9.047
9.840	5.403	8.827
9.124	4.995	8.520
8.085	4.686	8.104
11.877	5.922	8.959
12.270	7.060	9.319
13.155	6.723	8.973
13.253	7.537	9.184
14.503	6.961	8.509
16.315	7.537	7.535
15.458	7.032	7.990
16.863	7.874	7.156
17.466	8.422	6.670
17.466	9.265	6.743
17.368	9.798	6.824
15.205	10.627	8.407
16.624	11.062	7.282
15.374	12.017	7.875
14.377	11.750	8.537
15.964	12.734	7.084
14.335	12.481	8.253
13.337	12.734	8.552
13.057	13.099	8.455
11.863	12.888	8.867
10.810	13.043	8.912
11.750	13.464	8.568
10.697	14.208	8.186
11.765	14.194	8.072
12.467	14.475	7.686
13.141	14.278	7.626
14.391	13.562	7.594
14.517	14.559	6.695

15.809	14.644	5.577
15.416	13.408	7.057
13.646	14.967	6.776
14.166	15.247	6.173
12.832	15.879	6.070
12.003	15.500	6.812
12.663	14.939	7.207
11.413	15.191	7.261
11.666	14.714	7.667
10.880	14.686	7.792
9.911	14.264	8.146

9.377	14.559	7.870
9.489	13.717	8.500
10.107	13.323	8.773
10.023	12.748	9.070
9.714	15.528	6.972
8.759	15.149	7.231
8.619	14.166	8.058
9.138	14.208	8.116
7.018	14.208	7.518
7.327	15.023	6.906
7.973	15.416	6.731

7.298	15.613	6.220
8.366	15.879	6.298
9.279	16.329	5.893
7.369	9.489	9.409
6.863	6.175	8.554
7.453	5.810	8.593
8.099	5.838	8.794
7.650	5.318	8.387
6.863	5.220	8.037
6.442	4.841	7.583
6.273	6.006	8.212

HOM-10-2	
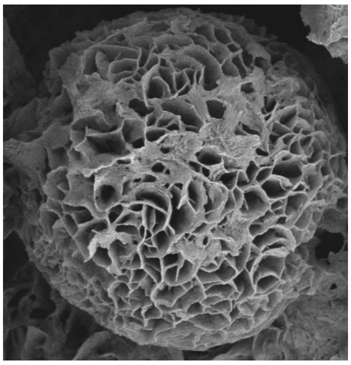	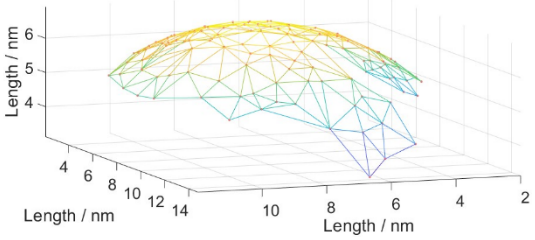<br>Average length =0.930±0.352μm

Modelling function: $\frac{(x-a)^2}{A}+\frac{(y-b)^2}{B}+\frac{z^2}{C}=1$ (a=7.49; b=7.47; A=C=6.92; B=7.18)

x	y	z
6.865	8.752	6.785
6.467	7.831	6.839
5.591	8.354	6.604
6.387	8.661	6.739
6.012	8.536	6.686
6.308	9.787	6.447
5.910	10.003	6.284
6.092	9.502	6.493
5.671	9.434	6.408
5.227	8.707	6.435
6.069	7.160	6.770
6.592	6.705	6.826
6.944	6.933	6.883
7.592	7.888	6.912
7.172	7.615	6.915
7.740	7.524	6.919
8.206	8.934	6.741
8.388	7.138	6.858
8.422	6.796	6.830
7.865	6.615	6.864
8.923	7.058	6.762
10.116	7.058	6.394
9.696	7.888	6.551
9.923	8.570	6.396
10.162	8.058	6.363
9.195	9.116	6.521
8.479	9.286	6.626
7.865	9.696	6.573
8.570	9.878	6.434
7.331	9.900	6.515
7.183	10.094	6.439
7.035	9.230	6.698
6.569	10.560	6.183
6.376	10.719	6.075
5.841	10.662	5.980
6.137	10.298	6.220
6.808	11.231	5.860
7.535	11.026	6.017
7.399	11.674	5.614
6.728	11.765	5.498
8.206	11.287	5.823
9.093	11.151	5.726
9.377	10.878	5.797
9.707	10.423	5.911
10.401	8.877	6.135
11.333	8.297	5.705
10.889	7.729	6.027
11.390	6.910	5.695
10.855	6.990	6.033
10.548	7.433	6.212
9.514	7.092	6.611
9.355	6.444	6.594
11.140	5.898	5.684
11.719	5.785	5.235
12.072	6.137	5.029
12.345	5.398	4.513
12.072	7.979	5.168
11.390	7.331	5.720
11.913	8.502	5.234
11.992	9.002	5.050
11.481	9.059	5.447
11.424	9.434	5.375
10.639	9.809	5.740
10.867	10.446	5.322
11.901	9.957	4.769
6.137	5.296	6.458
6.035	6.001	6.619
6.978	5.466	6.628
6.819	4.602	6.311
7.535	4.989	6.496
8.616	5.955	6.673
8.593	5.068	6.430
8.354	5.307	6.545
7.058	3.874	5.976
6.126	4.784	6.274
5.534	4.068	5.774
5.171	4.454	5.839
5.023	5.398	6.152
5.375	6.262	6.489
4.750	6.637	6.308
5.432	7.422	6.611
5.091	7.604	6.494
5.023	8.274	6.423
4.113	7.604	6.043
4.204	8.491	6.015
4.693	9.355	6.068
8.525	7.729	6.842
9.320	8.593	6.590
6.387	3.090	5.372
7.183	2.965	5.380
7.842	3.329	5.643
7.456	3.681	5.880

8.184	3.579	5.776
8.581	2.783	5.128
8.104	2.772	5.197
8.491	4.113	6.037
9.400	4.261	5.891
11.560	4.852	4.999
12.402	4.386	3.867
8.388	11.935	5.350
9.241	11.810	5.233

9.832	11.526	5.214
12.754	7.160	4.488
13.550	6.228	3.127
13.129	5.591	3.585
12.731	6.137	4.338
5.966	2.669	4.915
5.728	2.271	4.435
5.694	2.692	4.844
5.898	3.045	5.213

5.830	3.351	5.421
5.398	3.113	5.088
5.046	3.181	4.984
5.534	3.431	5.378
6.296	2.055	4.384
6.546	2.476	4.882
4.261	9.070	5.928
3.249	9.457	5.128

HOM-10-3

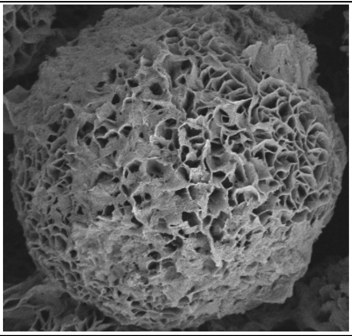

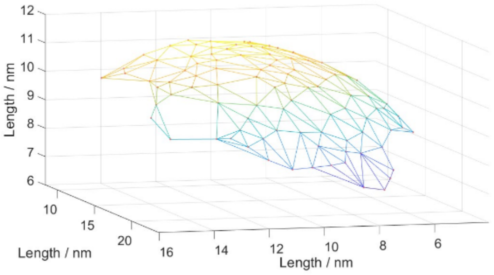

Average length =1.13±0.371μm

Modelling function: $\frac{(x-a)^2}{A}+\frac{(y-b)^2}{B}+\frac{z^2}{C}=1$ (a=12.1; b=11.4; A=C=11.4; B=11.1)

x	y	z
12.528	11.137	11.361
11.995	11.807	11.361
13.060	11.807	11.323
12.751	12.665	11.277
13.849	10.897	11.228
13.094	10.365	11.282
13.626	11.395	11.271
12.425	9.747	11.244
13.128	9.816	11.213
13.008	9.387	11.152
14.072	9.404	11.019
13.986	8.614	10.855
14.639	9.318	10.887
15.308	9.730	10.785
15.325	8.855	10.602
15.840	9.232	10.520
16.252	8.254	10.102
16.561	8.683	10.100
16.595	9.541	10.285
16.956	10.005	10.197
15.788	10.193	10.696
16.355	10.502	10.516
14.536	10.588	11.082
14.433	11.618	11.131
15.720	11.790	10.779
15.171	12.339	10.911
14.467	12.373	11.080
15.033	11.601	10.990
16.080	12.631	10.583
15.565	12.888	10.727
16.132	13.609	10.393
15.703	14.209	10.397
16.235	14.072	10.237
16.870	13.746	10.045
16.750	12.957	10.261
16.818	12.047	10.334
18.020	10.949	9.713
18.105	10.005	9.568
17.591	9.576	9.799
11.841	8.820	11.062
11.704	9.490	11.198
10.657	9.404	11.093
11.137	8.323	10.889
12.133	8.031	10.844
12.236	7.018	10.460
13.351	6.435	10.112
12.888	5.920	9.878
13.506	7.859	10.698
14.295	7.104	10.271
15.411	5.628	9.162
15.068	6.091	9.561
15.909	6.023	9.219
15.943	6.967	9.715
15.377	7.722	10.234
17.110	6.709	9.036
17.127	7.499	9.410
16.938	5.783	8.568
18.071	7.121	8.660
17.831	8.220	9.290
18.826	7.894	8.467
19.101	7.464	8.038
19.307	8.065	8.138
20.011	8.786	7.751
18.843	8.820	8.793
19.067	9.644	8.829
19.101	10.519	8.936
18.620	11.429	9.332
19.084	11.841	8.979
19.719	11.721	8.455
19.358	12.253	8.727
19.290	12.751	8.716
20.251	9.524	7.724
19.444	9.095	8.383
19.736	8.202	7.798
20.268	7.670	6.972
19.942	6.829	6.823
19.393	6.469	7.161
18.981	6.246	7.400
18.277	6.469	8.140
15.668	4.633	8.318
15.136	5.027	8.835
16.063	5.130	8.543
14.518	5.285	9.207
14.209	13.540	10.957
13.248	13.763	11.049
14.055	14.707	10.676
14.501	14.776	10.563
14.072	15.514	10.374
17.659	14.690	9.338
17.144	14.364	9.735
18.809	14.295	8.701
17.797	12.287	9.811
19.444	11.103	8.697
20.182	11.103	8.018
20.422	10.296	7.695

HOM-10-4

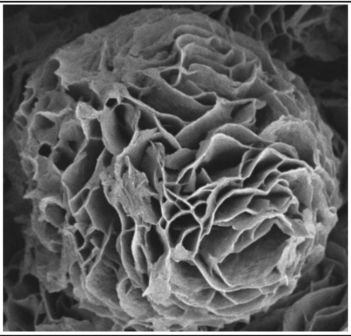

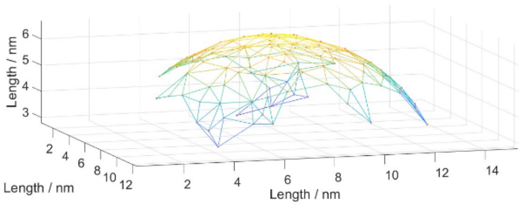

Average length =1.08±0.385μm

Modelling function: $\frac{(x-a)^2}{A}+\frac{(y-b)^2}{B}+\frac{z^2}{C}=1$ (a=7.34; b=7.01; A=C=6.71; B=6.75)

x	y	z
10.503	6.074	5.845
9.104	5.583	6.318
8.258	6.920	6.648
9.006	7.546	6.479
10.049	7.325	6.131
11.902	6.564	4.901
11.313	6.196	5.347
11.595	6.982	5.188
11.006	7.534	5.596
11.276	7.963	5.351
11.853	7.301	4.957
12.933	7.067	3.706
12.160	7.779	4.604
10.687	7.865	5.753
9.988	7.804	6.115
8.761	8.847	6.298
8.687	8.307	6.446
7.804	8.270	6.576
8.061	8.712	6.453
8.429	9.350	6.199
7.067	9.264	6.319
6.847	9.767	6.105
7.374	10.442	5.777
7.583	8.798	6.466
6.896	7.828	6.647
7.031	8.466	6.546
6.736	8.552	6.505
7.362	7.558	6.689
8.025	7.546	6.655
6.834	6.589	6.680
8.356	6.233	6.589
7.497	6.086	6.647
7.301	5.669	6.578
6.650	5.337	6.466
8.000	5.117	6.409
6.454	4.589	6.204
8.920	4.282	5.933
8.123	3.939	5.926
9.006	3.681	5.597
10.638	4.135	5.099
10.098	5.092	5.814
5.706	5.411	6.314
5.767	4.221	5.908
4.344	4.761	5.576
4.957	4.196	5.618
4.798	6.344	6.178
4.442	6.098	5.987
4.258	5.681	5.816
3.840	4.847	5.310
3.387	4.994	5.043
3.693	5.571	5.452
2.466	7.534	4.587
2.748	8.196	4.752
3.607	7.767	5.528
4.331	7.067	6.000
3.080	8.834	4.860
3.951	9.509	5.233
3.472	9.816	4.722
4.429	9.865	5.339
4.074	10.479	4.740
5.067	9.620	5.756
5.546	9.583	5.939
6.368	9.325	6.228
5.865	7.865	6.492
6.650	3.055	5.397
7.252	3.558	5.768
7.644	2.834	5.266
9.521	3.141	5.050
9.632	2.810	4.729
8.331	10.061	5.902
9.018	9.313	6.079
10.294	8.871	5.733
10.074	9.595	5.561
11.067	8.957	5.232
11.791	8.724	4.722
12.466	8.479	4.074
12.761	9.104	3.358
11.595	9.325	4.648
10.663	9.939	5.048
9.767	10.393	5.273
9.092	11.006	5.113
8.184	10.969	5.366
8.785	11.632	4.668
9.620	11.951	3.957
6.515	11.227	5.172
5.939	10.270	5.706
5.129	11.117	4.843
5.730	11.215	4.994
4.785	9.055	5.863
4.270	7.718	5.927
4.969	11.804	4.083
5.374	11.693	4.412
5.669	12.086	4.092
4.037	11.031	4.258
3.166	10.405	4.027

3.448	10.982	3.780
3.632	11.595	3.239
7.767	0.883	2.788
7.939	2.123	4.593
10.233	2.074	3.549

9.742	1.877	3.639
9.387	2.147	4.182
8.822	1.718	3.896
9.018	1.288	3.143
4.417	3.939	5.216

4.307	4.233	5.315
4.785	3.926	5.399
5.902	3.632	5.632
5.411	3.350	5.302
5.166	3.558	5.345

HOM-10-5	
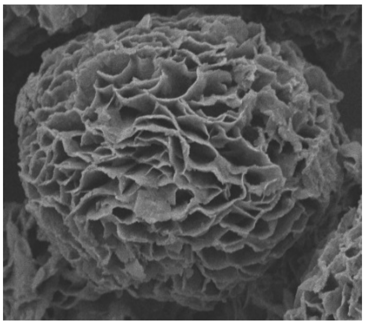	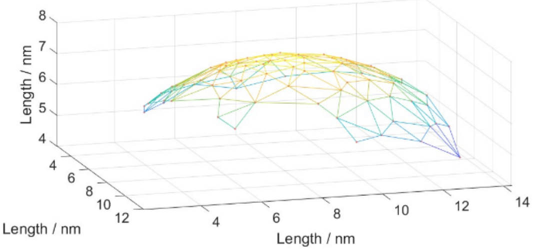<br>Average length =1.07±0.349μm
Modelling function: $\frac{(x-a)^2}{A}+\frac{(y-b)^2}{B}+\frac{z^2}{C}=1$ (a=8.35; b=7.37; A=C=7.52; B=6.64)	

x	y	z
6.214	5.748	6.977
8.079	5.418	7.186
7.454	4.667	6.814
7.192	5.304	7.056
8.398	6.123	7.390
9.728	6.510	7.331
9.239	5.327	7.103
8.762	7.533	7.510
10.411	6.123	7.096
11.423	6.407	6.779
11.036	5.714	6.771
10.263	5.611	6.997
10.707	5.179	6.699
8.568	4.497	6.779
6.965	4.281	6.515
5.361	4.292	5.960
6.237	3.860	6.028
7.488	3.621	6.150
9.353	3.473	6.007
7.897	2.950	5.596
10.684	4.315	6.258
11.446	4.963	6.290
12.140	7.033	6.486
10.263	7.226	7.274
9.933	7.840	7.335
11.491	7.840	6.814
11.423	8.557	6.732
12.777	7.681	6.070
10.502	8.932	6.987
9.387	8.728	7.290
9.637	9.387	7.050
8.421	8.739	7.361
8.603	8.102	7.473
8.079	7.715	7.509
8.159	8.386	7.432
7.579	7.602	7.480
7.351	7.795	7.442
7.249	7.351	7.443
6.840	7.511	7.369
7.943	6.783	7.483
7.215	6.442	7.363
6.351	6.419	7.174
6.055	7.204	7.164
6.407	6.771	7.238
5.020	6.794	6.717
5.429	7.249	6.934
7.659	9.512	7.087
10.070	10.172	6.598
9.308	9.831	6.920
8.364	10.445	6.666
8.830	10.980	6.293
10.980	9.626	6.566
12.140	10.206	5.645
12.435	9.865	5.646
12.981	9.456	5.434
12.401	9.001	6.061
11.776	9.365	6.302
4.770	5.100	6.099
4.269	4.895	5.667
4.519	4.053	5.275
5.270	3.689	5.453
4.269	7.101	6.316
4.383	7.545	6.392
5.566	8.102	6.941
5.532	9.046	6.713
6.385	8.546	7.140
7.215	8.568	7.313
5.247	9.774	6.290
6.453	9.865	6.709
5.600	10.490	6.045
11.139	6.999	6.974
12.424	6.112	6.160
4.895	5.873	6.467
4.087	5.975	5.997
4.918	6.316	6.590
6.646	5.998	7.162
10.752	10.741	6.017
11.582	11.036	5.371
10.183	11.355	5.728
9.262	11.685	5.641
13.015	10.559	4.661

HOM-14-1

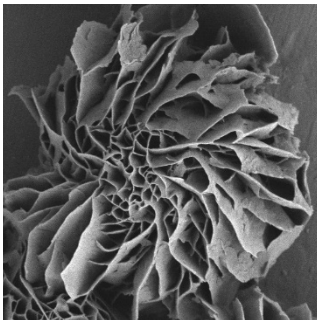

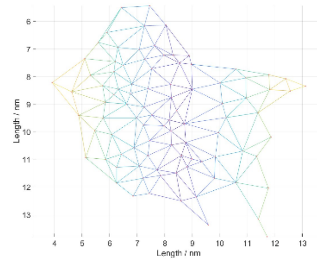

Average length =0.803±0.304μm

Modelling function: $z = 0$

x	y	z
8.210	8.274	0.000
7.531	7.705	0.000
7.389	8.431	0.000
8.242	8.747	0.000
8.431	9.221	0.000
7.247	9.095	0.000
7.531	9.379	0.000
7.232	9.742	0.000
6.979	9.489	0.000
7.453	10.310	0.000
8.242	10.468	0.000
8.226	9.963	0.000
7.974	9.774	0.000
8.163	9.568	0.000
8.716	9.695	0.000
9.347	11.068	0.000
8.984	10.974	0.000
8.763	10.374	0.000
8.605	10.847	0.000
8.810	11.258	0.000
8.416	11.479	0.000
9.805	11.668	0.000
9.837	10.895	0.000
11.337	10.879	0.000
11.858	10.184	0.000
11.384	9.300	0.000
10.089	8.968	0.000
10.231	9.474	0.000
10.958	8.763	0.000
10.942	8.037	0.000
10.579	7.721	0.000
10.010	8.226	0.000
8.889	7.232	0.000
9.000	8.447	0.000
8.968	7.547	0.000
8.542	7.500	0.000
8.305	7.216	0.000
7.958	7.216	0.000
7.279	7.358	0.000
7.626	6.868	0.000
7.074	7.010	0.000
7.263	6.158	0.000
8.037	6.126	0.000
7.453	5.432	0.000
6.426	5.495	0.000
6.300	6.932	0.000
6.205	7.453	0.000
6.489	7.579	0.000
6.805	8.258	0.000
6.158	7.816	0.000
6.758	8.605	0.000
6.316	8.447	0.000
6.158	8.653	0.000
6.616	8.984	0.000
5.747	8.321	0.000
5.842	8.921	0.000
9.947	10.026	0.000
10.831	10.689	0.000
10.579	11.826	0.000
11.763	12.031	0.000
11.400	13.152	0.000
11.716	13.784	0.000
9.584	13.358	0.000
9.142	12.347	0.000
8.589	12.489	0.000
7.437	12.221	0.000
7.847	11.574	0.000
7.721	11.368	0.000
7.216	11.668	0.000
7.074	11.226	0.000
7.389	10.847	0.000
6.426	10.800	0.000
6.884	10.563	0.000
6.079	10.089	0.000
5.763	9.489	0.000
5.463	9.947	0.000
11.779	7.926	0.000
11.810	8.463	0.000
12.552	8.510	0.000
13.105	8.337	0.000
12.395	8.068	0.000
5.100	7.342	0.000
5.589	6.758	0.000
5.905	6.395	0.000
5.305	7.942	0.000
4.626	8.542	0.000
4.879	9.395	0.000
3.916	8.210	0.000
5.132	10.926	0.000
5.858	10.989	0.000
6.253	11.274	0.000
6.253	11.842	0.000
6.853	12.316	0.000

HOM-14-2

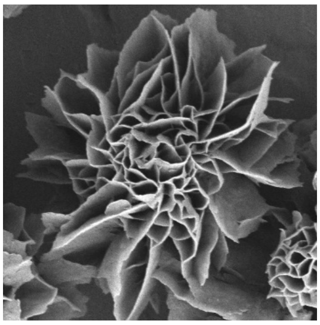

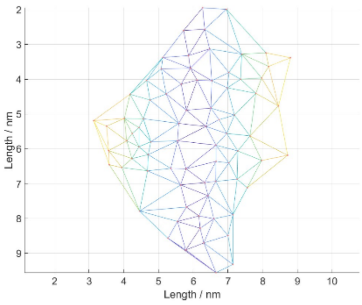

Average length =0.720±0.280μm

Modelling function: $z = 0$

x	y	z
4.183	4.430	0.000
5.223	4.421	0.000
4.659	4.033	0.000
5.655	4.139	0.000
5.082	3.699	0.000
5.866	3.320	0.000
5.126	3.373	0.000
5.725	2.597	0.000
6.333	2.562	0.000
6.272	1.945	0.000
6.474	3.126	0.000
5.919	3.681	0.000
6.113	4.025	0.000
6.536	4.042	0.000
6.985	4.245	0.000
7.029	3.804	0.000
6.959	1.989	0.000
7.364	3.998	0.000
7.400	3.276	0.000
8.096	3.240	0.000
7.972	3.963	0.000
8.801	3.381	0.000
8.175	3.628	0.000
7.805	4.404	0.000
7.188	5.029	0.000
8.457	4.765	0.000
7.637	5.628	0.000
6.968	5.558	0.000
7.285	6.060	0.000
7.972	6.333	0.000
8.721	6.184	0.000
7.558	7.118	0.000
7.267	6.871	0.000
6.651	6.932	0.000
7.029	6.404	0.000
6.659	6.360	0.000
6.659	5.893	0.000
6.369	5.355	0.000
6.747	5.161	0.000
6.510	4.827	0.000
5.805	4.888	0.000
4.756	4.597	0.000
5.584	5.267	0.000
5.549	5.769	0.000
4.782	5.796	0.000
4.562	5.135	0.000
4.016	5.126	0.000
4.245	5.558	0.000
4.439	5.937	0.000
4.086	5.769	0.000
4.439	5.390	0.000
4.703	6.166	0.000
4.686	6.633	0.000
5.355	6.562	0.000
5.796	6.571	0.000
5.637	7.038	0.000
6.430	7.347	0.000
5.805	7.673	0.000
6.333	6.968	0.000
6.598	8.008	0.000
7.144	7.893	0.000
7.003	8.492	0.000
6.166	7.928	0.000
6.201	8.281	0.000
5.549	8.087	0.000
5.276	8.571	0.000
5.787	8.906	0.000
6.307	8.704	0.000
6.659	9.558	0.000
7.144	9.320	0.000
4.456	7.787	0.000
3.945	6.536	0.000
4.236	6.280	0.000
3.566	6.051	0.000
3.584	6.457	0.000
3.135	5.188	0.000
3.505	5.346	0.000

HOM-14-3	
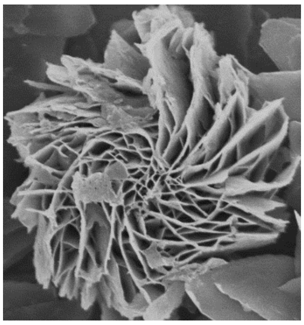	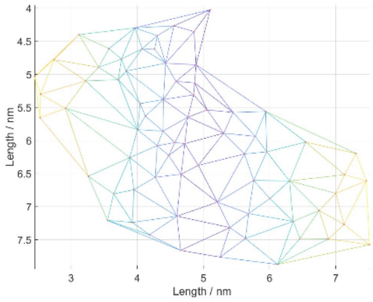<br>Average length =0.512±0.228μm
Modelling function: $z = 0$	

x	y	z
3.889	6.256	0.000
4.006	5.833	0.000
4.391	5.856	0.000
4.304	6.076	0.000
4.477	6.225	0.000
4.571	6.029	0.000
4.720	6.045	0.000
4.767	5.645	0.000
4.853	5.308	0.000
5.057	5.535	0.000
4.398	5.378	0.000
4.124	5.629	0.000
4.046	5.441	0.000
3.716	5.080	0.000
3.834	4.947	0.000
3.763	4.767	0.000
4.430	4.830	0.000
4.587	5.104	0.000
4.869	5.127	0.000
4.892	4.853	0.000
4.265	4.618	0.000
4.077	4.610	0.000
3.967	4.296	0.000
4.304	4.414	0.000

4.555	4.265	0.000
3.575	4.610	0.000
3.121	4.398	0.000
3.223	5.096	0.000
3.419	4.892	0.000
2.744	4.775	0.000
2.454	5.033	0.000
2.917	5.512	0.000
2.533	5.653	0.000
2.541	5.300	0.000
3.952	6.742	0.000
4.273	6.554	0.000
4.665	6.507	0.000
5.214	6.930	0.000
4.924	6.374	0.000
5.762	6.601	0.000
5.621	6.201	0.000
5.308	5.888	0.000
5.449	5.574	0.000
5.786	5.935	0.000
5.943	5.559	0.000
5.684	7.103	0.000
6.005	6.876	0.000
6.358	7.220	0.000
6.750	7.056	0.000

6.303	6.609	0.000
7.134	6.515	0.000
7.463	6.609	0.000
7.001	6.797	0.000
7.307	6.194	0.000
6.546	6.021	0.000
5.958	6.170	0.000
5.637	7.479	0.000
6.460	7.479	0.000
7.126	7.267	0.000
6.891	7.487	0.000
7.510	7.573	0.000
6.123	7.871	0.000
5.253	7.761	0.000
5.402	7.314	0.000
4.657	7.659	0.000
4.602	7.142	0.000
4.187	7.424	0.000
3.928	7.236	0.000
3.646	6.805	0.000
3.552	7.205	0.000
3.262	6.538	0.000
5.104	4.022	0.000
4.861	4.351	0.000

HOM-14-4

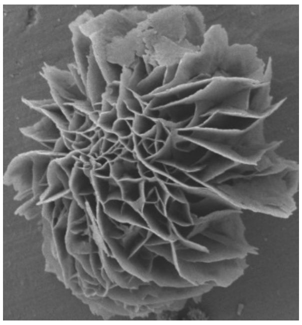

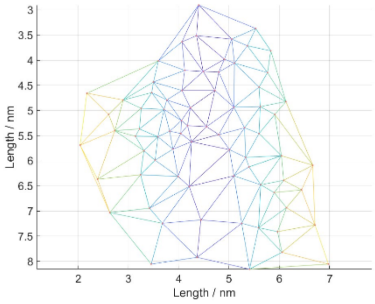

Average length =0.597±0.264μm

Modelling function: $z = 0$

x	y	z
4.067	4.600	0.000
4.489	4.222	0.000
4.122	4.200	0.000
4.800	4.233	0.000
4.722	4.600	0.000
4.233	4.944	0.000
4.800	5.478	0.000
5.100	4.933	0.000
5.600	4.533	0.000
5.622	4.922	0.000
5.311	5.344	0.000
5.600	5.456	0.000
5.911	5.078	0.000
6.133	4.822	0.000
5.767	4.244	0.000
5.111	4.056	0.000
4.911	3.978	0.000
5.367	3.711	0.000
5.056	3.578	0.000
5.833	3.811	0.000
5.533	3.367	0.000
4.356	3.511	0.000
4.400	2.900	0.000
4.067	3.633	0.000
3.600	4.011	0.000
3.500	4.433	0.000
3.767	4.789	0.000
3.711	4.978	0.000
3.589	5.522	0.000
4.044	5.456	0.000
4.178	5.300	0.000
4.056	5.200	0.000
3.822	5.411	0.000
4.256	5.622	0.000
4.556	5.322	0.000
5.011	5.778	0.000
5.100	6.300	0.000
5.400	6.122	0.000
5.633	6.478	0.000
6.089	6.389	0.000
6.444	6.578	0.000
5.900	6.633	0.000
6.044	5.900	0.000
5.333	5.933	0.000
6.667	6.089	0.000
6.711	7.278	0.000
6.144	6.933	0.000
5.967	7.411	0.000
5.589	7.333	0.000
5.256	7.244	0.000
4.444	7.178	0.000
4.211	6.511	0.000
3.989	6.311	0.000
3.244	6.278	0.000
3.889	5.767	0.000
3.533	5.989	0.000
3.311	5.800	0.000
3.144	5.511	0.000
3.222	5.367	0.000
3.256	4.956	0.000
3.467	4.644	0.000
2.900	4.789	0.000
2.733	5.400	0.000
2.611	5.078	0.000
2.178	4.656	0.000
2.044	5.689	0.000
2.389	6.367	0.000
2.633	7.033	0.000
3.422	6.944	0.000
3.678	7.244	0.000
3.456	8.056	0.000
4.367	7.922	0.000
5.411	8.156	0.000
6.056	7.822	0.000
6.978	8.056	0.000

HOM-14-5	
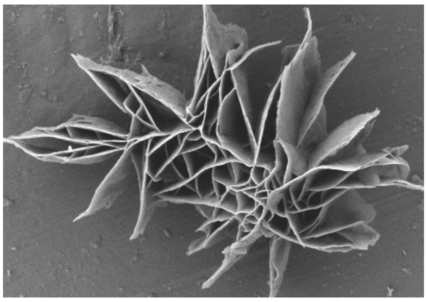	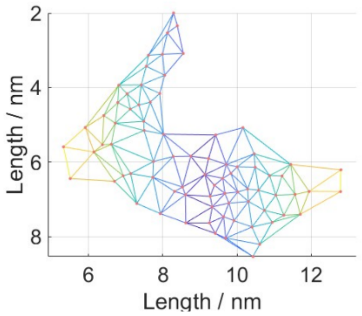 Average length =0.619±0.263μm
Modelling function: $z = 0$	

x	y	z
8.287	5.849	0.000
8.757	5.841	0.000
8.024	5.251	0.000
8.534	6.669	0.000
9.139	6.335	0.000
9.394	6.622	0.000
9.713	6.064	0.000
9.235	5.904	0.000
9.618	6.436	0.000
9.835	6.303	0.000
10.005	6.127	0.000
10.287	6.722	0.000
10.159	5.076	0.000
10.462	5.777	0.000
10.021	6.802	0.000
9.676	6.839	0.000
9.278	6.919	0.000
9.336	7.201	0.000
9.782	7.100	0.000
9.639	7.466	0.000
9.920	7.572	0.000
10.404	7.742	0.000
10.308	7.057	0.000
10.738	7.296	0.000
10.946	7.615	0.000
10.579	6.754	0.000

11.211	6.372	0.000
11.046	6.887	0.000
11.562	6.866	0.000
11.259	7.424	0.000
11.939	6.786	0.000
11.700	7.402	0.000
12.784	6.792	0.000
12.794	6.207	0.000
11.445	6.064	0.000
10.876	6.361	0.000
10.489	6.138	0.000
9.697	8.040	0.000
10.430	8.534	0.000
10.611	8.205	0.000
9.416	7.912	0.000
9.256	7.631	0.000
8.624	7.631	0.000
8.614	6.876	0.000
8.321	6.786	0.000
7.939	7.381	0.000
7.737	6.409	0.000
7.753	5.984	0.000
7.493	4.906	0.000
7.923	4.157	0.000
8.056	3.668	0.000
8.555	3.084	0.000
8.396	2.340	0.000

8.130	2.531	0.000
8.295	2.000	0.000
7.620	3.131	0.000
7.562	3.429	0.000
7.997	3.105	0.000
7.360	4.465	0.000
7.668	4.390	0.000
7.503	5.150	0.000
6.728	4.954	0.000
6.382	5.533	0.000
7.131	4.576	0.000
6.797	4.401	0.000
7.424	3.928	0.000
6.823	3.934	0.000
7.046	4.167	0.000
6.622	5.522	0.000
6.940	6.367	0.000
7.142	6.308	0.000
7.307	7.110	0.000
5.920	5.070	0.000
6.420	4.752	0.000
6.154	5.729	0.000
5.517	6.441	0.000
5.336	5.591	0.000
6.707	6.515	0.000
9.426	5.272	0.000

HOM-15-1

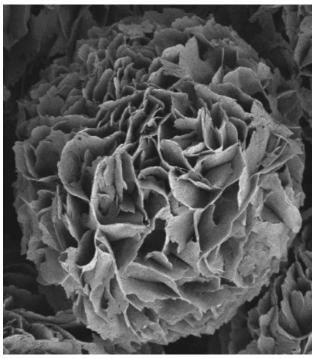

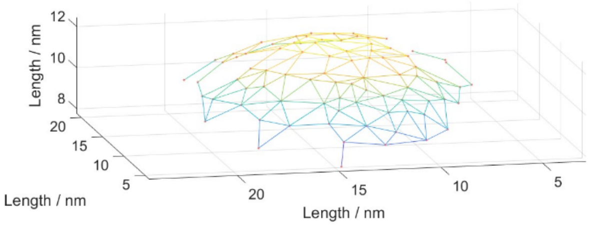

Average length =1.588±0.458μm

Modelling function: $\frac{(x-a)^2}{A}+\frac{(y-b)^2}{B}+\frac{z^2}{C}=1$ (a=13.1; b=14.4; A=C=12.2; B=13.8)

x	y	z
12.972	10.778	11.816
12.028	11.111	11.841
12.972	11.944	12.046
13.917	11.472	11.939
15.028	12.528	11.977
17.000	11.583	11.342
16.278	10.500	11.317
14.528	9.750	11.448
15.278	9.444	11.229
14.167	8.833	11.164
13.444	8.583	11.108
12.750	9.056	11.290
12.833	8.056	10.881
12.083	7.667	10.650
14.222	7.639	10.631
14.444	6.833	10.171
13.500	7.222	10.466
15.028	7.472	10.431
16.917	8.528	10.422
17.417	10.167	10.839
18.167	10.722	10.672
18.333	12.000	10.873
17.583	12.500	11.275
15.833	13.889	11.925
15.028	14.361	12.087
14.139	13.694	12.180
13.722	14.167	12.220

11.333	14.722	12.100
11.472	13.306	12.088
11.722	12.139	11.995
10.611	12.944	11.909
9.917	12.167	11.647
11.333	11.306	11.797
10.861	9.611	11.263
10.694	8.556	10.832
9.972	9.833	11.121
9.806	10.861	11.361
9.972	16.889	11.609
11.556	17.111	11.888
12.222	18.444	11.654
13.667	16.944	12.008
14.194	15.500	12.147
16.028	17.306	11.597
18.306	15.556	11.034
17.250	15.139	11.499
18.083	7.972	9.647
18.639	9.639	10.089
20.278	10.250	9.234
19.639	11.528	10.049
19.944	12.500	10.025
15.056	6.194	9.674
13.306	5.778	9.582
12.056	6.556	10.036
11.083	6.778	10.019
10.250	6.806	9.829

11.194	7.500	10.441
9.806	7.861	10.272
9.000	9.806	10.789
7.750	10.333	10.396
8.917	8.417	10.210
8.194	8.583	9.959
9.194	6.333	9.145
10.444	5.333	8.860
11.917	5.472	9.284
12.583	4.806	8.815
14.556	5.111	8.969
14.889	4.083	7.976
16.861	6.806	9.535
18.444	6.528	8.557
7.194	16.389	10.551
7.722	13.694	10.963
6.917	11.556	10.247
19.806	17.417	9.887
19.722	14.611	10.303
18.000	18.694	10.546
17.556	16.611	11.230
16.472	21.000	10.193
14.833	20.389	10.875
13.944	19.083	11.469
13.333	20.944	10.754
11.500	20.222	10.959
11.028	18.722	11.419
8.333	18.611	10.610

HOM-15-2

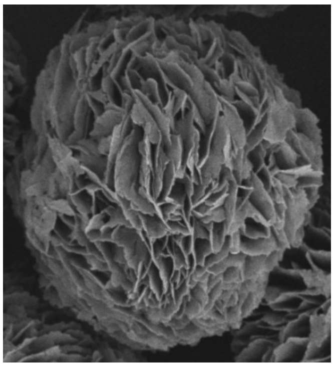

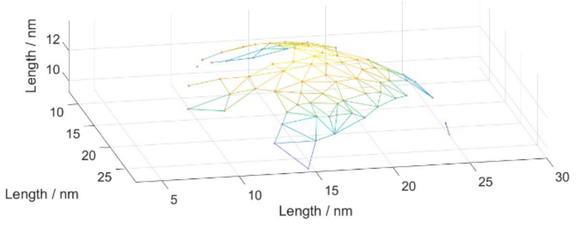

Average length =1.797±0.530μm

Modelling function: $\frac{(x-a)^2}{A}+\frac{(y-b)^2}{B}+\frac{z^2}{C}=1$ (a=15.8; b=16.9; A=C=13.9; B=15.6)

x	y	z
16.208	15.762	13.860
16.283	12.379	13.300
17.695	12.528	13.209
18.253	15.204	13.596
17.249	14.981	13.719
17.770	16.766	13.757
18.662	17.100	13.596
19.665	11.859	12.570
17.770	10.632	12.577
17.175	8.662	11.727
16.506	9.517	12.230
15.576	11.004	12.875
15.465	14.498	13.737
14.164	18.587	13.726
15.502	18.625	13.813
17.063	17.695	13.823
17.621	18.922	13.657
16.580	19.108	13.735
17.212	20.632	13.415
17.695	21.413	13.159
19.926	18.959	13.134
18.699	18.364	13.524
20.967	17.770	12.867
19.963	17.100	13.252
20.892	16.059	12.901
22.007	17.063	12.420
23.123	17.435	11.784
21.599	19.405	12.415
20.818	20.260	12.594
19.219	20.595	13.051
19.442	22.268	12.514
20.000	23.346	11.916
21.524	21.673	11.907
20.558	21.599	12.350
22.454	20.595	11.726
12.454	19.554	13.291
13.606	22.007	12.949
14.610	19.814	13.605
15.502	21.413	13.298
15.167	22.639	12.903
14.721	23.680	12.464
15.576	23.643	12.522
16.543	22.788	12.839
17.323	23.717	12.393
17.770	23.383	12.472
18.885	24.647	11.638
18.104	24.461	11.916
17.063	24.721	11.941
15.725	24.833	11.953
16.543	24.870	11.908
16.617	26.171	11.125
15.316	26.580	10.867
13.941	24.164	12.152
13.457	25.019	11.626
14.498	28.141	9.520
12.714	26.171	10.733
10.297	22.156	11.884
10.929	20.781	12.562
12.491	21.227	12.943
10.781	18.439	12.905
9.033	17.063	12.165
12.416	11.152	12.489
11.264	10.558	11.885
13.643	11.896	13.001
14.461	11.710	13.051
15.279	7.063	10.785
12.416	7.955	10.894
13.494	7.361	10.770
15.576	8.625	11.790
20.297	9.331	11.285
23.309	15.874	11.642
24.201	14.907	10.908
23.680	13.457	11.013
24.796	17.472	10.555
24.944	20.818	9.829
24.944	21.970	9.398
8.401	19.926	11.476
10.223	14.015	12.493

HOM-15-3

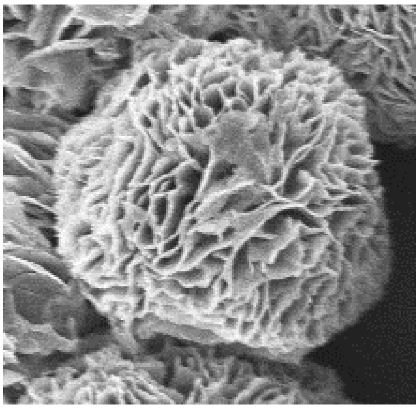

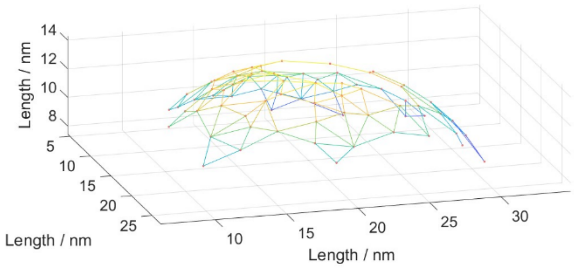

Average length =2.167±0.679μm

Modelling function: $\frac{(x-a)^2}{A}+\frac{(y-b)^2}{B}+\frac{z^2}{C}=1$ (a=19.0; b=16.7; A=C=14.4; B=13.5)

x	y	z
16.583	9.667	12.108
17.667	8.917	11.757
18.333	9.500	12.228
17.833	11.000	13.055
19.708	8.583	11.565
18.958	7.167	10.288
20.167	6.792	9.818
20.417	8.375	11.334
21.375	9.000	11.674
22.500	8.583	11.061
21.917	6.583	9.204
24.833	6.667	7.841
23.417	10.167	11.894
23.792	7.917	9.953
24.833	13.958	12.915
24.958	9.542	10.770
27.875	11.292	9.897
28.292	9.833	8.377
26.375	10.625	10.653
29.125	11.583	8.821
28.417	15.583	10.936
25.833	17.375	12.729
23.917	16.375	13.593
26.792	14.667	12.006
30.542	16.667	8.756

29.458	17.208	10.002
28.250	18.500	10.964
26.333	19.792	12.020
22.250	20.083	13.602
24.708	20.958	12.475
20.292	22.750	12.840
18.500	21.750	13.370
20.583	21.125	13.547
19.208	19.542	14.107
21.042	19.583	13.959
16.583	20.208	13.713
15.458	23.292	12.064
12.875	23.292	10.966
13.875	21.042	12.640
13.917	22.125	12.169
15.458	20.417	13.396
17.208	18.667	14.158
17.000	17.125	14.281
18.583	13.542	14.040
21.333	15.667	14.218
16.375	13.958	13.888
15.625	12.000	13.110
13.083	15.667	13.098
15.750	16.042	14.037
14.875	16.750	13.817
13.542	16.917	13.338

12.833	18.792	12.826
11.458	17.625	12.230
10.958	16.500	11.947
12.500	11.583	11.662
14.458	10.750	12.143
13.875	13.500	13.044
11.708	10.000	10.190
12.583	9.083	10.054
13.917	8.500	10.303
15.417	7.792	10.278
16.667	8.292	11.090
16.958	7.000	9.889
20.125	5.625	8.290
9.750	16.750	11.032
11.458	21.000	11.370
10.500	22.500	9.821
18.167	23.333	12.535
17.667	24.792	11.465
18.792	26.083	10.372
20.250	25.208	11.140
21.958	22.792	12.546
23.458	23.750	11.493
26.208	22.750	10.738
29.083	20.917	9.354
28.917	20.000	9.935
31.292	18.583	7.396

HOM-15-4

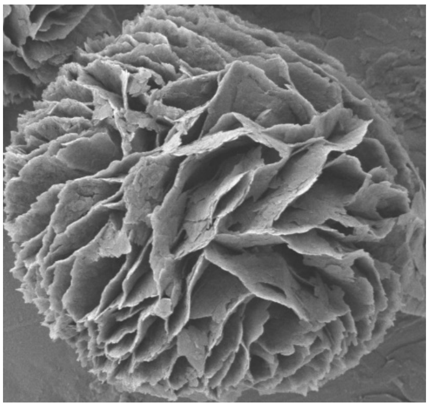

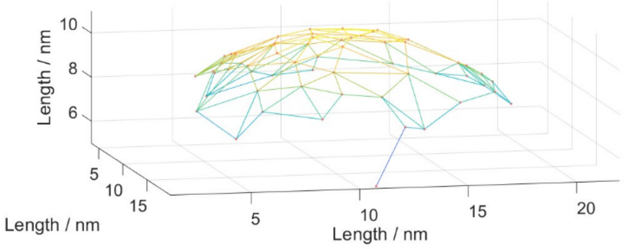

Average length =2.167±0.679μm

Modelling function: $\frac{(x-a)^2}{A}+\frac{(y-b)^2}{B}+\frac{z^2}{C}=1$ (a=11.1; b=10.6; A=C=10.9; B=10.4)

x	y	z
11.274	12.284	10.737
13.226	8.995	10.545
12.616	10.826	10.775
11.310	10.405	10.880
14.784	8.342	9.971
12.931	6.847	9.995
10.300	8.216	10.570
7.963	11.268	10.398
8.342	13.879	9.952
13.816	12.068	10.425
17.647	7.626	8.128
17.037	8.616	8.888
18.321	8.174	7.746
19.289	9.416	7.065
18.131	10.763	8.306
17.500	11.353	8.767
16.679	11.900	9.244
13.247	15.289	9.474
10.658	13.310	10.497
9.816	15.268	9.640
8.258	15.605	9.107
8.805	17.458	7.859
11.689	16.847	8.684
9.900	16.574	8.830
17.689	13.226	8.213
15.479	16.005	8.204
13.395	17.963	7.344
12.489	18.047	7.477
10.763	19.795	5.099
8.974	7.521	10.185
10.805	5.458	9.474
12.237	5.963	9.691
14.805	4.974	8.394
16.595	5.900	8.024
7.816	12.510	10.180
6.426	14.721	8.831
6.384	15.942	8.061
5.100	16.237	6.905
6.468	12.489	9.647
5.268	10.742	9.188
6.468	9.984	9.829
4.805	9.121	8.747
6.405	9.395	9.741
6.405	8.005	9.443
6.616	6.363	8.886
9.121	6.216	9.684
7.837	5.079	8.650
11.458	3.521	8.001
6.447	4.005	7.049
6.489	5.479	8.296
8.531	4.089	8.120
9.605	3.458	7.806
11.500	4.363	8.727
9.647	11.731	10.720
3.963	13.163	7.765
5.732	11.731	9.392
10.700	6.847	10.154
14.089	6.658	9.630

HOM-15-5

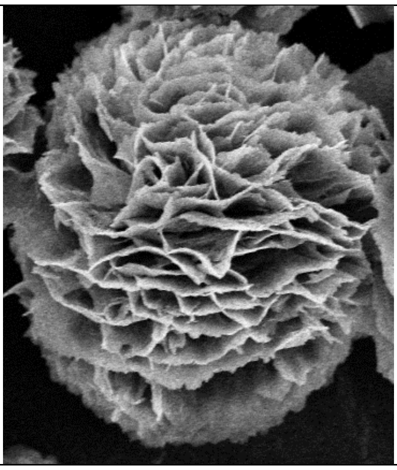

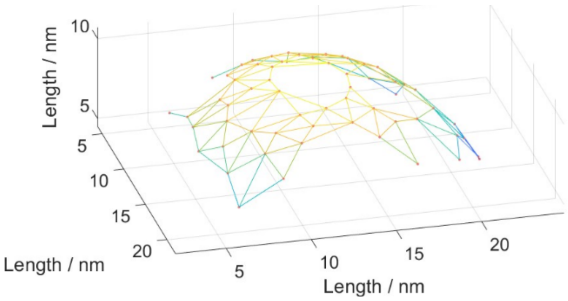

Average length =2.300±0.681μm

Modelling function: $\frac{(x-a)^2}{A}+\frac{(y-b)^2}{B}+\frac{z^2}{C}=1$ (a=15.2; b=12.1; A=C=13.9; B=10.9)

x	y	z
14.193	13.732	10.687
13.403	16.663	10.555
9.912	14.984	10.646
9.748	13.370	10.645
10.373	11.526	10.587
13.568	12.020	10.681
15.873	11.065	9.931
12.415	9.715	10.315
11.098	10.933	10.572
12.909	10.933	10.581
11.065	8.727	9.951
9.879	10.176	10.217
9.287	8.760	9.622
8.134	9.978	9.627
8.200	11.460	9.992
6.915	13.041	9.588
8.628	13.600	10.346
7.113	14.160	9.713
5.137	13.831	8.425
7.640	17.486	9.486
12.020	17.519	10.454
10.933	18.771	10.019
10.439	17.684	10.286
8.825	18.441	9.667
13.238	18.804	10.000
14.819	17.453	10.098
15.906	18.244	9.502
17.420	17.223	9.053
13.963	15.478	10.644
19.100	14.424	8.302
20.121	15.906	7.124
18.507	12.613	8.720
17.190	13.139	9.594
16.926	10.110	9.220
18.869	8.595	7.250
15.741	7.870	8.922
15.148	8.957	9.595
12.382	7.377	9.418
10.604	6.092	8.582
14.193	5.829	8.258
8.364	5.631	7.542
12.975	4.676	7.601
4.973	9.089	7.241
5.203	12.283	8.373
4.874	16.169	7.968
6.685	15.543	9.380
6.026	17.256	8.619
5.829	19.100	7.780
7.936	19.462	8.917
8.529	21.372	8.136
5.960	21.208	6.575
10.011	19.759	9.480
14.588	19.759	9.360
19.594	18.507	6.825
18.935	6.454	5.683
16.531	20.911	7.959
17.519	5.862	6.579
14.588	7.377	9.076
20.483	13.864	6.910
20.746	15.016	6.504
21.767	14.325	4.929
21.998	13.666	4.459

HOM-1-6 (NMP-EtOH)

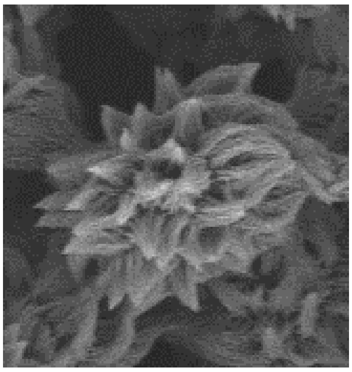

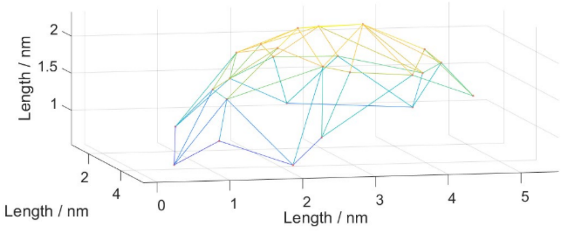

Average length =1.05±0.311μm

Modelling function: $\frac{(x-a)^2}{A}+\frac{(y-b)^2}{B}+\frac{z^2}{C}=1$ (a=2.93; b=3.20; A=C=2.36; B=2.24)

x	y	z
2.870	2.475	2.231
3.296	1.728	1.735
2.123	2.265	1.985
1.648	2.895	1.956
2.414	3.241	2.304
2.025	3.747	2.104
3.321	3.179	2.328
2.494	4.438	1.919

2.833	4.586	1.852
1.272	3.994	1.460
1.099	4.302	0.937
1.179	3.543	1.544
4.074	3.611	2.019
4.475	2.827	1.740
4.364	2.179	1.531
4.333	1.685	1.014
2.710	1.216	1.057

1.790	1.840	1.484
2.395	1.796	1.754
0.802	2.926	0.982
0.654	3.519	0.536
2.327	5.123	1.048
1.932	5.123	0.683
3.716	4.469	1.780
4.679	3.889	1.410

HOM-1-7 (NMP-EtOH)	
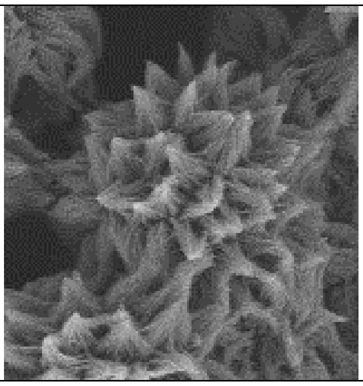	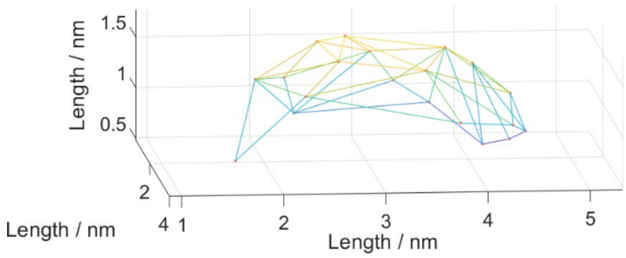 Average length =0.943±0.306μm

Modelling function: $\frac{(x-a)^2}{A}+\frac{(y-b)^2}{B}+\frac{z^2}{C}=1$ (a=3.09; b=2.40; A=C=1.80; B=1.80)

x	y	z
2.759	2.364	1.765
2.512	2.123	1.677
1.895	2.210	1.325
1.611	3.160	0.676
2.284	3.309	1.323
2.636	3.031	1.620

3.469	3.228	1.550
3.790	1.827	1.553
4.062	1.802	1.389
3.099	1.340	1.449
2.259	1.364	1.207
2.395	0.944	0.785
4.340	2.759	1.242

4.321	3.247	1.002
3.772	3.654	1.094
3.364	1.012	1.106
3.716	0.963	0.877
4.228	1.093	0.475
4.463	1.389	0.569
4.593	1.698	0.694

HOM-1-8 (NMP-EtOH)

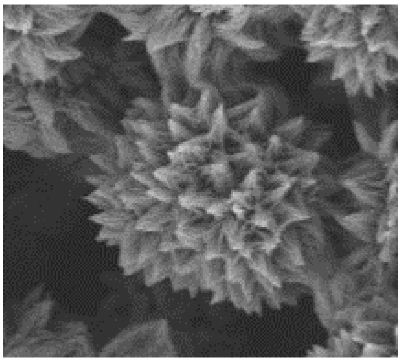

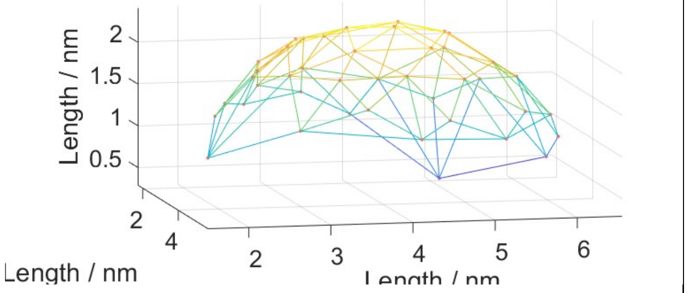

Average length =0.824±0.269μm

Modelling function: $\frac{(x-a)^2}{A}+\frac{(y-b)^2}{B}+\frac{z^2}{C}=1$ (a=4.02; b=3.75; A=C=2.44; B=2.19)

x	y	z
3.531	2.846	2.166
3.241	3.043	2.172
3.383	2.302	1.717
4.358	2.068	1.526
4.074	1.784	1.080
3.426	2.031	1.394
4.920	3.457	2.238
4.790	3.809	2.308
4.321	3.352	2.376
3.586	3.877	2.393
4.130	4.031	2.413
3.074	3.358	2.204
2.901	3.716	2.165
2.617	3.377	1.951

2.741	2.765	1.764
2.809	2.444	1.541
2.660	2.358	1.306
5.019	2.117	1.280
4.994	2.833	1.984
5.457	2.698	1.578
6.204	2.994	0.662
5.154	1.827	0.280
6.000	4.185	1.327
5.735	3.500	1.702
5.272	4.315	1.990
5.556	4.827	1.459
4.784	4.957	1.883
4.062	5.062	1.952
4.463	4.568	2.215

3.531	4.574	2.205
2.759	4.488	1.919
2.432	3.932	1.839
5.216	5.321	1.204
4.506	5.451	1.458
4.123	5.623	1.264
3.531	5.346	1.599
3.272	4.938	1.908
2.741	5.173	1.346
1.821	4.210	0.925
2.000	3.790	1.367
2.346	2.741	1.375
6.235	3.531	0.973

Particle in reference[1]

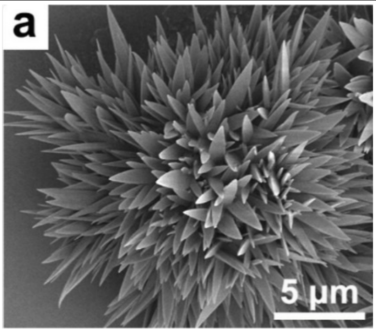

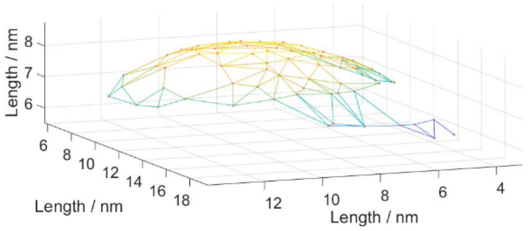

Average length =1.181±0.406μm

Modelling function: $\frac{(x-a)^2}{A}+\frac{(y-b)^2}{B}+\frac{z^2}{C}=1$ (a=11.6; b=9.20; A=C=8.64; B=8.56)

x	y	z
12.847	9.690	8.530
13.759	9.580	8.347
12.719	9.124	8.562
13.504	8.723	8.406
13.869	7.828	8.211
14.161	9.726	8.223
14.599	9.489	8.083
14.891	8.577	7.948
14.982	7.518	7.751
15.274	7.172	7.529
13.394	6.314	7.926
12.956	7.372	8.326
12.701	7.518	8.396
12.117	6.953	8.320
12.226	6.332	8.115
11.569	7.482	8.465
12.135	5.292	7.667
11.496	5.675	7.875
11.661	4.818	7.423
11.880	4.398	7.147
11.314	4.288	7.073
10.456	5.109	7.510
10.219	5.511	7.682
10.383	7.628	8.412
11.223	8.759	8.623
9.599	7.464	8.231
9.380	6.515	7.910
9.270	5.201	7.289
10.821	9.270	8.609
10.620	9.580	8.581
12.172	10.620	8.498
10.730	10.912	8.424
11.715	11.058	8.432
8.504	9.854	8.055
8.741	8.193	8.104
8.942	7.263	7.999
9.051	6.807	7.908
11.734	12.245	8.072
11.131	12.318	8.034
10.036	11.478	8.187
13.102	12.865	7.653
14.270	12.591	7.455
13.303	10.949	8.276
14.526	11.058	7.895
15.018	10.401	7.825
15.493	11.442	7.352
15.730	10.128	7.509
15.657	9.307	7.607
15.383	8.960	7.745
15.620	8.102	7.547
15.547	7.847	7.544
16.241	5.985	6.499
16.807	7.445	6.634
16.934	8.741	6.751
16.861	8.540	6.791
15.803	5.310	6.423
15.803	4.617	5.939
14.088	5.821	7.527
14.416	4.069	6.301
13.704	3.084	5.654
10.109	4.325	6.954
9.799	4.635	7.095
9.234	8.796	8.312
8.303	9.142	8.003
9.471	10.182	8.325
9.106	10.602	8.162
9.872	12.920	7.595
11.168	13.504	7.456
13.029	13.777	7.150
11.150	13.960	7.167
13.869	13.376	7.178

Particle in reference[2]	
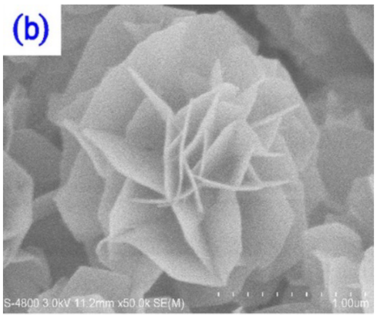	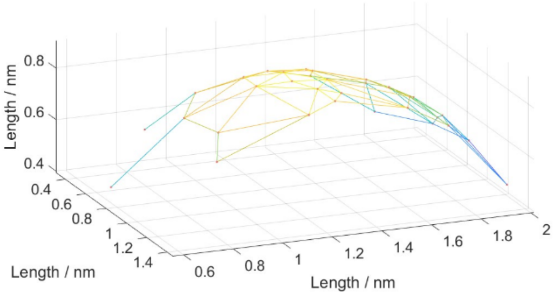 Average length =0.218±0.080μm
Modelling function: $\frac{(x-a)^2}{A}+\frac{(y-b)^2}{B}+\frac{z^2}{C}=1$ (a=1.26; b=1.04; A=C=0.875; B=0.977)	

x	y	z
1.123	0.771	0.830
1.090	0.976	0.856
1.246	0.863	0.860
1.267	0.655	0.804
1.215	1.021	0.874
1.473	0.572	0.738
1.357	0.813	0.845
1.348	1.040	0.871
1.270	1.139	0.870
1.345	1.189	0.861

1.293	1.253	0.853
1.161	1.317	0.833
0.851	1.184	0.763
0.790	1.319	0.695
0.820	0.922	0.749
0.981	0.641	0.748
1.440	0.674	0.791
1.643	0.704	0.727
1.610	0.787	0.769
1.730	0.960	0.735
1.591	1.241	0.790

1.676	1.054	0.770
1.761	1.229	0.697
2.012	1.189	0.427
1.936	1.000	0.554
1.863	0.870	0.615
1.818	1.028	0.674
1.887	0.766	0.559
1.681	0.839	0.746
1.754	0.520	0.551
0.858	0.444	0.564
0.556	0.849	0.490

Particle in reference[3]	
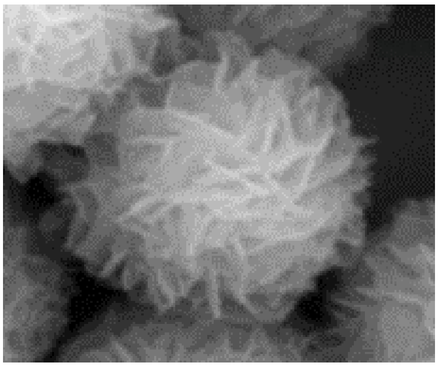	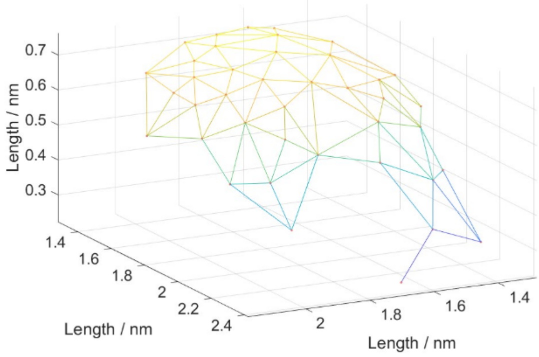<br>Average length =0.160±0.046μm

Modelling function: $\frac{(x-a)^2}{A}+\frac{(y-b)^2}{B}+\frac{z^2}{C}=1$ (a=1.57; b=1.73; A=C=0.768; B=0.722)

x	y	z
1.725	1.562	0.729
1.578	1.649	0.762
1.871	1.696	0.703
1.709	1.722	0.754
1.905	1.489	0.638
1.809	1.453	0.665
1.496	1.436	0.697
1.676	1.335	0.632
1.842	1.340	0.583
2.002	1.556	0.603
1.893	1.595	0.679
2.115	1.609	0.520
1.958	1.402	0.559
2.029	1.860	0.596
1.940	1.816	0.664
1.825	1.824	0.716
1.744	1.875	0.731
1.587	1.847	0.757
1.495	1.796	0.761
1.700	1.973	0.710
1.827	1.940	0.687
1.802	2.022	0.661
1.711	2.042	0.677
1.773	2.158	0.583
1.907	2.056	0.594
1.940	1.938	0.633
2.102	1.944	0.500
2.049	2.042	0.496
2.196	1.927	0.385
2.089	1.789	0.558
1.438	1.669	0.754
1.407	1.571	0.731
2.107	1.440	0.447
2.060	1.385	0.457
2.125	1.300	0.257
2.207	1.493	0.337
2.293	1.636	0.223
1.542	2.040	0.693
1.471	1.882	0.745
1.624	1.933	0.735

Particle in reference[4]

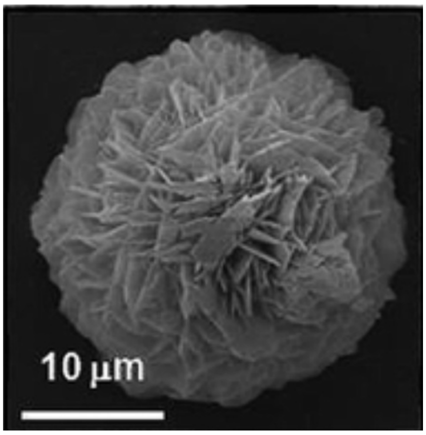

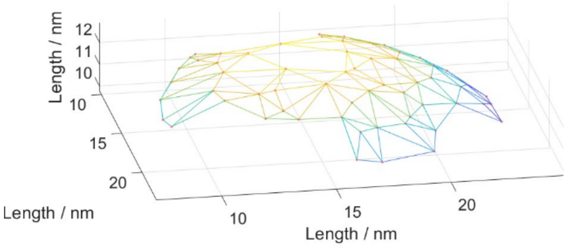

Average length =1.80±0.567μm

Modelling function: $\frac{(x-a)^2}{A}+\frac{(y-b)^2}{B}+\frac{z^2}{C}=1$ (a=15.0; b=15.0; A=C=26.0; B=16.0)

x	y	z
17.000	14.895	12.837
17.947	15.395	12.649
17.000	16.711	12.718
16.553	17.079	12.723
19.632	18.632	11.579
19.711	16.868	11.966
19.605	13.105	12.015
19.211	14.632	12.294
21.316	17.395	11.105
20.500	15.789	11.754
18.553	12.526	12.263
17.132	13.079	12.678
15.526	18.079	12.597
16.421	19.368	12.137
17.289	20.711	11.425
17.316	18.447	12.301
18.895	19.711	11.455
18.605	20.184	11.342
19.947	20.053	10.892
19.684	21.237	10.377
18.895	18.105	11.996
18.079	17.816	12.299
16.500	21.026	11.390
17.211	22.368	10.443
15.816	20.263	11.829
11.132	17.895	12.032
13.289	18.158	12.464
14.026	16.605	12.842
11.500	15.553	12.481
11.447	14.289	12.462
12.816	14.158	12.770
11.816	13.079	12.439
14.342	13.553	12.891
10.868	12.868	12.119
10.026	12.895	11.800
10.763	11.526	11.771
11.079	10.974	11.708
16.526	10.763	12.199
17.553	11.053	12.127
16.368	12.263	12.634
19.263	11.263	11.711
20.789	11.421	11.093
21.763	12.605	10.858
21.921	15.105	11.012
21.026	13.395	11.418
23.342	13.342	9.854
22.395	13.895	10.649
22.816	15.684	10.376
23.474	16.395	9.770
23.289	14.816	10.029
16.105	22.368	10.613
13.947	20.211	11.827
13.395	19.579	12.026
8.579	17.132	11.054
8.816	17.921	11.007
10.816	16.842	12.136
8.763	15.447	11.359
12.579	18.158	12.344
12.579	19.789	11.802
9.447	14.263	11.700

Particle in reference[5]

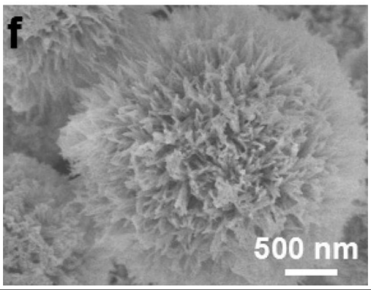

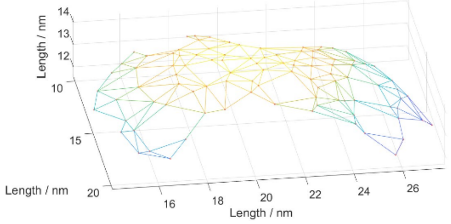

Average length =1.07±0.414μm

Modelling function: $\frac{(x-a)^2}{A}+\frac{(y-b)^2}{B}+\frac{z^2}{C}=1$ (a=20.1; b=14.2; A=C=14.1; B=12.7)

x	y	z
19.500	14.341	14.144
19.659	13.864	14.146
20.659	14.341	14.148
20.841	14.795	14.125
21.227	14.273	14.117
21.773	14.000	14.063
22.455	14.432	13.965
21.932	14.727	14.031
22.341	14.864	13.965
22.182	15.386	13.946
21.023	15.341	14.072
22.886	15.841	13.765
23.114	16.386	13.621
23.818	15.182	13.626
23.932	15.682	13.537
24.500	15.182	13.423
24.000	14.341	13.620
23.091	14.750	13.833
23.273	13.932	13.804
22.886	13.341	13.857
23.386	13.159	13.733
23.023	12.864	13.783
24.205	13.705	13.551
24.886	13.864	13.334
21.205	13.318	14.085
20.409	12.864	14.079
20.068	12.455	14.026
19.136	13.000	14.062
18.568	13.477	14.050
19.091	14.205	14.120
19.659	15.432	14.082
19.409	16.000	13.993
18.000	16.341	13.787
17.523	15.614	13.823
17.523	14.886	13.894
17.977	14.295	13.993
17.523	14.068	13.915
16.705	12.273	13.570
17.705	12.795	13.862
17.909	13.205	13.940
17.977	12.523	13.870
18.932	11.591	13.807
19.432	11.341	13.780
18.932	10.591	13.524
18.636	10.432	13.440
24.341	12.159	13.329
23.841	12.364	13.514
23.636	12.886	13.643
25.068	13.273	13.233
24.886	12.227	13.158
25.500	13.273	13.064
25.909	13.182	12.880
26.477	14.136	12.660
26.591	14.841	12.580
26.977	15.068	12.357
26.636	15.545	12.486
25.773	15.114	12.947
23.864	17.000	13.291
24.545	16.773	13.139
24.114	16.955	13.230
24.386	17.705	12.918
24.386	18.182	12.744
24.705	18.545	12.481
26.318	17.045	12.329
26.364	16.386	12.474
26.068	16.477	12.598
27.386	16.455	11.893
27.773	16.455	11.649
22.932	17.045	13.506
22.909	17.523	13.372
22.659	17.886	13.302
21.159	17.614	13.590
20.295	16.864	13.837
20.659	16.455	13.919
20.455	16.136	13.986
18.568	16.159	13.897
18.273	17.750	13.457
19.091	17.727	13.552
15.409	18.750	12.330
15.841	18.864	12.434
14.886	17.364	12.659
15.386	17.568	12.790
15.159	16.864	12.912
16.386	16.341	13.438
16.818	16.750	13.462
16.591	19.500	12.350
17.318	18.841	12.860
16.114	13.273	13.538
15.955	15.000	13.497
15.000	15.295	13.136
14.091	15.295	12.743
14.432	14.000	12.958
15.182	12.841	13.179
15.682	12.227	13.261
16.364	11.045	13.189
26.341	18.182	11.915
25.886	19.136	11.692

Supplementary References

1 Li, Q. *et al.* Shear Stress Triggers Ultrathin-Nanosheet Carbon Nitride Assembly for Photocatalytic H2O2 Production Coupled with Selective Alcohol Oxidation. *J. Am. Chem. Soc.* (2023).

2 Li, T. *et al.* Large-Scale Self-Assembly of 3D Flower-like Hierarchical Ni/Co-LDHs Microspheres for High-Performance Flexible Asymmetric Supercapacitors. *ACS Appl. Mater. Interfaces* **8**, 2562-2572 (2016).

3 Xu, Z. *et al.* Nitrogen-Doped Porous Carbon Superstructures Derived from Hierarchical Assembly of Polyimide Nanosheets. *Adv. Mater.* **28**, 1981-1987 (2016).

4 Zhang, F., Bao, Y., Ma, S., Liu, L. & Shi, X. Hierarchical flower-like nickel phenylphosphonate microspheres and their calcined derivatives for supercapacitor electrodes. *J. Mater. Chem. A* **5**, 7474-7481 (2017).

5 Li, Q. *et al.* Hierarchical MoS2/NiCo2S4@C urchin-like hollow microspheres for asymmetric supercapacitors. *Chem. Eng. J.* **380**, 122544 (2020).
