# Supplementary material for: Hierarchical organic microspheres from diverse molecular building blocks
Source: Nat Commun. 2024 Jun 13;15:5041. doi: 10.1038/s41467-024-49379-7 (PMC11176358; doi:10.1038/s41467-024-49379-7)
Supplement: Supplementary file 6 — Supplementary Data 2 [file 41467_2024_49379_MOESM6_ESM.pdf]

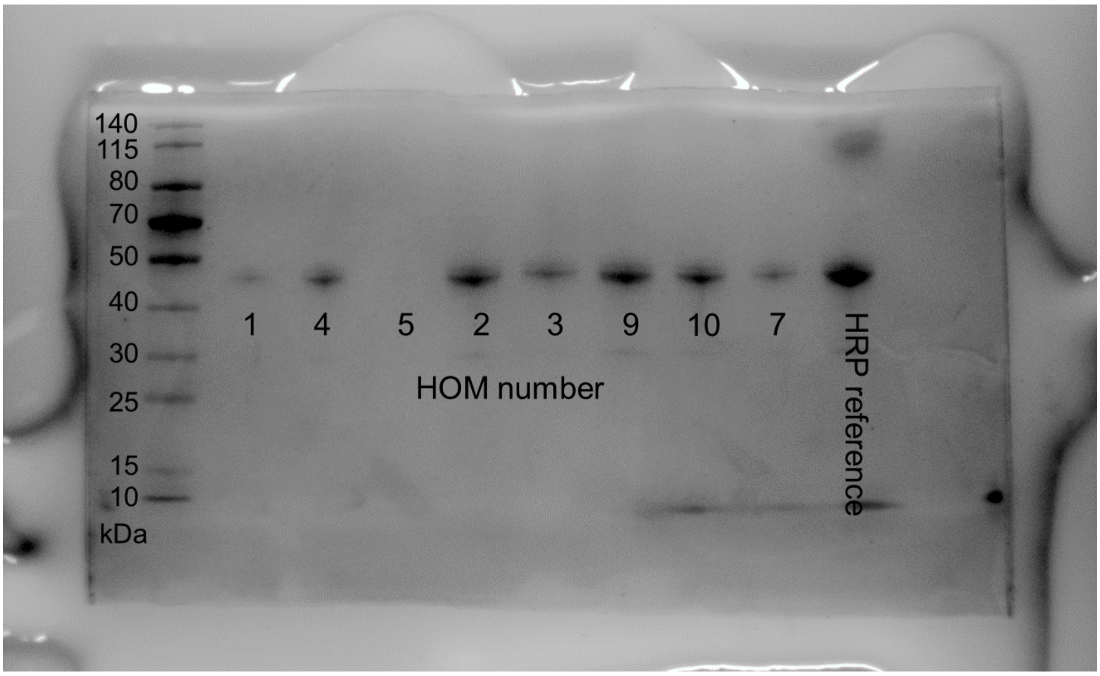

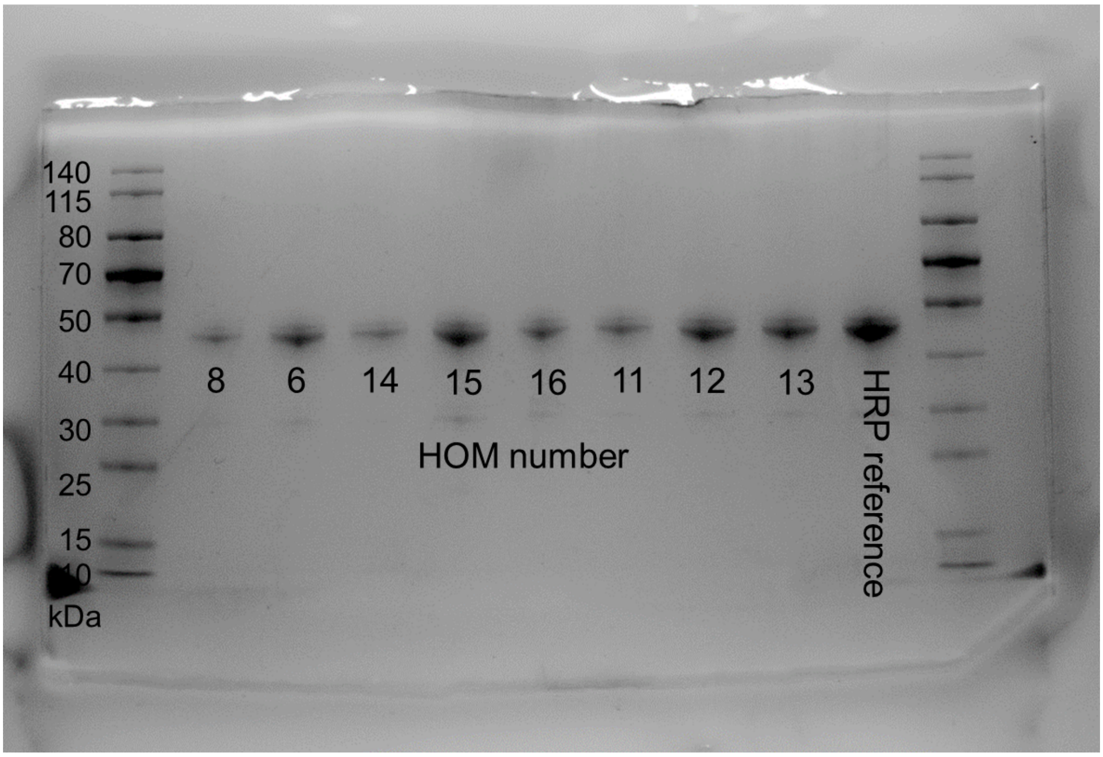

Supplementary Data 2a. Uncropped SDS-PAGE for protein supernatant in measuring loading capacity of HOM-1 to 16 for HRP.

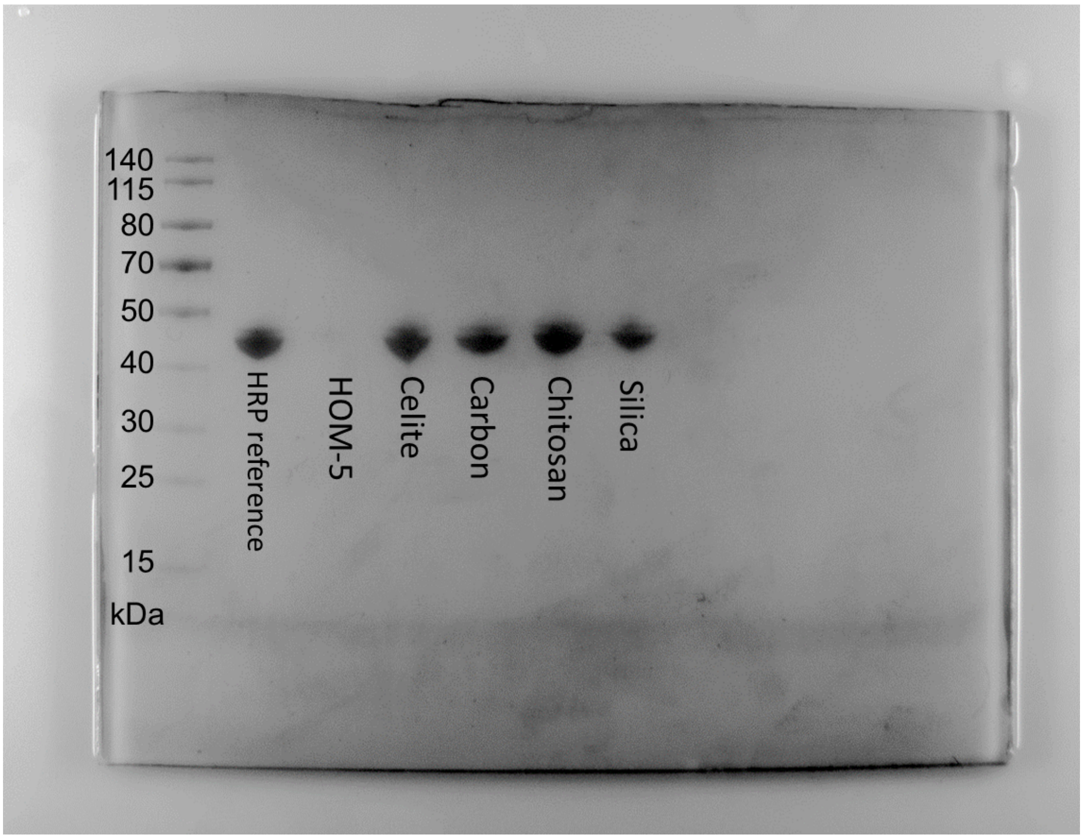

Supplementary Data 2b. Uncropped SDS-PAGE image of protein supernatant without treatment (the first lane) and after loading with various materials (the 2nd to the 6th lanes).

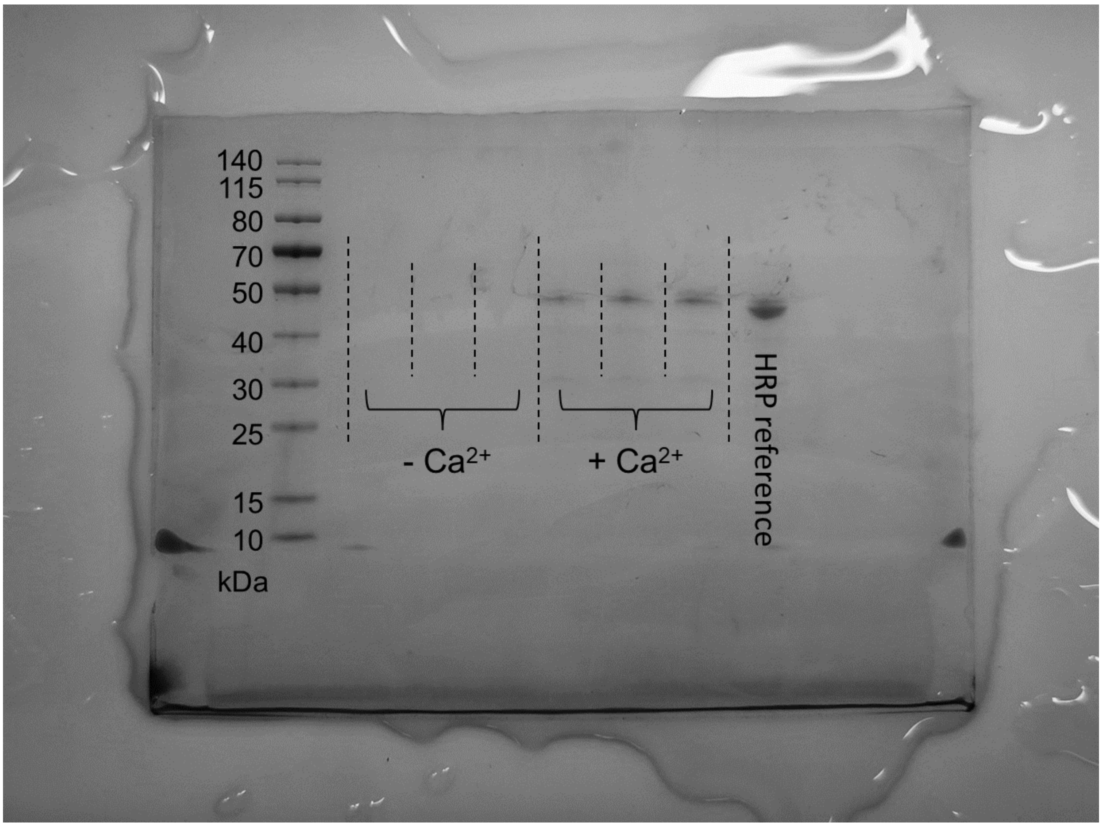

Supplementary Data 2c. Uncropped SDS-PAGE for protein supernatant of HOM-5 with or without Calcium ion (three parallel experiments).

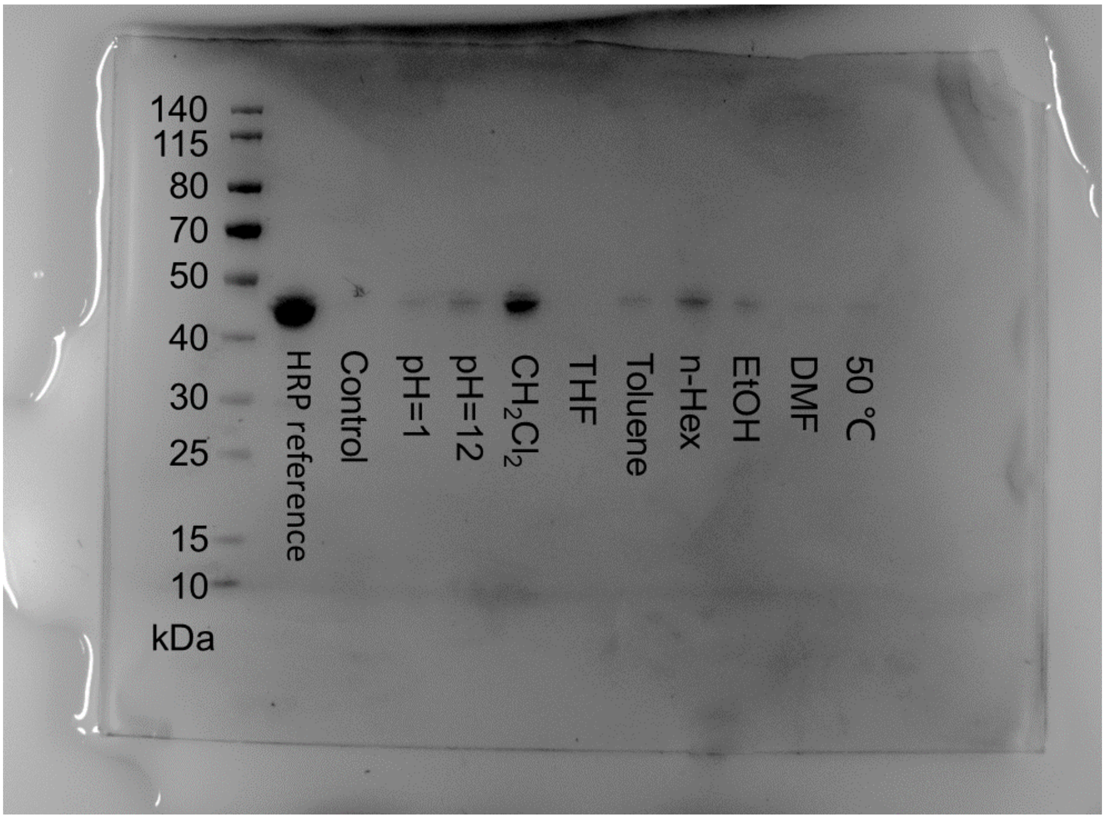

Supplementary Data 2d. Uncropped SDS-PAGE image of protein supernatant without carrier (the first lane) and with HOM-5 after various treatment (the 2nd to the 11th lanes).
